# Acid-Catalyzed Oxy-aminomethylation of Styrenes

**DOI:** 10.1021/acscatal.3c05342

**Published:** 2024-01-02

**Authors:** Marian Guillén, Sensheng Liu, C. David Díaz-Oviedo, Martin Klussmann, Benjamin List

**Affiliations:** Max-Planck-Institut für Kohlenforschung, Kaiser-Wilhelm-Platz 1, 45470, Mülheim an der Ruhr, Germany

**Keywords:** olefin, *sym*-trioxane, sulfonamide, carbamate, amino alcohol, aza-Prins reaction, iminium ion

## Abstract

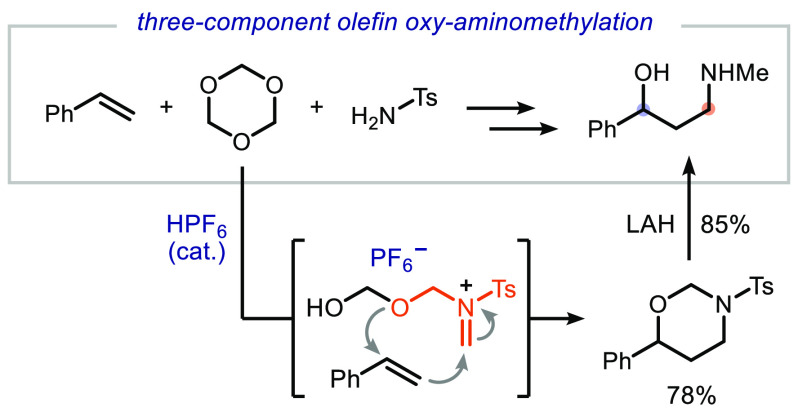

We report a strong Brønsted acid-catalyzed three-component oxy-aminomethylation of styrenes with *sym*-trioxane and sulfonamides or carbamates. This transformation provides a variety of 1,3-oxazinanes in moderate to good yields under mild reaction conditions. The obtained heterocycles can be readily transformed into the corresponding 1,3-amino alcohols, which are useful building blocks for the synthesis of pharmaceutically relevant molecules. Mechanistic studies suggest the intermediacy of an in situ formed 1,3,5-dioxazinane and a subsequent reaction with the olefin.

Olefins are abundant and inexpensive starting materials for chemical synthesis. Consequently, the chemistry of alkene functionalization has received much attention in the last century, which has led to the development of fundamental reactions such as the Ziegler–Natta polymerization, the Diels–Alder cycloaddition, or the Mizoroki–Heck reaction, and hydrogenations, to just name a few.^1−6^ The direct heterofunctionalization of C=C bonds is a particularly powerful strategy for the transformation of feedstock chemicals into highly functionalized compounds, as exemplified by reactions such as dihydroxylation and aminohydroxylation, hydroamination, and many others.^7−9^ From these type of transformations, the Prins reaction and the aza-version thereof stand out for enabling the direct synthesis of 1,3-difunctionalized moieties, which are widely present in pharmaceutically relevant molecules.^10^ The Prins reaction has been extensively documented, and several catalytic methodologies are available nowadays.^11,12^ Analogously, the aza-Prins reaction of an alkene, formaldehyde, and ammonia represents a straightforward approach to transform olefins into 1,3-amino alcohols. Formally, this would involve the formation of a formaldehyde-derived imine, which is subsequently attacked by the nucleophilic olefin, and the intermediate β-amino carbocation could be trapped with water to deliver the corresponding 1,3-amino alcohol (Figure 1A). Surprisingly, despite the significant synthetic potential of this transformation, it remains hitherto underdeveloped with only a few reports in the literature to date, either with extremely limited scope^13,14^ or biased substrates with tethered nucleophiles to trap highly reactive intermediates,^15^ as well as with the use of preformed electrophiles that lead to a two-component process.^16^ We aimed, therefore, to contribute to closing this gap and present here our work on the Brønsted acid-catalyzed three-component reaction of aryl olefins, formaldehyde, and ammonia surrogates, such as sulfonamides or carbamates (Figure 1B). The proposed transformation not only contributes to the field of olefin functionalization but also benefits from the wide availability of sulfonamides and carbamates, which are common and widely used pharmacophores.

**Figure 1:**
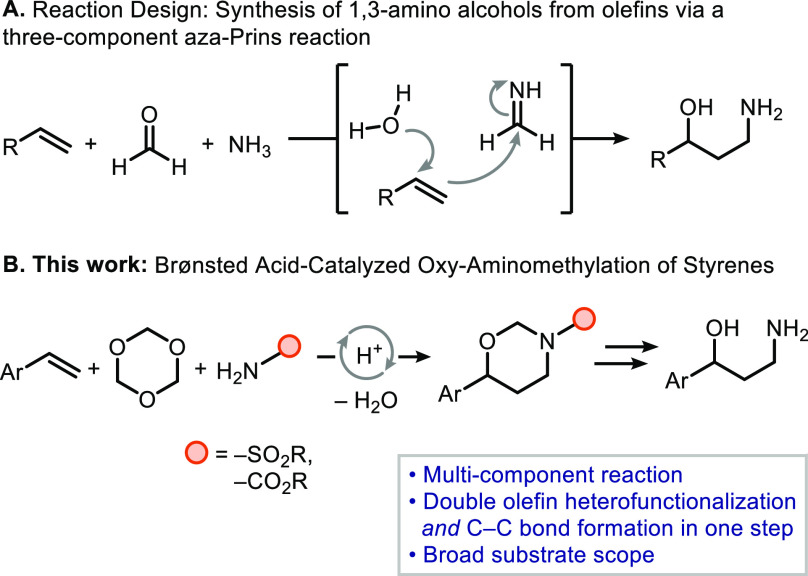
(A) Reaction design: multicomponent aza-Prins reaction of olefins, formaldehyde, and ammonia. (B) Our approach: Brønsted acid-catalyzed oxy-aminomethylation reaction of aryl olefins.

At the onset of our investigation, we studied the reaction of styrene (**1a**), *sym*-trioxane (**2**) as a formaldehyde source, and *p*-toluenesulfonamide (**3a**) (Table 1 and Table SI-1) by testing the performance of several common Brønsted acids as catalysts. When substrates **1a**, **2**, and **3a** were mixed in a 1:1.5:3 ratio, the use of a weak Brønsted acid, such as acetic acid (AcOH), in catalytic amounts led to the formation of 1,3-oxazinane **4a** in poor yield after 24 h at 60 °C (Table 1, entry 1).

**Table 1:**
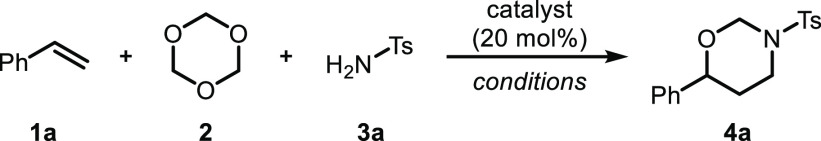
Reaction Development^a^

entry	catalyst	solvent	temperature (°C)	yield **4a** (%)
1	AcOH	CH_2_Cl_2_	60	<5
2	*p*-TsOH	CH_2_Cl_2_	60	38
3	TfOH	CH_2_Cl_2_	60	32
4	Tf_2_NH	CH_2_Cl_2_	60	10
5	HPF_6_	CH_2_Cl_2_	60	58
6	none	CH_2_Cl_2_	60	0
7	HPF_6_	CHCl_3_	60	**78** (68^b^)
8	HPF_6_	CHCl_3_	25	12
9^c^	HPF_6_	CHCl_3_	60	7
10^d^	HPF_6_	CHCl_3_	60	<5

a Conditions: **1a** (0.2 mmol), **2** (1.5 equiv), **3a** (3.0 equiv), catalyst (20 mol %), solvent (2 mL), 24 h. All yields referred to are ^1^H NMR yield (internal standard: 1,3,5-trimethoxybenzene); isolated yield in parentheses.

b Performed on 10 mmol scale.

c Using paraformaldehyde (4.5 equiv) instead of trioxane.

d Using formalin (4.5 equiv) instead of trioxane. Ts = 4-MeC_6_H_4_SO_2_. See the Supporting Information for further details.

The use of either *p*-toluenesulfonic acid (*p*-TsOH) or trifluoromethanesulfonic acid (TfOH) resulted in higher conversions, thereby affording the product in 38% and 32% yield, respectively (entries 2 and 3). As expected, the choice of catalyst plays a crucial role since other established strong Brønsted acids were found to be less efficient. Bis(trifluoromethane)sulfonimide (Tf_2_NH) as catalyst led to a complex mixture and gave only 10% of the desired product **4a** (entry 4). The use of the strong Brønsted acid hexafluorophosphoric acid (HPF_6_) in catalytic amounts led to the formation of 1,3-oxazinane **4a** in 58% yield after 24 h at 60 °C (entry 5). In the absence of acid catalyst, no product was observed (entry 6). Upon screening several solvents, the use of chloroform proved beneficial and resulted in an increased yield (78%, entry 7). Remarkably, the HPF_6_-catalyzed reaction also proceeded at room temperature, although with a considerably reduced rate (entry 8). Satisfactorily, the reaction could be performed on a larger scale (10 mmol of olefin) to provide 2.2 g of product **4a** (isolated yield of 68%, entry 7). *sym*-Trioxane proved to be an adequate source of formaldehyde given the significant decrease in yield when paraformaldehyde or formalin were used (entries 9–10).

With these optimized conditions, we explored the scope of the transformation (Table 2A). Regarding the olefin component, we found that terminal styrenes containing either weakly electron-donating (Me, *t*-Bu, CH_2_Cl) or electron-withdrawing groups (F, Cl, Br) as para substituents, afforded the corresponding 1,3-oxazinanes in moderate to good yields (**4b**–**4d** and **4e**–**4g**). More electron-deficient olefins, though, proved to be nonreactive under the tested conditions (see the Supporting Information for details). Styrenes with *meta*- or *ortho*-substituted rings were also suitable substrates, as is the case of **1h** and **1i**; remarkably, olefin **1j** possessing a challenging double ortho substitution pattern could also be converted into the corresponding 1,3-oxazinane **4j** in moderate yield. The methodology could also be applied to a naphthyl-substituted olefin, which formed product **4k** in 38% yield. Cyclic and/or internal aryl olefins also proved to be suitable substrates for the transformation. For example, 1*H*-indene reacted to form product **4l** exclusively as the cis diastereomer. Conversely, dialin reacted to produce **4m** in 32% yield as a diastereomeric mixture (cis/trans ratio = 1:1). The internal noncyclic olefin **1n** reacted to produce 1,3-oxazinane **4n** as a mixture of trans and cis isomers in a 2:1 ratio, whereas the isomeric cis olefin was not converted under the same reaction conditions. Finally, our methodology proved to be highly selective for aryl olefins, as observed for substrate **1l**, which contained both an alkyl olefin and an aryl olefin, where only product **4o** was formed in 53% yield. Several alkyl olefins did not react under the reaction conditions (see the Supporting Information for details).

**Table 2:**
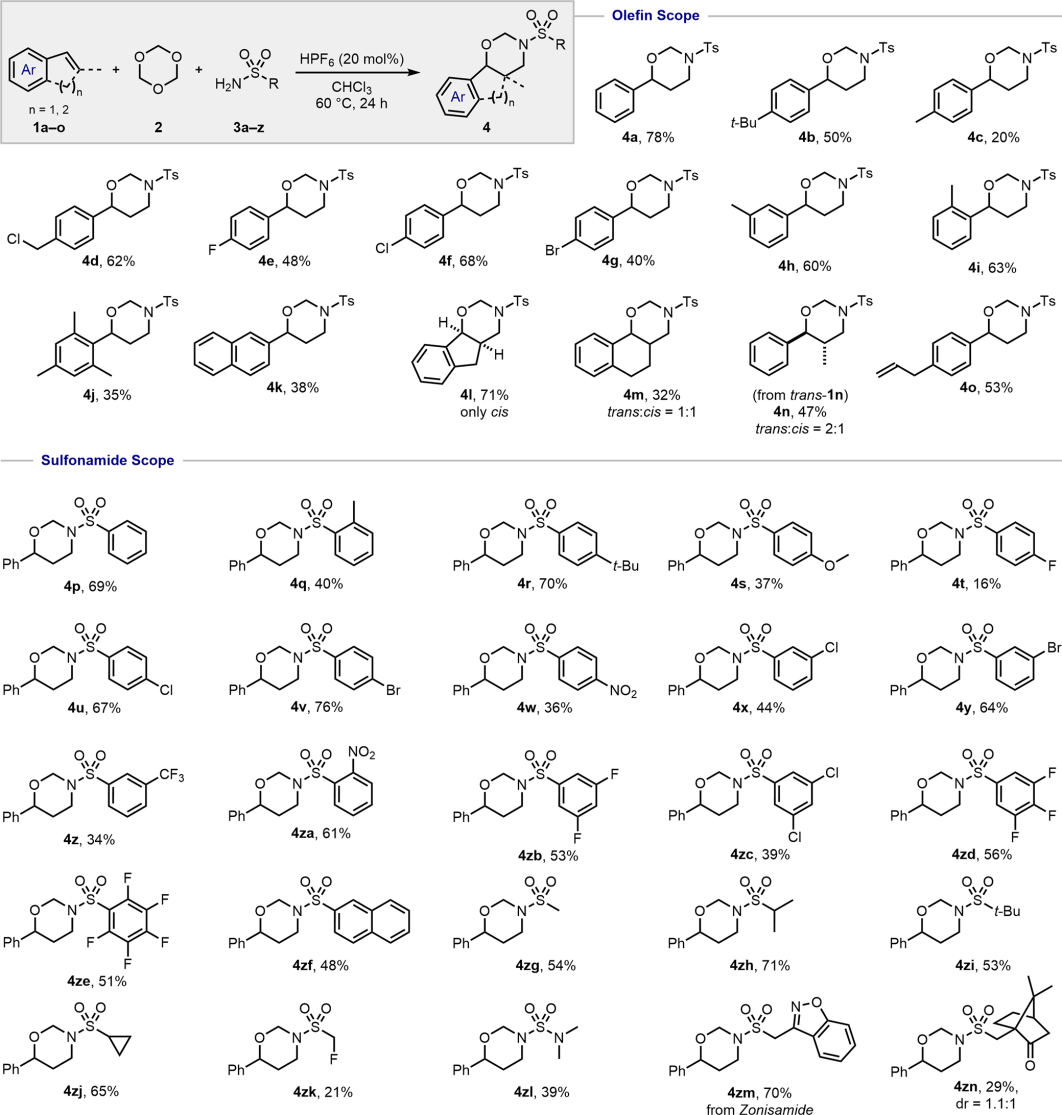
Olefin/Sulfonamide Scope^a^

a All the reactions were conducted with **1** (0.2 mmol), **2** (1.5 equiv), **3** (3.0 equiv), and aq HPF_6_ 55% (20 mol %) in CHCl_3_ (2 mL) at 60 °C for 24 h. The data reported here corresponds to the isolated yields.

Subsequently, the scope with respect to various sulfonamides was explored considering the broad availability of these compounds. As shown in Table 2B, benzenesulfonamide itself, as well as derivatives thereof containing electron-donating (Me, *t*-Bu, OMe) substituents, represent suitable substrates (see products **4p**–**4s**). Furthermore, a broad variety of benzenesulfonamides with electron-withdrawing groups (F, Cl, Br, NO_2_, CF_3_) in a variety of substitution patterns on the aromatic ring could also be transformed to the corresponding 1,3-oxazinanes (**4t**–**4ze**) in moderate yields. Naphthalene-derived sulfonamide also reacted to produce the corresponding heterocycle **4zf**. The reaction also proceeded with aliphatic sulfonamides to provide the corresponding products (**4zg**–**4zj**) in good yields. It is worth mentioning that the reaction with fluoromethanesulfonamide led to the formation of the expected oxazinane **4zk** in 21% yield, along with 46% of 4-phenyl-1,3-dioxane, the product of the Prins reaction between olefin and formaldehyde, probably because of the decreased nucleophilic character of the sulfonamide, which hinders its reaction with trioxane to form the oxy-aminomethylating species. It is worth mentioning that products **4za** and **4zg** contain nosylate and mesylate groups, respectively, which are frequently used protecting groups and for which deprotection strategies are known.^17−19^ Using *N*,*N*-dimethylsulfamide, desired product **4zl** could be isolated in 39% yield. Given the broad spectrum of sulfonamide drugs,^20,21^ we wondered if our methodology could be applied for the derivatization of pharmaceutically active molecules containing that functional group as a late-stage modification approach. Zonisamide, a medication used to treat the symptoms of epilepsy and Parkinson’s disease,^22^ reacted to give product **4zm** in 70% yield. Considering the commercial availability of enantiopure camphorsulfonic acid and derivatives and their extended use as chiral auxiliaries and resolving agents,^23−25^ we decided to make use of (1*S*)-10-camphorsulfonamide in our three-component transformation. The reaction afforded product **4zn** in 29% yield, although with negligible diastereoselectivity (dr = 1.1:1).

Importantly, carbamates proved also to be suitable substrates for the multicomponent transformation. When phenyl carbamate **5a** was used as the ammonia surrogate, product **6a** was obtained in 34% yield (Table 3). By virtue of the extended use of carbamates (Boc, Cbz, Fmoc, among others) as protecting groups in the synthesis of peptides, we considered the potential of *N*-carbamoyl-protected oxazinanes for the preparation of modified/unnatural peptide derivatives.^26,27^ Gratifyingly, both benzyl carbamate (**5b**) and 9-fluorenylmethyl carbamate (**5c**) underwent the HPF_6_-catalyzed three-component reaction with *sym*-trioxane and styrene **1a** to afford the corresponding *N*-Cbz-protected (**6b**) and the *N*-Fmoc-protected (**6c**) 1,3-oxazinanes (23% and 37% yield, respectively; see Table 3). Similarly, terminal styrenes **1b**, **1f**, **1h**, and **1p** also proved to be suitable substrates for this methodology. In our attempt to prepare an *N*-Boc-protected oxazinane, *tert*-butyl carbamate proved to be incompatible with the strongly acidic catalyst (see the Supporting Information for more details).

**Table 3:**
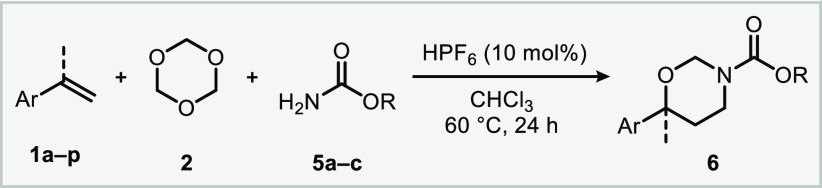

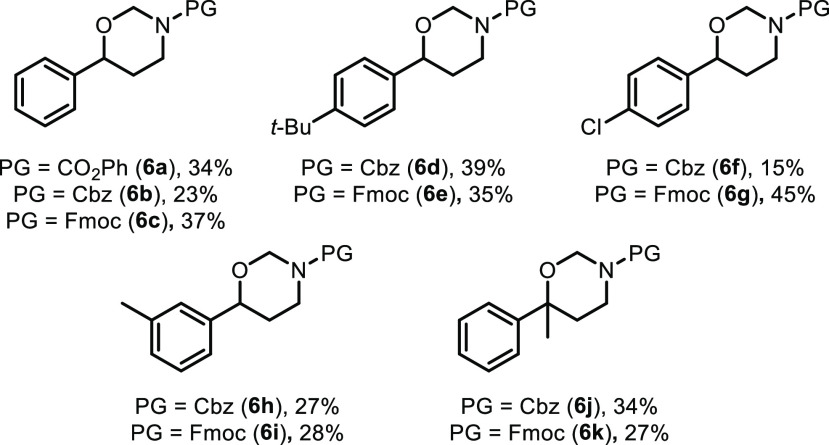
Reaction Using Carbamates^a^

a Conditions: **1** (0.5 mmol), **2** (1.5 equiv), **5** (1.5 equiv), and aq HPF_6_ 55% (10 mol %) in CHCl_3_ (0.5 mL) at 60 °C for 24 h.

The obtained oxy-aminomethylation products already display the connectivity of the desired 1,3-amino alcohols, which require only the removal of the *N,O*-acetal functionality and the nitrogen protecting group. Choosing appropriate conditions allowed the selective removal of these moieties. For example, by refluxing with HCl in methanol,^28,29^ 1,3-oxazinane **4a** underwent a clean *N,O*-acetal ring opening to give access to *N*-Ts-protected amino alcohol **7a** in 89% yield (Scheme 1a). Treatment of **4a** with Mg powder and sonication followed by the *N,O*-acetal ring opening procedure afforded the corresponding free 1,3-amino alcohol **7b** in 75% yield over the two steps (Scheme 1b). Furthermore, 1,3-oxazinane **4a** can be readily reduced to the corresponding *N*-methyl species, a characteristic feature of several pharmaceutically active substances, such as atomoxetine, fluoxetine and nisoxetine.^30^ When refluxed with diisobutylaluminum hydride (DIBAL) in THF, heterocycle **4a** underwent a clean reductive ring cleavage to produce *N*-Ts-protected amino alcohol **7c** in 80% yield (Scheme 1c). Importantly, refluxing our reaction product **4a** in the presence of LiAlH_4_ enabled direct conversion into *N*-methyl amino alcohol **7d** in 85% yield (Scheme 1d).

**Scheme 1:**
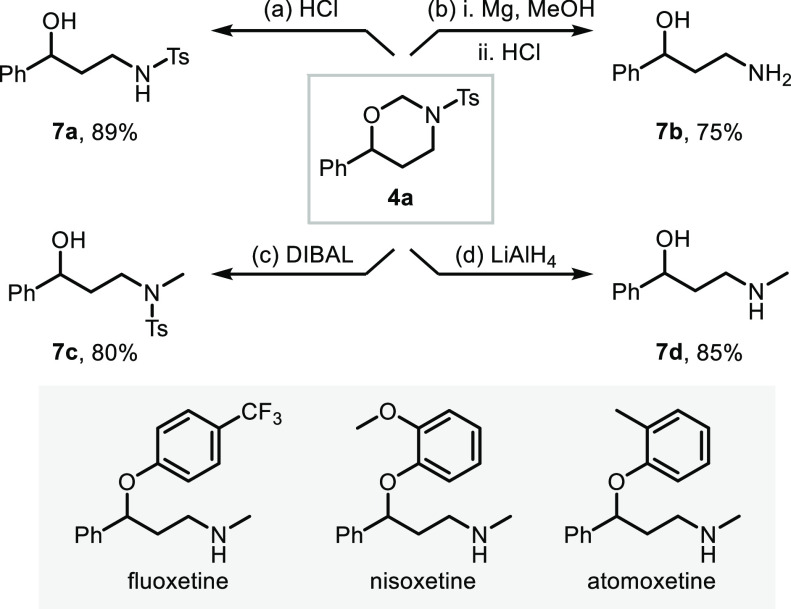
Derivatization of 1,3-Oxazinane to the Corresponding 1,3-Amino alcohol Reaction conditions: (a) concd HCl 37% (2 equiv), MeOH, reflux, 6 h, then 95 °C, 1 h. (b) (i) Mg powder (5 equiv), MeOH/THF 2.5:1 v/v, sonication, rt, 3 h; (ii) concd HCl 37% (2 equiv), MeOH, reflux, 6 h, then 95 °C, 1 h. (c) DIBAL (5 equiv), THF, reflux overnight. (d) LiAlH_4_ (5 equiv), THF, reflux 3 days.

Next, we dedicated our efforts to gain insight into the mechanistic pathway of the three-component olefin oxy-aminomethylation. To shed light on the actual nature of the electrophile, we conducted several experiments in the absence of olefin. The acid-catalyzed reaction of *sym*-trioxane (**2**) and sulfonamide **3a** results in the formation of several sulfonamide-formaldehyde condensation products, such as the monosubstituted 1,3,5-dioxazinane **8a**, the disubstituted 1,3,5-oxadiazinane **8b**, and the trisubstituted 1,3,5-triazinane **8c**. Also, in the presence of catalytic HPF_6_ at 60 °C, both **8b** and **8c** reacted with *sym*-trioxane to form **8a**, which indicates a dynamic equilibrium between all of these heterocycles in our three-component reaction (see Scheme 2 and the Supporting Information for further details). When each one of these condensation products was reacted with styrene **1a** in the presence of catalytic amounts of HPF_6_, only **8a** showed significant reactivity forming **4a** in 70% yield, thereby suggesting **8a** to be the actual reactive precursor in the annulation with styrene (Scheme 2).

**Scheme 2:**
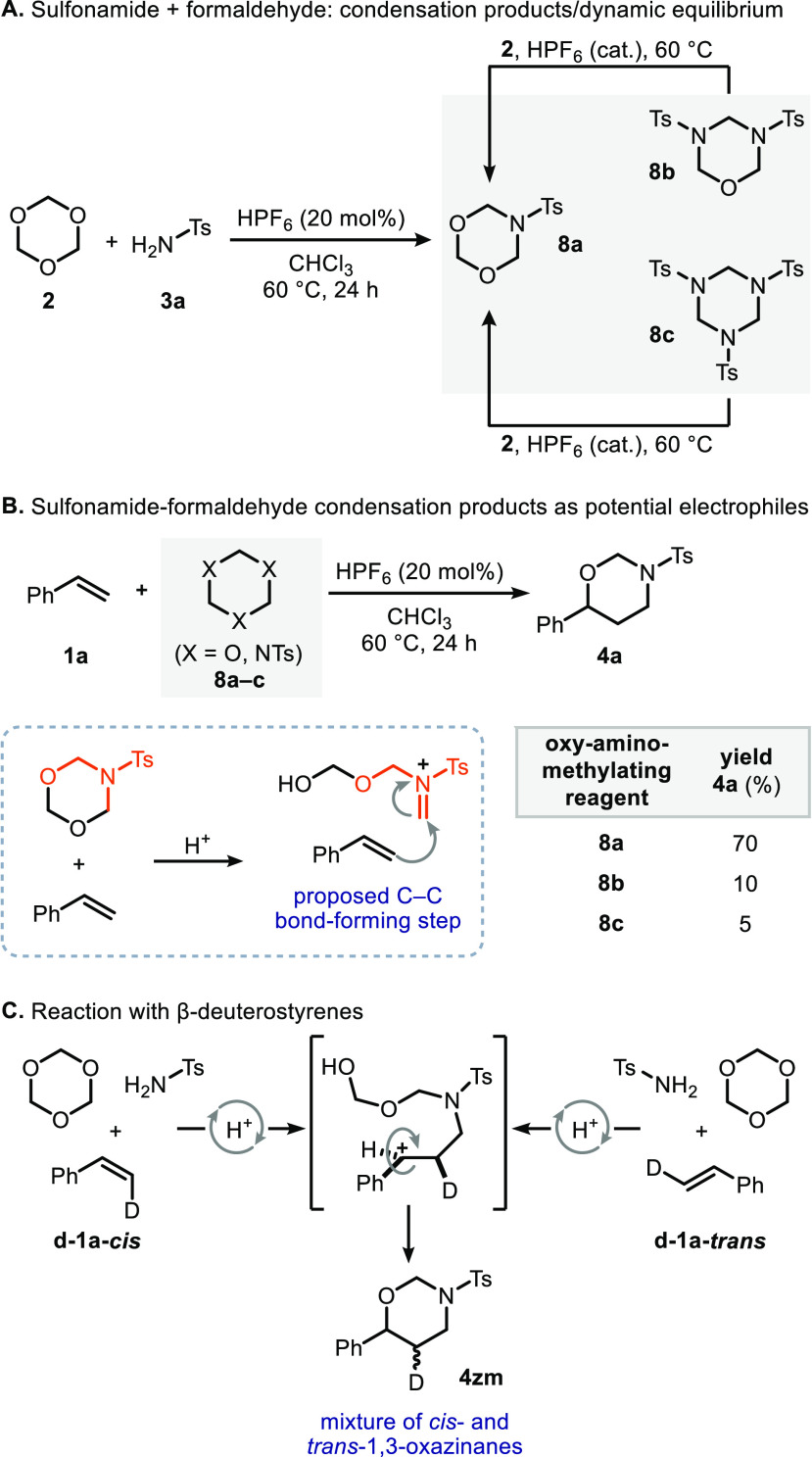
Toward the Elucidation of the Actual Mechanism in the Three-Component Transformation

To gain further understanding of the reaction mechanism, we studied the three-component reaction using β-deuterium-labeled styrenes (**d-1a-*****cis*** and **d-1a-*****trans***, respectively) as substrates.^31^ The HPF_6_-catalyzed reaction of these olefins led in both cases to *cis*/*trans* mixtures of the corresponding 1,3-oxazinane, which is consistent with the intermediacy of a benzylic cation.

On the basis of these results, we can now propose a mechanism for our three-component reaction (Scheme 3). Accordingly, in the first part of the reaction, *sym*-trioxane **2** engages in an acid-catalyzed condensation with *p*-toluenesulfonamide **3a** to form several cyclic products under a dynamic equilibrium from which 1,3,5-dioxazinane **8a** is proposed to be the actual oxy-aminomethylating precursor. Subsequent protonation with the Brønsted acid catalyst produces highly reactive intermediate **I-1** that, upon ring opening, forms an *N*-sulfonyl iminium ion. This highly electrophilic species undergoes an aza-Prins reaction with olefin **1a** (see **I-2**) to generate benzylic carbocation **I-3** followed by ring closure and release of formaldehyde to give the desired product **4a**.

**Scheme 3:**
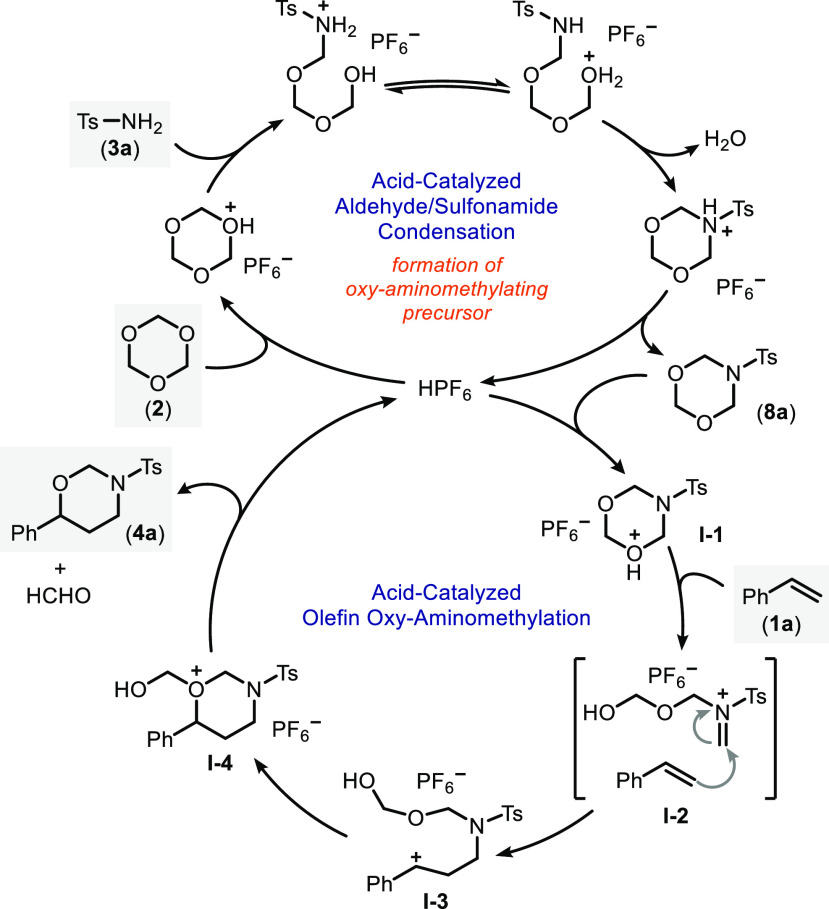
Proposed Reaction Mechanism

In conclusion, a Brønsted acid-catalyzed three-component oxy-aminomethylation of olefins was developed. 1,3-Oxazinane derivatives are obtained from the reaction of a wide range of sulfonamides/carbamates with *sym*-trioxane and aryl olefins by using the strong Brønsted acid HPF_6_ as catalyst. Preliminary mechanistic studies suggest the product from partial aldehyde/sulfonamide condensation to be a key intermediate for the oxy-aminomethylation reaction. The obtained 1,3-oxazinanes can be easily transformed into valuable 1,3-amino alcohols, which are highly demanded building blocks in synthesis.

## Supporting Information Available

Experimental details and analytical data for all new compounds and NMR spectra (PDF)
