# Supplementary material for: Acid-Catalyzed Oxy-aminomethylation of Styrenes
Source: ACS Catal. 2024 Jan 2;14(2):751–6. doi: 10.1021/acscatal.3c05342 (PMC10804369; doi:10.1021/acscatal.3c05342)
Supplement: Supplementary file 1 — cs3c05342_si_001.pdf [file cs3c05342_si_001.pdf]

Supporting Information for

# Acid-Catalyzed Oxy-Aminomethylation of Styrenes

Marian Guillén, Sensheng Liu, C. David Díaz-Oviedo, Martin Klussmann, and Benjamin List*

Max-Planck-Institut für Kohlenforschung, Kaiser-Wilhelm-Platz 1, 45470 Mülheim an der Ruhr, Germany
*E-mail: list@kofo.mpg.de

1. Materials and Methods .... 2
2. Substrate Synthesis .... 3
3. Reaction Development .... 6
4. Acid Catalyzed Oxy-Aminomethylation of Styrenes .... 8
5. Synthesis of 1,3-Amino Alcohols: Deprotection and Ring Opening .... 23
6. Current Limitations .... 25
7. Mechanistic Studies .... 26
   7.1. Preliminary Monitoring of the Three-Component Reaction .... 26
   7.2. On the Oxy-Aminomethylating Species: Experiments on the Formaldehyde-Sulfonamide Condensation .... 27
   7.3. Stereospecificity: experiments with β-deuterostyrenes .... 29
8. Copies of NMR spectra .... 30
9. References .... 90

# 1. Materials and Methods

Unless otherwise stated, all reactions were magnetically stirred and conducted in oven-dried (90 °C) or flame-dried glassware in anhydrous solvents under argon, applying standard Schlenk techniques. Solvents and liquid reagents, as well as solutions of solid or liquid reagents were added via syringes, stainless steel or polyethylene cannulas through rubber septa or through a weak argon counter-flow. Solid reagents were added through a weak argon counter-flow. Cooling baths were prepared in Dewar vessels, filled with ice/water (0 °C), cooled acetone (< –78 °C) or dry ice/acetone (–78 °C). Heated oil baths were used for reactions requiring elevated temperatures. Solvents were removed under reduced pressure at 40 °C using a rotary evaporator, and unless otherwise stated, the remaining compound was dried in high vacuum ($10^{-3}$ mbar) at room temperature. All given yields are isolated yields of chromatographically and NMR-spectroscopically pure materials, unless otherwise stated.

Chemicals were purchased from commercial suppliers (including abcr, Acros, Alfa Aesar, Fluorochem, Merck, and TCI) and used without further purification unless otherwise stated.

Solvents (CyH, $CH_2Cl_2$, $Et_2O$, THF, toluene) were dried by distillation from an appropriate drying agent in the technical department of the Max-Planck-Institut für Kohlenforschung and received in Schlenk flasks under Ar.[1] Other anhydrous solvents were purchased from commercial suppliers and used as received.

Reactions were monitored by thin layer chromatography (TLC) on silica gel pre-coated plastic sheets (0.2 mm, Macherey-Nagel). Visualization was accomplished by irradiation with UV light (254 nm and 366 nm) and/or phosphomolybdic acid (PMA) stain and/or Cerium Ammonium Molybdate (CAM) stain and/or permanganate stain.

Column chromatography was carried out using Merck silica gel (60 Å, 230−400 mesh, particle size 0.040−0.063 mm) using technical grade solvents. Elution was accelerated using compressed air. Automated column chromatography was conducted on a Biotage® IsoleraTM ISO-4SW instrument, using SNAP Ultra HP-SphereTM 25 µm chromatography cartridges. All fractions containing a desired substance were combined and concentrated in vacuo, then redissolved in an appropriate solvent and filtered through cotton to remove silica residues.

$^{1}H$, $^{13}C$, $^{19}F$, $^{31}P$ nuclear magnetic resonance (NMR) spectra were recorded on a Bruker AV-500, AV-400 or DPX-300 spectrometer in a suitable deuterated solvent. The solvent employed and respective measuring frequency are indicated for each experiment. Chemical shifts are reported with $Me_4Si$ serving as a universal reference of all nuclides and with two or one digits after the comma. The resonance multiplicity is described as s (singlet), d (doublet), t (triplet), q (quadruplet), p (pentet), hept (heptet), m (multiplet), and b (broad). All spectra were recorded at 298 K unless otherwise noted, processed with the program MestReNova 14.2, and coupling constants are reported as observed. The residual deuterated solvent signal relative to $Me_4Si$ was used as the internal reference in $^{1}H$ NMR spectra (e.g. $CDCl_3$ = 7.26 ppm) and are reported as follows: chemical shift in ppm (multiplicity, coupling constant *J* in Hz, number of protons). $^{13}C$, $^{19}F$, $^{31}P$ NMR spectra were referenced according to Ξ-values (IUPAC recommendations 2008)[2] relative to the internal references set in $^{1}H$ NMR spectra (e.g. $^{13}C$: $Me_4Si$, $^{19}F$: $CCl_3F$, $^{31}P$: $H_3PO_4$; each 0.00 ppm). All spectra are broadband decoupled unless otherwise noted.

Electron impact (EI) mass spectrometry (MS) was performed on a Finnigan MAT 8200 (70 eV) or MAT 8400 (70 eV) spectrometer. Electrospray ionization (ESI) mass spectrometry was conducted on a Bruker ESQ 3000 spectrometer. High resolution mass spectrometry (HRMS) was performed on a Finnigan MAT 95 (EI) or Bruker APEX III FTMS (7T magnet, ESI). The ionization method and mode of detection employed is indicated for the respective experiment and all masses are reported in atomic units per elementary charge (*m/z*) with an intensity normalized to the most intense peak.

## 2. Substrate Synthesis

Substrates **1a–1n**, **2**, **3a–z** and **5a–c** were purchased from Sigma-Aldrich or abcr. Other substrates were prepared by methods indicated below.

**1-allyl-4-vinylbenzene (1o)**

*(Following a reported procedure[3])*: A two-necked round-bottom flask under argon atmosphere was charged with $Pd_2(dba)_3$ (4.6 mg, 5 µmol, 0.1 mol%), 4-vinylphenyl boronic acid (888 mg, 6 mmol, 1.2 equiv.) and 1,4-dioxane (6.5 mL). Triphenylphosphite (2.5 µL, 10 µmol, 0.2 mol%) and allyl alcohol (290 mg, 5 mmol) were added and the mixture was heated at 80 °C (oil bath) for 6 h. After cooling to rt, the reaction mixture was diluted with MTBE (50 mL) and washed with brine (1 x 30 mL), dried over anhydrous $Na_2SO_4$, filtered and concentrated under reduced pressure. Purification by flash column chromatography on silica gel (*n*-pentane) afforded the corresponding olefin **1o**.

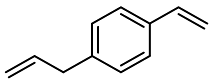

Obtained as a colorless liquid (480 mg, 67%).
$^1$H NMR (501 MHz, $CD_2Cl_2$): δ 7.35 (d, *J* = 8.1 Hz, 2H), 7.16 (d, *J* = 8.2 Hz, 2H), 6.71 (dd, *J* = 17.6, 10.9 Hz, 1H), 5.97 (ddt, *J* = 16.9, 10.1, 6.7 Hz, 1H), 5.72 (dd, *J* = 17.6, 1.0 Hz, 1H), 5.20 (dd, *J* = 10.9, 1.0 Hz, 1H), 5.13 – 5.03 (m, 2H), 3.38 (d, *J* = 6.8 Hz, 2H).
$^{13}$C NMR (126 MHz, CD2Cl2): δ 140.4 (C), 137.9 (CH), 137.0 (CH), 135.9 (C), 129.1 (CH), 126.6 (CH), 115.9 ($CH_2$), 113.3 ($CH_2$), 40.3 ($CH_2$). Spectroscopic data was consistent with the values reported in the literature.[3]

***cis*-Styrene-(β)-*d* (*d*-1a-*cis*)**

*(Following a reported procedure[3])*: A Schlenk flask under argon atmosphere was charged with phenylacetylene-*d*[4] (245 mg, 2.4 mmol) and dry $CH_2Cl_2$ (7.5 mL). The flask was covered with aluminum foil and the mixture was cooled to 0 °C. Schwartz's Reagent (680 mg, 2.6 mmol, 1.1 equiv.) was then added in two equal portions in rapid succession (over 2 min). The mixture was allowed to stir at 0 °C for 15 min, then the cold bath was removed and the stirring was continued at rt in the dark for 2 h. The flask was cooled to 0 °C, and the mixture was quenched with water (0.35 mL, 19.2 mmol, 8 equiv.) and stirred vigorously at rt for 3 h. The mixture was diluted with $CH_2Cl_2$ (3 mL), followed by the addition of anhydrous $Na_2SO_4$ and filtration. The filtrate was concentrated under reduced pressure (400 mbar, water bath of rotavap at 25 °C; no heating, product is volatile) until 1 mL remained. *n*-Pentane (2 mL) was added and the mixture was filtered over a Celite® pad to remove the white precipitate; the filter cake was rinsed with *n*-pentane and the filtrate was again concentrated under reduced pressure (400 mbar, 25 °C). Purification by flash column chromatography on silica gel (*n*-pentane; removal of solvent on rotavap at 400 mbar, 25 °C) afforded the corresponding olefin ***d*-1a-*cis***.

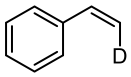

Obtained as a colorless liquid (75 mg, 30%). Approx. 99% D-incorporation.
$^1$H NMR (501 MHz, $CDCl_3$) δ 7.45 – 7.40 (m, 2H), 7.37 – 7.31 (m, 2H), 7.26 (tt, *J* = 7.3, 1.3 Hz, 1H), 6.72 (dt, *J* = 10.9, 2.6 Hz, 1H), 5.23 (d, *J* = 10.9 Hz, 1H). Spectroscopic data was consistent with the values reported in the literature.[3]

***trans*-Styrene-(β)-*d* (*d*-1a-*trans*)**

*(Following a reported procedure[3])*: A Schlenk flask under argon atmosphere was charged with phenylacetylene (306 mg, 3.0 mmol) and dry $CH_2Cl_2$ (7.5 mL). The flask was covered with aluminum foil and the mixture was cooled to 0 °C. Schwartz's Reagent (850 mg, 3.3 mmol, 1.1 equiv.) was then added in two equal portions in rapid succession (over 2 min). The mixture was allowed to stir at 0 °C for 15 min, then the cold bath was removed and the stirring was continued at rt in the dark for 2 h. The flask was cooled to 0 °C, and the mixture was quenched with $D_2O$ (0.45 mL, 99.9% D, 24.9 mmol, 8.3 equiv.) and stirred vigorously at rt for 3 h. The mixture was diluted with $CH_2Cl_2$ (3 mL), followed by the addition of anhydrous $Na_2SO_4$ and filtration. The filtrate was concentrated under reduced pressure (400 mbar,

water bath of rotavap at 25 °C; no heating, product is volatile) until 1 mL remained. *n*-Pentane (2 mL) was added and the mixture was filtered over a Celite® pad to remove the white precipitate; the filter cake was rinsed with *n*-pentane and the filtrate was again concentrated under reduced pressure (400 mbar, 25 °C). Purification by flash column chromatography on silica gel (*n*-pentane; removal of solvent on rotavap at 400 mbar, 25 °C) afforded the corresponding olefin ***d*-1a-*trans***.

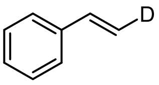

Obtained as a colorless liquid (95 mg, 30%). Approx. 91% D-incorporation.
$^1$H NMR (501 MHz, $CDCl_3$) δ 7.45 – 7.40 (m, 2H), 7.37 – 7.31 (m, 2H), 7.26 (tt, *J* = 7.3, 1.3 Hz, 1H), 6.72 (dt, *J* = 17.5, 1.5 Hz, 1H), 5.75 (d, *J* = 17.7 Hz, 1H). Spectroscopic data was consistent with the values reported in the literature.[3]

**5-tosyl-1,3,5-dioxazinane (8a)**

*(Following a reported procedure*[5]*)*: To a stirred solution of *sym*-trioxane (1.80 g, 20 mmol, 2 equiv.) in acetic acid (5 mL), *p*-toluenesulfonamide (1.71 g, 10 mmol) was added at rt. After 5 min, methanesulfonic acid (10 ml) was added dropwise over 2 min and the stirring was continued at 35 °C for 15 min. The mixture was cooled down to 0 °C, then diluted with $CHCl_3$ (100 mL), washed with ice-water (2 x 50 mL) and aq. 5% $NaHCO_3$ (2 x 50 mL). Collected organic phases were dried over anhydrous $Na_2SO_4$ and evaporated to afford product **8a**.

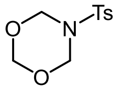

Obtained following as a white solid (2.1 g, 86%).
$^1$H NMR (300 MHz, $CDCl_3$) δ 7.92 – 7.81 (m, 2H), 7.38 – 7.23 (m, 2H), 5.21 (s, 4H), 4.89 (s, 2H), 2.44 (s, 3H). Spectroscopic data was consistent with the values reported in the literature.[5]
$^{13}$C NMR (75 MHz, $CDCl_3$) δ 143.5, 136.7, 129.1, 127.7, 93.6, 77.0, 21.1.
EI-HRMS: *m/z* calculated for $C_{10}H_{13}NO_4S^+$ ($[M]^+$): 243.0560; found: 243.0560.

**3,5-ditosyl-1,3,5-oxadiazinane (8b)**

*(Following a reported procedure*[5]*)*: *p*-Toluenesulfonamide (1.71 g, 10 mmol, 2 equiv.) and *sym*-trioxane (450 mg, 5 mmol) in trifluoroacetic acid (10 ml) were stirred for 2 h at 35 °C. The mixture was then cooled down to 0 °C and $CHCl_3$ (50 ml) was added, followed by 25 mL of ice-water. The organic phase was separated, washed with ice-water (25 mL) and aq. 5% $NaHCO_3$ (2 x 25 mL), dried over anhydrous $Na_2SO_4$ and evaporated. The residue was purified by column chromatography on silica gel (*iso*-hexane/MTBE mixtures from 9:1 to 2:1 *v/v* ) to afford **8b**.

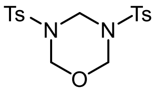

Obtained following as a white solid (169 mg, 9%).
$^1$H NMR (501 MHz, $CDCl_3$) δ 7.71 (d, *J* = 8.3 Hz, 4H), 7.35 (d, *J* = 8.2 Hz, 4H), 4.94 (s, 2H), 4.82 (s, 4H), 2.47 (s, 6H). Spectroscopic data was consistent with the values reported in the literature.[5]
$^{13}$C NMR (126 MHz, $CDCl_3$) δ 144.4, 135.5, 129.9, 127.7, 78.1, 59.9, 21.6.
ESI-HRMS: *m/z* calculated for $C_{17}H_{20}N_2O_5S_2Na^+$ ($[M+Na]^+$): 419.0709; found: 419.0709.

**1,3,5-tritosyl-1,3,5-triazinane (8c)**

*(Following a reported procedure*[5]*)*: a solution of *sym*-trioxane (30 mg, 0.33 mmol) in acetic acid (0.25 mL), was added dropwise to a stirring solution of *p*-toluenesulfonamide (171 mg, 1 mmol) in methanesulfonic acid (1 mL). The mixture was further stirred for 15 min at rt. After cooling down to 0 °C, 10 g of crushed ice were added and the mixture was maintained for 2h in an ice-bath with occasional shaking. The precipitate was filtered off and washed with ice-water, aq. 5% $NaHCO_3$ and water. The dried crude product was recrystallized from methanol to obtain product **8c**.

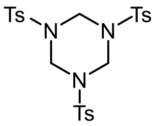

Obtained following as a white solid (163 mg, 90%).

$^{1}H$ NMR (501 MHz, $CDCl_3$) δ 7.61 (d, *J* = 8.3 Hz, 6H), 7.32 – 7.28 (d, *J* = 8.2 Hz, 6H), 4.55 (s, 6H), 2.44 (s, 9H). Spectroscopic data was consistent with the values reported in the literature.[5]

$^{13}C$ NMR (126 MHz, $CDCl_3$) δ 144.6, 134.6, 129.9, 127.6, 60.0, 21.7.

EI-HRMS: *m/z* calculated for $C_{24}H_{27}N_3O_6S_3Na^+$ ($[M+Na]^+$): 572.0954; found: 572.0954

## 3. Reaction Development

At the onset of our studies, we tested the reaction of styrene (**1a**) with *sym*-trioxane (**2**) and *p*-toluenesulfonamide (**3a**) using as catalysts several Brønsted- or Lewis acids (Table S-1).

*Table S-1. Optimization of reaction conditions.*[a]

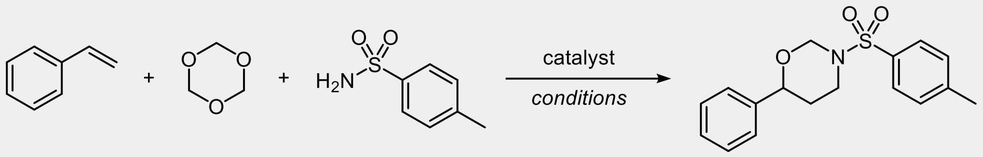


**1a** **2** **3a** **4a**

| Entry | Catalyst (*x* mol%) | Solvent | Temp (°C) | Yield (%) |
|---|---|---|---|---|
| 1 | $Fe(OTf)_3$ (20) | DCE | 25 | 28 |
| 2 | $InCl_3$ (20) | DCE | 25 | 5 |
| 3 | $Zn(OTf)_2$ (20) | DCE | 25 | 0 |
| 4 | $CrCl_3$ (20) | DCE | 25 | 0 |
| 5 | $Sc(OTf)_3$ (20) | DCE | 25 | 0 |
| 6 | $Cu(OAc)_2$ (20) | DCE | 25 | 0 |
| 7 | $Fe(OTf)_3$ (20) | DCE | 60 | 26 |
| 8 | AcOH (20) | $CH_2Cl_2$ | 60 | <5 |
| 9 | *p*-TsOH (20) | $CH_2Cl_2$ | 60 | 38 |
| 10 | $CF_3SO_3H$ (20) | $CH_2Cl_2$ | 60 | 32 |
| 11 | $CF_3COOH$ (10) | $CH_2Cl_2$ | 60 | 0 |
| 12 | (*R*)-TRIP (10) | $CH_2Cl_2$ | 60 | 0 |
| 13 | $Tf_2NH$ (20) | $CH_2Cl_2$ | 60 | 10 |
| 14 | $H_3PO_4$ (20) | $CHCl_3$ | 60 | 0 |
| 15 | $HPF_6$ (10) | DCE | 60 | 58 |
| 16 | $HPF_6$ (20) | DCE | 60 | 68 |
| 17 | $HPF_6$ (20) | DCE | 100 | 58 |
| 18 | $HPF_6$ (20) | DCE | 80 | 56 |
| 19 | $HPF_6$ (20) | DCE | 40 | 49 |
| 20 | $HPF_6$ (20) | DCE | 25 | 8 |
| 21 | $HPF_6$ (20) | $CH_2Cl_2$ | 60 | 68 |
| 22 | ***$HPF_6$ (20)*** | ***$CHCl_3$*** | ***60*** | ***78*** |
| 23 | $HPF_6$ (20) | $CH_3CN$ | 60 | 5 |
| 24 | $HPF_6$ (20) | PhMe | 60 | 20 |
| 25 | - | $CHCl_3$ | 60 | 0 |
| 26[b] | $HPF_6$ (20) | $CHCl_3$ | 60 | 47 |
| 27[c] | $HPF_6$ (20) | $CHCl_3$ | 60 | 41 |
| 28[d] | $HPF_6$ (20) | $CHCl_3$ | 60 | 37 |

[a]**1a** (0.2 mmol), **2** (1.5 equiv.), **3a** (3 equiv.), catalyst, in 2 mL solvent, at the indicated temperature for 24 h; all yields were determined by $^1$H NMR spectroscopy using 1,3,5-trimethoxybenzene as internal standard. [b]**3a** (1.5 equiv.). [c]**2** (1 equiv.), **3a** (1 equiv.). [d]**2** (2 equiv.), **3a** (1 equiv.).

# 4. Acid Catalyzed Oxy-Aminomethylation of Styrenes

*General Procedure A:*

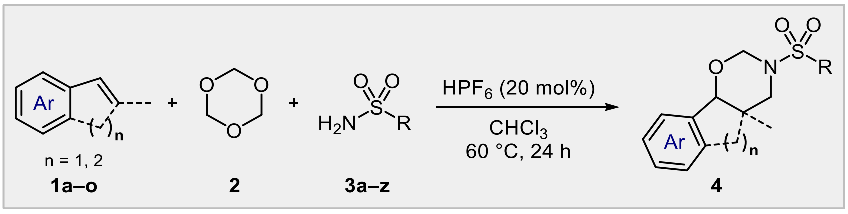


An oven-dried 10 mL glass tube (with screw cap) was charged with olefin **1** (0.2 mmol), *sym*-trioxane **2** (0.3 mmol, 1.5 equiv.), sulfonamide **3** (0.6 mmol, 3 equiv.) and $CHCl_3$ (2 mL). $HPF_6$ (20 mol%, 55% in water) was added in one portion. The tube was closed and the reaction mixture was stirred at 60 °C for 24 h. After the reaction was completed, the mixture was cooled to room temperature and concentrated under reduced pressure to give a crude product. This residue was further purified by column chromatography (silica, *iso*-hexane/ethyl acetate mixtures from 10:1 to 5:1 *v/v*) to give the desired products **4**.

*General Procedure B:*

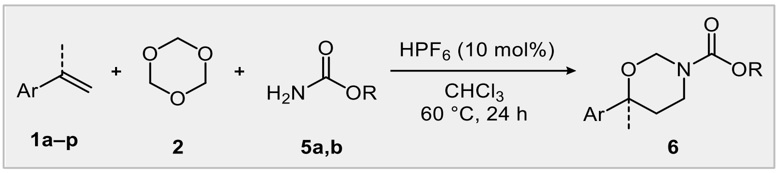


An oven-dried 10 mL glass tube (with screw cap) was charged with olefin **1** (0.5 mmol), *sym*-trioxane **2** (0.75 mmol, 1.5 equiv.), carbamate **5** (0.75 mmol, 1.5 equiv.) and $CHCl_3$ (0.5 mL). $HPF_6$ (10 mol%, 55% in water) was added in one portion. The tube was closed under argon and the reaction mixture was stirred at 60 °C for 24 h. After the reaction was completed, the mixture was cooled to room temperature and concentrated under reduced pressure to give a crude product. This residue was further purified by column chromatography (silica, *iso*-hexane/MTBE mixtures from 5:1 to 2:1 *v/v*) to give the desired products **6**.

**6-phenyl-3-tosyl-1,3-oxazinane** (**4a**)

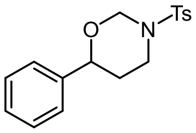

Obtained following the *General Procedure A*, as a white solid (49.5 mg, 78%).
$^1$H NMR (501 MHz, $CDCl_3$) δ 7.88 (d, *J* = 8.3 Hz, 2H), 7.38 (d, *J* = 8.0 Hz, 2H), 7.31 – 7.22 (m, 3H), 7.03 – 6.94 (m, 2H), 5.72 (dd, *J* = 11.2, 2.2 Hz, 1H), 4.66 (d, *J* = 11.2 Hz, 1H), 4.44 (dd, *J* = 10.9, 3.2 Hz, 1H), 4.06 (ddt, *J* = 14.5, 4.6, 2.2 Hz, 1H), 3.40 (ddd, *J* = 14.5, 12.3, 3.9 Hz, 1H), 2.48 (s, 3H), 1.49 – 1.35 (m, 2H).
$^{13}$C NMR (126 MHz, $CDCl_3$) δ 143.8 (C), 141.0 (C), 137.8 (C), 129.9 (CH), 128.5 (CH), 128.1 (CH), 128.0 (CH), 125.8 (CH), 79. (CH), 78.5 ($CH_2$), 44.9 ($CH_2$), 30.9 ($CH_2$), 21.7 ($CH_3$).
ESI-HRMS: *m/z* calculated for $C_{17}H_{19}NO_3SNa^+$ ($[M+Na]^+$): 340.0978; found: 340.0976.

**6-(4-(*tert*-butyl)phenyl)-3-tosyl-1,3-oxazinane** (**4b**)

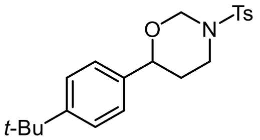

Obtained following the *General Procedure A*, as a white solid (37.3 mg, 50%).
$^1$H NMR (501 MHz, $CDCl_3$) δ 7.87 (d, *J* = 8.3 Hz, 2H), 7.37 (d, *J* = 7.9 Hz, 2H), 7.27 (d, *J* = 8.4 Hz, 2H), 6.90 (d, *J* = 8.3 Hz, 2H), 5.68 (dd, *J* = 11.2, 2.2 Hz,

1H), 4.63 (d, *J* = 11.2 Hz, 1H), 4.41 – 4.37 (m, 1H), 4.04 (ddt, *J* = 14.5, 4.1, 2.3 Hz, 1H), 3.40 – 3.33 (m, 1H), 2.48 (s, 3H), 1.46 – 1.40 (m, 2H), 1.28 (s, 9H).
$^{13}$C NMR (126 MHz, $CDCl_3$) δ 151.2 (C), 143.8 (C), 137.89 (C), 137.86 (C), 129.9 (CH), 128.1 (CH), 125.7 (CH), 125.4 (CH), 79.7 (CH), 78.6 ($CH_2$), 44.9 ($CH_2$), 34.7 (C), 31.4 ($CH_3$), 30.7 ($CH_2$), 21.7 ($CH_3$).
ESI-HRMS: *m/z* calculated for $C_{21}H_{27}NO_3SNa^+$ ($[M+Na]^+$): 396.1604; found: 396.1602.

**6-(*p*-tolyl)-3-tosyl-1,3-oxazinane** (**4c**)

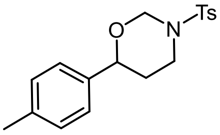


Obtained following the *General Procedure A*, as a white solid (14.7 mg, 20%).
$^{1}$H NMR (501 MHz, $CDCl_3$) δ 7.86 (d, *J* = 8.3 Hz, 2H), 7.36 (d, *J* = 7.9 Hz, 2H), 7.06 (d, *J* = 7.9 Hz, 2H), 5.69 (dd, *J* = 11.2, 2.3 Hz, 1H), 4.63 (d, *J* = 11.2 Hz, 1H), 4.38 (dd, *J* = 9.8, 4.2 Hz, 1H), 4.09 – 3.99 (m, 1H), 3.42 – 3.32 (m, 1H), 2.47 (s, 3H), 2.30 (s, 3H), 1.44 – 1.32 (m, 2H).
$^{13}$C NMR (126 MHz, $CDCl_3$) δ 143.8 (C), 138.1 (C), 137.9 (C), 129.9 (CH), 129.2 (CH), 128.14 (C), 128.09 (CH), 125.8 (CH), 79.6 (CH), 78.5 ($CH_2$), 44.9 ($CH_2$), 30.9 ($CH_2$), 21.7 ($CH_3$), 21.2 ($CH_3$).
ESI-HRMS: *m/z* calculated for $C_{18}H_{21}NO_3SNa^+$ ($[M+Na]^+$): 354.1134; found: 354.1134.

**6-(4-(chloromethyl)phenyl)-3-tosyl-1,3-oxazinane** (**4d**)

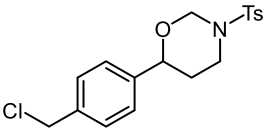


Obtained following the *General Procedure A*, as a white solid (45.2 mg, 62%).
$^{1}$H NMR (501 MHz, $CDCl_3$) δ 7.86 (d, *J* = 8.4 Hz, 2H), 7.36 (d, *J* = 8.0 Hz, 2H), 7.28 (d, *J* = 8.2 Hz, 2H), 6.97 (d, *J* = 8.2 Hz, 2H), 5.70 (dd, *J* = 11.2, 2.2 Hz, 1H), 4.64 (d, *J* = 11.3 Hz, 1H), 4.54 (s, 2H), 4.43 (dd, *J* = 11.2, 2.8 Hz, 1H), 4.04 (ddt, *J* = 14.5, 4.5, 2.1 Hz, 1H), 3.38 (ddd, *J* = 14.5, 12.6, 3.5 Hz, 1H), 2.47 (s, 3H), 1.47 – 1.31 (m, 2H).
$^{13}$C NMR (126 MHz, $CDCl_3$) δ 143.9 (C), 141.3 (C), 137.8 (C), 137.3 (C), 129.9 (CH), 128.8 (CH), 128.1 (CH), 126.2 (CH), 79.3 (CH), 78.5 ($CH_2$), 46.0 ($CH_2$), 44.8 ($CH_2$), 31.0 ($CH_2$), 21.7 ($CH_3$).
ESI-HRMS: *m/z* calculated for $C_{18}H_{20}ClNO_3SNa^+$ ($[M+Na]^+$): 388.0745; found: 388.0743.

**6-(4-fluorophenyl)-3-tosyl-1,3-oxazinane** (**4e**)

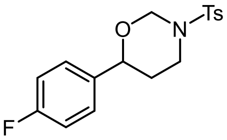


Obtained following the *General Procedure A*, as a white solid (32.1 mg, 48%).
$^{1}$H NMR (501 MHz, $CDCl_3$) δ 7.85 (d, *J* = 8.3 Hz, 2H), 7.36 (d, *J* = 8.1 Hz, 2H), 6.94 (d, *J* = 7.0 Hz, 4H), 5.69 (dd, *J* = 11.2, 2.3 Hz, 1H), 4.63 (d, *J* = 11.3 Hz, 1H), 4.41 (dd, *J* = 11.2, 3.0 Hz, 1H), 4.04 (ddt, *J* = 14.5, 4.5, 2.1 Hz, 1H), 3.37 (ddd, *J* = 14.5, 12.5, 3.6 Hz, 1H), 2.46 (s, 3H), 1.47 – 1.30 (m, 2H).
$^{19}$F NMR (471 MHz, $CDCl_3$) δ -114.16 (s, 1F).
$^{13}$C NMR (126 MHz, $CDCl_3$) δ 162.5 (d, *J* = 246.3 Hz, C), 143.9 (C), 137.8 (C), 136.9 (d, *J* = 3.0 Hz, C), 129.9 (CH), 128.1 (CH), 127.5 (d, *J* = 8.0 Hz, CH), 115.4 (d, *J* = 21.7 Hz, CH), 79.0 (CH), 78.5 ($CH_2$), 44.8 ($CH_2$), 31.1 ($CH_2$), 21.7 ($CH_3$).
ESI-HRMS: *m/z* calculated for $C_{17}H_{18}FNO_3SNa^+$ ($[M+Na]^+$): 358.0884; found: 358.0880.

**6-(4-chlorophenyl)-3-tosyl-1,3-oxazinane** (**4f**)

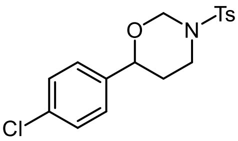


Obtained following the *General Procedure A*, as a white solid (43.1 mg, 68%).
$^{1}$H NMR (501 MHz, $CDCl_3$) δ 7.85 (d, *J* = 8.3 Hz, 2H), 7.36 (d, *J* = 8.0 Hz, 2H), 7.22 (d, *J* = 8.5 Hz, 2H), 6.90 (d, *J* = 8.4 Hz, 2H), 5.69 (dd, *J* = 11.2, 2.2 Hz, 1H), 4.63 (d, *J* = 11.3 Hz, 1H), 4.41 (dd, *J* = 11.3, 2.8 Hz, 1H), 4.04 (ddt, *J* = 14.5, 4.5, 2.1 Hz, 1H), 3.37 (ddd, *J* = 14.5, 12.7, 3.4 Hz, 1H), 2.46 (s, 3H), 1.43 (dq, *J* = 13.5, 2.7 Hz, 1H), 1.33 (dddd, *J* = 13.7, 12.8, 11.3, 4.9 Hz, 1H).
$^{13}$C NMR (126 MHz, $CDCl_3$) δ 143.9 (C), 139.6 (C), 137.8 (C), 133.8 (C), 129.9 (CH), 128.7 (CH), 128.1 (CH), 127.2 (CH), 78.9 (CH), 78.4 ($CH_2$), 44.8 ($CH_2$), 31.0 ($CH_2$), 21.7 ($CH_3$).

ESI-HRMS: *m/z* calculated for $C_{17}H_{18}ClNO_3SNa^+$ ($[M+Na]^+$): 374.0588; found: 374.0589.

**6-(4-bromophenyl)-3-tosyl-1,3-oxazinane** (**4g**)

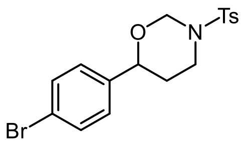


Obtained following the *General Procedure A*, as a white solid (32.1 mg, 40%).
$^1H$ NMR (501 MHz, $CDCl_3$) δ 7.85 (d, *J* = 8.4 Hz, 2H), 7.37 (d, *J* = 8.5 Hz, 2H), 7.35 (d, *J* = 8.0 Hz, 2H), 6.84 (d, *J* = 8.4 Hz, 2H), 5.69 (dd, *J* = 11.2, 2.2 Hz, 1H), 4.62 (d, *J* = 11.2 Hz, 1H), 4.39 (dd, *J* = 11.4, 2.7 Hz, 1H), 4.03 (ddt, *J* = 14.5, 4.5, 2.1 Hz, 1H), 3.37 (ddd, *J* = 14.5, 12.7, 3.4 Hz, 1H), 2.46 (s, 3H), 1.42 (dq, *J* = 13.8, 2.8 Hz, 1H), 1.32 (dddd, *J* = 13.7, 12.8, 11.4, 4.9 Hz, 1H).
$^{13}C$ NMR (126 MHz, $CDCl_3$) δ 143.9 (C), 140.1 (C), 137.8 (C), 131.6 (CH), 129.9 (CH), 128.1 (CH), 127.5 (CH), 121.9 (C), 78.9 (CH), 78.4 ($CH_2$), 44.7 ($CH_2$), 31.0 ($CH_2$), 21.7 ($CH_3$).
ESI-HRMS: *m/z* calculated for $C_{17}H_{18}BrNO_3SNa^+$ ($[M+Na]^+$): 418.0083; found: 418.0084.

**6-(*m*-tolyl)-3-tosyl-1,3-oxazinane** (**4h**)

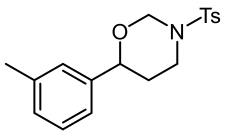


Obtained following the *General Procedure A*, as a white solid (40.0 mg, 60%).
$^1H$ NMR (501 MHz, $CDCl_3$) δ 7.88 (d, *J* = 8.3 Hz, 2H), 7.37 (d, *J* = 8.1 Hz, 2H), 7.14 (t, *J* = 7.6 Hz, 1H), 7.05 (d, *J* = 7.7 Hz, 1H), 6.78 (d, *J* = 7.6 Hz, 1H), 6.73 (s, 1H), 5.70 (dd, *J* = 11.3, 2.2 Hz, 1H), 4.65 (d, *J* = 11.3 Hz, 1H), 4.39 (dd, *J* = 10.4, 3.6 Hz, 1H), 4.05 (ddt, *J* = 14.5, 4.7, 2.3 Hz, 1H), 3.44 – 3.34 (m, 1H), 2.47 (s, 3H), 2.29 (s, 3H), 1.44 – 1.30 (m, 2H).
$^{13}C$ NMR (126 MHz, $CDCl_3$) δ 143.8 (C), 140.9 (C), 138.2 (C), 138.0 (C), 129.9 (CH), 128.9 (CH), 128.4 (CH), 128.1 (CH), 126.5 (CH), 123.0 (CH), 79.8 (CH), 78.5 ($CH_2$), 44.9 ($CH_2$), 30.9 ($CH_2$), 21.7 ($CH_3$), 21.5 ($CH_3$).
ESI-HRMS: *m/z* calculated for $C_{18}H_{21}NO_3SNa^+$ ($[M+Na]^+$): 354.1134; found: 354.1133.

**6-(*o*-tolyl)-3-tosyl-1,3-oxazinane** (**4i**)

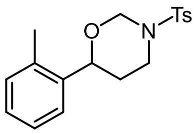


Obtained following the *General Procedure A*, as a white solid (42.0 mg, 63%).
$^1H$ NMR (501 MHz, $CDCl_3$) δ 7.87 (d, *J* = 8.3 Hz, 2H), 7.37 (d, *J* = 8.2 Hz, 2H), 7.13 (td, *J* = 7.3, 1.6 Hz, 1H), 7.12 – 7.04 (m, 2H), 6.80 (dd, *J* = 7.7, 1.6 Hz, 1H), 5.72 (dd, *J* = 11.3, 2.2 Hz, 1H), 4.67 (d, *J* = 11.2 Hz, 1H), 4.62 (dd, *J* = 11.1, 2.7 Hz, 1H), 4.07 (ddt, *J* = 14.4, 4.5, 2.1 Hz, 1H), 3.39 (ddd, *J* = 14.4, 12.6, 3.5 Hz, 1H), 2.46 (s, 3H), 2.22 (s, 3H), 1.46 – 1.38 (m, 1H), 1.34 (dddd, *J* = 13.9, 12.7, 11.1, 4.8 Hz, 1H).
$^{13}C$ NMR (126 MHz, $CDCl_3$) δ 143.9 (C), 139.1 (C), 137.8 (C), 134.0 (C), 130.4 (CH), 129.9 (CH), 128.0 (CH), 127.8 (CH), 126.3 (CH), 125.6 (CH), 78.7 ($CH_2$), 76.5 (CH), 45.0 ($CH_2$), 29.6 ($CH_2$), 21.7 ($CH_3$), 19.0 ($CH_3$).
ESI-HRMS: *m/z* calculated for $C_{18}H_{21}NO_3SNa^+$ ($[M+Na]^+$): 354.1134; found: 354.1132.

**6-mesityl-3-tosyl-1,3-oxazinane** (**4j**)

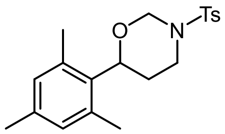


Obtained following the *General Procedure A*, as a white solid (25.1 mg, 35%).
$^1H$ NMR (501 MHz, $CDCl_3$) δ 7.80 (d, *J* = 8.4 Hz, 2H), 7.33 (d, *J* = 8.0 Hz, 2H), 6.74 (s, 2H), 5.62 (dd, *J* = 10.9, 2.2 Hz, 1H), 4.77 (dd, *J* = 11.9, 2.4 Hz, 1H), 4.54 (d, *J* = 11.0 Hz, 1H), 4.13 (ddt, *J* = 14.2, 4.4, 1.9 Hz, 1H), 3.28 (ddd, *J* = 14.2, 12.6, 3.3 Hz, 1H), 2.45 (s, 3H), 2.19 (s, 3H), 2.06 (s, 6H), 2.01 – 1.90 (m, 1H), 1.40 – 1.32 (m, 1H).
$^{13}C$ NMR (126 MHz, $CDCl_3$) δ 143.7 (C), 137.5 (C), 137.2 (C), 135.9 (C), 133.1 (C), 130.12 (CH), 130.06 (CH), 127.5 (CH), 78.7 ($CH_2$), 78.2 (CH), 45.3 ($CH_2$), 27.8 ($CH_2$), 21.6 ($CH_3$), 20.8 ($CH_3$), 20.6 ($CH_3$).
ESI-HRMS: *m/z* calculated for $C_{20}H_{26}NO_3S^+$ ($[M+H]^+$): 360.1628; found: 360.1627.

**6-(naphthalen-2-yl)-3-tosyl-1,3-oxazinane** (**4k**)

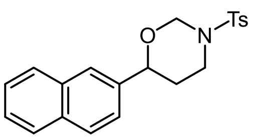

Obtained following the *General Procedure A*, as a white solid (28.1 mg, 38%).
$^1$H NMR (501 MHz, CDCl$_3$) δ 7.91 (d, *J* = 8.3 Hz, 2H), 7.81 – 7.77 (m, 1H), 7.76 – 7.71 (m, 2H), 7.50 – 7.43 (m, 3H), 7.40 (d, *J* = 8.1 Hz, 2H), 7.04 (dd, *J* = 8.5, 1.7 Hz, 1H), 5.76 (dd, *J* = 11.3, 2.2 Hz, 1H), 4.72 (d, *J* = 11.3 Hz, 1H), 4.60 (dd, *J* = 11.0, 3.0 Hz, 1H), 4.09 (ddt, *J* = 14.5, 4.4, 2.2 Hz, 1H), 3.44 (ddd, *J* = 14.5, 12.5, 3.7 Hz, 1H), 2.48 (s, 3H), 1.55 – 1.40 (m, 2H).
$^{13}$C NMR (126 MHz, CDCl$_3$) δ 143.9, 138.5, 138.0, 133.3, 133.2, 130.0, 128.3, 128.2, 128.0, 127.8, 126.4, 126.2, 124.7, 123.8, 79.7, 78.6, 44.9, 31.0, 21.8.
ESI-HRMS: *m/z* calculated for $C_{21}H_{21}NO_3SNa^+$ ([M+Na]$^+$): 390.1134; found: 390.1138.

**(4a*R**,9b*S**)-3-tosyl-2,3,4,4a,5,9b-hexahydroindeno[2,1-*e*][1,3]oxazine** (**4l**)

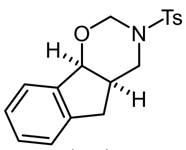

*(rac)*

Obtained following the *General Procedure A*, as a white solid (27.0 mg, 41%).
$^1$H NMR (600 MHz, CDCl$_3$) δ 7.77 (d, *J* = 8.3 Hz, 2H), 7.35 (d, *J* = 7.2 Hz, 1H), 7.31 (d, *J* = 7.9 Hz, 2H), 7.29 – 7.23 (m, 2H), 7.22 (d, *J* = 7.3 Hz, 1H), 4.96 (d, *J* = 5.5 Hz, 1H), 4.91 (d, *J* = 10.2 Hz, 1H), 4.84 (dd, *J* = 10.2, 1.0 Hz, 1H), 3.55 (ddd, *J* = 13.8, 5.8, 1.0 Hz, 1H), 3.32 (dd, *J* = 13.8, 7.0 Hz, 1H), 2.79 (dd, *J* = 15.7, 7.0 Hz, 1H), 2.60 (dd, *J* = 15.7, 5.3 Hz, 1H), 2.43 (s, 3H), 2.37 (qd, *J* = 5.5, 1.5 Hz, 1H).
$^{13}$C NMR (151 MHz, CDCl$_3$) δ 143.7 (C), 142.7 (C), 140.0 (C), 137.0 (C), 129.9 (CH), 129.0 (CH), 127.6 (CH), 127.2 (CH), 125.7 (CH), 124.8 (CH), 80.2 (CH), 74.2 (CH$_2$), 45.4 (CH$_2$), 36.0 (CH), 33.6 (CH$_2$), 21.7 (CH$_3$).
ESI-HRMS: *m/z* calculated for $C_{18}H_{20}NO_3S^+$ ([M+H]$^+$): 330.1158; found: 330.1159.

**(4a*S**,10b*S**)-3-tosyl-3,4,4a,5,6,10b-hexahydro-2*H*-naphtho[2,1-e][1,3]oxazine** (*trans*-**4m**)

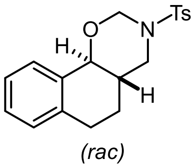

*(rac)*

Obtained following the *General Procedure A*, as a white solid (10.4 mg, 16%).
$^1$H NMR (600 MHz, CDCl$_3$) δ 7.75 (d, *J* = 8.3 Hz, 2H), 7.44 – 7.39 (m, 1H), 7.25 (d, *J* = 8.2 Hz, 2H), 7.21 – 7.13 (m, 2H), 7.06 – 7.01 (m, 1H), 5.74 (dd, *J* = 10.4, 2.0 Hz, 1H), 4.63 (d, *J* = 10.4 Hz, 1H), 4.19 (d, *J* = 10.2 Hz, 1H), 3.92 (ddd, *J* = 13.4, 4.4, 2.0 Hz, 1H), 2.88 (dd, *J* = 13.4, 11.4 Hz, 1H), 2.80 – 2.75 (m, 2H), 2.38 (s, 3H), 1.68 (ddt, *J* = 13.2, 5.0, 3.0 Hz, 1H), 1.55 (tdt, *J* = 7.2, 4.3, 2.2 Hz, 1H), 1.45 – 1.35 (m, 1H).
$^{13}$C NMR (151 MHz, CDCl$_3$) δ 143.7 (C), 137.0 (C), 135.7 (C), 135.4 (C), 129.9 (CH), 128.7 (CH), 127.6 (CH), 127.5 (CH), 126.1 (CH), 125.0 (CH), 80.7 (CH), 78.6 (CH$_2$), 49.9 (CH$_2$), 35.9 (CH), 28.0 (CH$_2$), 24.4 (CH$_2$), 21.7 (CH$_3$).
ESI-HRMS: *m/z* calculated for $C_{19}H_{21}NO_3SNa^+$ ([M+Na]$^+$): 366.1134; found: 366.1138.

**(4a*R**,10b*S**)-3-tosyl-3,4,4a,5,6,10b-hexahydro-2*H*-naphtho[2,1-e][1,3]oxazine** (*cis*-**4m**)

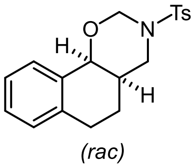

*(rac)*

Obtained following the *General Procedure A*, as a white solid (10.4 mg, 16%).
$^1$H NMR (600 MHz, CDCl$_3$) δ 7.71 (d, *J* = 8.3 Hz, 2H), 7.31 (d, *J* = 7.9 Hz, 2H), 7.28 (dd, *J* = 7.5, 1.7 Hz, 1H), 7.23 (td, *J* = 7.4, 1.7 Hz, 1H), 7.21 – 7.17 (m, 1H), 7.10 (d, *J* = 7.3 Hz, 1H), 5.25 (dd, *J* = 9.6, 1.8 Hz, 1H), 4.57 (d, *J* = 9.7 Hz, 1H), 4.46 (d, *J* = 3.1 Hz, 1H), 3.73 (ddd, *J* = 13.2, 3.5, 1.8 Hz, 1H), 3.34 (dd, *J* = 13.1, 3.8 Hz, 1H), 2.81 (ddd, *J* = 17.1, 5.5, 3.7 Hz, 1H), 2.73 (ddd, *J* = 17.0, 10.9, 5.8 Hz, 1H), 2.43 (s, 3H), 1.96 (dtd, *J* = 12.9, 11.1, 5.6 Hz, 1H), 1.90 – 1.85 (m, 1H), 1.60 – 1.52 (m, 1H).
$^{13}$C NMR (151 MHz, CDCl$_3$) δ 143.7 (C), 137.3 (C), 136.6 (C), 134.1 (C), 130.3 (CH), 129.9 (CH), 129.1 (CH), 128.7 (CH), 127.4 (CH), 126.4 (CH), 77.3 (CH), 75.5 (CH$_2$), 48.7 (CH$_2$), 33.4 (CH), 28.5 (CH$_2$), 22.5 (CH$_2$), 21.7 (CH$_3$).
ESI-HRMS: *m/z* calculated for $C_{19}H_{21}NO_3SNa^+$ ([M+Na]$^+$): 366.1134; found: 366.1138.

**(5*S**,6*S**)-5-methyl-6-phenyl-3-tosyl-1,3-oxazinane** (*trans*-**4n**)

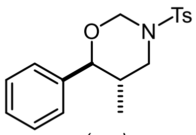

(rac)

Obtained following the *General Procedure A*, as a white solid (20.7 mg, 31%).
$^{1}H$ NMR (600 MHz, $CDCl_3$) δ 7.93 – 7.86 (m, 2H), 7.45 – 7.38 (m, 2H), 7.30 – 7.21 (m, 3H), 6.92 – 6.84 (m, 2H), 5.69 (dd, *J* = 11.2, 2.2 Hz, 1H), 4.60 (d, *J* = 11.2 Hz, 1H), 4.01 (ddd, *J* = 14.5, 4.8, 2.2 Hz, 1H), 3.92 (d, *J* = 10.1 Hz, 1H), 2.96 (dd, *J* = 14.5, 11.8 Hz, 1H), 2.52 (s, 3H), 1.49 (ddqd, *J* = 11.4, 10.0, 6.7, 4.7 Hz, 1H), 0.51 (d, *J* = 6.7 Hz, 3H).
$^{13}C$ NMR (151 MHz, $CDCl_3$) δ 143.8 (C), 139.1 (C), 137.9 (C), 130.0 (CH), 128.5 (CH), 128.4 (CH), 128.1 (CH), 127.2 (CH), 86.7 (CH), 78.3 ($CH_2$), 51.5 ($CH_2$), 33.3 (CH), 21.7 ($CH_3$), 14.3 ($CH_3$).
ESI-HRMS: *m/z* calculated for $C_{18}H_{21}NO_3SNa^+$ ($[M+Na]^+$): 354.1134; found: 354.1136.

**(5*R**,6*S**)-5-methyl-6-phenyl-3-tosyl-1,3-oxazinane** (*cis*-**4n**)

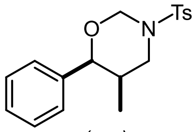

(rac)

Obtained following the *General Procedure A*, as a white solid (10.4 mg, 16%).
$^{1}H$ NMR (600 MHz, $CDCl_3$) δ 7.72 – 7.68 (m, 2H), 7.35 – 7.30 (m, 4H), 7.24 (ddt, *J* = 8.5, 6.7, 1.4 Hz, 1H), 7.20 (ddt, *J* = 6.9, 1.3, 0.8 Hz, 2H), 5.54 (dd, *J* = 9.1, 2.1 Hz, 1H), 4.58 (d, *J* = 2.5 Hz, 1H), 4.34 (d, *J* = 9.1 Hz, 1H), 3.79 (dt, *J* = 12.2, 2.2 Hz, 1H), 3.16 (dd, *J* = 12.2, 3.1 Hz, 1H), 2.44 (s, 3H), 1.99 (dddd, *J* = 9.7, 7.0, 4.2, 2.0 Hz, 1H), 0.73 (d, *J* = 6.9 Hz, 1H).
$^{13}C$ NMR (151 MHz, $CDCl_3$) δ 143.7 (C), 139.8 (C), 136.2 (C), 129.9 (CH), 128.3 (CH), 127.3 (CH), 127.3 (CH), 125.3 (CH), 81.5 (CH), 79.2 ($CH_2$), 51.2 ($CH_2$), 33.8 (CH), 21.7 ($CH_3$), 11.4 ($CH_3$).
ESI-HRMS: *m/z* calculated for $C_{18}H_{21}NO_3SNa^+$ ($[M+Na]^+$): 354.1134; found: 354.1136.

**6-(4-allylphenyl)-3-tosyl-1,3-oxazinane** (**4o**)

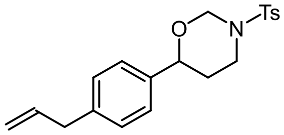

Obtained following the *General Procedure A*, as a white solid (37.8 mg, 53%).
$^{1}H$ NMR (501 MHz, $CDCl_3$) δ 7.86 (d, *J* = 8.1 Hz, 2H), 7.36 (d, *J* = 8.0 Hz, 2H), 7.08 (d, *J* = 8.0 Hz, 2H), 6.89 (d, *J* = 7.9 Hz, 2H), 5.92 (ddt, *J* = 17.5, 9.5, 6.7 Hz, 1H), 5.69 (dd, *J* = 11.2, 2.2 Hz, 1H), 5.10 – 5.01 (m, 2H), 4.64 (d, *J* = 11.2 Hz, 1H), 4.40 (dd, *J* = 10.1, 4.0 Hz, 1H), 4.04 (ddt, *J* = 14.5, 4.9, 2.5 Hz, 1H), 3.41 – 3.36 (m, 1H), 3.34 (d, *J* = 6.8 Hz, 2H), 2.47 (s, 3H), 1.45 – 1.33 (m, 2H).
$^{13}C$ NMR (126 MHz, $CDCl_3$) δ 143.8, 140.0, 138.8, 137.8, 137.3, 129.9, 128.7, 128.1, 126.0, 116.0, 79.6, 78.5, 44.9, 40.0, 30.9, 21.7.
ESI-HRMS: *m/z* calculated for $C_{20}H_{23}NO_3S^+$ ($[M]^+$): 357.1393; found: 357.1399.

**6-phenyl-3-(phenylsulfonyl)-1,3-oxazinane** (**4p**)

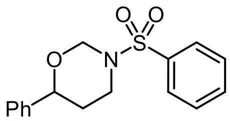

Obtained following the *General Procedure A*, as a white solid (41.8 mg, 69%).
$^{1}H$ NMR (501 MHz, $CDCl_3$) δ 8.03 – 7.97 (m, 2H), 7.67 – 7.60 (m, 1H), 7.61 – 7.54 (m, 2H), 7.28 – 7.21 (m, 3H), 6.98 – 6.92 (m, 2H), 5.73 (dd, *J* = 11.3, 2.2 Hz, 1H), 4.67 (d, *J* = 11.3 Hz, 1H), 4.43 (dd, *J* = 11.3, 2.7 Hz, 1H), 4.06 (ddt, *J* = 14.6, 4.6, 2.1 Hz, 1H), 3.41 (ddd, *J* = 14.5, 12.7, 3.3 Hz, 1H), 1.45 (dq, *J* = 13.6, 2.8 Hz, 1H), 1.35 (dddd, *J* = 13.8, 12.9, 11.3, 4.9 Hz, 1H).
$^{13}C$ NMR (126 MHz, $CDCl_3$) δ 140.9 (C), 140.8 (C), 133.0 (CH), 129.3 (CH), 128.5 (CH), 128.1 (CH), 128.0 (CH), 125.8 (CH), 79.8 (CH), 78.5 ($CH_2$), 44.9 ($CH_2$), 30.9 ($CH_2$).
ESI-HRMS: *m/z* calculated for $C_{16}H_{17}NO_3SNa^+$ ($[M+Na]^+$): 326.0821; found: 326.0820.

**6-phenyl-3-(*o*-tolylsulfonyl)-1,3-oxazinane** (**4q**)

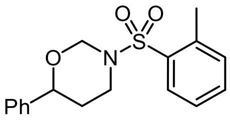

Obtained following the *General Procedure A*, as a white solid (25.4 mg, 40%).
$^{1}H$ NMR (501 MHz, $CDCl_3$) δ 8.06 (dd, *J* = 7.9, 1.4 Hz, 1H), 7.48 (td, *J* = 7.5, 1.4 Hz, 1H), 7.37 – 7.24 (m, 5H), 7.16 – 7.13 (m, 2H), 5.66 (dd, *J* = 11.1, 2.2 Hz, 1H),

4.70 (d, *J* = 11.1 Hz, 1H), 4.51 (dd, *J* = 10.1, 3.9 Hz, 1H), 3.92 (ddt, *J* = 14.2, 4.5, 2.4 Hz, 1H), 3.41 – 3.34 (m, 1H), 2.72 (s, 3H), 1.66 – 1.57 (m, 2H).
$^{13}$C NMR (126 MHz, $CDCl_3$) δ 141.0 (C), 138.2 (C), 138.1 (C), 133.10 (CH), 133.08 (CH), 130.3 (CH), 128.6 (CH), 128.1 (CH), 126.4 (CH), 125.9 (CH), 79.8 (CH), 77.7 ($CH_2$), 44.7 ($CH_2$), 31.9 ($CH_2$), 20.7 ($CH_3$).
ESI-HRMS: *m/z* calculated for $C_{17}H_{19}NO_3SNa^+$ ($[M+Na]^+$): 340.0978; found: 340.0974.

**3-((4-(*tert*-butyl)phenyl)sulfonyl)-6-phenyl-1,3-oxazinane (4r)**

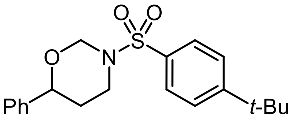


Obtained following the *General Procedure A*, as a white solid (50.2 mg, 70%).
$^{1}$H NMR (501 MHz, $CDCl_3$) δ 7.91 (d, *J* = 8.6 Hz, 2H), 7.58 (d, *J* = 8.5 Hz, 2H), 7.25 – 7.19 (m, 3H), 6.92 – 6.87 (m, 2H), 1.43 – 1.23 (m, 2H), 5.71 (dd, *J* = 11.4, 2.2 Hz, 1H), 4.67 (d, *J* = 11.4 Hz, 1H), 4.41 (dd, *J* = 11.3, 2.8 Hz, 1H), 4.09 (ddt, *J* = 14.6, 4.5, 2.1 Hz, 1H), 3.42 (ddd, *J* = 14.6, 12.7, 3.5 Hz, 1H), 1.43 – 1.23 (m, 2H), 1.38 (s, 9H).
$^{13}$C NMR (126 MHz, $CDCl_3$) δ 156.9 (C), 141.0 (C), 137.9 (C), 128.5 (CH), 128.2 (CH), 127.9 (CH), 126.3 (CH), 125.9 (CH), 79.9 (CH), 78.6 ($CH_2$), 45.0 ($CH_2$), 35.4 (C), 31.3 ($CH_3$), 30.8 ($CH_2$).
ESI-HRMS: *m/z* calculated for $C_{20}H_{25}NO_3SNa^+$ ($[M+Na]^+$): 382.1447; found: 382.1450.

**3-((4-methoxyphenyl)sulfonyl)-6-phenyl-1,3-oxazinane (4s)**

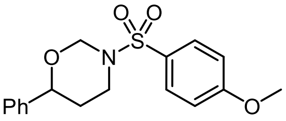


Obtained following the *General Procedure A*, as a white solid (24.6 mg, 37%).
$^{1}$H NMR (501 MHz, $CDCl_3$) δ 7.92 (d, *J* = 8.9 Hz, 2H), 7.30 – 7.23 (m, 3H), 7.04 (d, *J* = 8.9 Hz, 2H), 7.01 (dd, *J* = 7.6, 2.0 Hz, 2H), 5.71 (dd, *J* = 11.2, 2.2 Hz, 1H), 4.66 (d, *J* = 11.3 Hz, 1H), 4.45 (dd, *J* = 11.1, 2.9 Hz, 1H), 4.03 (ddt, *J* = 14.5, 4.5, 2.2 Hz, 1H), 3.90 (s, 3H), 3.40 (ddd, *J* = 14.5, 12.5, 3.6 Hz, 1H), 1.50 – 1.33 (m, 2H).
$^{13}$C NMR (126 MHz, $CDCl_3$) δ 163.3 (C), 141.0 (C), 132.4 (C), 130.2 (CH), 128.5 (CH), 128.1 (CH), 125.8 (CH), 114.5 (CH), 79.7 (CH), 78.5 ($CH_2$), 55.8 ($CH_3$), 44.8 ($CH_2$), 30.9 ($CH_2$).
ESI-HRMS: *m/z* calculated for $C_{17}H_{19}NO_4SNa^+$ ($[M+Na]^+$): 356.0927; found: 356.0927.

**3-((4-fluorophenyl)sulfonyl)-6-phenyl-1,3-oxazinane (4t)**

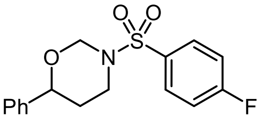


Obtained following the *General Procedure A*, as a white solid (10.3 mg, 16%).
$^{1}$H NMR (501 MHz, $CDCl_3$) δ 8.07 – 8.01 (m, 2H), 7.35 – 7.25 (m, 5H), 7.03 (dd, *J* = 7.8, 1.8 Hz, 2H), 5.74 (dd, *J* = 11.3, 2.2 Hz, 1H), 4.72 (d, *J* = 11.3 Hz, 1H), 4.50 (dd, *J* = 11.4, 2.6 Hz, 1H), 4.08 (ddt, *J* = 14.6, 4.6, 2.1 Hz, 1H), 3.46 (ddd, *J* = 14.6, 12.8, 3.3 Hz, 1H), 1.53 (dq, *J* = 13.8, 2.7 Hz, 1H), 1.45 – 1.33 (m, 1H).
$^{19}$F NMR (471 MHz, $CDCl_3$) δ -104.96 (s, 1F).
$^{13}$C NMR (126 MHz, $CDCl_3$) δ 165.4 (d, *J* = 255.3 Hz, C), 140.7 (C), 136.9 (d, *J* = 3.2 Hz, C), 130.7 (d, *J* = 9.2 Hz, CH), 128.6 (CH), 128.2 (CH), 125.6 (CH), 116.5 (d, *J* = 22.5 Hz, CH), 79.7 (CH), 78.5 ($CH_2$), 44.9 ($CH_2$), 30.9 ($CH_2$).
ESI-HRMS: *m/z* calculated for $C_{16}H_{16}FNO_3SNa^+$ ($[M+Na]^+$): 344.0727; found: 344.0726.

**3-((4-chlorophenyl)sulfonyl)-6-phenyl-1,3-oxazinane (4u)**

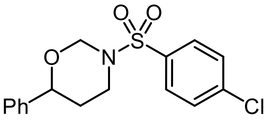


Obtained following the *General Procedure A*, as a white solid (45.2 mg, 67%).
$^{1}$H NMR (501 MHz, $CDCl_3$) δ 7.93 (d, *J* = 8.6 Hz, 2H), 7.55 (d, *J* = 8.6 Hz, 2H), 7.32 – 7.23 (m, 3H), 6.98 – 6.94 (m, 2H), 5.69 (dd, *J* = 11.3, 2.2 Hz, 1H), 4.68 (d, *J* = 11.4 Hz, 1H), 4.46 (dd, *J* = 11.3, 2.7 Hz, 1H), 4.05 (ddt, *J* = 14.6, 4.5, 2.1 Hz, 1H), 3.43 (ddd, *J* = 14.6, 12.8, 3.5 Hz, 1H), 1.49 (dq, *J* = 13.8, 3.0 Hz, 1H), 1.39 (dddd, *J* = 13.8, 12.8, 11.3, 5.0 Hz, 1H).

$^{13}C$ NMR (126 MHz, $CDCl_3$) δ 140.7 (C), 139.6 (C), 139.5 (C), 129.6 (CH), 129.5 (CH), 128.7 (CH), 128.3 (CH), 125.7 (CH), 79.7 (CH), 78.5 ($CH_2$), 45.0 ($CH_2$), 31.0 ($CH_2$).
ESI-HRMS: *m/z* calculated for $C_{16}H_{16}ClNO_3SNa^+$ ([M+Na]$^+$): 360.0432; found: 360.0431.

**3-((4-bromophenyl)sulfonyl)-6-phenyl-1,3-oxazinane** (**4v**)

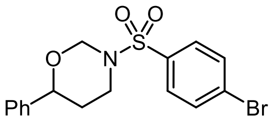

Obtained following the *General Procedure A*, as a white solid (57.9 mg, 76%).
$^1H$ NMR (501 MHz, $CDCl_3$) δ 7.85 (d, *J* = 8.6 Hz, 2H), 7.72 (d, *J* = 8.6 Hz, 2H), 7.32 – 7.23 (m, 3H), 6.97 – 6.93 (m, 2H), 5.69 (dd, *J* = 11.3, 2.2 Hz, 1H), 4.68 (d, *J* = 11.4 Hz, 1H), 4.46 (dd, *J* = 11.3, 2.8 Hz, 1H), 4.06 (ddt, *J* = 14.6, 4.5, 2.1 Hz, 1H), 3.43 (ddd, *J* = 14.7, 12.8, 3.5 Hz, 1H), 1.51 – 1.46 (m, 1H), 1.40 (dddd, *J* = 13.7, 12.7, 11.2, 4.9 Hz, 1H).
$^{13}C$ NMR (126 MHz, $CDCl_3$) δ 140.7 (C), 140.0 (C), 132.6 (CH), 129.6 (CH), 128.7 (CH), 128.3 (CH), 128.1 (C), 125.7 (CH), 79.7 (CH), 78.5 ($CH_2$), 45.0 ($CH_2$), 30.9 ($CH_2$).
ESI-HRMS: *m/z* calculated for $C_{16}H_{16}BrNO_3SNa^+$ ([M+Na]$^+$): 403.9927; found: 403.9926.

**3-((4-nitrophenyl)sulfonyl)-6-phenyl-1,3-oxazinane** (**4w**)

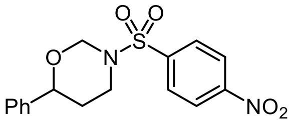

Obtained following the *General Procedure A*, as a white solid (25.0 mg, 36%).
$^1H$ NMR (501 MHz, $CDCl_3$) δ 8.40 (d, *J* = 8.8 Hz, 2H), 8.18 (d, *J* = 8.8 Hz, 2H), 7.28 – 7.23 (m, 3H), 6.97 – 6.93 (m, 2H), 5.73 (dd, *J* = 11.3, 2.2 Hz, 1H), 4.74 (d, *J* = 11.4 Hz, 1H), 4.50 (dd, *J* = 11.5, 2.5 Hz, 1H), 4.08 (ddt, *J* = 14.5, 4.6, 2.1 Hz, 1H), 3.49 (ddd, *J* = 14.7, 12.9, 3.3 Hz, 1H), 1.55 (dq, *J* = 13.8, 2.7 Hz, 1H), 1.33 (dddd, *J* = 13.9, 12.9, 11.4, 4.9 Hz, 1H).
$^{13}C$ NMR (126 MHz, $CDCl_3$) δ 150.3 (C), 146.8 (C), 140.4 (C), 129.3 (CH), 128.7 (CH), 128.4 (CH), 125.4 (CH), 124.5 (CH), 79.6 (CH), 78.5 ($CH_2$), 45.0 ($CH_2$), 31.1 ($CH_2$).
ESI-HRMS: *m/z* calculated for $C_{16}H_{16}N_2O_5SNa^+$ ([M+Na]$^+$): 371.0672; found: 371.0673.

**3-((3-chlorophenyl)sulfonyl)-6-phenyl-1,3-oxazinane** (**4x**)

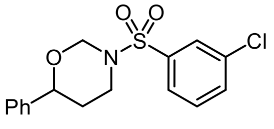

Obtained following the *General Procedure A*, as a white solid (30.0 mg, 44%).
$^1H$ NMR (501 MHz, $CDCl_3$) δ 8.01 (t, *J* = 1.9 Hz, 1H), 7.88 (dt, *J* = 7.8, 1.5 Hz, 1H), 7.59 (ddd, *J* = 8.0, 2.2, 1.1 Hz, 1H), 7.50 (t, *J* = 7.9 Hz, 1H), 7.31 – 7.23 (m, 3H), 7.05 – 7.00 (m, 2H), 5.73 (dd, *J* = 11.3, 2.2 Hz, 1H), 4.70 (d, *J* = 11.3 Hz, 1H), 4.48 (dd, *J* = 11.5, 2.6 Hz, 1H), 4.03 (ddt, *J* = 14.7, 4.6, 2.1 Hz, 1H), 3.45 (ddd, *J* = 14.6, 12.9, 3.3 Hz, 1H), 1.49 (dq, *J* = 13.8, 2.7 Hz, 1H), 1.37 – 1.25 (m, 1H).
$^{13}C$ NMR (126 MHz, $CDCl_3$) δ 142.6 (C), 140.8 (C), 135.6 (C), 133.1 (CH), 130.5 (CH), 128.6 (CH), 128.2 (CH), 128.1 (CH), 126.2 (CH), 125.6 (CH), 79.7 (CH), 78.5 ($CH_2$), 44.9 ($CH_2$), 31.2 ($CH_2$).
CI-HRMS: *m/z* calculated for $C_{16}H_{17}ClNO_3S^+$ ([M+H]$^+$): 338.0612; found: 338.0618.

**3-((3-bromophenyl)sulfonyl)-6-phenyl-1,3-oxazinane** (**4y**)

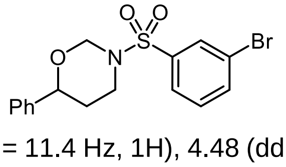

Obtained following the *General Procedure A*, as a white solid (49.0 mg, 64%).
$^1H$ NMR (501 MHz, $CDCl_3$) δ 8.16 (t, *J* = 1.8 Hz, 1H), 7.92 (ddd, *J* = 8.0, 1.8, 1.1 Hz, 1H), 7.75 (ddd, *J* = 8.0, 1.9, 1.0 Hz, 1H), 7.43 (t, *J* = 7.9 Hz, 1H), 7.32 – 7.23 (m, 3H), 7.06 – 7.02 (m, 2H), 5.73 (dd, *J* = 11.3, 2.2 Hz, 1H), 4.70 (d, *J* = 11.4 Hz, 1H), 4.48 (dd, *J* = 11.5, 2.6 Hz, 1H), 4.02 (ddt, *J* = 14.6, 4.6, 2.0 Hz, 1H), 3.45 (ddd, *J* = 14.6, 12.9, 3.3 Hz, 1H), 1.49 (dq, *J* = 13.9, 2.3 Hz, 1H), 1.37 – 1.23 (m, 1H).
$^{13}C$ NMR (126 MHz, $CDCl_3$) δ 142.8 (C), 140.8 (C), 136.0 (CH), 130.9 (CH), 130.8 (CH), 128.6 (CH), 128.2 (CH), 126.6 (CH), 125.7 (CH), 123.4 (C), 79.7 (CH), 78.5 ($CH_2$), 44.9 ($CH_2$), 31.2 ($CH_2$).
CI-HRMS: *m/z* calculated for $C_{16}H_{17}BrNO_3S^+$ ([M+H]$^+$): 382.0107; found: 382.0110.

**6-phenyl-3-((3-(trifluoromethyl)phenyl)sulfonyl)-1,3-oxazinane** (**4z**)

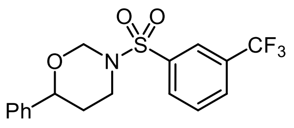

Obtained following the *General Procedure A*, as a white solid (25.0 mg, 34%).

$^{1}H$ NMR (501 MHz, $CDCl_3$) δ 8.27 (s, 1H), 8.19 (d, *J* = 7.9 Hz, 1H), 7.88 (d, *J* = 7.8 Hz, 1H), 7.71 (t, *J* = 7.9 Hz, 1H), 7.30 – 7.23 (m, 3H), 7.00 – 6.93 (m, 2H), 5.75 (dd, *J* = 11.3, 2.3 Hz, 1H), 4.72 (d, *J* = 11.4 Hz, 1H), 4.48 (dd, *J* = 11.4, 2.5 Hz, 1H), 4.06 (ddt, *J* = 14.7, 4.6, 2.1 Hz, 1H), 3.48 (ddd, *J* = 14.6, 12.9, 3.3 Hz, 1H), 1.51 (dq, *J* = 13.8, 2.7 Hz, 1H), 1.27 (tdd, *J* = 13.5, 11.5, 4.9 Hz, 1H).

$^{19}F$ NMR (471 MHz, $CDCl_3$) δ -62.75 (s, 3F).

$^{13}C$ NMR (126 MHz, $CDCl_3$) δ 142.2 (C), 140.6 (C), 132.2 (q, *J* = 33.4 Hz, C), 131.3 (CH), 130.1 (CH), 129.6 (q, *J* = 3.6 Hz, CH), 128.6 (CH), 128.2 (CH), 125.5 (CH), 125.0 (q, *J* = 3.8 Hz, CH), 123.4 (q, *J* = 273.0 Hz, C), 79.7 (CH), 78.5 ($CH_2$), 45.0 ($CH_2$), 31.1 ($CH_2$).

CI-HRMS: *m/z* calculated for $C_{17}H_{17}F_3NO_3S^+$ ($[M+H]^+$): 372.0876; found: 372.0884.

**3-((2-nitrophenyl)sulfonyl)-6-phenyl-1,3-oxazinane** (**4za**)

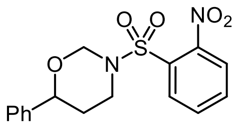

Obtained following the *General Procedure A*, as a white solid (45.2 mg, 61%).

$^{1}H$ NMR (501 MHz, $CDCl_3$) δ 8.19 – 8.15 (m, 1H), 7.76 – 7.71 (m, 2H), 7.67 – 7.61 (m, 1H), 7.34 – 7.24 (m, 3H), 7.17 – 7.12 (m, 2H), 5.66 (dd, *J* = 11.1, 2.3 Hz, 1H), 4.74 (d, *J* = 11.1 Hz, 1H), 4.56 (dd, *J* = 11.5, 2.6 Hz, 1H), 4.16 (ddt, *J* = 14.3, 4.7, 2.2 Hz, 1H), 3.44 (ddd, *J* = 14.2, 12.5, 3.2 Hz, 1H), 1.85 (dddd, *J* = 14.0, 12.6, 11.4, 4.8 Hz, 1H), 1.74 (dq, *J* = 14.0, 2.7 Hz, 1H).

$^{13}C$ NMR (126 MHz, $CDCl_3$) δ 140.8, 133.9, 133.7, 131.9, 131.3, 128.6, 128.2, 125.8, 124.2, 79.8, 78.5, 45.3, 32.2.

ESI-HRMS: *m/z* calculated for $C_{16}H_{16}N_2O_5SNa^+$ ($[M+Na]^+$): 371.0672; found: 371.0674.

**3-((3,5-difluorophenyl)sulfonyl)-6-phenyl-1,3-oxazinane** (**4zb**)

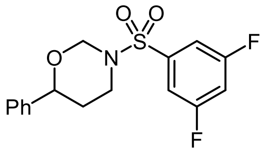

Obtained following the *General Procedure A*, as a white solid (38.0 mg, 53%).

$^{1}H$ NMR (501 MHz, $CDCl_3$) δ 7.57 – 7.51 (m, 2H), 7.33 – 7.25 (m, 3H), 7.10 – 7.03 (m, 3H), 5.70 (dd, *J* = 11.3, 2.3 Hz, 1H), 4.71 (d, *J* = 11.3 Hz, 1H), 4.50 (dd, *J* = 11.5, 2.6 Hz, 1H), 4.03 (ddt, *J* = 14.5, 4.6, 2.1 Hz, 1H), 3.47 (ddd, *J* = 14.6, 12.9, 3.3 Hz, 1H), 1.56 (dq, *J* = 13.9, 2.7 Hz, 1H), 1.36 (dddd, *J* = 13.9, 12.9, 11.4, 4.9 Hz, 1H).

$^{19}F$ NMR (471 MHz, $CDCl_3$) δ -105.51 (s, 2F).

$^{13}C$ NMR (126 MHz, $CDCl_3$) δ 163.9 (d, *J* = 11.5 Hz, C), 161.8 (d, *J* = 11.5 Hz, C), 143.8 (t, *J* = 8.2 Hz, C), 140.5 (C), 128.7 (CH), 128.2 (CH), 125.4 (CH), 111.7 (d, *J* = 7.3 Hz, CH), 111.5 (d, *J* = 7.2 Hz, CH), 108.7 (t, *J* = 24.9 Hz, CH), 79.6 (CH), 78.4 ($CH_2$), 44.9 ($CH_2$), 31.2 ($CH_2$).

CI-HRMS: *m/z* calculated for $C_{16}H_{16}F_2NO_3S^+$ ($[M+H]^+$): 340.0813; found: 340.0819.

**3-((3,5-dichlorophenyl)sulfonyl)-6-phenyl-1,3-oxazinane** (**4zc**)

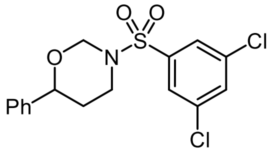

Obtained following the *General Procedure A*, as a white solid (30.7 mg, 39%).

$^{1}H$ NMR (501 MHz, $CDCl_3$) δ 7.89 (d, *J* = 1.9 Hz, 2H), 7.59 (t, *J* = 1.9 Hz, 1H), 7.35 – 7.24 (m, 3H), 7.13 – 7.04 (m, 2H), 5.73 (dd, *J* = 11.4, 2.3 Hz, 1H), 4.72 (d, *J* = 11.4 Hz, 1H), 4.52 (dd, *J* = 11.5, 2.6 Hz, 1H), 4.00 (ddt, *J* = 14.7, 4.8, 2.3 Hz, 1H), 3.48 (ddd, *J* = 14.8, 13.0, 3.3 Hz, 1H), 1.54 (dq, *J* = 14.0, 2.7 Hz, 1H), 1.36 – 1.24 (m, 1H).

$^{13}C$ NMR (126 MHz, $CDCl_3$) δ 143.8 (C), 140.7 (C), 136.3 (C), 133.0 (CH), 128.7 (CH), 128.3 (CH), 126.5 (CH), 125.5 (CH), 79.7 (CH), 78.5 ($CH_2$), 44.9 ($CH_2$), 31.4 ($CH_2$).
ESI-HRMS: *m/z* calculated for $C_{16}H_{15}Cl_2NO_3SNa^+$ ($[M+Na]^+$): 394.0042; found: 394.0045.

**6-phenyl-3-((3,4,5-trifluorophenyl)sulfonyl)-1,3-oxazinane** (**4zd**)

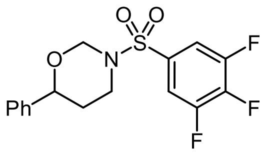

Obtained following the *General Procedure A*, as a white solid (40.0 mg, 56%). $^1H$ NMR (501 MHz, $CDCl_3$) δ 7.67 (t, *J* = 6.2 Hz, 2H), 7.37 – 7.25 (m, 3H), 7.12 – 7.05 (m, 2H), 5.69 (dd, *J* = 11.3, 2.3 Hz, 1H), 4.72 (d, *J* = 11.4 Hz, 1H), 4.53 (dd, *J* = 11.4, 2.6 Hz, 1H), 4.01 (ddt, *J* = 14.6, 4.6, 2.1 Hz, 1H), 3.48 (ddd, *J* = 14.6, 12.9, 3.3 Hz, 1H), 1.59 (dq, *J* = 13.9, 2.7 Hz, 1H), 1.41 – 1.31 (m, 1H).
$^{19}F$ NMR (471 MHz, $CDCl_3$) δ -129.56 (d, *J* = 20.1 Hz, 2F), -151.52 (t, *J* = 20.0 Hz, 1F).
$^{13}C$ NMR (126 MHz, $CDCl_3$) δ 140.5, 128.8, 128.3, 125.4, 113.3 (d, *J* = 6.2 Hz), 113.1 (d, *J* = 6.2 Hz), 79.6, 78.5, 45.0, 31.3. *(Other aromatic carbons could not be observed)*
CI-HRMS: *m/z* calculated for $C_{16}H_{15}F_3NO_3SNa^+$ ($[M+H]^+$): 358.0719; found: 358.0727.

**3-((perfluorophenyl)sulfonyl)-6-phenyl-1,3-oxazinane** (**4ze**)

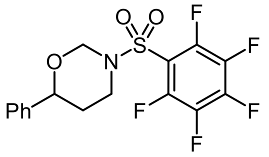

Obtained following the *General Procedure A*, as a white solid (40.0 mg, 51%). $^1H$ NMR (501 MHz, $CDCl_3$) δ 7.35 – 7.27 (m, 3H), 7.13 – 7.09 (m, 2H), 5.71 (dd, *J* = 11.2, 2.3 Hz, 1H), 4.77 (d, *J* = 11.2 Hz, 1H), 4.57 (dd, *J* = 11.4, 2.6 Hz, 1H), 4.25 (ddt, *J* = 14.3, 4.6, 2.1 Hz, 1H), 3.54 (ddd, *J* = 14.3, 12.8, 3.3 Hz, 1H), 1.79 (dq, *J* = 14.0, 2.7 Hz, 1H), 1.64 (tdd, *J* = 13.5, 11.4, 5.0 Hz, 1H).
$^{19}F$ NMR (471 MHz, $CDCl_3$) δ -134.60 (dt, *J* = 20.8, 6.0 Hz, 2F), -145.53 (tt, *J* = 20.9, 6.5 Hz, 1F), -157.93 – -158.85 (m, 2F).
$^{13}C$ NMR (126 MHz, $CDCl_3$) δ 140.4 (C), 128.8 (CH), 128.3 (CH), 125.2 (CH), 79.2 (CH), 78.2 ($CH_2$), 45.3 ($CH_2$), 31.8 ($CH_2$). *(Carbon atoms from the $C_6F_5$ unit were not visible)*
CI-HRMS: *m/z* calculated for $C_{16}H_{13}F_5NO_3S^+$ ($[M+H]^+$): 394.0531; found: 394.0530.

**3-(naphthalen-2-ylsulfonyl)-6-phenyl-1,3-oxazinane** (**4zf**)

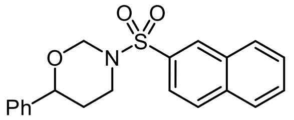

Obtained following the *General Procedure A*, as a white solid (35.4 mg, 48%).
$^1H$ NMR (501 MHz, $CDCl_3$) δ 8.55 (d, *J* = 1.8 Hz, 1H), 8.16 – 7.84 (m, 4H), 7.66 (dddd, *J* = 20.4, 8.2, 6.9, 1.3 Hz, 2H), 7.23 – 7.05 (m, 3H), 6.89 – 6.76 (m, 2H), 5.81 (dd, *J* = 11.2, 2.2 Hz, 1H), 4.71 (d, *J* = 11.2 Hz, 1H), 4.42 (dd, *J* = 11.3, 2.7 Hz, 1H), 4.11 (ddt, *J* = 14.5, 4.5, 2.1 Hz, 1H), 3.43 (ddd, *J* = 14.5, 12.7, 3.4 Hz, 1H), 1.45 – 1.24 (m, 2H).
$^{13}C$ NMR (126 MHz, $CDCl_3$) δ 140.8 (C), 137.7 (C), 135.1 (C), 132.5 (C), 129.51 (CH), 129.46 (CH), 129.3 (CH), 129.1 (CH), 128.5 (CH), 128.1 (CH), 128.0 (CH), 127.8 (CH), 125.7 (CH), 123.4 (CH), 79.7 (CH), 78.5 ($CH_2$), 44.9 ($CH_2$), 31.1 ($CH_2$).
ESI-HRMS: *m/z* calculated for $C_{20}H_{19}NO_3SNa^+$ ($[M+Na]^+$): 376.0978; found: 376.0977.

**3-(methylsulfonyl)-6-phenyl-1,3-oxazinane** (**4zg**)

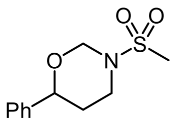

Obtained following the *General Procedure A*, as a white solid (26.0 mg, 54%).
$^1H$ NMR (501 MHz, $CDCl_3$) δ 7.37 (ddt, *J* = 7.8, 5.5, 1.3 Hz, 2H), 7.34 – 7.29 (m, 3H), 5.53 (dd, *J* = 11.6, 2.3 Hz, 1H), 4.74 (d, *J* = 11.6 Hz, 1H), 4.64 (dd, *J* = 11.3, 2.7 Hz, 1H), 4.14 (ddt, *J* = 14.8, 4.7, 2.0 Hz, 1H), 3.53 (ddd, *J* = 14.8, 12.9, 3.5 Hz, 1H), 3.10 (s, 3H), 1.96 (dddd, *J* = 14.0, 12.9, 11.4, 5.0 Hz, 1H), 1.87 – 1.80 (m, 1H).
$^{13}C$ NMR (126 MHz, $CDCl_3$) δ 140.9 (C), 128.8 (CH), 128.3 (CH), 125.6 (CH), 79.7 (CH), 78.2 ($CH_2$), 44.9 ($CH_2$), 42.0 ($CH_3$), 31.9 ($CH_2$).
CI-HRMS: *m/z* calculated for $C_{11}H_{16}NO_3S^+$ ($[M+H]^+$): 242.0845; found: 242.0850.

**3-(isopropylsulfonyl)-6-phenyl-1,3-oxazinane (4zh)**

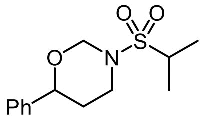

Obtained following the *General Procedure A*, as a white solid (39.0 mg, 71%).

$^1$H NMR (501 MHz, $CDCl_3$) δ 7.55 – 7.23 (m, 5H), 5.41 (dd, *J* = 11.0, 2.2 Hz, 1H), 4.69 (d, *J* = 10.9 Hz, 1H), 4.58 (dd, *J* = 11.4, 2.5 Hz, 1H), 4.01 (ddt, *J* = 14.2, 4.7, 2.2 Hz, 1H), 3.44 (ddd, *J* = 14.3, 12.5, 3.1 Hz, 1H), 3.19 (hept, *J* = 6.9 Hz, 1H), 1.98 (dddd, *J* = 13.7, 12.5, 11.4, 4.9 Hz, 1H), 1.83 (dq, *J* = 13.6, 2.6 Hz, 1H), 1.44 (dd, *J* = 21.2, 6.8 Hz, 6H).

$^{13}$C NMR (126 MHz, $CDCl_3$) δ 141.1 (C), 128.7 (CH), 128.2 (CH), 125.9 (CH), 79.7 (CH), 78.2 ($CH_2$), 54.5 (CH), 45.3 ($CH_2$), 33.3 ($CH_2$), 17.0 ($CH_3$), 16.6 ($CH_3$).

CI-HRMS: *m/z* calculated for $C_{13}H_{20}NO_3S^+$ ($[M+H]^+$): 270.1158; found: 270.1157.

**3-(*tert*-butylsulfonyl)-6-phenyl-1,3-oxazinane (4zi)**

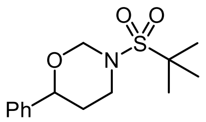

Obtained following the *General Procedure A*, as a white solid (30.0 mg, 53%).

$^1$H NMR (501 MHz, $CDCl_3$) δ 7.36 (d, *J* = 4.4 Hz, 4H), 7.30 (ddd, *J* = 8.8, 5.0, 3.8 Hz, 1H), 5.37 (dd, *J* = 10.7, 2.3 Hz, 1H), 4.69 (d, *J* = 10.6 Hz, 1H), 4.57 (dd, *J* = 11.4, 2.6 Hz, 1H), 4.03 (ddt, *J* = 13.9, 4.7, 2.2 Hz, 1H), 3.43 (ddd, *J* = 13.8, 12.4, 2.9 Hz, 1H), 2.04 (dddd, *J* = 13.7, 12.4, 11.4, 4.8 Hz, 1H), 1.82 (dq, *J* = 13.6, 2.5 Hz, 1H), 1.42 (s, 9H).

$^{13}$C NMR (126 MHz, $CDCl_3$) δ 141.2 (C), 128.7 (CH), 128.1 (CH), 126.0 (CH), 79.7 (CH), 79.0 ($CH_2$), 61.1 (C), 46.6 ($CH_2$), 33.7 ($CH_2$), 24.4 ($CH_3$).

CI-HRMS: *m/z* calculated for $C_{14}H_{22}NO_3S^+$ ($[M+H]^+$): 284.1315; found: 284.1313.

**3-(cyclopropylsulfonyl)-6-phenyl-1,3-oxazinane (4zj)**

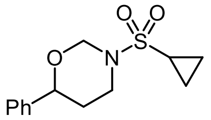

Obtained following the *General Procedure A*, as a white solid (34.7 mg, 65%).

$^1$H NMR (501 MHz, $CDCl_3$) δ 7.40 – 7.34 (m, 2H), 7.34 – 7.29 (m, 3H), 5.52 (dd, *J* = 11.5, 2.2 Hz, 1H), 4.73 (d, *J* = 11.5 Hz, 1H), 4.63 (dd, *J* = 11.4, 2.6 Hz, 1H), 4.08 (ddt, *J* = 14.7, 4.5, 2.0 Hz, 1H), 3.52 (ddd, *J* = 14.7, 12.9, 3.2 Hz, 1H), 2.56 (tt, *J* = 7.9, 4.9 Hz, 1H), 2.09 (dddd, *J* = 13.7, 12.9, 11.4, 4.9 Hz, 1H), 1.82 (dq, *J* = 13.8, 2.7 Hz, 1H), 1.37 – 1.23 (m, 2H), 1.16 – 1.03 (m, 2H).

$^{13}$C NMR (126 MHz, $CDCl_3$) δ 141.2 (C), 128.8 (CH), 128.2 (CH), 125.7 (CH), 79.6 (CH), 78.5 ($CH_2$), 45.2 ($CH_2$), 32.5 ($CH_2$), 31.5 (CH), 6.5 ($CH_2$), 6.0 ($CH_2$).

CI-HRMS: *m/z* calculated for $C_{13}H_{18}NO_3S^+$ ($[M+H]^+$): 268.1002; found: 268.1006.

**3-((fluoromethyl)sulfonyl)-6-phenyl-1,3-oxazinane (4zk)**

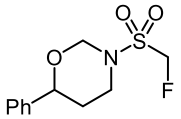

Obtained following the *General Procedure A*, as a white solid (11.0 mg, 21%).

$^1$H NMR (501 MHz, $CDCl_3$) δ 7.44 – 7.30 (m, 5H), 5.44 (dd, *J* = 11.4, 2.3 Hz, 1H), 5.35 (d, *J* = 10.0 Hz, 1H), 5.27 – 5.23 (m, 1H), 5.15 (d, *J* = 10.0 Hz, 1H), 4.77 (d, *J* = 11.5 Hz, 1H), 4.62 (d, *J* = 10.8 Hz, 1H), 4.16 (ddt, *J* = 14.5, 4.7, 2.1 Hz, 1H), 3.55 (ddd, *J* = 14.4, 12.8, 3.3 Hz, 1H), 2.14 – 2.04 (m, 1H), 1.83 (dq, *J* = 14.0, 2.7 Hz, 1H).

$^{19}$F NMR (471 MHz, $CDCl_3$) δ -211.07 (s, 1F).

$^{13}$C NMR (126 MHz, $CDCl_3$) δ 140.6 (C), 128.8 (CH), 128.4 (CH), 125.9 (CH), 89.7 (d, *J* = 215.1 Hz, $CH_2$), 80.0 (CH), 78.1 ($CH_2$), 45.5 ($CH_2$), 32.5 (d, *J* = 2.9 Hz, $CH_2$).

ESI-HRMS: *m/z* calculated for $C_{11}H_{14}FNO_3SNa^+$ ($[M+Na]^+$): 282.0571; found: 282.0572.

***N*,*N*-dimethyl-6-phenyl-1,3-oxazinane-3-sulfonamide (4zl)**

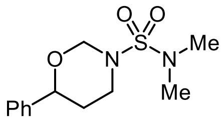

Obtained following the *General Procedure A*, as a white solid (21.0 mg, 39%).

$^1$H NMR (501 MHz, $CDCl_3$) δ 7.40 – 7.29 (m, 5H), 5.43 (dd, *J* = 11.1, 2.3 Hz, 1H), 4.67 (d, *J* = 11.1 Hz, 1H), 4.58 (dd, *J* = 11.5, 2.5 Hz, 1H), 3.91 (ddt, *J* = 14.3, 4.6,

2.1 Hz, 1H), 3.40 (ddd, *J* = 14.3, 12.7, 3.1 Hz, 1H), 2.88 (s, 6H), 2.04 (dddd, *J* = 13.7, 12.6, 11.4, 4.8 Hz, 1H), 1.77 (dq, *J* = 13.7, 2.6 Hz, 1H).
$^{13}$C NMR (126 MHz, $CDCl_3$) δ 141.1, 128.7, 128.1, 125.8, 79.7, 78.7, 45.2, 38.3, 32.2.
ESI-HRMS: *m/z* calculated for $C_{12}H_{18}N_2O_3SNa^+$ ([M+Na]$^+$): 293.0930; found: 293.0929.

**3-(((6-phenyl-1,3-oxazinan-3-yl)sulfonyl)methyl)benzo[*d*]isoxazole** (**4zm**)

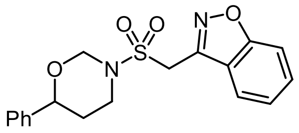

Obtained following the *General Procedure A*, as a white solid (50.1 mg, 70%).
$^1$H NMR (501 MHz, $CDCl_3$) δ 7.98 (dt, *J* = 8.0, 1.0 Hz, 1H), 7.64 – 7.58 (m, 2H), 7.42 – 7.28 (m, 6H), 5.29 (dd, *J* = 10.9, 2.2 Hz, 1H), 4.79 (d, *J* = 1.9 Hz, 2H), 4.59 (d, *J* = 10.9 Hz, 1H), 4.52 (dd, *J* = 11.4, 2.5 Hz, 1H), 3.96 (ddt, *J* = 14.1, 4.7, 2.1 Hz, 1H), 3.42 (ddd, *J* = 14.0, 12.7, 3.1 Hz, 1H), 2.02 (dddd, *J* = 13.8, 12.7, 11.4, 5.0 Hz, 1H), 1.77 (dq, *J* = 13.8, 2.6 Hz, 1H).
$^{13}$C NMR (126 MHz, $CDCl_3$) δ 164.0 (C), 149.5 (C), 140.6 (C), 130.7 (CH), 128.7 (CH), 128.3 (CH), 126.0 (CH), 124.5 (CH), 122.8 (CH), 120.8 (C), 110.1 (CH), 80.0 (CH), 78.1 ($CH_2$), 50.6 ($CH_2$), 45.2 ($CH_2$), 32.6 ($CH_2$).
ESI-HRMS: *m/z* calculated for $C_{18}H_{18}N_2O_4SNa^+$ ([M+Na]$^+$): 381.0879; found: 381.0879.

**(1*S*,4*R*)-7,7-dimethyl-1-((6-phenyl-1,3-oxazinan-3-yl)sulfonyl)bicyclo[2.2.1]heptan-2-one** (**4zna**)

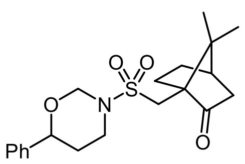

Obtained following the *General Procedure A*, as a mixture of two diastereomers in almost equal amounts (dr = 1.3:1). White solid (19.7 mg, 29% global yield).
*Diastereomer A (major)*
$^1$H NMR (600 MHz, $CDCl_3$) *δ* 7.39 – 7.28 (m, 5H), 5.54 (dd, *J* = 11.3, 2.2 Hz, 1H), 4.72 (d, *J* = 11.3 Hz, 1H), 4.61 (d, *J* = 11.5, 1H), 4.13 (ddt, *J* = 14.3, 4.7, 2.1 Hz, 1H), 3.72 (d, *J* = 14.6 Hz, 1H), 3.47 (ddd, *J* = 14.4, 12.8, 3.2 Hz, 1H), 2.93 (d, *J* = 14.3 Hz, 1H), 2.61 – 2.53 (m, 1H), 2.43 – 2.37 (m, 1H), 2.15 – 2.00 (m, 3H), 1.97 (d, *J* = 18.4 Hz, 1H), 1.85 – 1.80 (m, 1H), 1.70 – 1.65 (m, 1H), 1.47 – 1.42 (m, 1H), 1.17 (s, 3H), 0.92 (s, 3H).
$^{13}$C NMR (125 MHz, $CDCl_3$) *δ* 215.2, 141.1, 128.8, 128.2, 125.9, 79.8, 77.9, 58.9, 51.2, 47.9, 44.9, 43.1, 42.7, 32.7, 27.0, 25.2, 20.2, 19.9.
ESI-HRMS: *m/z* calculated for $C_{20}H_{27}NO_4SNa^+$ ([M+Na]$^+$): 400.1553; found: 400.1554.

*Diastereomer B (minor)*
$^1$H NMR (600 MHz, $CDCl_3$) *δ* 7.39 – 7.28 (m, 5H), 5.51 (dd, *J* = 11.3, 2.2 Hz, 1H), 4.70 (d, *J* = 11.3 Hz, 1H), 4.60 (d, *J* = 11.5 Hz, 1H), 4.17 (ddt, *J* = 14.7, 4.8, 2.1 Hz, 1H), 3.56 (d, *J* = 14.7 Hz, 1H), 3.52 (ddd, *J* = 14.7, 4.7, 2.1 Hz, 1H), 3.08 (d, *J* = 14.6 Hz, 1H), 2.61 – 2.53 (m, 1H), 2.43 – 2.37 (m, 1H), 2.15 – 2.00 (m, 3H), 1.97 (d, *J* = 18.4 Hz, 1H), 1.85 – 1.80 (m, 1H), 1.70 – 1.65 (m, 1H), 1.47 – 1.42 (m, 1H), 1.17 (s, 3H), 0.92 (s, 3H).
$^{13}$C NMR (125 MHz, $CDCl_3$) *δ* 215.2, 141.0, 128.8, 128.2, 125.9, 79.9, 78.0, 58.8, 50.9, 48.1, 44.8, 42.9, 42.7, 32.2, 27.1, 25.2, 20.2, 19.9.
ESI-HRMS: *m/z* calculated for $C_{20}H_{27}NO_4SNa^+$ ([M+Na]$^+$): 400.1553; found: 400.1554.

**Phenyl 6-phenyl-1,3-oxazinane-3-carboxylate** (**6a**)

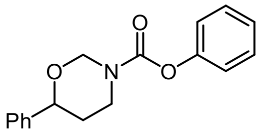

Obtained by adapting the *General Procedure B* using 3 equiv. of carbamate **5a**, 20 mol% of $HPF_6$, and 2 mL of $CHCl_3$ as a white solid (19.0 mg, 34%).
NMR at room temperature (298 K): broad bands, rotameric mixture.
NMR at 233 K (–40 °C) shows two main rotamers (ratio approx. 65:35).
$^1$H NMR (600 MHz, $CDCl_3$, 233 K) δ 7.44 – 7.37 (m, 6H), 7.37 – 7.32 (m, 1H), 7.27 – 7.23 (m, 1H), 7.17 – 7.12 (m, 2H), 5.92 (dd, *J* = 10.6, 2.1 Hz, 0.65H, *major*), 5.87 (dd, *J* = 10.2, 2.1 Hz, 0.37H, *minor*), 4.75 (d, *J* = 10.6 Hz, 0.66H, *major*), 4.66 (dd, *J* = 11.6, 2.2 Hz, 1H), 4.59 (d, *J* = 10.3 Hz, 0.36H, *minor*), 4.53

(ddt, *J* = 13.7, 4.5, 2.1 Hz, 0.39H, *minor*), 4.46 (ddt, *J* = 13.5, 4.5, 2.0 Hz, 0.67H, *major*), 3.47 (ddd, *J* = 13.7, 12.5, 3.1 Hz, 0.39H, *minor*), 3.30 (ddd, *J* = 13.5, 12.5, 3.1 Hz, 0.66H, *major*), 2.02 (tddd, *J* = 21.2, 12.5, 11.3, 4.8 Hz, 1H), 1.89 (ddq, *J* = 13.3, 9.9, 2.6 Hz, 1H).
$^{13}$C NMR (151 MHz, $CDCl_3$, 233 K) δ 153.3 (C, *minor*), 153.1 (C, *major*), 150.74 (C, *major*), 150.71 (C, *minor*), 141.0 (C, *minor*), 140.7 (C, *major*), 129.5 (CH), 128.73 (CH, *major*), 128.65 (CH, *minor*), 128.3 (CH, *major*), 128.1 (CH, *minor*), 126.0 (CH, *major*), 125.84 (CH, *minor*), 125.80 (CH, *major*), 125.78 (CH, *minor*), 121.9 (CH, *major*), 121.8 (CH, *minor*), 79.9 (CH, *major*), 79.7 (CH, *minor*), 76.8 ($CH_2$, *major*), 76.5 ($CH_2$, *minor*), 43.7 ($CH_2$, *minor*), 43.1 ($CH_2$, *major*), 33.5 ($CH_2$, *minor*), 32.7 ($CH_2$, *major*).
CI-HRMS: *m/z* calculated for $C_{17}H_{18}NO_3^+$ ($[M+H]^+$): 284.1281; found: 284.1281

**Benzyl 6-phenyl-1,3-oxazinane-3-carboxylate** (**6b**)

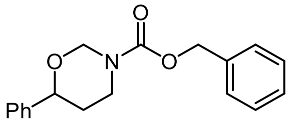

Obtained by adapting the *General Procedure B* using 3 equiv. of carbamate **5b**, 20 mol% of $HPF_6$, and 2 mL of $CHCl_3$ as a white solid (30 mg, 23%)
NMR at room temperature (298 K): broad bands, rotameric mixture.
NMR at 233 K (–40 °C) shows two main rotamers (ratio approx. 65:35).
$^{1}$H NMR (600 MHz, $CDCl_3$, 233K) δ 7.42 – 7.29 (m, 10H), 5.84 (dd, *J* = 10.3, 2.0 Hz, 0.37H), 5.73 (dd, *J* = 10.5, 2.1 Hz, 0.62H), 5.23 (d, *J* = 12.2 Hz, 0.60H), 5.20 (d, *J* = 12.2 Hz, 0.40H), 5.14 (d, *J* = 4.6 Hz, 0.60H), 5.12 (d, *J* = 4.6 Hz, 0.40H), 4.59 (dd, *J* = 11.4, 2.4 Hz, 1H), 4.58 (d, *J* = 10.6 Hz, 0.62H), 4.50 (d, *J* = 10.3 Hz, 0.38H), 4.41 (ddt, *J* = 13.5, 4.5, 2.0 Hz, 0.64H), 4.32 (ddt, *J* = 13.7, 4.5, 2.1 Hz, 0.39H), 3.29 (ddd, *J* = 13.7, 12.4, 3.1 Hz, 0.37H), 3.20 (td, *J* = 13.0, 3.0 Hz, 0.62H), 1.97 – 1.89 (m, 0.62H), 1.88 – 1.73 (m, 1.40H).
$^{13}$C NMR (151 MHz, $CDCl_3$, 233K) δ 154.8 (C, *minor*), 154.6 (C, *major*), 141.1 (C, *minor*), 140.9 (C, *major*), 136.04 (C, *minor*), 136.02 (C, *major*), 128.65 (CH, *major*), 128.64 (CH, *minor*), 128.61 (CH, *major*), 128.60 (CH, *minor*), 128.3 (CH), 128.3 (CH), 128.2 (CH), 128.2 (CH), 128.1 (CH), 128.0 (CH), 126.0 (CH, *major*), 125.9 (CH, *minor*), 79.9 (CH, *major*), 79.7 (CH, *minor*), 76.5 ($CH_2$, *major*), 76.4 ($CH_2$, *minor*), 67.52 ($CH_2$, *major*), 67.49 ($CH_2$, *minor*), 43.2 ($CH_2$, *minor*), 43.0 ($CH_2$, *major*), 33.4 ($CH_2$, *minor*), 32.8 ($CH_2$, *major*).
ESI-HRMS: *m/z* calculated for $C_{18}H_{19}NO_3Na^+$ ($[M+Na]^+$): 320.1257; found: 320.1255.

**(9*H*-fluoren-9-yl)methyl 6-phenyl-1,3-oxazinane-3-carboxylate** (**6c**)

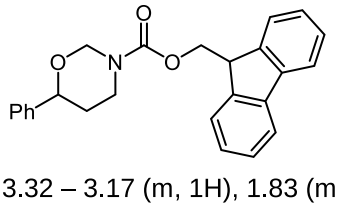

Obtained by adapting the *General Procedure B* using 1 mmol of olefin **1a** and 3 equiv. of carbamate **5c**, as a white solid (136 mg, 37%)
NMR at room temperature (298 K): broad bands, rotameric mixture.
$^{1}$H NMR (501 MHz, $CDCl_3$) δ 7.78 (d, *J* = 7.5 Hz, 2H), 7.69 – 7.57 (m, 2H), 7.45 – 7.28 (m, 9H), 5.77 (br d, *J* = 12.2 Hz, 1H), 4.70 – 4.14 (m, 6H), 3.32 – 3.17 (m, 1H), 1.83 (m, 2H).
$^{13}$C NMR (126 MHz, $CDCl_3$) δ 154.8 (C), 144.1 (C), 144.0 (C), 141.5 (C), 141.4 (CH), 128.6 (CH), 128.0 (CH), 127.9 (CH), 127.3 (CH), 127.2 (CH), 126.0 (CH), 125.2 (CH), 120.2 (CH), 120.1 (CH), 80.0 (CH), 76.8 ($CH_2$), 67.9 ($CH_2$), 47.4 (CH), 43.5 ($CH_2$), 33.2 ($CH_2$).
ESI-HRMS: *m/z* calculated for $C_{25}H_{23}NO_3Na^+$ ($[M+Na]^+$): 408.1570; found: 408.1570.

**Benzyl 6-phenyl-1,3-oxazinane-3-carboxylate** (**6d**)

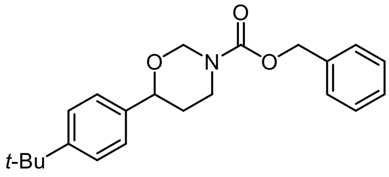

Obtained by adapting the *General Procedure B* using 1 mmol of olefin **1b** and 3 equiv. of carbamate **5b** as a colorless oil (130 mg, 39%).
NMR at room temperature (298 K): broad bands, rotameric mixture.

$^1$H NMR (501 MHz, $CDCl_3$) δ 7.46 – 7.17 (m, 10H), 5.77 (m, 0.6H), 5.36 – 4.99 (m, 2.6H), 4.53 (dd, *J* = 11.3, 2.5 Hz, 1H), 4.51 – 4.25 (m, 1H), 3.41 – 3.06 (m, 0.7H), 2.04 – 1.69 (m, 1.8H), 1.31 (s, 9H).
$^{13}$C NMR (126 MHz, $CDCl_3$) δ 155.3 (C), 154.8 (C), 150.9 (C), 150.3 (C), 138.3 (C), 136.6 (C), 128.6 (CH), 128.5 (CH), 128.2 (CH), 128.1 (CH), 127.8 (CH), 125.8 (CH), 125.8 (CH), 125.6 (CH), 125.5 (CH), 79.65 (CH), 76.8 ($CH_2$), 67.5 ($CH_2$), 66.7 (C), 43.3 ($CH_2$), 34.6 (C), 31.4 ($CH_3$).
ESI-HRMS: *m/z* calculated for $C_{22}H_{27}NO_3Na^+$ ($[M+Na]^+$): 376.1883; found: 376.1882.

**(9*H*-fluoren-9-yl)methyl 6-phenyl-1,3-oxazinane-3-carboxylate** (**6e**)

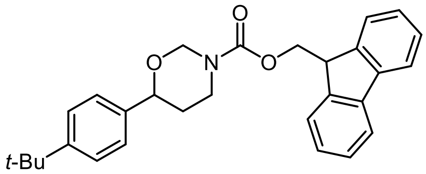


Obtained following the *General Procedure B*, as a white solid (75.0 mg, 35%).
NMR at room temperature (298 K): broad bands, rotameric mixture.
$^1$H NMR (501 MHz, $CDCl_3$) δ 7.78 (d, *J* = 7.5 Hz, 2H), 7.62 (m, 2H), 7.44 – 7.36 (m, 4H), 7.36 – 7.27 (m, 4H), 5.75 (m, 1H), 4.71 – 4.46 (m, 3.6H), 4.44 – 4.34 (m, 1.3H), 4.33 – 4.27 (m, 1.2H), 4.25 – 4.14 (m, 0.4H), 3.31 – 3.11 (m, 1H), 1.96 (m, 0.7H), 1.85 – 1.74 (m, 1.5H), 1.33 (s, 9H).
$^{13}$C NMR (126 MHz, $CDCl_3$) δ 154.9 (C), 151.1 (C), 144.1 (C), 144.0 (C), 141.5 (C), 138.3 (C), 127.9 (CH), 127.3 (CH), 125.9 (CH), 125.6 (CH), 125.2 (CH), 120.2 (CH), 79.9 (CH), 76.8 ($CH_2$), 67.9 ($CH_2$), 47.4 (CH), 43.5 ($CH_2$), 34.7 (C), 31.5 ($CH_3$).
ESI-HRMS: *m/z* calculated for $C_{29}H_{31}NO_3Na^+$ ($[M+Na]^+$): 464.2196; found: 464.2196.

**Benzyl 6-phenyl-1,3-oxazinane-3-carboxylate** (**6f**)

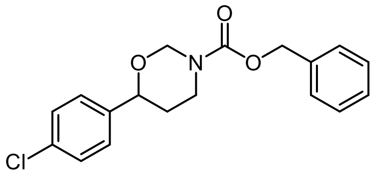


Obtained by adapting the *General Procedure B* using 1 mmol of olefin **1f** as a white solid (50.2 mg, 15%).
NMR at room temperature (298 K): broad bands, rotameric mixture.
$^1$H NMR (501 MHz, $CDCl_3$) δ 7.41 – 7.33 (m, 4H), 7.34 – 7.28 (m, 3H), 7.26 (d, *J* = 8.2 Hz, 2H), 5.77 (m, 1H), 5.25 – 5.11 (m, 2H), 4.54 (dd, *J* = 10.7, 3.2 Hz, 1H), 4.52 – 4.43 (m, 0.5H), 4.42 – 4.23 (m, 1.5H), 3.30 – 3.10 (m, 0.6H), 1.90 – 1.70 (m, 2.1H).
$^{13}$C NMR (126 MHz, $CDCl_3$) δ 154.8 (C), 139.9 (C), 136.5 (C), 133.7 (CH), 128.8 (CH), 128.7 (CH), 128.3 (CH), 128.2 (CH), 128.2 (CH), 127.3 (CH), 79.1 (CH), 76.8 ($CH_2$), 67.7 ($CH_2$), 43.3 ($CH_2$).
ESI-HRMS: *m/z* calculated for $C_{18}H_{18}NO_3ClNa^+$ ($[M+Na]^+$): 354.0867; found: 354.0867.

**(9*H*-fluoren-9-yl)methyl 6-phenyl-1,3-oxazinane-3-carboxylate** (**6g**)

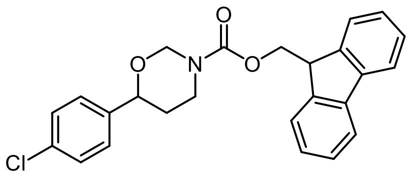


Obtained following the *General Procedure B*, as a white solid (37.8 mg, 45%).
NMR at room temperature (298 K): broad bands, rotameric mixture.
$^1$H NMR (501 MHz, $CDCl_3$) δ 7.77 (d, J = 7.6 Hz, 2H), 7.61 (s, 2H), 7.41 (t, J = 7.4 Hz, 2H), 7.37 – 7.24 (m, 6H), 5.84 – 5.68 (m, 1H), 4.65 – 4.45 (m, 3H), 4.47 – 4.34 (m, 1.4H), 4.33 – 4.23 (s, 1.1H), 4.20 – 4.09 (s, 0.4H), 3.30 – 3.14 (m, 1H), 1.91 – 1.57 (m, 2.1H).
$^{13}$C NMR (126 MHz, $CDCl_3$) δ 143.9, 141.5, 139.9, 128.8, 127.9, 127.3, 127.3, 125.2, 120.2, 76.7, 47.4, 43.4.
ESI-HRMS: *m/z* calculated for $C_{25}H_{22}NO_3ClNa^+$ ($[M+Na]^+$): 442.1180; found: 442.1183.

**Benzyl 6-phenyl-1,3-oxazinane-3-carboxylate** (**6h**)

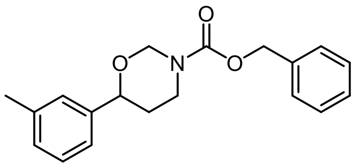

Obtained by adapting the *General Procedure B* using 1 mmol of olefin **1h** as a white solid (82.9 mg, 27%).

NMR at room temperature (298 K): broad bands, rotameric mixture.

$^{1}$H NMR (501 MHz, $CDCl_3$) δ 7.40 – 7.27 (m, 5H), 7.22 (t, *J* = 7.6 Hz, 1H), 7.16 (s, 1H), 7.10 (t, *J* = 8.2 Hz, 2H), 5.92 – 5.58 (m, 1H), 5.24 – 5.12 (m, 2H), 4.57 – 4.43 (m, 2H), 4.42 – 4.25 (m, 1H), 3.32 – 3.04 (m, 1H), 2.34 (s, 3H), 1.98 – 1.81 (m, 1H), 1.81 – 1.69 (m, 1H).

$^{13}$C NMR (126 MHz, $CDCl_3$) δ 154.9 (C), 141.4 (C), 138.3 (C), 136.6 (C), 128.8 (CH), 128.7 (CH), 128.5 (CH), 128.3 (CH), 128.2 (CH), 126.7 (CH), 123.1 (CH), 79.9 (CH), 76.8 ($CH_2$), 67.6 ($CH_2$), 43.4 ($CH_2$), 33.5 ($CH_2$), 33.1 ($CH_2$), 21.7 ($CH_3$).

ESI-HRMS: *m/z* calculated for $C_{19}H_{21}NO_3Na^+$ ($[M+Na]^+$): 334.1414; found: 334.1413.

**(9*H*-fluoren-9-yl)methyl 6-phenyl-1,3-oxazinane-3-carboxylate** (**6i**)

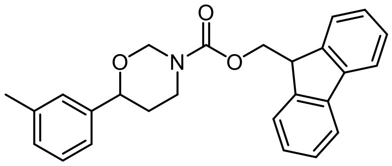

Obtained following the *General Procedure B*, as a white solid (54 mg, 28%).

NMR at room temperature (298 K): broad bands, rotameric mixture.

$^{1}$H NMR (501 MHz, $CDCl_3$) δ 7.77 (d, *J* = 7.5 Hz, 2H), 7.67 – 7.55 (m, 3H), 7.48 – 7.29 (m, 4H), 7.29 – 7.21 (m, 1H), 7.21 – 7.01 (m, 3H), 5.83 – 5.71 (m, 1H), 4.65 – 4.46 (m, 4H), 4.45 – 4.34 (m, 1.3H), 4.34 – 4.23 (m, 1.2H), 4.24 – 4.11 (m, 0.5H), 3.29 – 3.13 (m, 1H), 2.36 (s, 3H), 1.99 – 1.85 (m, 0.6H), 1.85 – 1.69 (m, 1.4H).

$^{13}$C NMR (126 MHz, $CDCl_3$) δ 154.8, 144.0, 141.5, 141.3, 138.3, 128.8, 128.5, 127.9, 127.3, 127.2, 126.7, 125.2, 123.1, 120.2, 120.1, 80.1, 76.8, 67.9, 47.4, 43.5, 33.2, 21.6.

ESI-HRMS: *m/z* calculated for $C_{26}H_{25}NO_3Na^+$ ($[M+Na]^+$): 422.1727; found: 422.1726.

**Benzyl 6-phenyl-1,3-oxazinane-3-carboxylate** (**6j**)

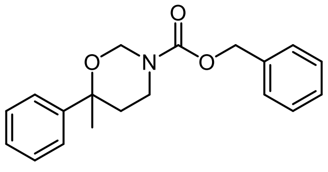

Obtained by adapting the *General Procedure B* using 0.7 mmol of olefin **1p** and 0.65 mL $CHCl_3$ as a colorless oil (75 mg, 34%).

NMR at room temperature (298 K): broad bands, rotameric mixture.

$^{1}$H NMR (500 MHz, $CDCl_3$) δ 7.40 – 7.38 (m, 4H), 7.37 – 7.35 (m, 4H), 7.34 – 7.29 (m, 3H), 7.29 – 7.26 (m, 1H), 5.30 (d, *J* = 10.5 Hz, 1H), 5.18 (d, *J* = 5.7 Hz, 2H), 4.59 (d, *J* = 10.5 Hz, 1H), 4.01 – 3.92 (m, 1H), 3.26 (t, *J* = 11.4 Hz, 1H), 2.31 (d, *J* = 14.0 Hz, 1H), 2.01 (ddd, *J* = 14.7, 10.3, 3.8 Hz, 1H), 1.46 (s, 3H).

$^{13}$C NMR (151 MHz, $CDCl_3$) δ 155.0 (C), 143.6 (C), 136.6 (C), 128.9 (CH), 128.6 (CH), 128.5 (CH), 128.2 (CH), 128.1 (CH), 128.0 (CH), 127.3 (CH), 125.9 (CH), 124.9 (CH), 76.7 (C), 71.3 ($CH_2$), 67.4 ($CH_2$), 40.1 ($CH_2$), 33.8 ($CH_2$), 32.2 ($CH_3$).

ESI-HRMS: *m/z* calculated for $C_{19}H_{21}NO_3Na^+$ ($[M+Na]^+$): 334.1414; found: 334.1415.

**(9*H*-fluoren-9-yl)methyl 6-phenyl-1,3-oxazinane-3-carboxylate** (**6k**)

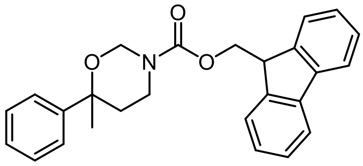

Obtained by adapting the *General Procedure B* using 0.75 mL $CHCl_3$ as a white solid (56.1 mg, 27%).

NMR at room temperature (298 K): broad bands, rotameric mixture.

$^{1}$H NMR (501 MHz, $CDCl_3$) δ 7.78 (d, *J* = 7.5 Hz, 2H), 7.62 (m, *J* = 7.7 Hz, 2H), 7.48 – 7.38 (m, 6H), 7.32 (m, *J* = 7.4, 5.5, 1.1 Hz, 3H), 5.34 (d, *J* = 10.5 Hz, 1H), 4.63 (d, *J* = 10.7 Hz, 0.5H), 4.54 – 4.37 (m, 2.5H), 4.33 – 4.26 (m, 1H), 4.19 – 4.13 (m, 0.1H), 4.04 (d, *J* = 13.7 Hz, 0.5H), 3.83 (d, *J* = 13.3 Hz, 0.3H), 3.72 (s, 0.5H), 3.23 (ddd, *J* = 13.9, 11.3, 3.1 Hz, 1H), 2.57 (s, 0.1H), 2.40 – 2.32 (m, 0.6H), 2.26 (d, *J* = 13.9 Hz, 0.3H), 2.11 – 1.98 (m, 0.6H), 1.87 (p, *J* = 7.5 Hz, 0.4H), 1.46 (d, *J* = 22.0 Hz, 3H).

$^{13}C$ NMR (126 MHz, $CDCl_3$) δ 155.1 (C), 144.2 (C), 144.4 (C), 141.6 (C), 128.9 (CH), 127.9 (CH), 127.3 (CH), 125.9 (CH), 125.2 (CH), 120.1 (CH), 76.7 (C), 71.3 ($CH_2$), 67.8 ($CH_2$), 47.6 (CH), 40.3 ($CH_2$), 34.2 ($CH_2$), 31.8 ($CH_3$).
ESI-HRMS: *m/z* calculated for $C_{26}H_{25}NO_3Na^+$ ($[M+Na]^+$): 422.1727; found: 422.1728.

## 5. Synthesis of 1,3-Amino Alcohols: Deprotection and Ring Opening

**_N_-(3-hydroxy-3-phenylpropyl)-4-methylbenzenesulfonamide (7a)**

*(Obtained adapting a reported procedure[6])*: the substrate **4a** (158.7 mg, 0.5 mmol) was dissolved in MeOH (1 mL) and treated with concentrated HCl 37% (80 µL, 1 mmol, 2 equiv.). The mixture was heated at 72 °C for 6 h and then to 95 °C during another hour while the MeOH was distilled off. After cooling to room temperature, the mixture was treated with water (5 mL), extracted with toluene, and made basic with excess aq. 50% NaOH. The mixture was taken up in toluene and concentrated under reduced pressure to give a crude product. Further purification by silica gel column chromatography with *iso*-hexane/ethyl acetate provides the desired product **7a**.

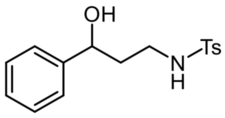


Obtained as a white solid (48.0 mg, 89%).
$^{1}$H NMR (300 MHz, $CDCl_3$) δ 7.68 (d, *J* = 8.3 Hz, 2H), 7.36 – 7.08 (m, 7H), 5.13 (s, 1H), 4.73 (t, *J* = 6.3 Hz, 1H), 3.03 (ddt, *J* = 24.8, 12.7, 6.2 Hz, 2H), 2.36 (s, 3H), 1.78 (q, *J* = 6.1 Hz, 2H).
$^{13}$C NMR (75 MHz, $CDCl_3$) δ 143.7 (C), 143.3 (C), 136.9 (C), 129.7 (CH, 128.6 ($CH_2$), 127.8 (CH), 127.1 (CH), 125.5 (CH), 73.2 (CH), 40.8 ($CH_2$), 37.7 ($CH_2$), 21.5 ($CH_3$). Spectroscopic data was consistent with the values reported in the literature.[7]
ESI-HRMS: *m/z* calculated for $C_{16}H_{19}NO_3S^+$ ([M]$^+$): 304.1013; found: 304.1013.

**3-amino-1-phenylpropan-1-ol (7b)**

*(Obtained adapting a reported procedure[6])*: a mixture of freshly washed Mg powder (60.8 mg, 2.5 mmol, 5 equiv.) and **4a** (158.7 mg, 0.5 mmol) in a 10 mL glass tube was dissolved with a MeOH-THF mixture (5 mL total, 2.5:1 *v/v*) and sonicated at room temperature for 3 hours, until completion of the starting material was observed on TLC. The mixture was filtered over silica, washed with ethyl acetate (2×1 mL) and evaporated under reduced pressure. The crude deprotected product was then dissolved in MeOH (1 mL) and treated with concentrated HCl 37% (80 µL, 1 mmol, 2 equiv.). The mixture was heated at 72 °C for 6 h and then to 95 °C during another hour while the MeOH was distilled off. After cooling to room temperature, the mixture was treated with water (5 mL), extracted with toluene, and made basic with excess aq. 50% NaOH. The free amine was taken up in toluene and concentrated under reduced pressure to give a crude product. Further purification by silica gel column chromatography with *iso*-hexane/ethyl acetate provides the desired product **7b**.

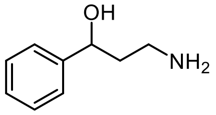


Obtained as a colorless viscous oil (56.6 mg, 75%).
$^{1}$H NMR (501 MHz, $CDCl_3$) δ 7.40 – 7.31 (m, 4H), 7.27 – 7.22 (m, 1H), 4.95 (dd, *J* = 8.7, 3.2 Hz, 1H), 3.09 (ddd, *J* = 12.4, 5.9, 4.1 Hz, 1H), 3.03 (br s, 3H), 2.96 (ddd, *J* = 12.7, 9.2, 3.9 Hz, 1H), 1.87 (ddt, *J* = 14.5, 6.0, 3.6 Hz, 1H), 1.77 (dtd, *J* = 14.4, 8.9, 4.0 Hz, 1H).
$^{13}$C NMR (126 MHz, $CDCl_3$) δ 145.2 (C), 128.4 (CH), 127.2 (CH), 125.8 (CH), 75.5 (CH), 40.6 ($CH_2$), 39.7 ($CH_2$). Spectroscopic data was consistent with the values reported in the literature.[8-9]

**_N_-(3-hydroxy-3-phenylpropyl)-N,4-dimethylbenzenesulfonamide (7c)**

A flame-dried Schlenk flask under argon was charged with **1a** (31.7 mg, 0.1 mmol) and dry toluene (1 mL). Diisobutylaluminium hydride (1.2 M in toluene, 0.41 mL, 0.5 mmol, 5.0 equiv.) was then added slowly and the reaction mixture was refluxed overnight, when full conversion of the starting material was observed by TLC. The mixture was cooled down to room temperature and quenched with aq. sat. $NH_4Cl$ (5 mL). The phases were separated and the aqueous phase extracted with ethyl acetate (3×10 mL). The combined organic layers were washed with brine, dried over $Na_2SO_4$ and concentrated under reduced pressure. The residue was purified by silica gel column chromatography (pentane/$Et_2O$ 5:1) to yield product **7c** (80% yield, 25.5 mg).

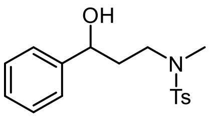


Obtained as a yellowish oil (25.5 mg, 80%).

$^1$H NMR (400 MHz, $CDCl_3$) δ 7.72 – 7.65 (m, 2H), 7.42 – 7.27 (m, 7H), 4.90 (dd, *J* = 9.1, 4.1 Hz, 1H), 3.50 – 3.45 (m, 1H), 2.92 – 2.86 (m, 1H), 2.78 (s, 3H), 2.66 (s, 1H), 2.44 (s, 3H), 2.02 – 1.76 (m, 2H).

$^{13}$C NMR (101 MHz, $CDCl_3$) δ = 144.2, 143.6, 134.4, 129.9, 128.7, 127.7, 127.6, 125.9, 70.9, 47.3, 37.1, 35.3, 21.3. Spectroscopic data was consistent with the values reported in the literature.[10]

**3-(methylamino)-1-phenylpropan-1-ol (7d)**

A flame-dried Schlenk flask under argon was charged with Lithium aluminum hydride (38.0 mg, 1 mmol, 5.0 equiv.) in 1 mL dry THF. A solution of **1a** (63.5 mg, 0.2 mmol) in dry THF (1 mL) was slowly added to the flask. After refluxing for 3 days (the reduction was monitored by means of TLC), the mixture was quenched with 1.5 mL of water under ice cooling. 2 mL of aq. 20% sodium potassium tartrate were added, and the mixture was stirred at room temperature for 30 min. The phases were then separated and the aqueous phase was extracted with ethyl acetate (3×5 mL). The combined organic layers were washed with brine, dried over $Na_2SO_4$ and concentrated under reduced pressure to afford product **7d** (85% yield, 28.0 mg).

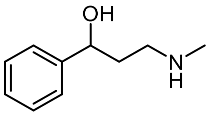


Obtained as a yellowish oil (28.0 mg, 85%).

$^1$H NMR (501 MHz, $CDCl_3$) δ 7.43 – 7.31 (m, 4H), 7.30 – 7.24 (m, 1H), 4.96 (dd, *J* = 8.7, 3.2 Hz, 1H), 3.81 (bs, 2H), 2.96 – 2.83 (m, 2H), 2.47 (s, 3H), 1.90 (ddt, *J* = 14.6, 5.8, 3.3 Hz, 1H), 1.79 (dtd, *J* = 14.5, 9.2, 3.8 Hz, 1H).

$^{13}$C NMR (101 MHz, $CDCl_3$) δ = 144.9, 128.39, 127.16, 125.7, 74.9, 50.0, 36.5, 35.7. Spectroscopic data was consistent with the values reported in the literature.[11]

## 6. Current Limitations

Several olefins proved to be challenging, since they are at the edges of the reactivity: some of them are either too reactive and produce complex mixtures, whereas others display very low reactivity and form only traces of product or no product at all.

In addition to sulfonamides and carbamates, other types of nitrogen nucleophiles were tested, but proved incompatible under the reaction conditions: either no reaction occurred or complex mixtures were formed. These limitations are summarized in Figure S-1.

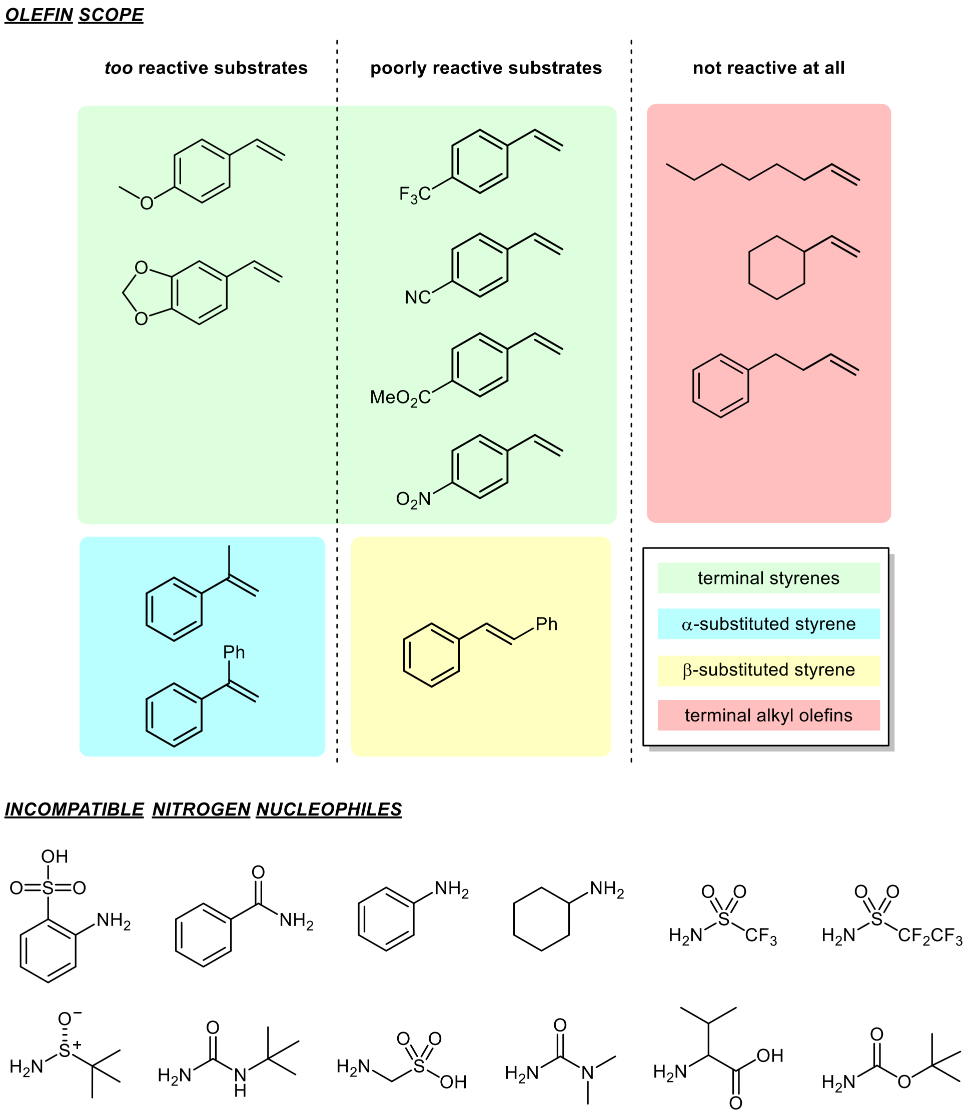


*Figure S-1. Current substrate limitations of the three-component olefin oxy-aminomethylation.*

## 7. Mechanistic Studies

### *7.1. Preliminary Monitoring of the Three-Component Reaction*

To gain some insight on the acid-catalyzed three-component olefin oxy-aminomethylation, we conducted a $^1$H NMR monitoring of the reaction mixture over time.

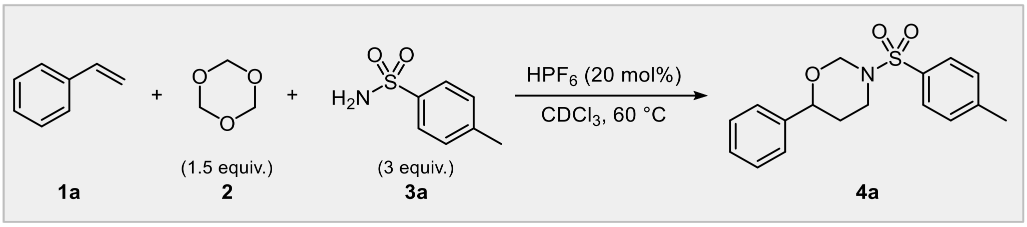


*Procedure:* Styrene (**1a**, 0.02 mmol), *sym*-trioxane (**2**, 0.03 mmol, 1.5 equiv.), and *p*-toluenesulfonamide (**3a**, 0.06 mmol, 3 equiv.) were added into a J-Young NMR tube, followed by adding 0.5 mL $CDCl_3$ and $HPF_6$ (20 mol%). The reaction mixture was analyzed at different times in a 300 MHz NMR spectrometer at 60 °C.

Under these conditions, in addition to the desired product **4a**, also some products from sym-trioxane/sulfonamide condensation were obtained (see Figure S-2), namely 5-tosyl-1,3,5-dioxazinane (**8a**), 3,5-ditosyl-1,3,5-oxadiazinane (**8b**), and 1,3,5-tritosyl-1,3,5-triazinane (**8c**).

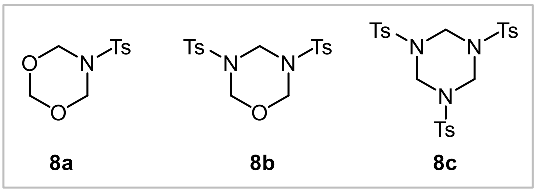


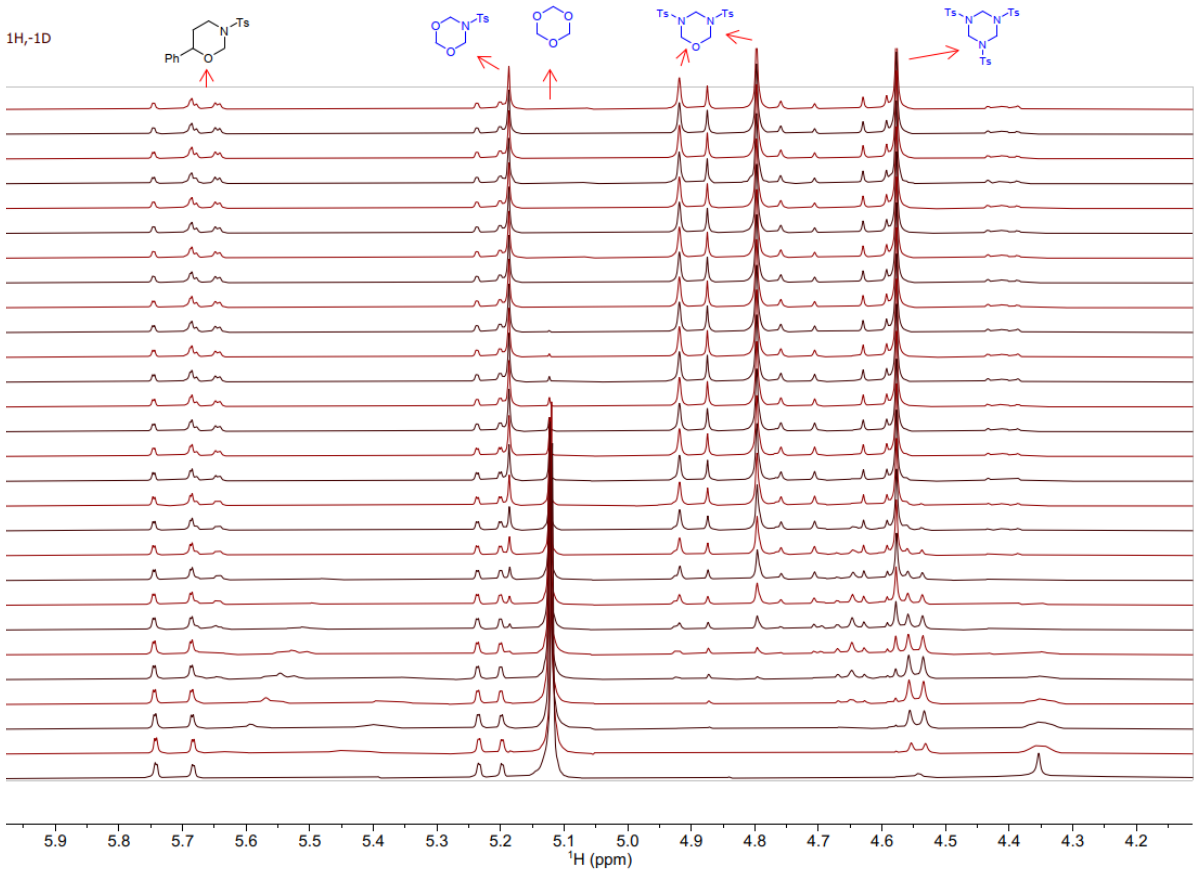


*Figure S-2. $^1$H NMR monitoring of the three-component reaction.*

### 7.2. On the Oxy-Aminomethylating Species: Experiments on the Formaldehyde-Sulfonamide Condensation

To further study the actual nature of the electrophile, we performed several $^1$H NMR monitoring experiments in the absence of the olefin.

**Reaction between 2 and 3a:**

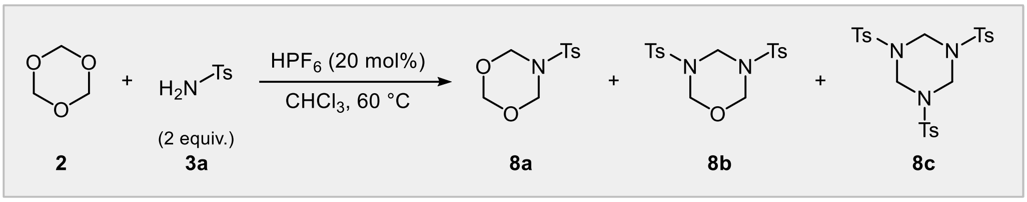


<u>*Procedure:*</u> **2a** (0.03 mmol) and **3a** (0.06 mmol, 2 equiv.) were added into a J-Young NMR tube, followed by adding 0.5 mL $CDCl_3$ and $HPF_6$ (20 mol%). The reaction mixture was analyzed at different times in a 300 MHz NMR spectrometer at 60 °C.

The reaction of *sym*-trioxane and *p*-toluenesulfonamide in the presence of catalytic amounts of $HPF_6$ led to the formation of the previously observed condensation products **8a–c** (see Figure S-2).

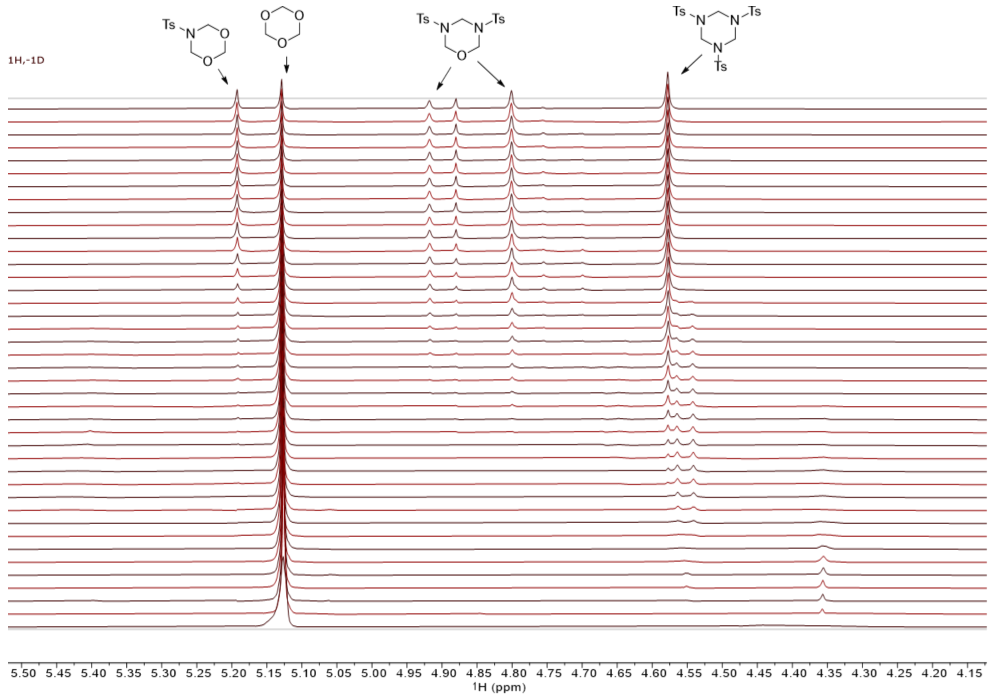


*Figure S-3. $^1$H NMR monitoring of the $HPF_6$ catalyzed condensation of sym-trioxane and p-toluenesulfonamide.*

Considering the known dynamic nature of the sulfonamide-formaldehyde condensation,[12-14] we wanted to determine if such a scenario could also take place in our system. This means, if the formed condensation products can also interconvert under the presence of formaldehyde and sulfonamide. To do so, we conducted the next two experiments in 0.1 mmol scale (in $CDCl_3$ at 60 °C), analyzing aliquots of the reaction mixtures at certain times by $^1$H NMR.

**Reaction between 2 and 8c:**

The reaction of **8c** (1,3,5-tritosyl-1,3,5-triazinane) with *sym*-trioxane was studied both without and with catalytic amounts of $HPF_6$. As can be seen on Figure S-4, the conversion of **8c** into dioxazinane **8a** could only take place under strong acid catalysis.

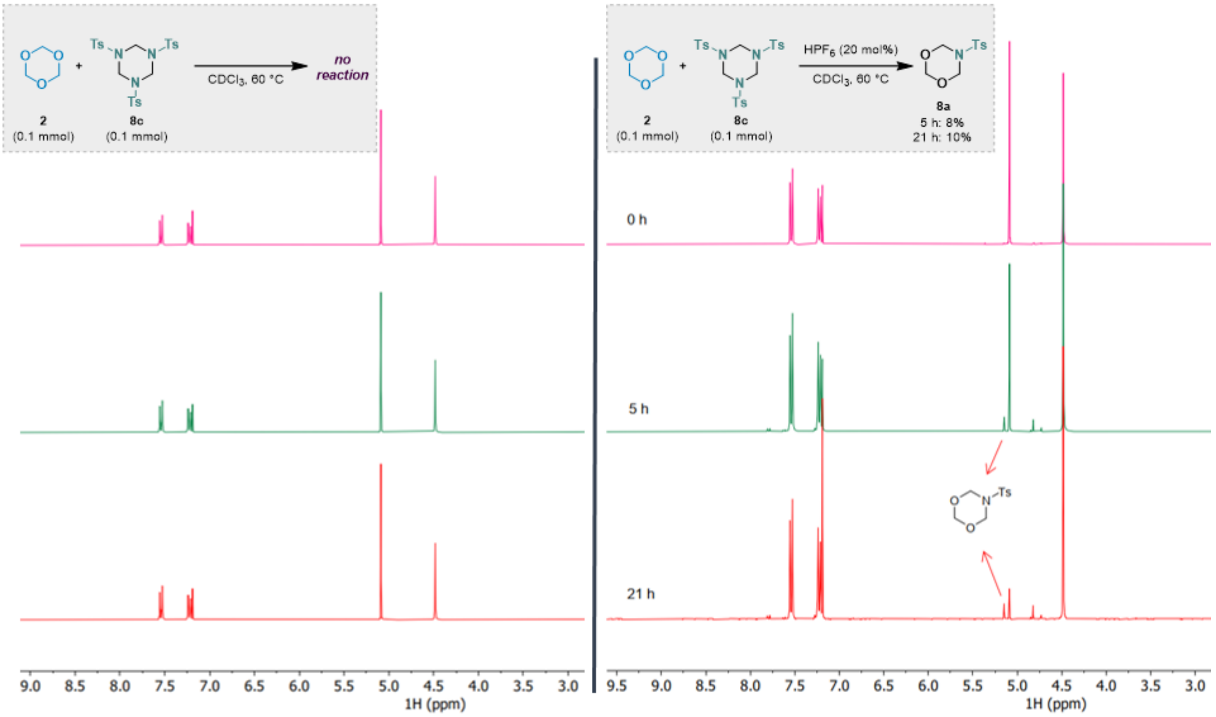


*Figure S-4. $^{1}$H NMR monitoring of the reaction between **8c** and sym-trioxane, without and with $HPF_6$ as catalyst.*

**Reaction between 2 and 8b:**

Similarly, oxadiazinane **8b** reacted with *sym*-trioxane in the presence of $HPF_6$ to produce **8a**, as observed in the $^{1}$H NMR monitoring presented in Figure S-5.

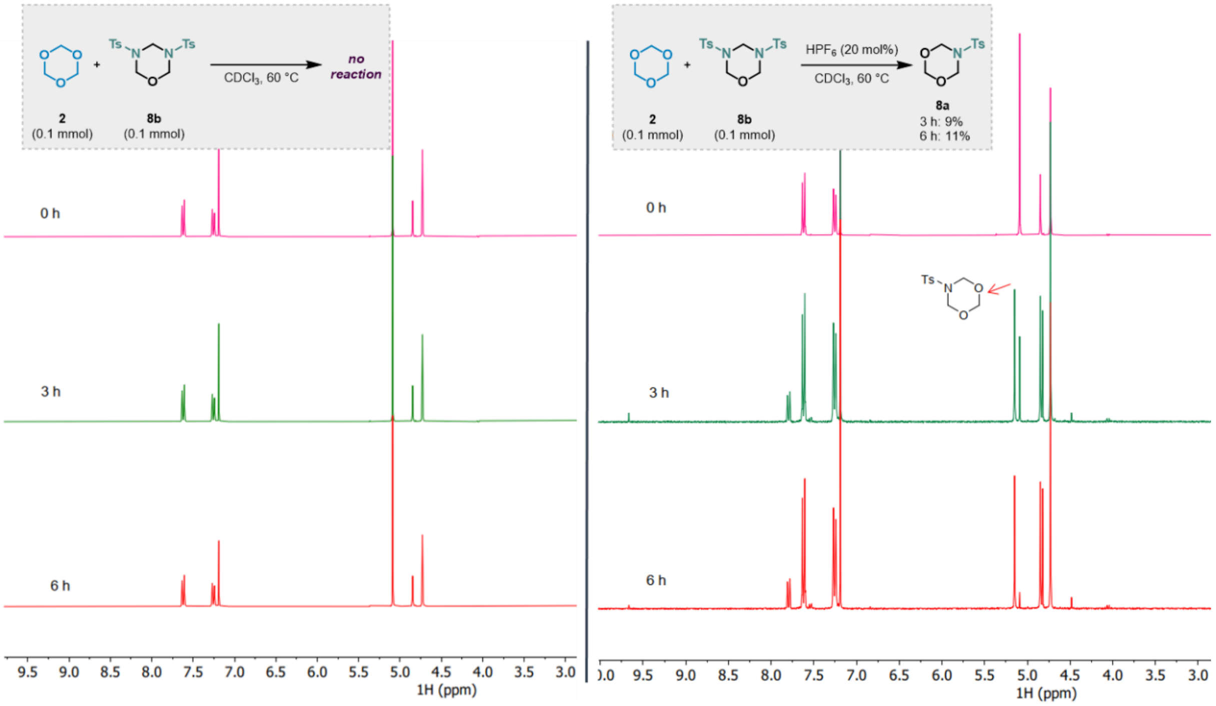


*Figure S-5. $^{1}$H NMR monitoring of the reaction between **8b** and sym-trioxane, without and with $HPF_6$ as catalyst.*

## 7.3. Stereospecificity: Experiments with β-Deuterostyrenes

Two possible mechanisms for the acid catalyzed oxy-aminomethylation of olefins can be considered: either a stepwise reaction pathway (involving the intermediacy of a benzyl cation), or a (pseudo)-concerted pathway.

We considered studying the three-component reaction using β-deuterium-labeled styrenes (**d-1a-*cis*** and **d-1a-*trans***, respectively) as substrates. As shown on the $^1$H NMR spectra (see Figure S-6), the $HPF_6$ catalyzed reaction of these β-deuterostyrenes led in both cases to *cis/trans* mixtures of the corresponding 1,3-oxazinane. This result points toward the intermediacy of a benzylic cation in the reaction mechanism.[3]

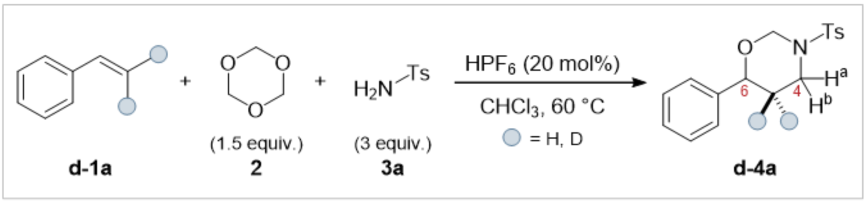


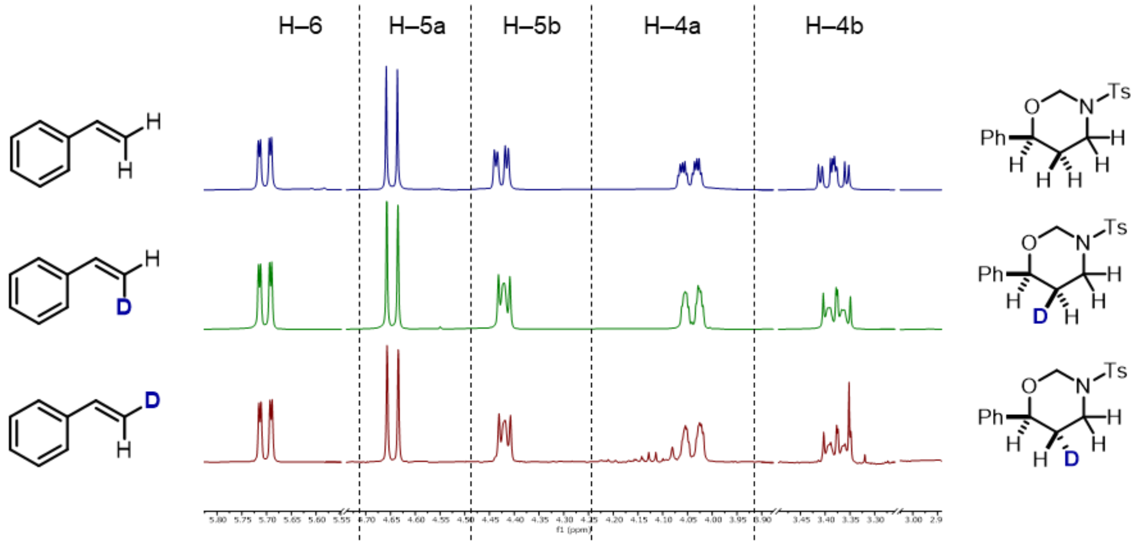


*Figure S-6. $^1$H NMR analysis of the oxy-aminomethylation reaction of β-deuterostyrenes (**d-1a**) under $HPF_6$ catalysis.*

# 8. Copies of NMR Spectra

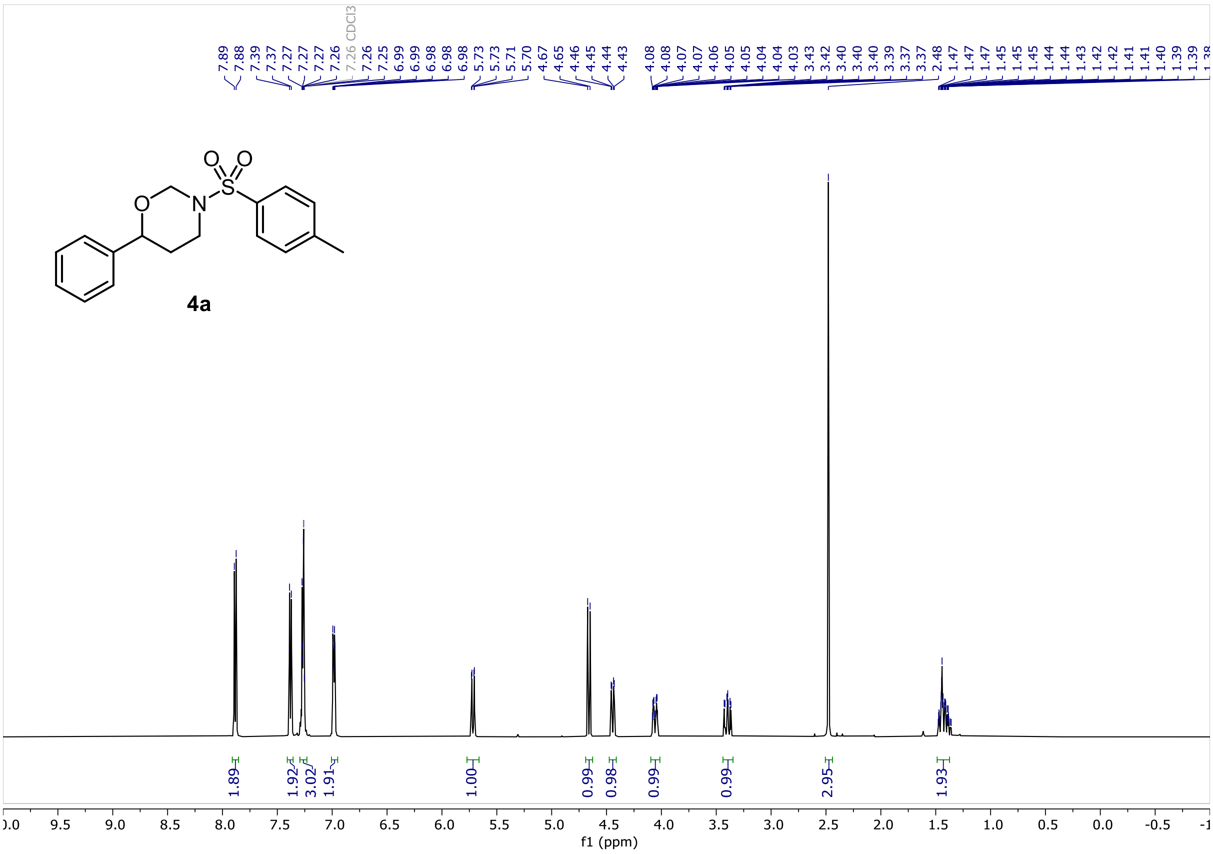


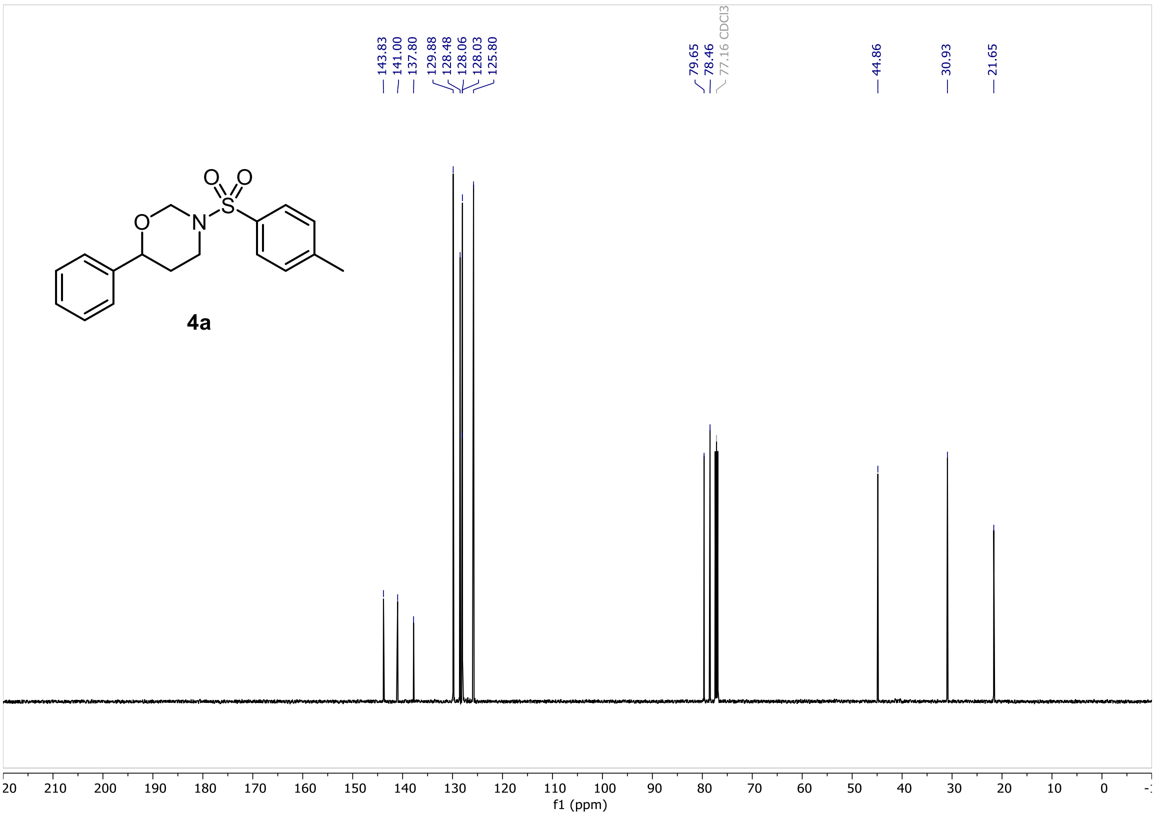


NMR spectra for compound **4a**: $^{1}H$ (501 MHz) and $^{13}C$ (126 MHz), in $CDCl_3$.

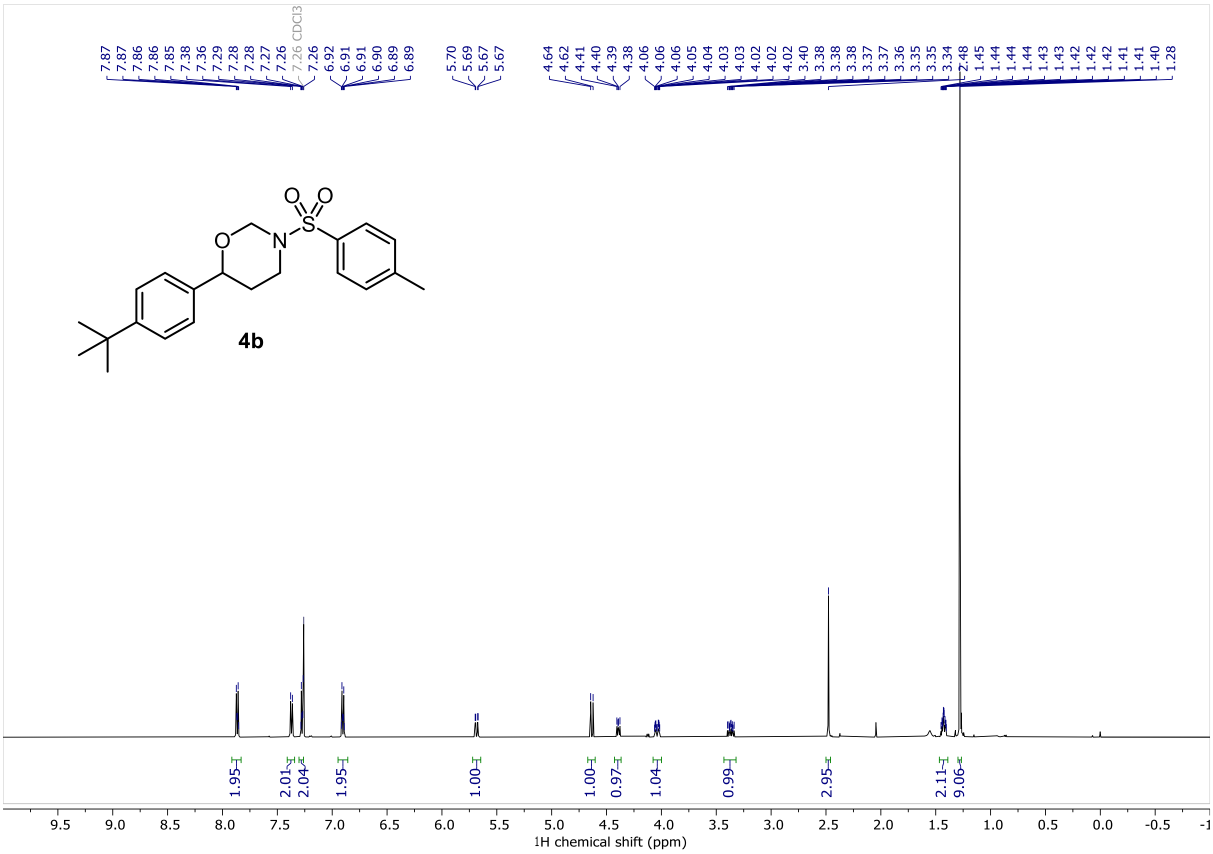

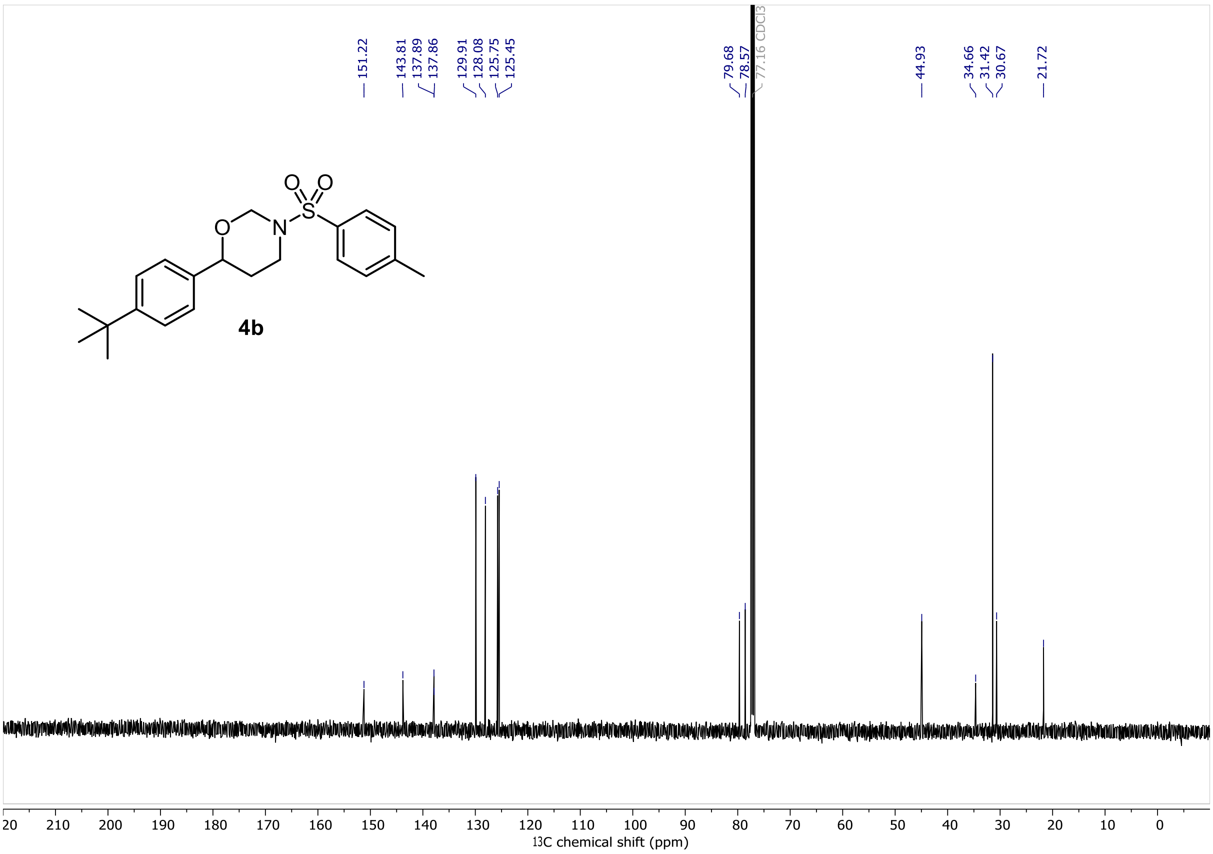

NMR spectra for compound **4b**: $^{1}$H (501 MHz) and $^{13}$C (126 MHz), in $CDCl_3$.

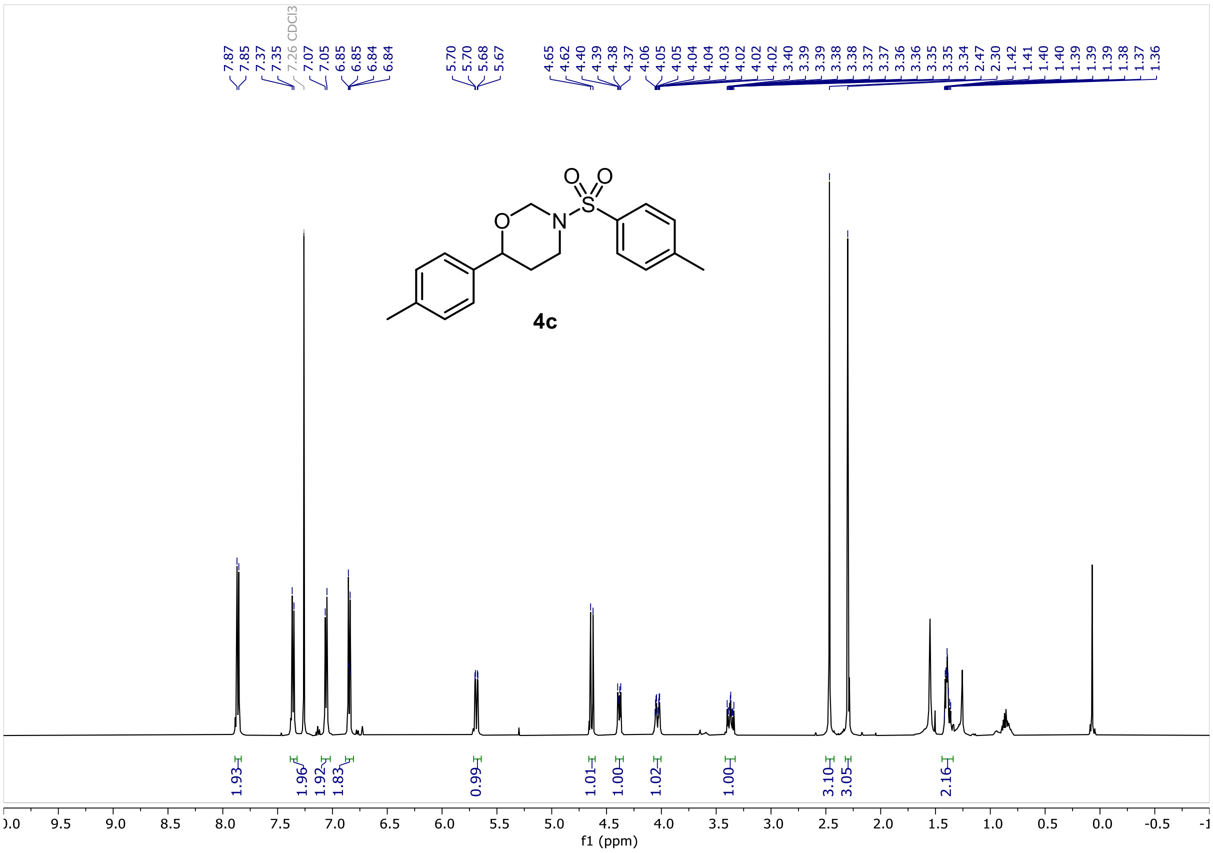

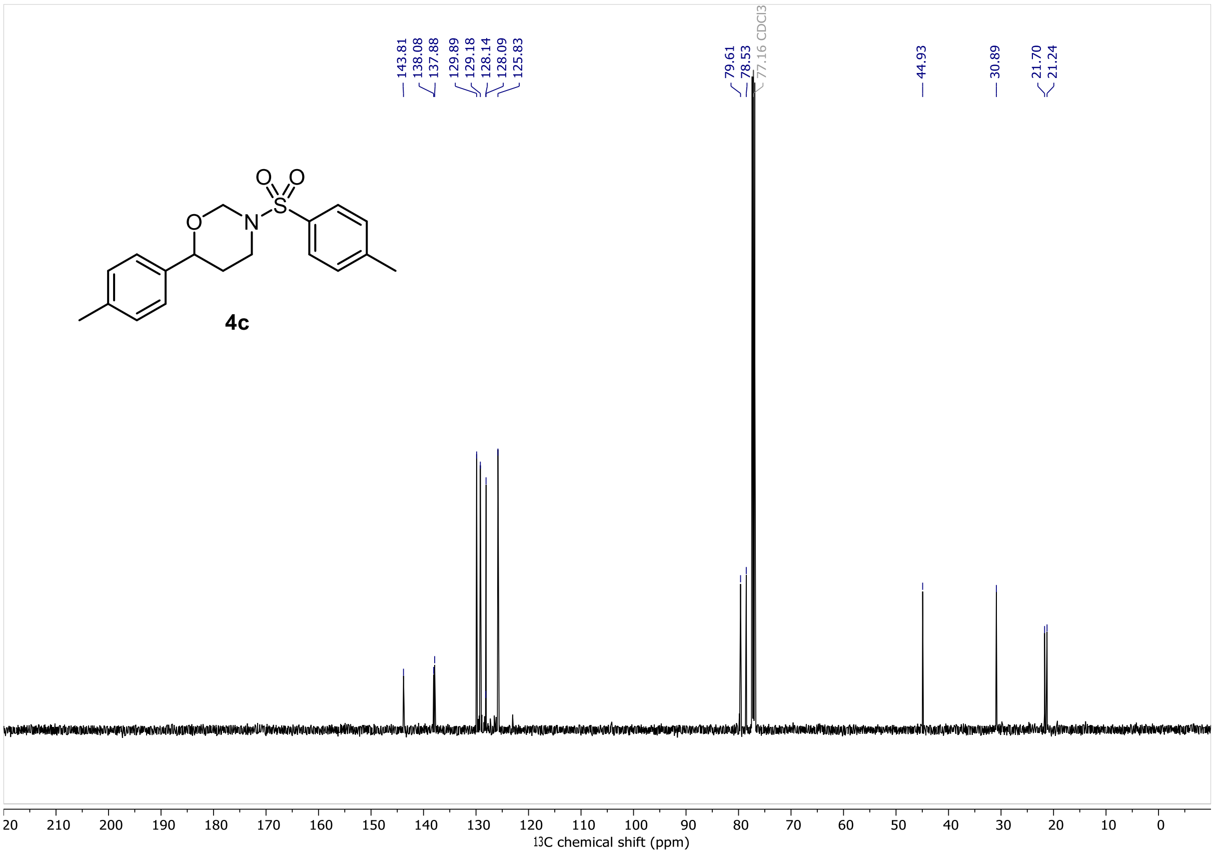

NMR spectra for compound **4c**: $^{1}$H (501 MHz) and $^{13}$C (126 MHz), in $CDCl_3$.

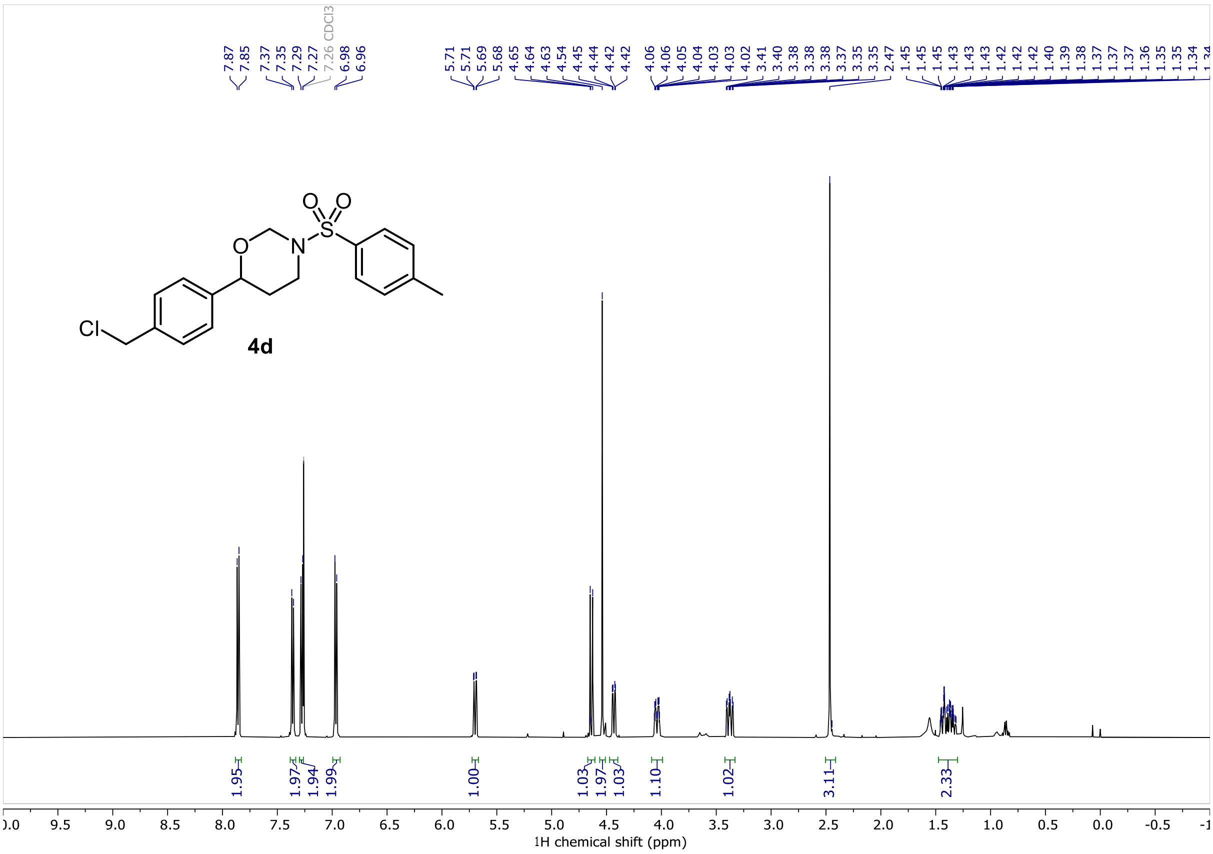


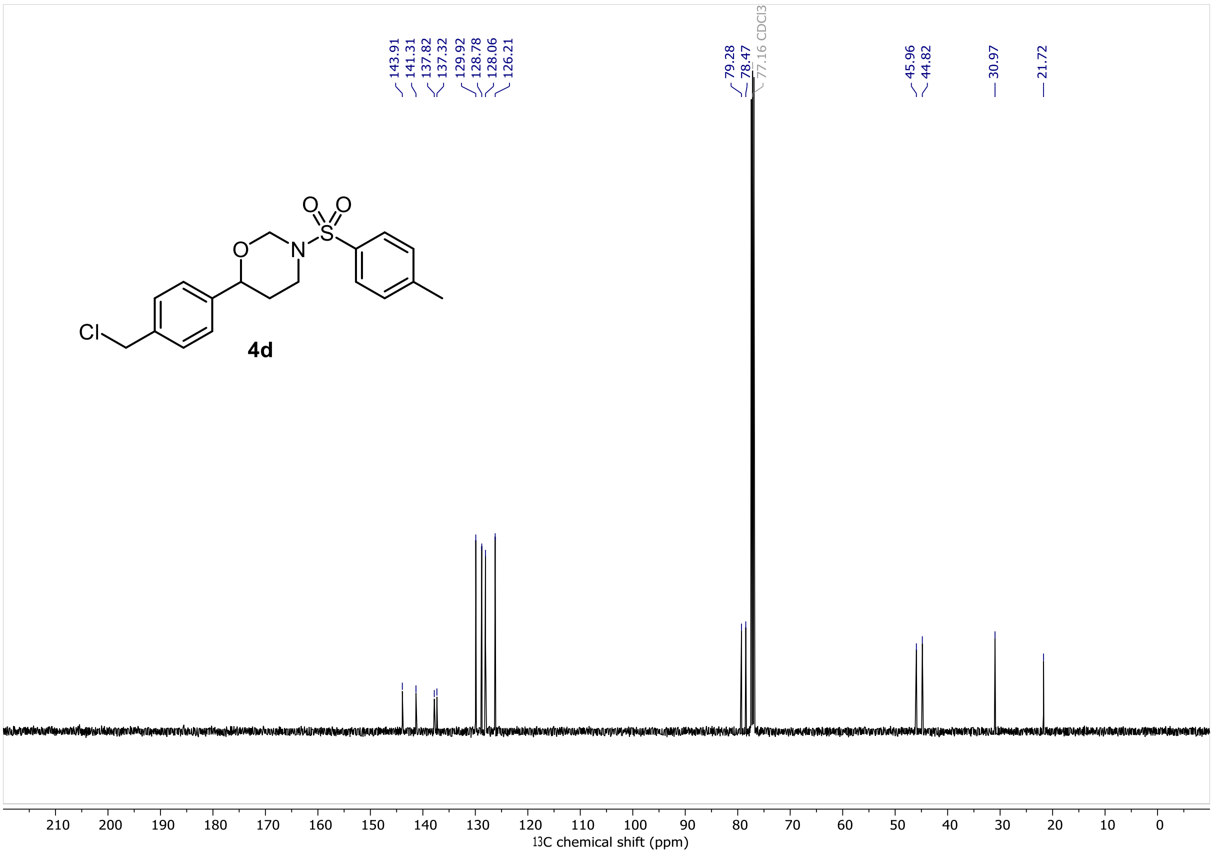


NMR spectra for compound **4d**: $^{1}H$ (501 MHz) and $^{13}C$ (126 MHz), in $CDCl_3$.

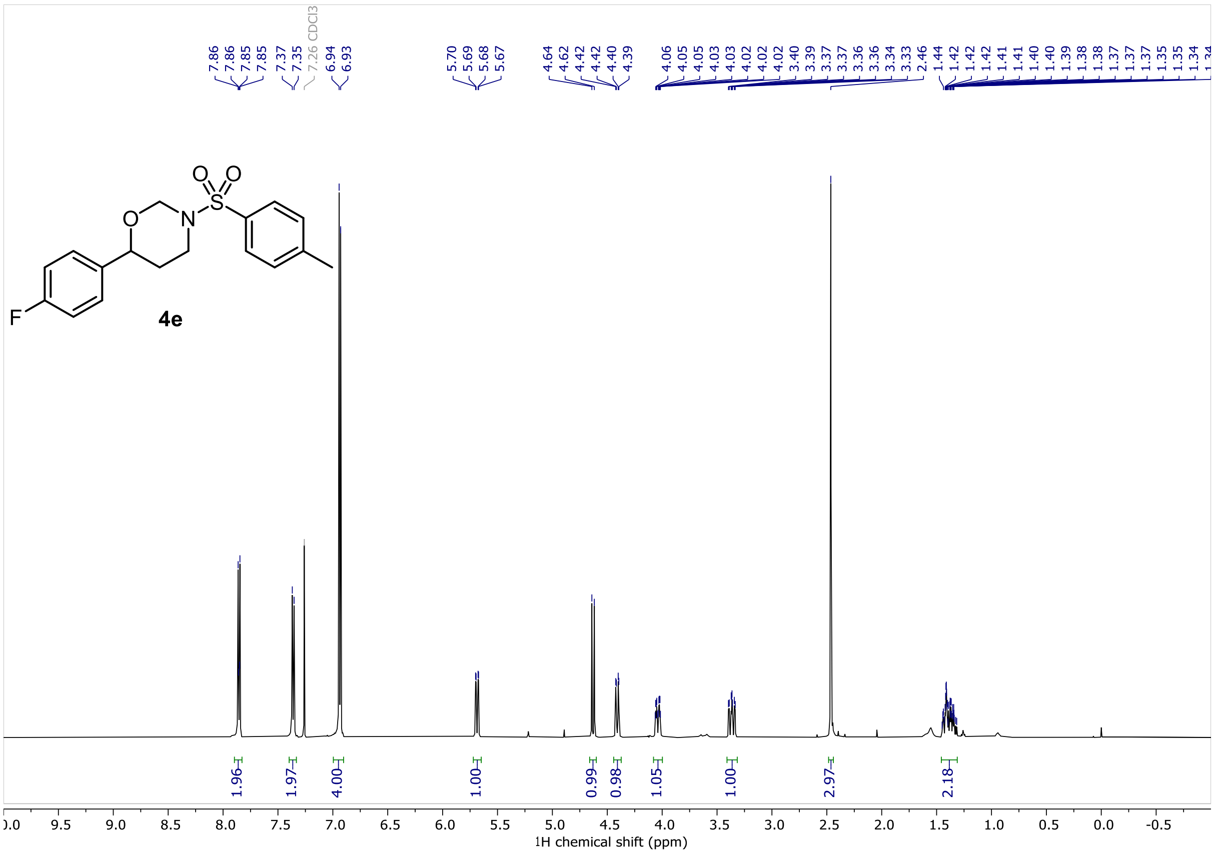

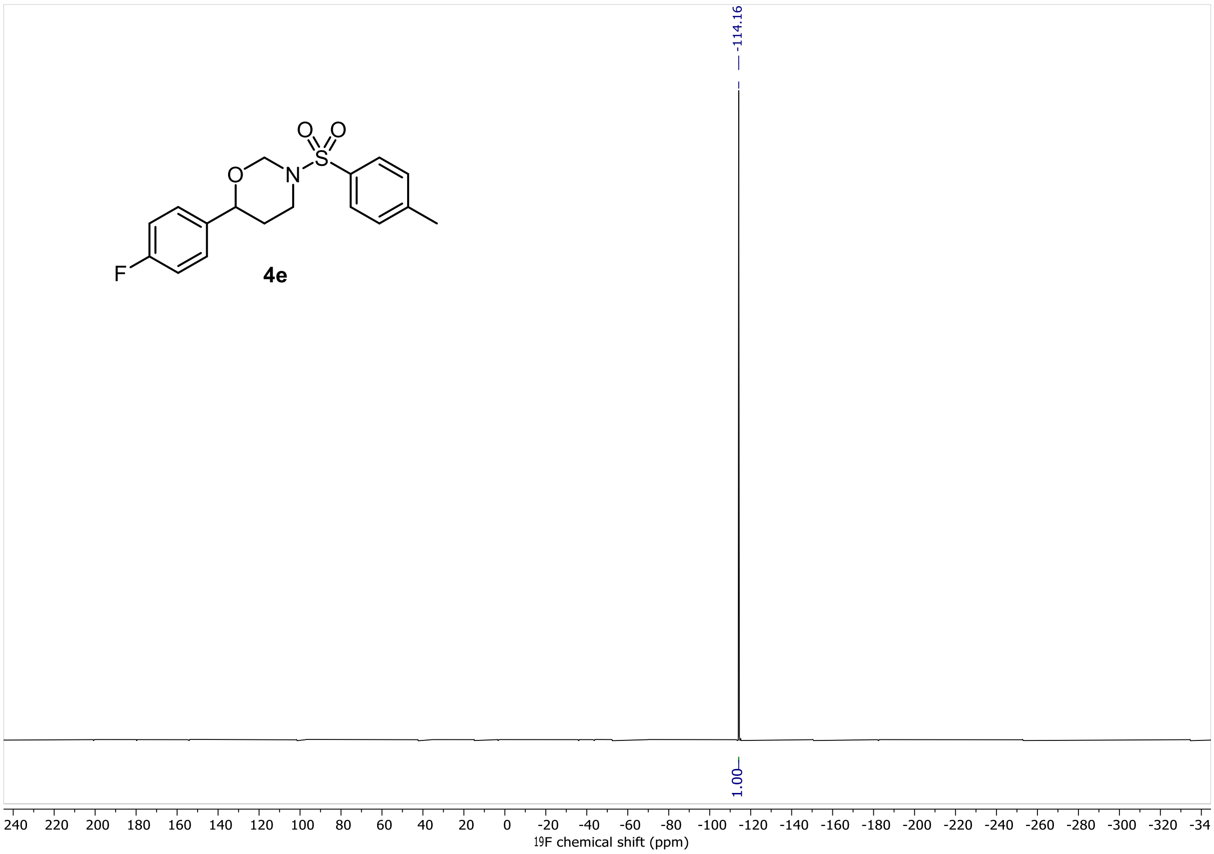

NMR spectra for compound **4e**: $^{1}H$ (501 MHz) and $^{19}F$ (471 MHz), in $CDCl_3$.

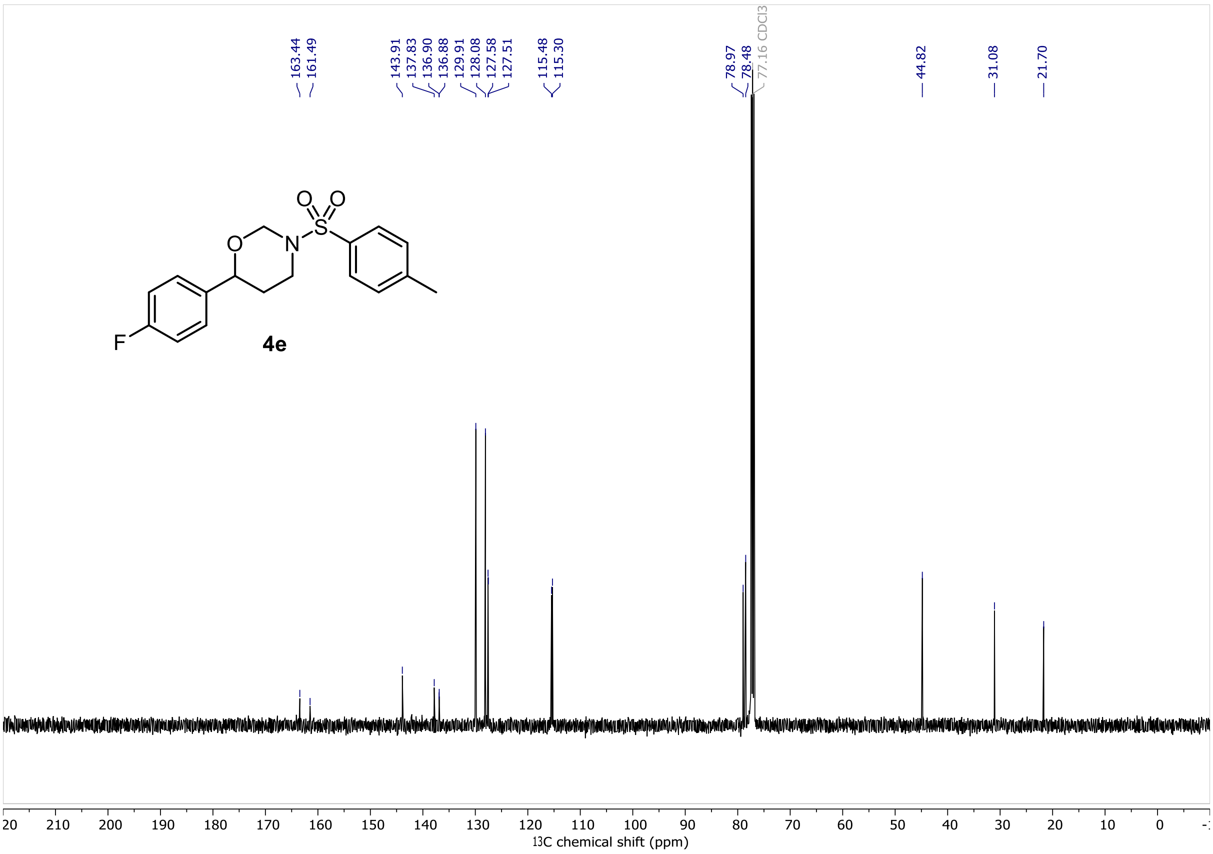


NMR spectra for compound **4e** *(continuation)*: $^{13}$C (126 MHz), in $CDCl_3$.

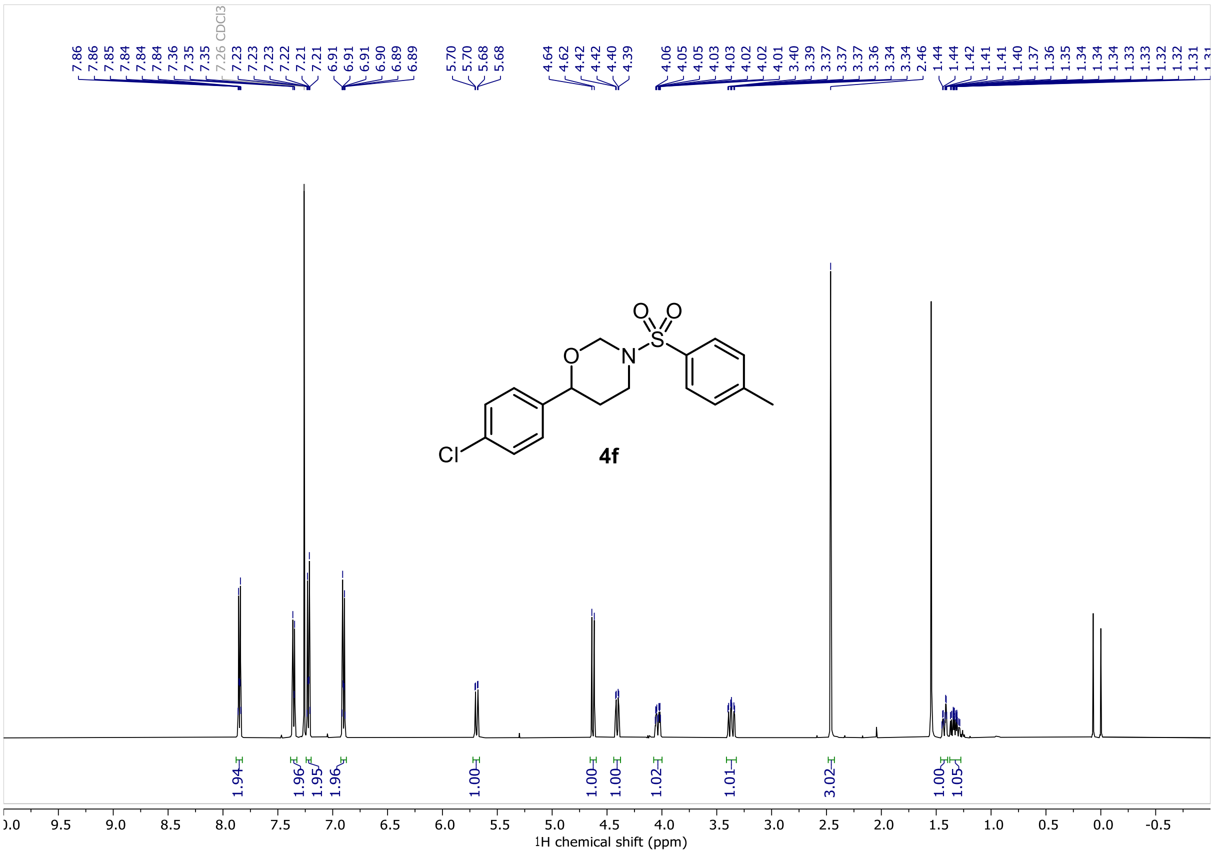


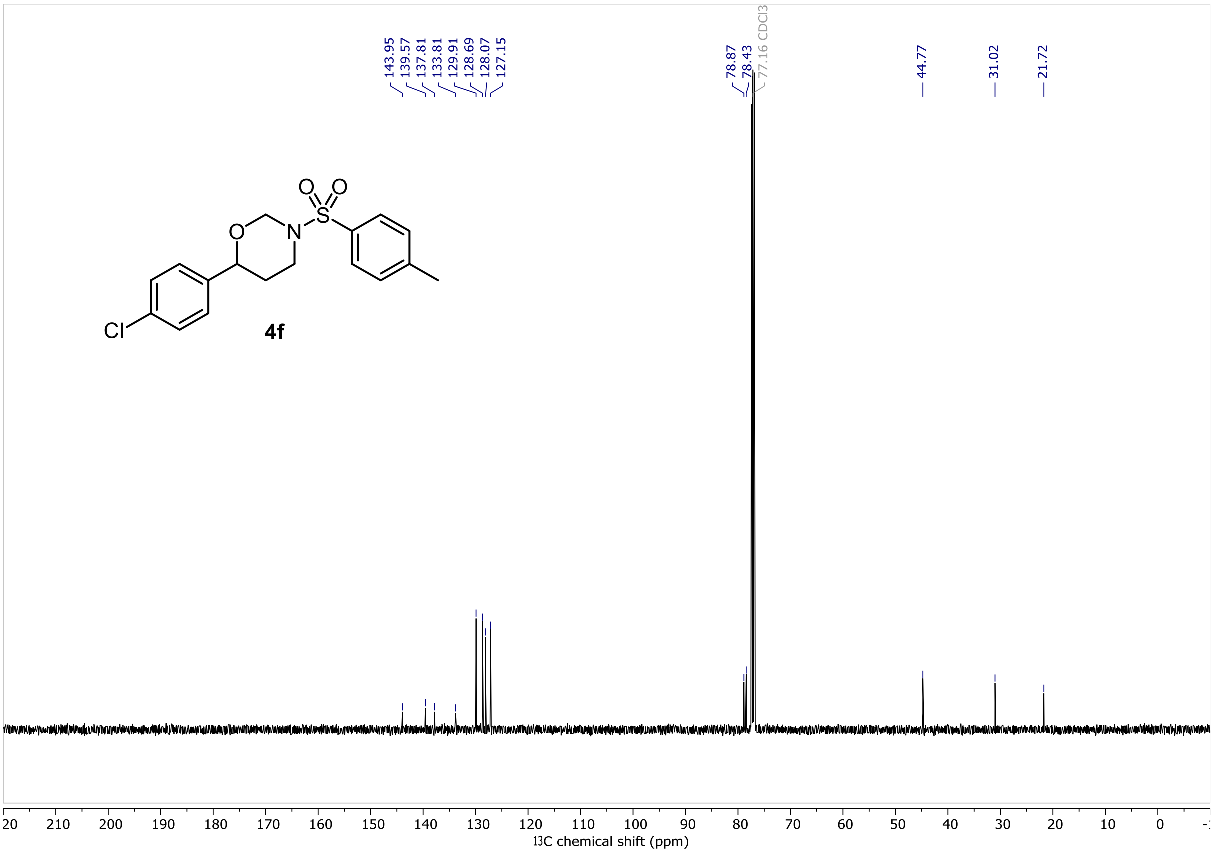


NMR spectra for compound **4f**: $^{1}$H (501 MHz) and $^{13}$C (126 MHz), in $CDCl_3$.

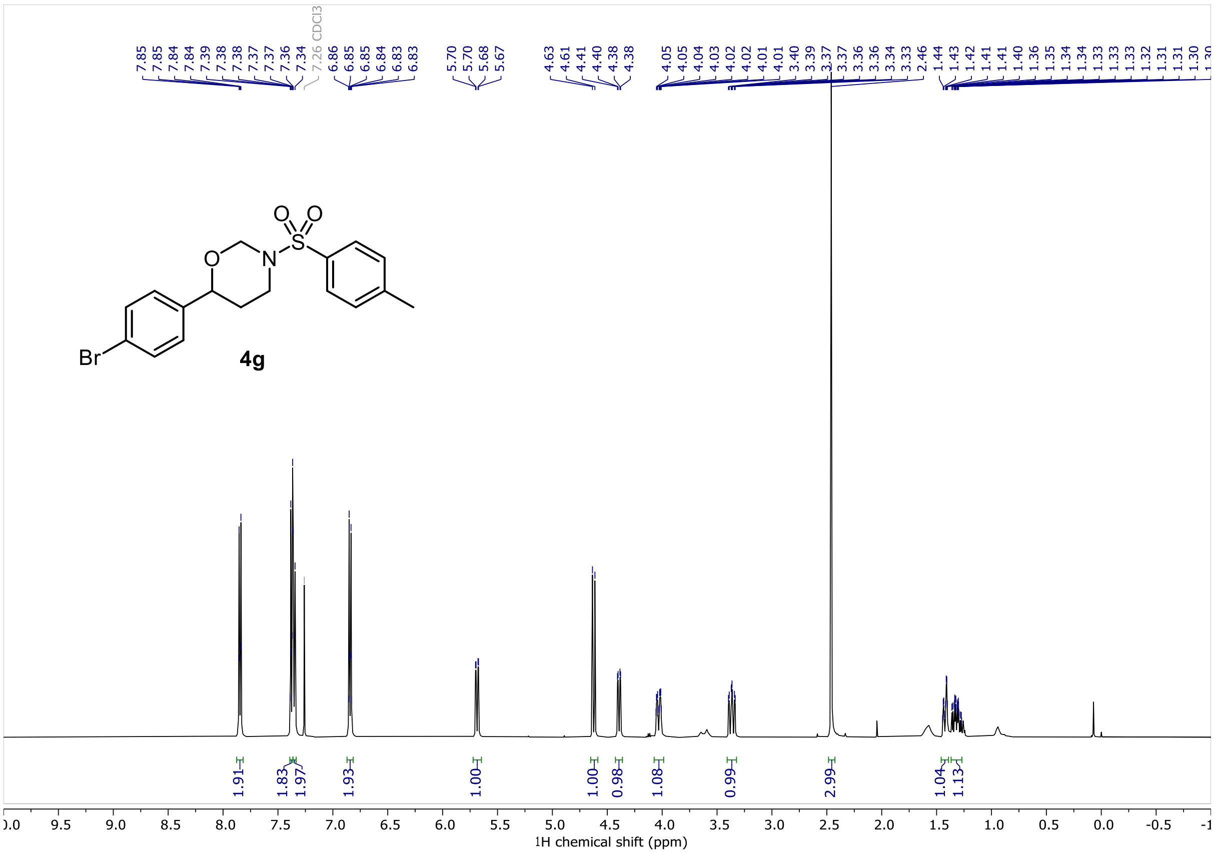

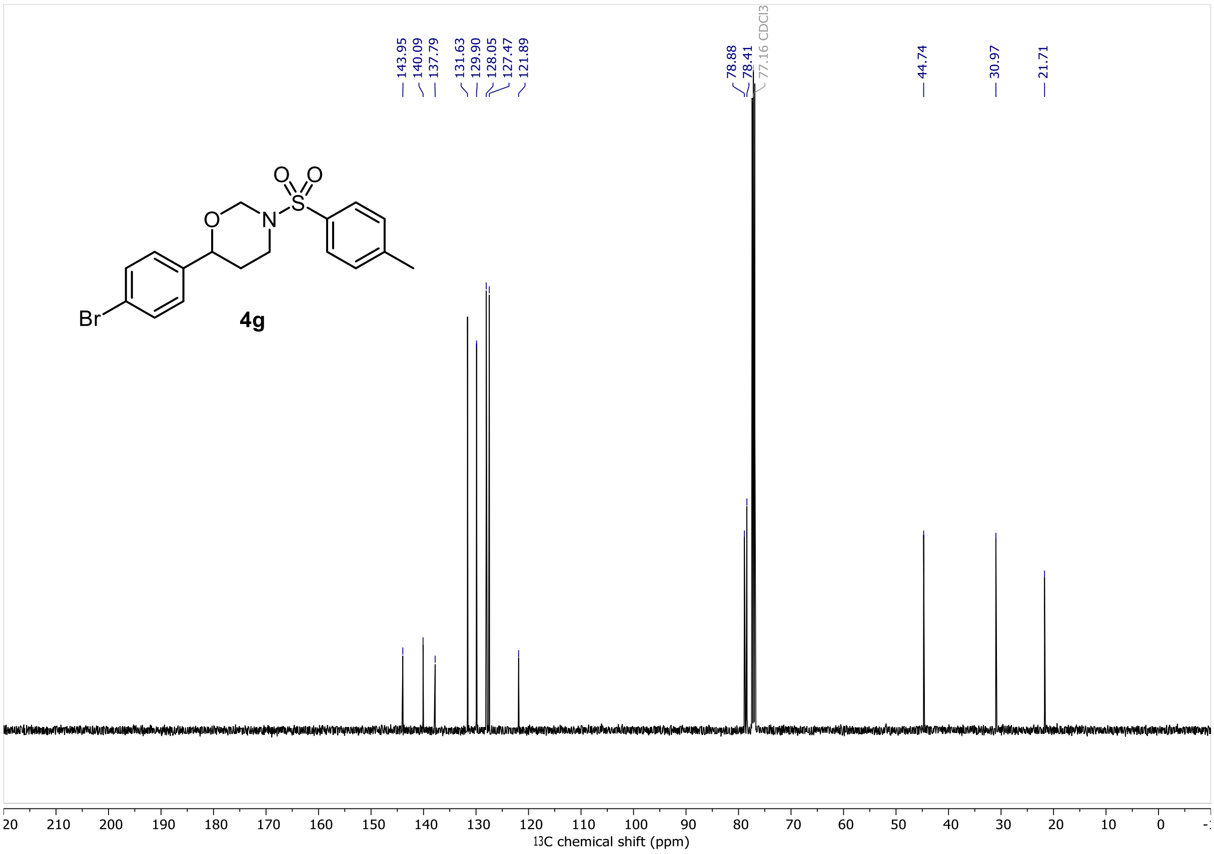

NMR spectra for compound **4g**: $^{1}$H (501 MHz) and $^{13}$C (126 MHz), in $CDCl_3$.

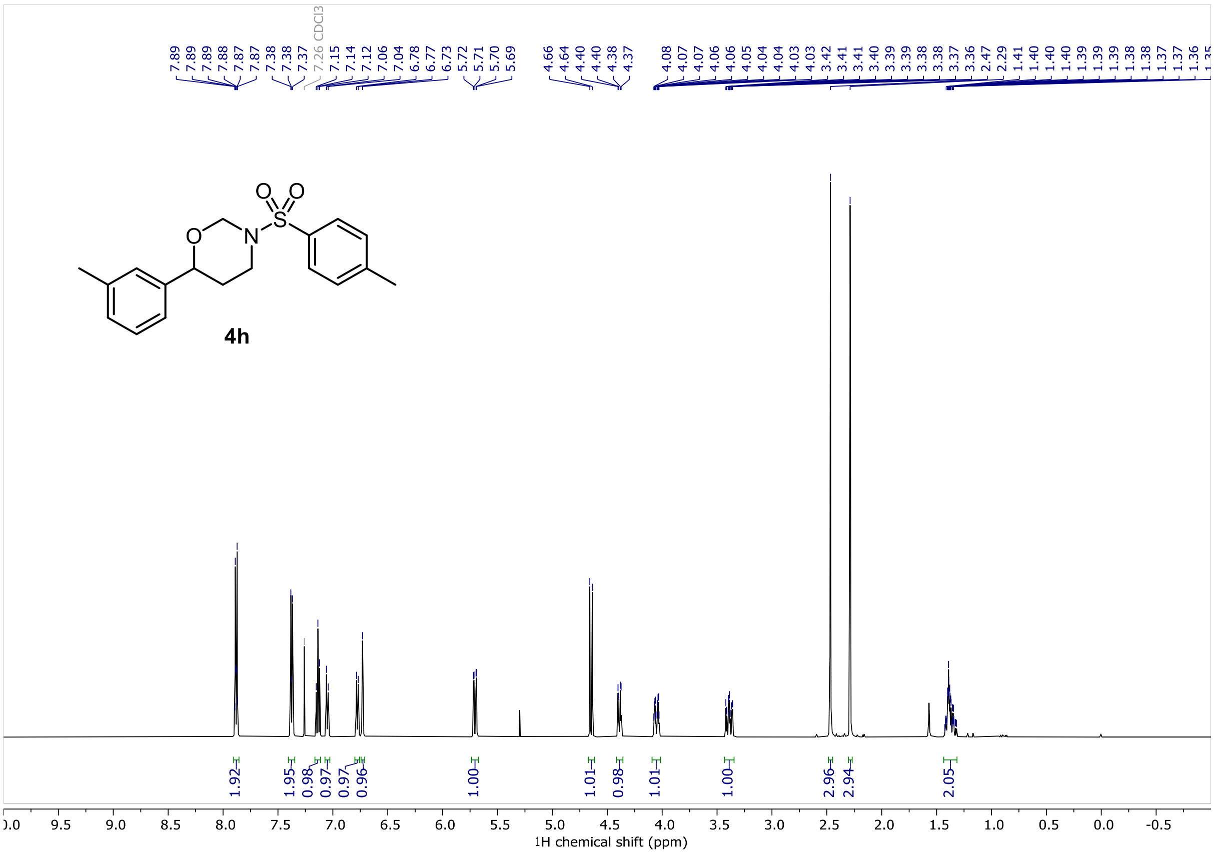


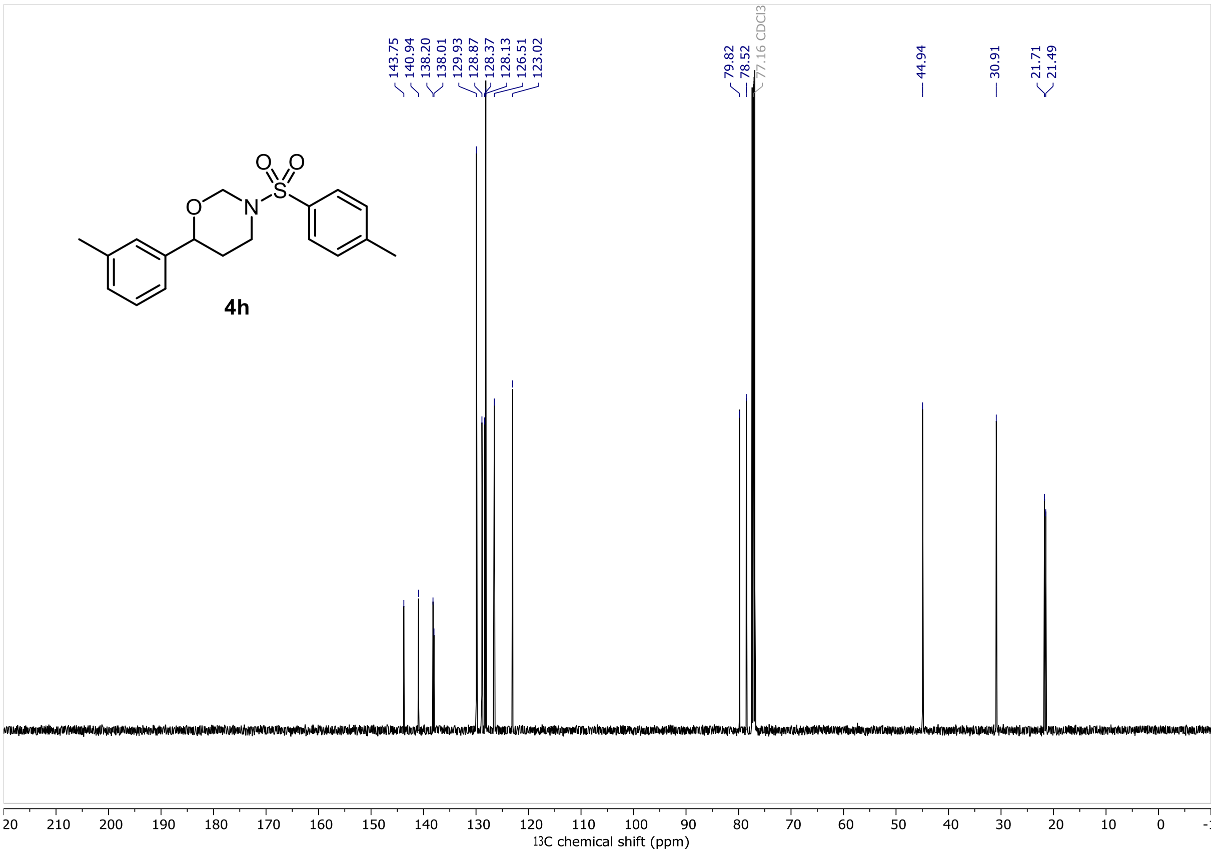


NMR spectra for compound **4h**: $^{1}$H (501 MHz) and $^{13}$C (126 MHz), in $CDCl_3$.

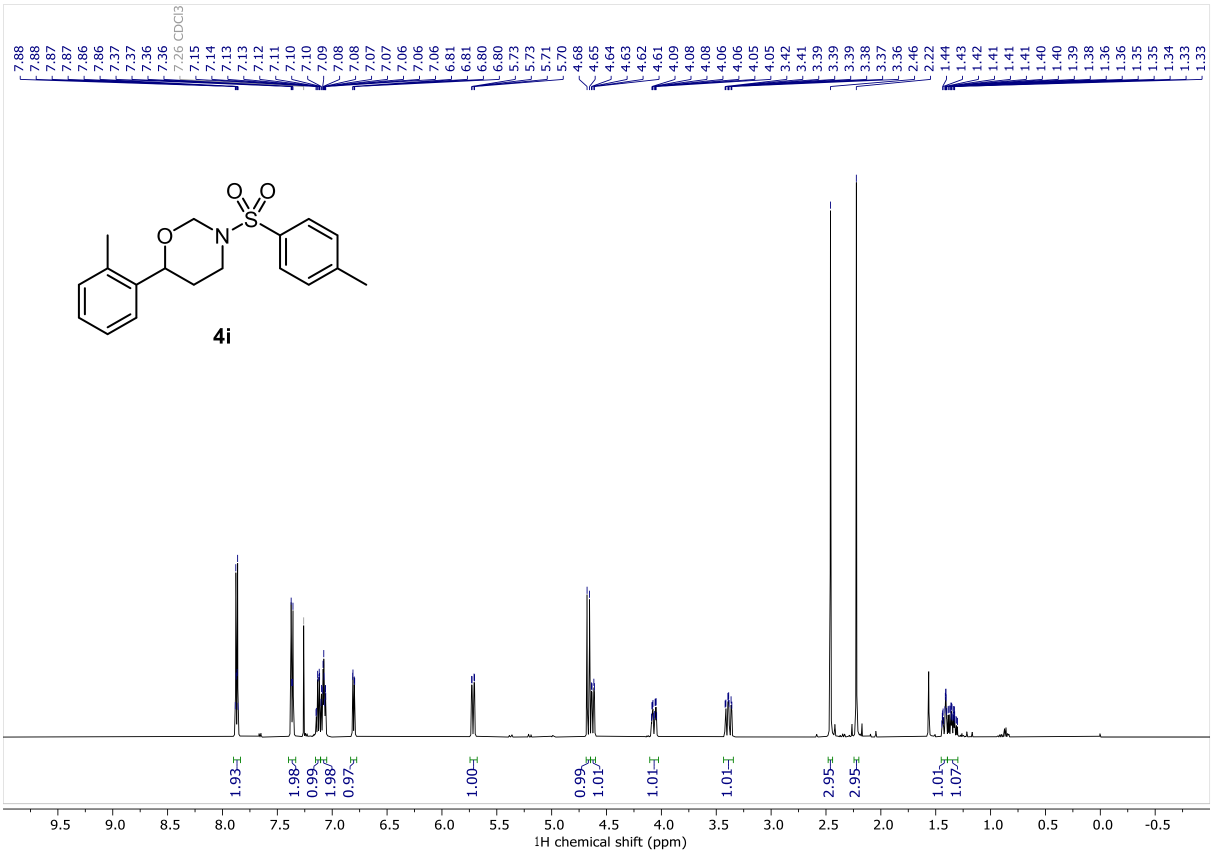


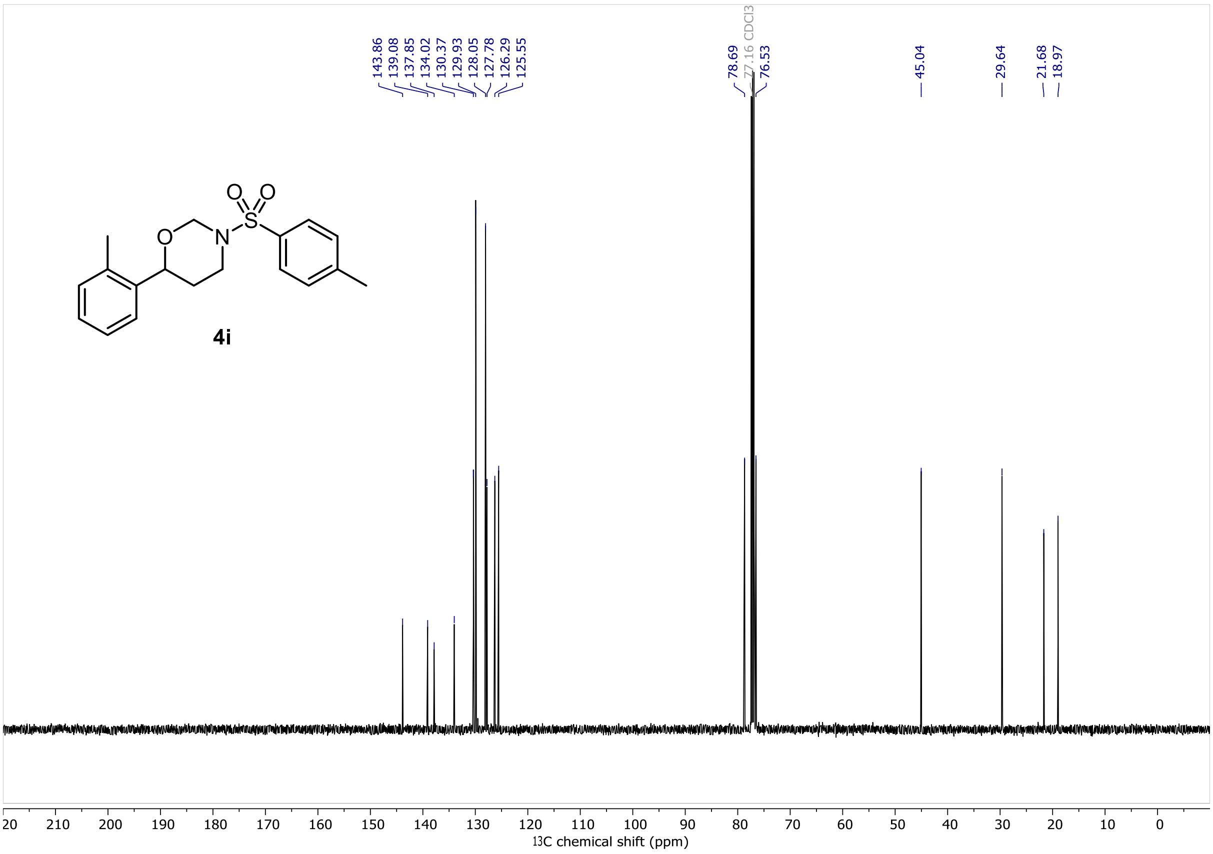


NMR spectra for compound **4i**: $^{1}H$ (501 MHz) and $^{13}C$ (126 MHz), in $CDCl_3$.

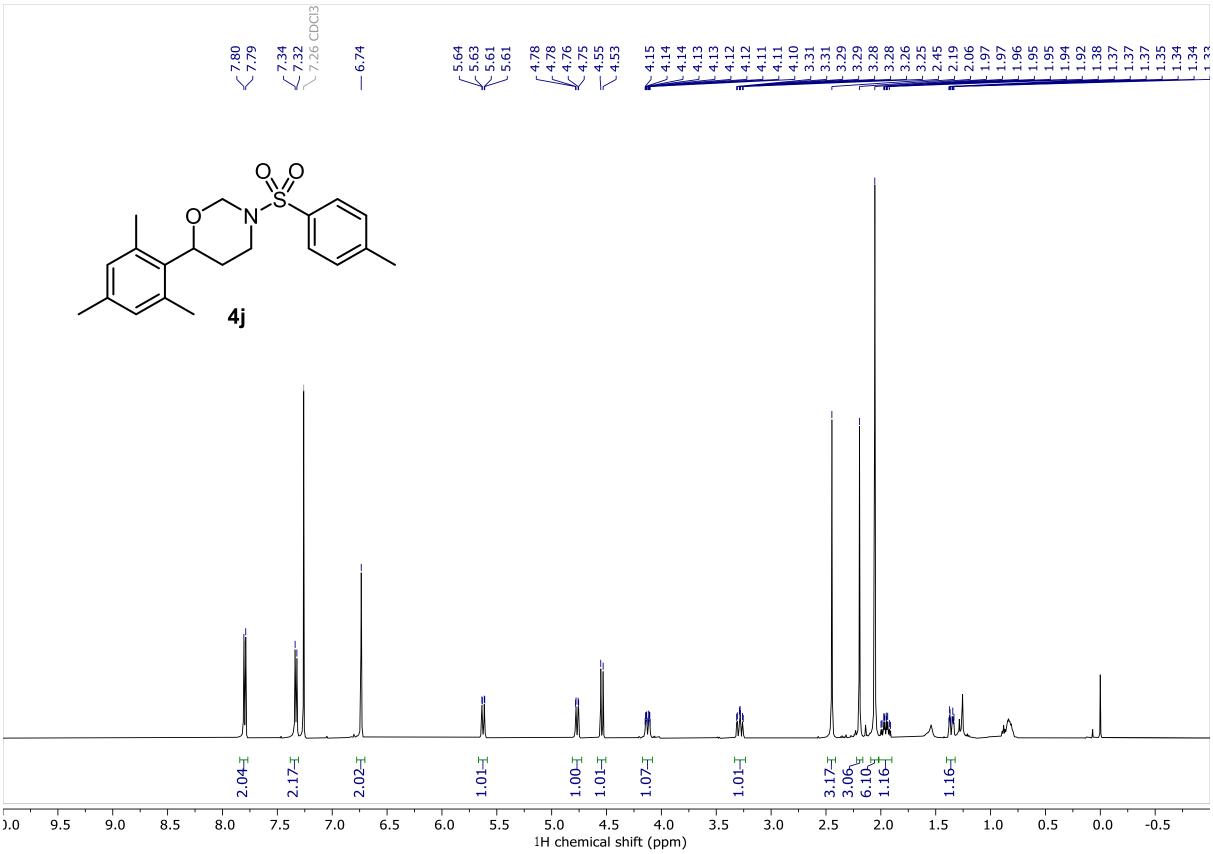


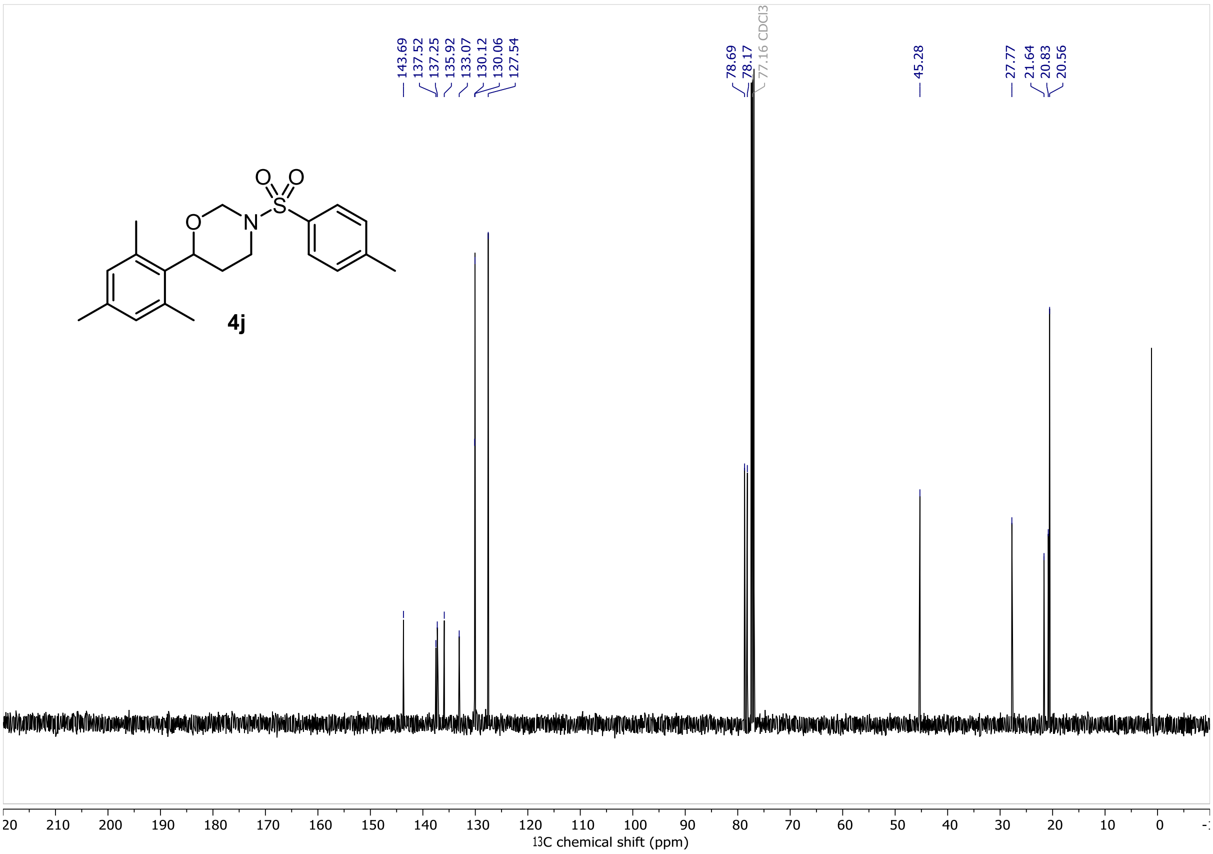


NMR spectra for compound **4j**: $^{1}H$ (501 MHz) and $^{13}C$ (126 MHz), in $CDCl_3$.

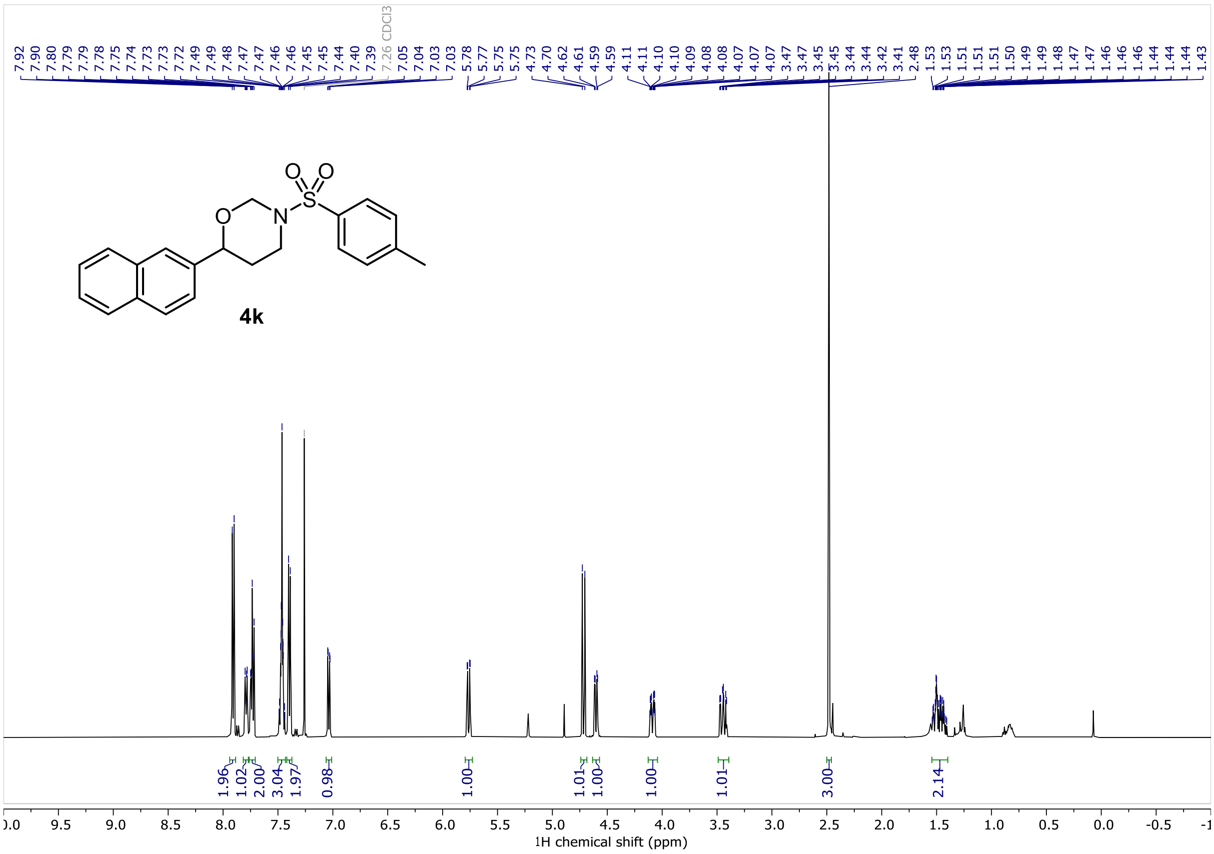

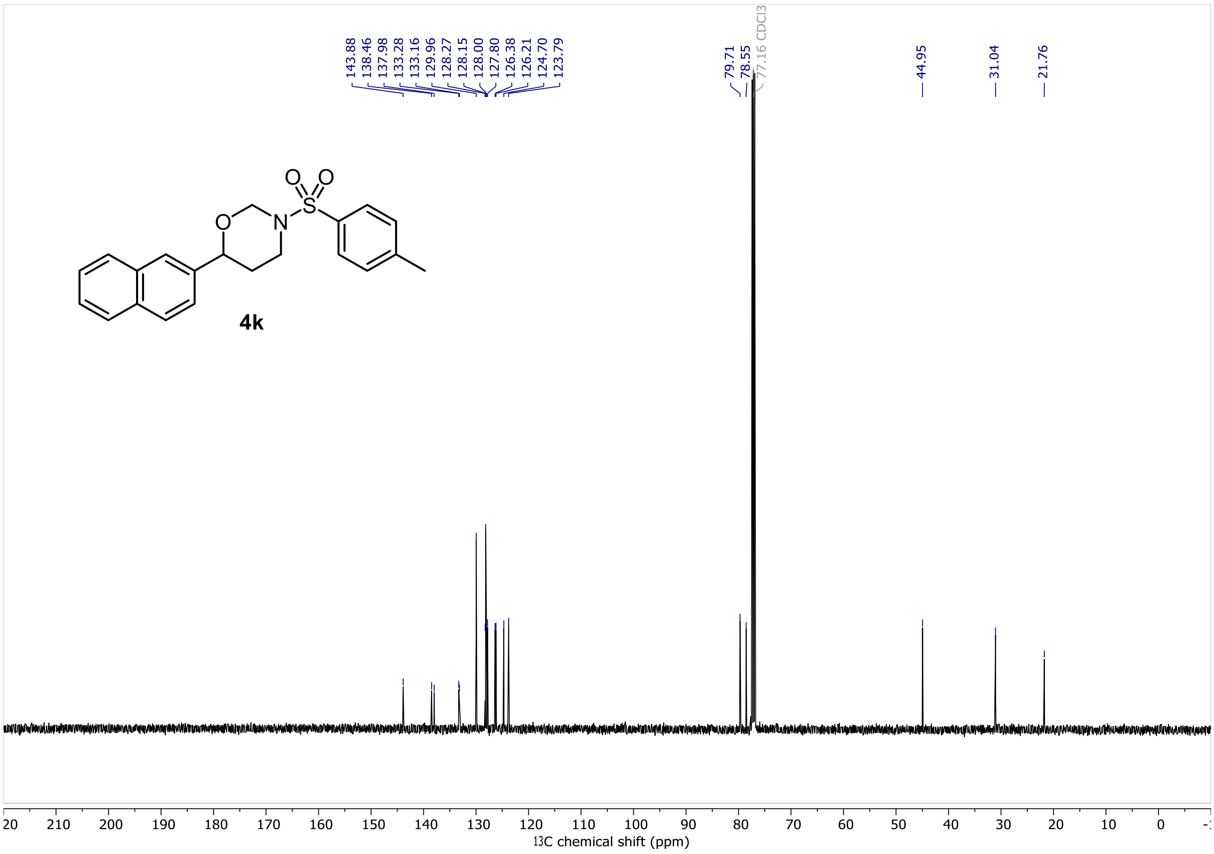

NMR spectra for compound **4k**: $^{1}$H (501 MHz) and $^{13}$C (126 MHz), in $CDCl_3$.

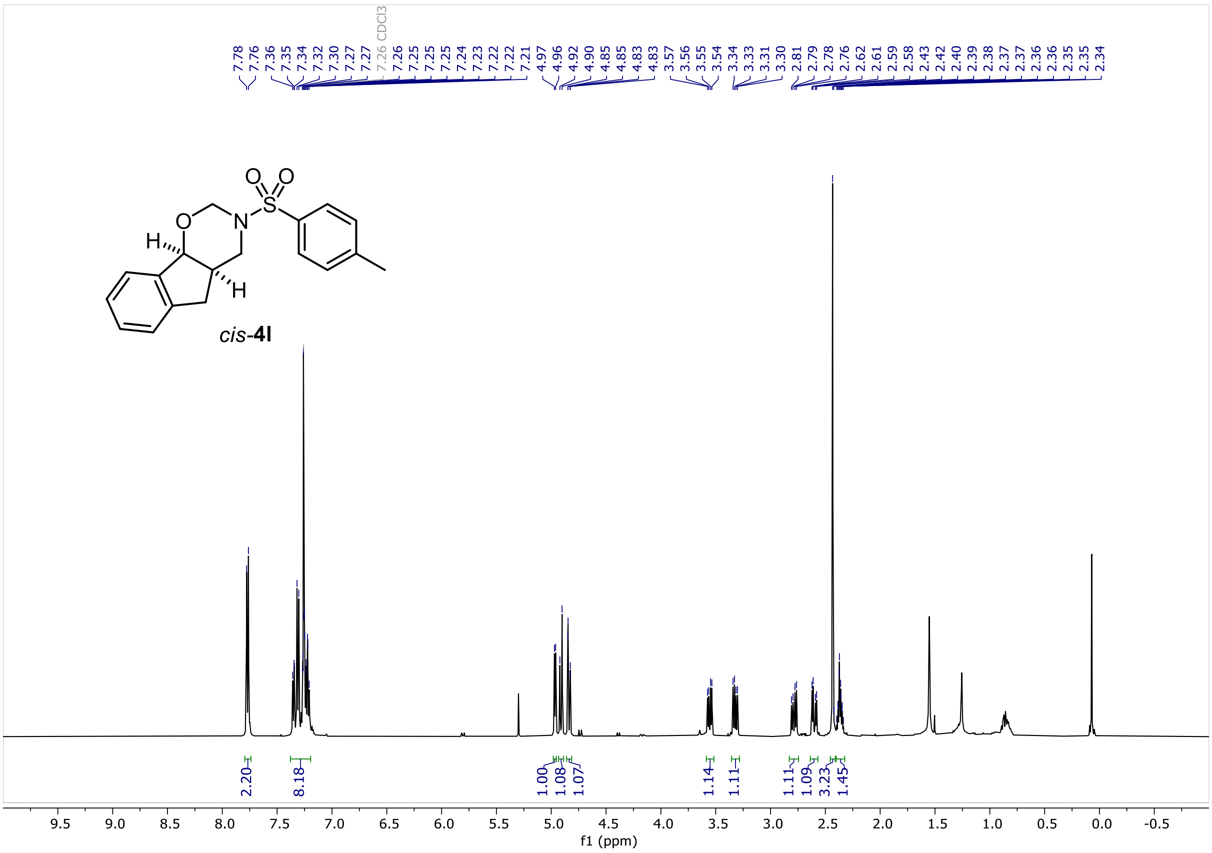


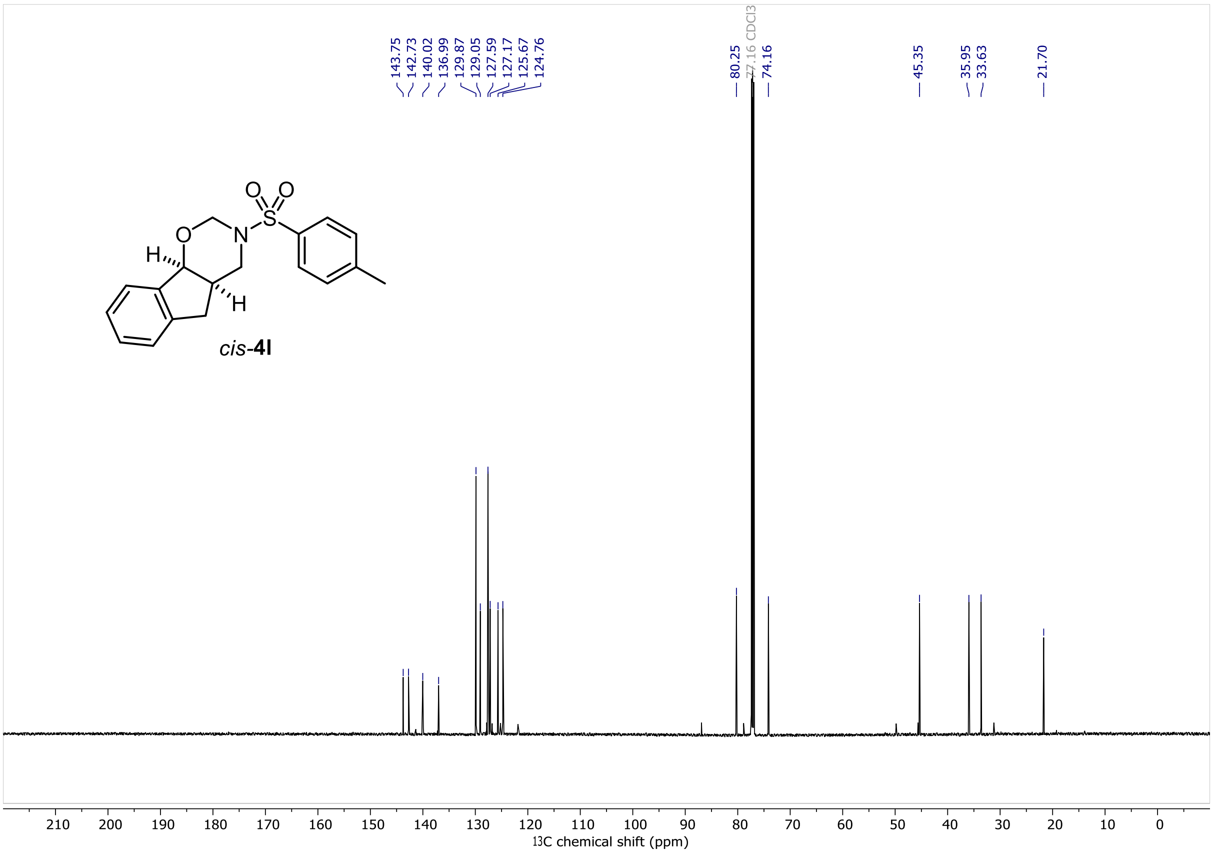


NMR spectra for compound *cis*-**4l**: $^{1}$H (600 MHz) and $^{13}$C (151 MHz), in $CDCl_3$.

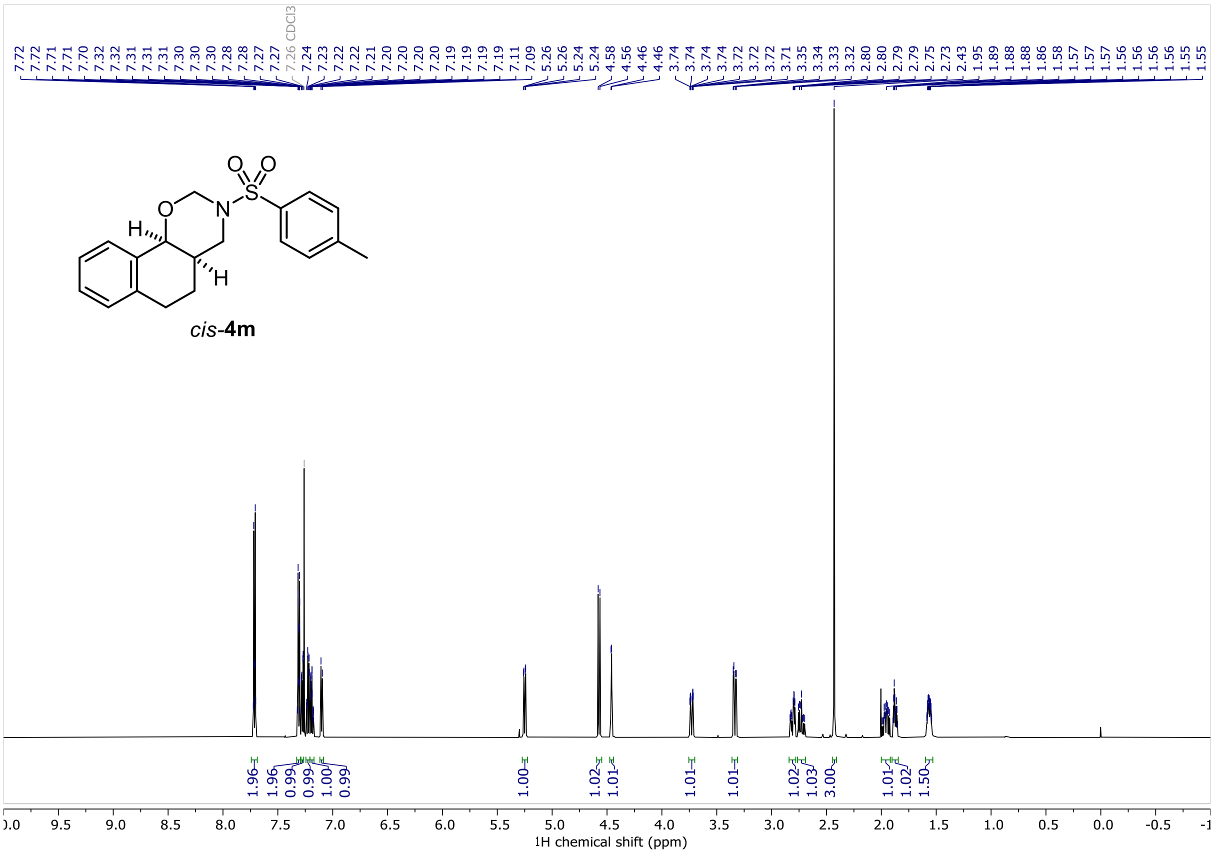

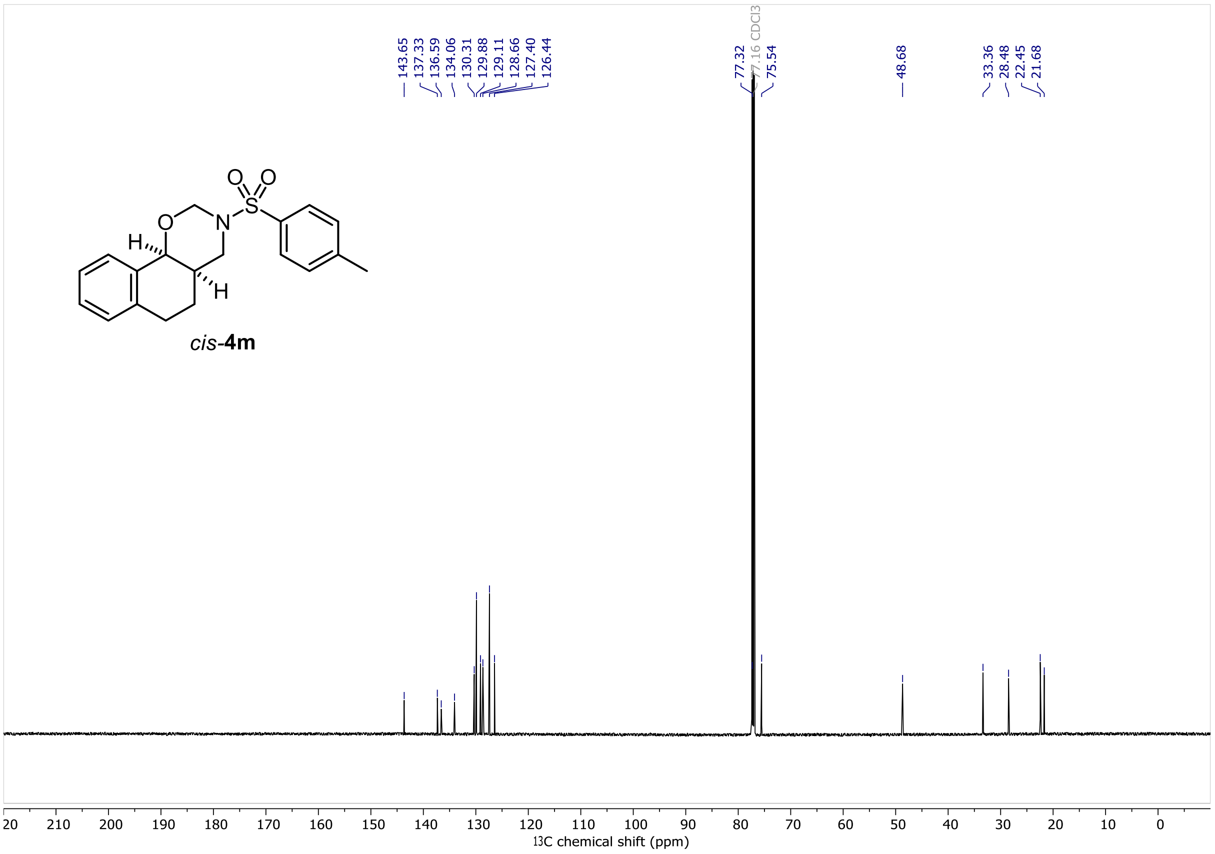

NMR spectra for compound *cis*-**4m**: $^{1}$H (600 MHz) and $^{13}$C (151 MHz), in $CDCl_3$.

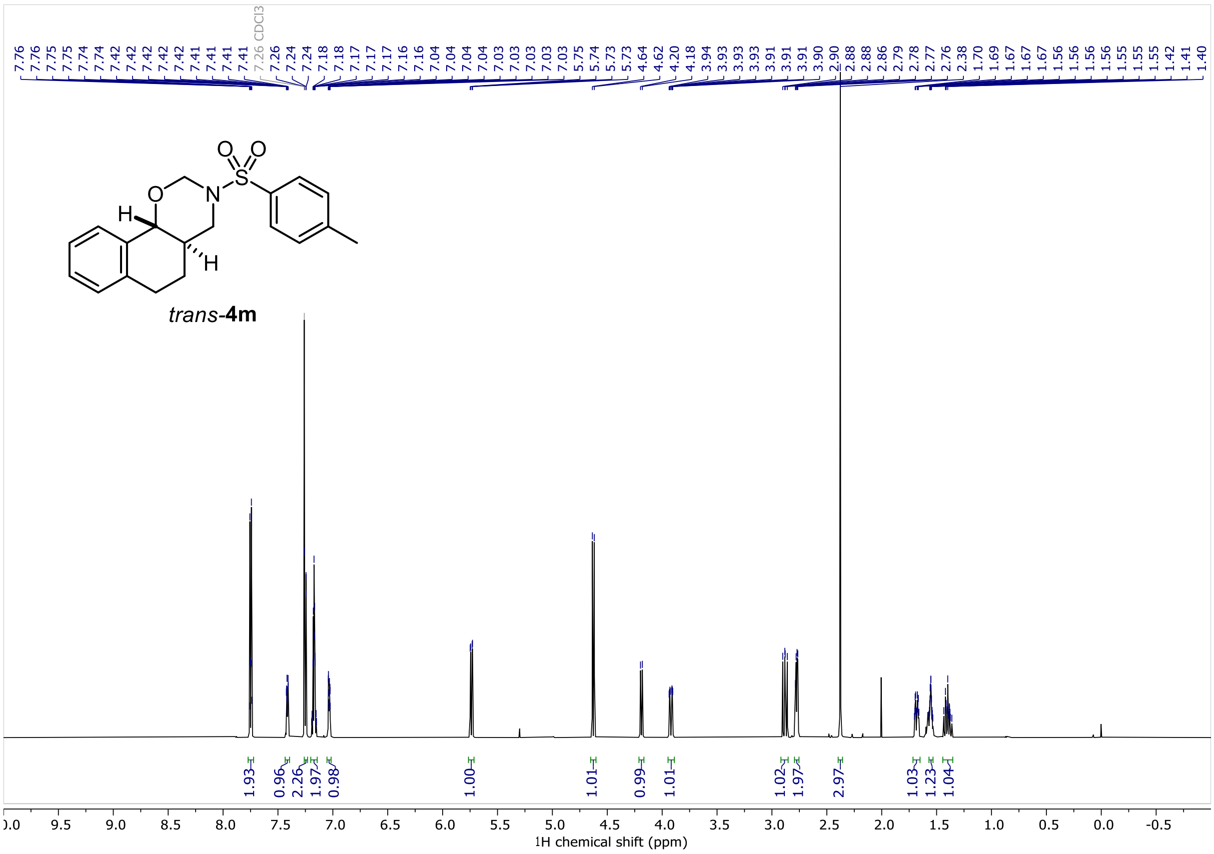


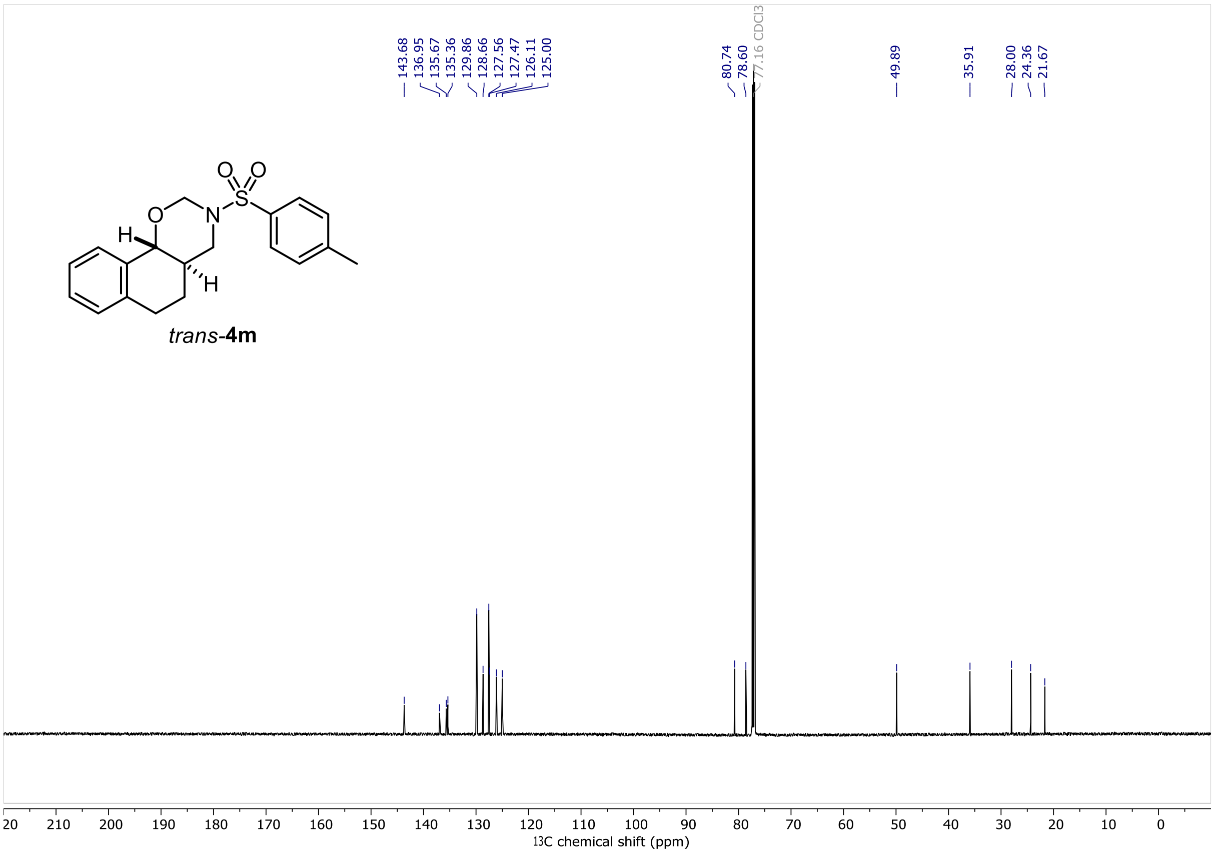


NMR spectra for compound *trans*-**4m**: $^{1}H$ (600 MHz) and $^{13}C$ (151 MHz), in $CDCl_3$.

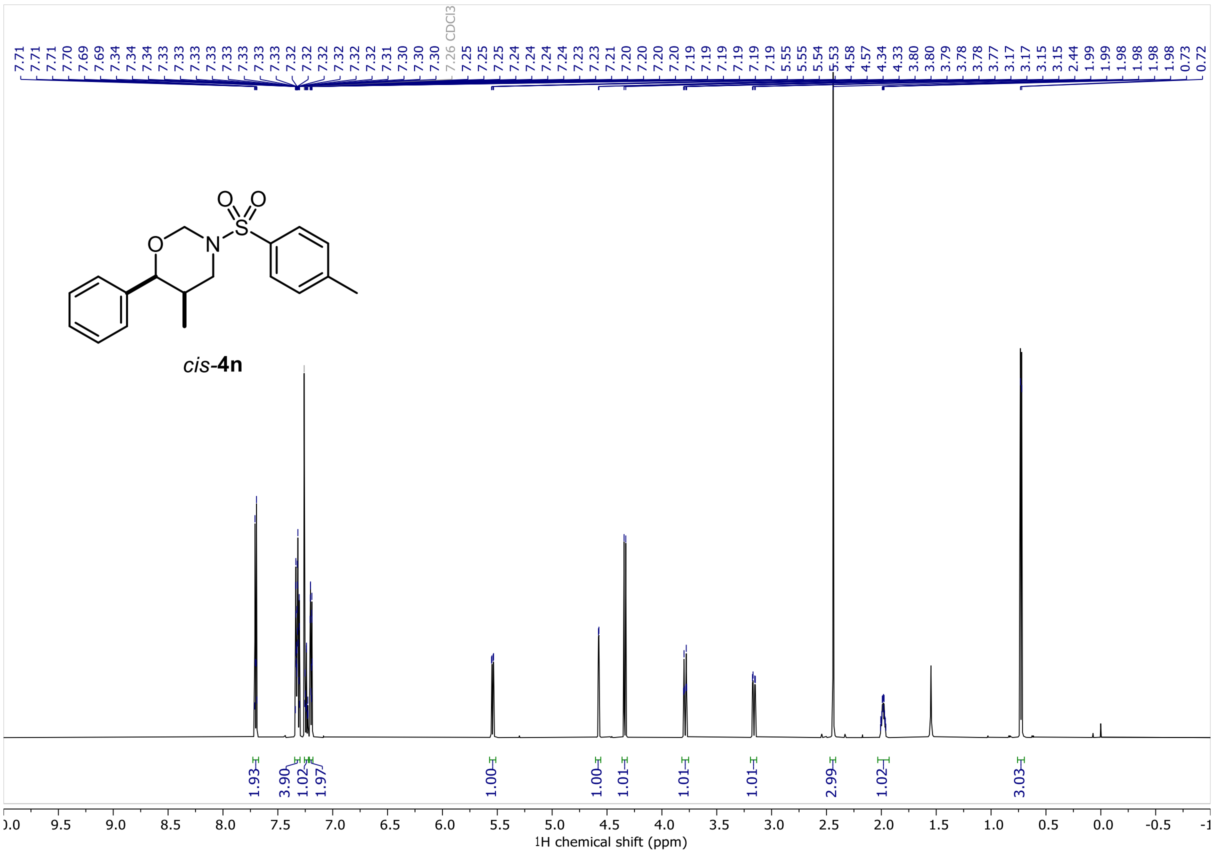


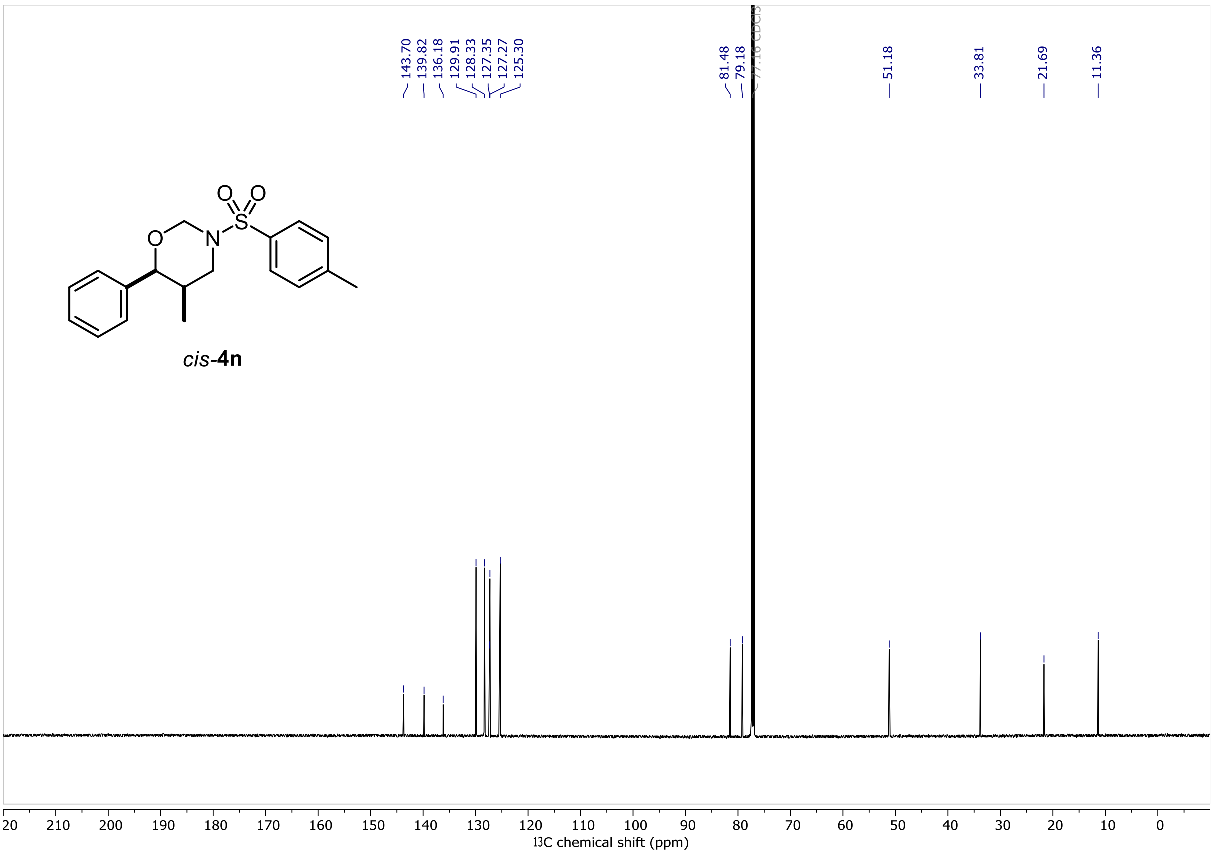


NMR spectra for compound *cis*-**4n**: $^{1}$H (600 MHz) and $^{13}$C (151 MHz), in $CDCl_3$.

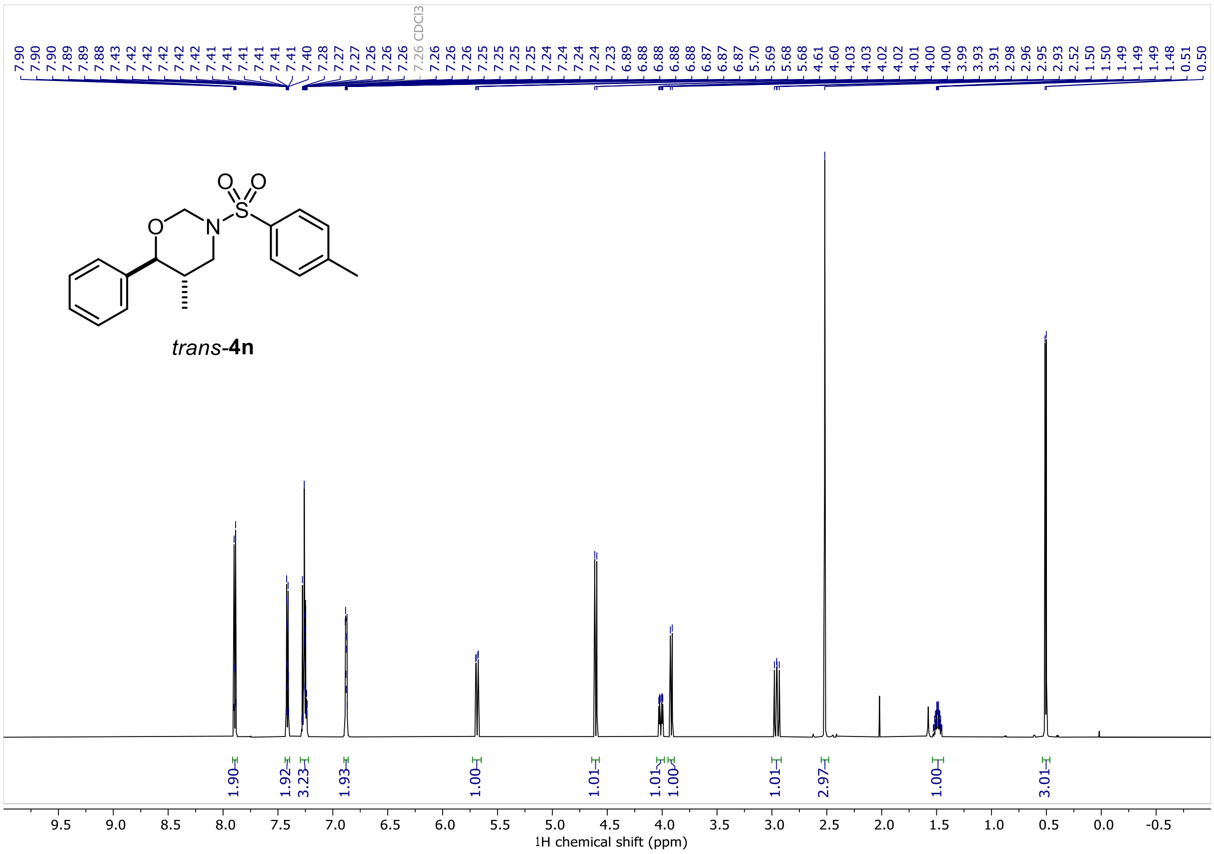

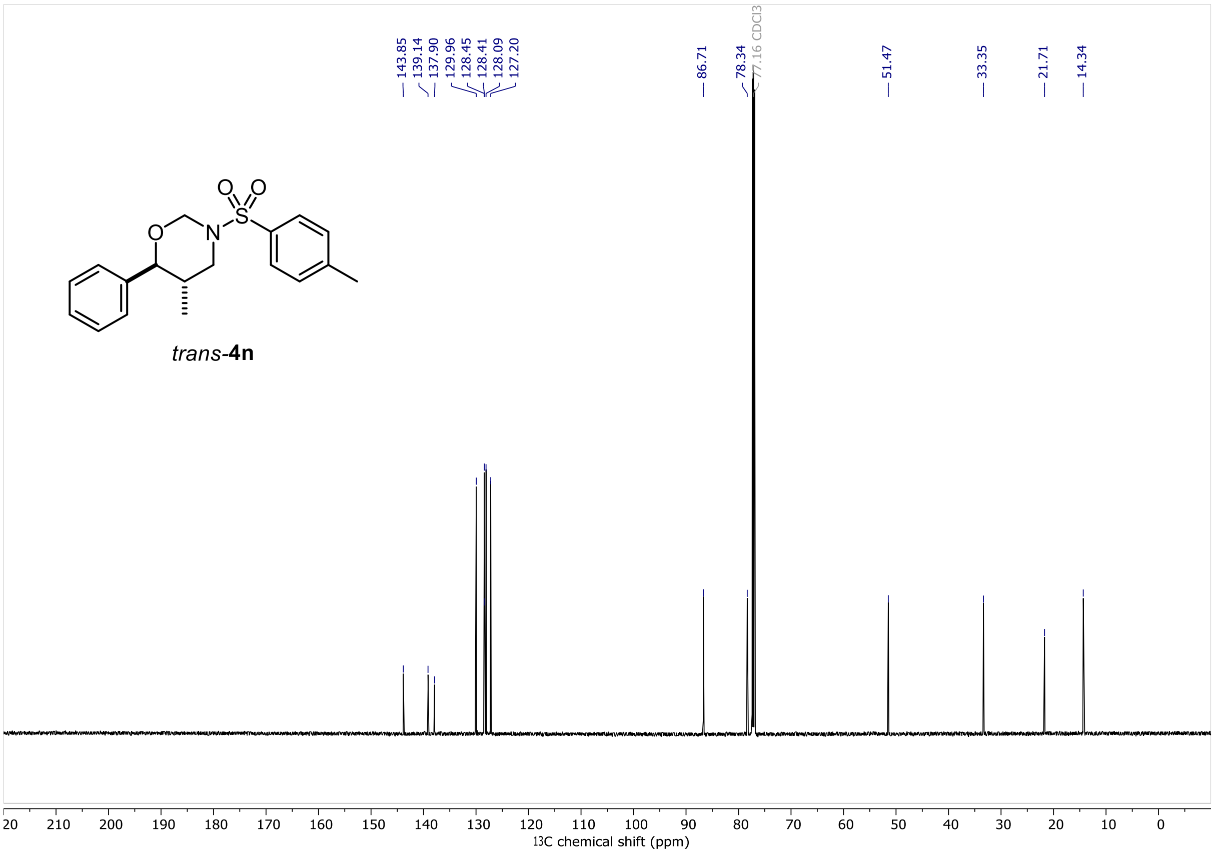

NMR spectra for compound *trans*-**4n**: $^{1}H$ (600 MHz) and $^{13}C$ (151 MHz), in $CDCl_3$.

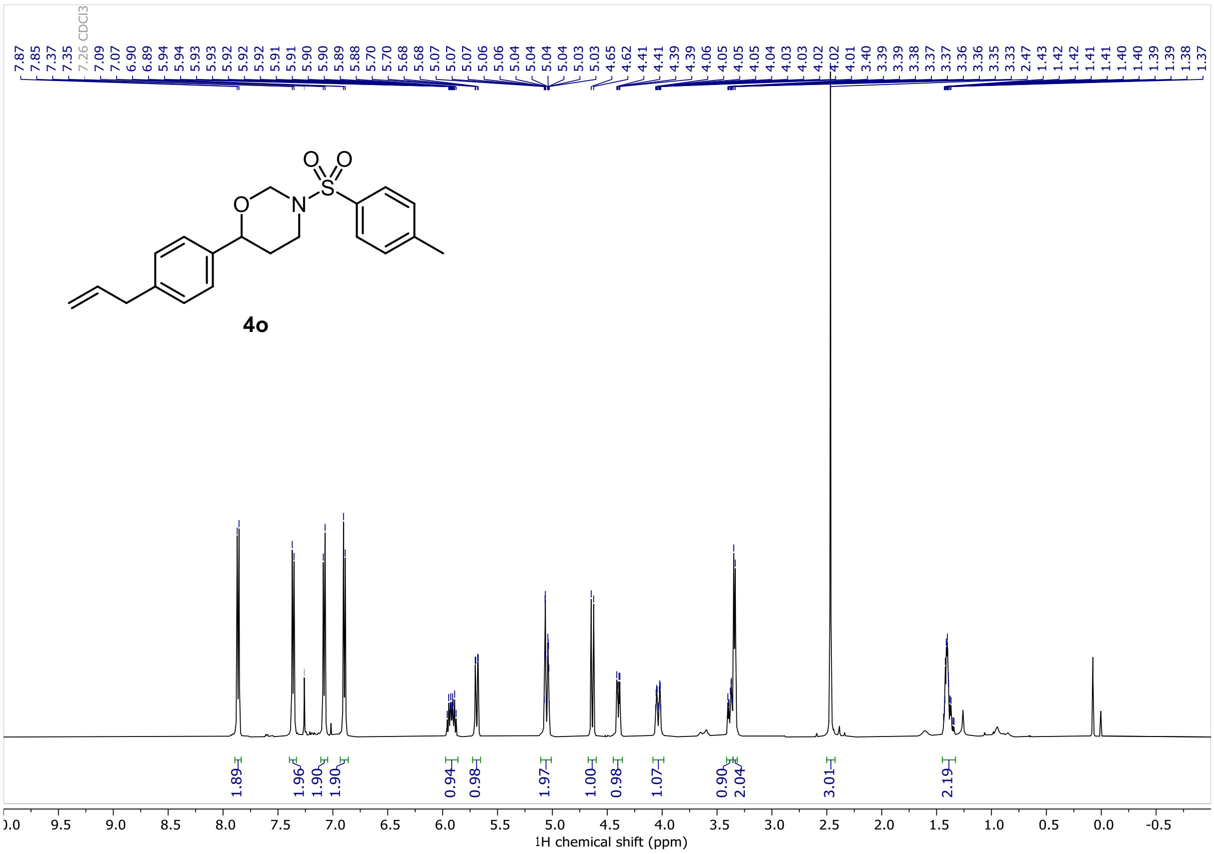


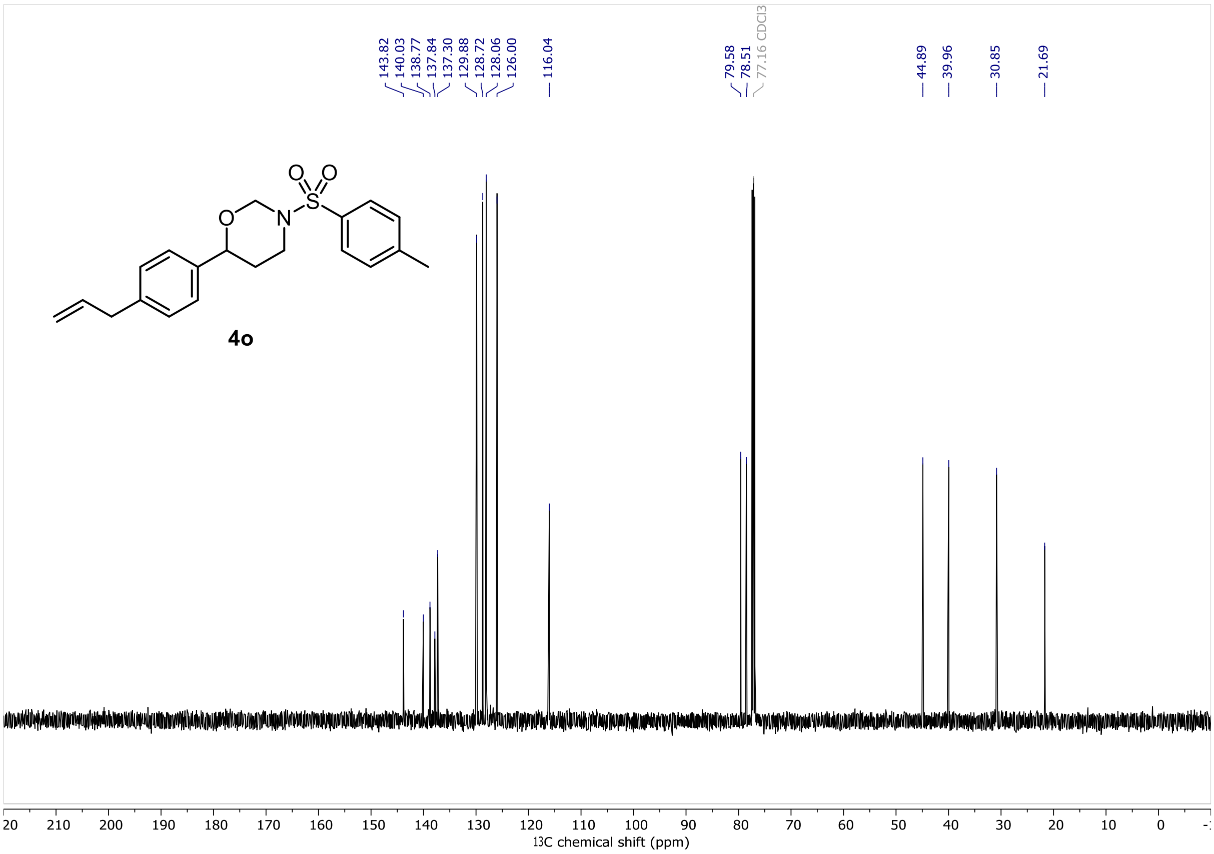


NMR spectra for compound **4o**: $^{1}$H (501 MHz) and $^{13}$C (126 MHz), in $CDCl_3$.

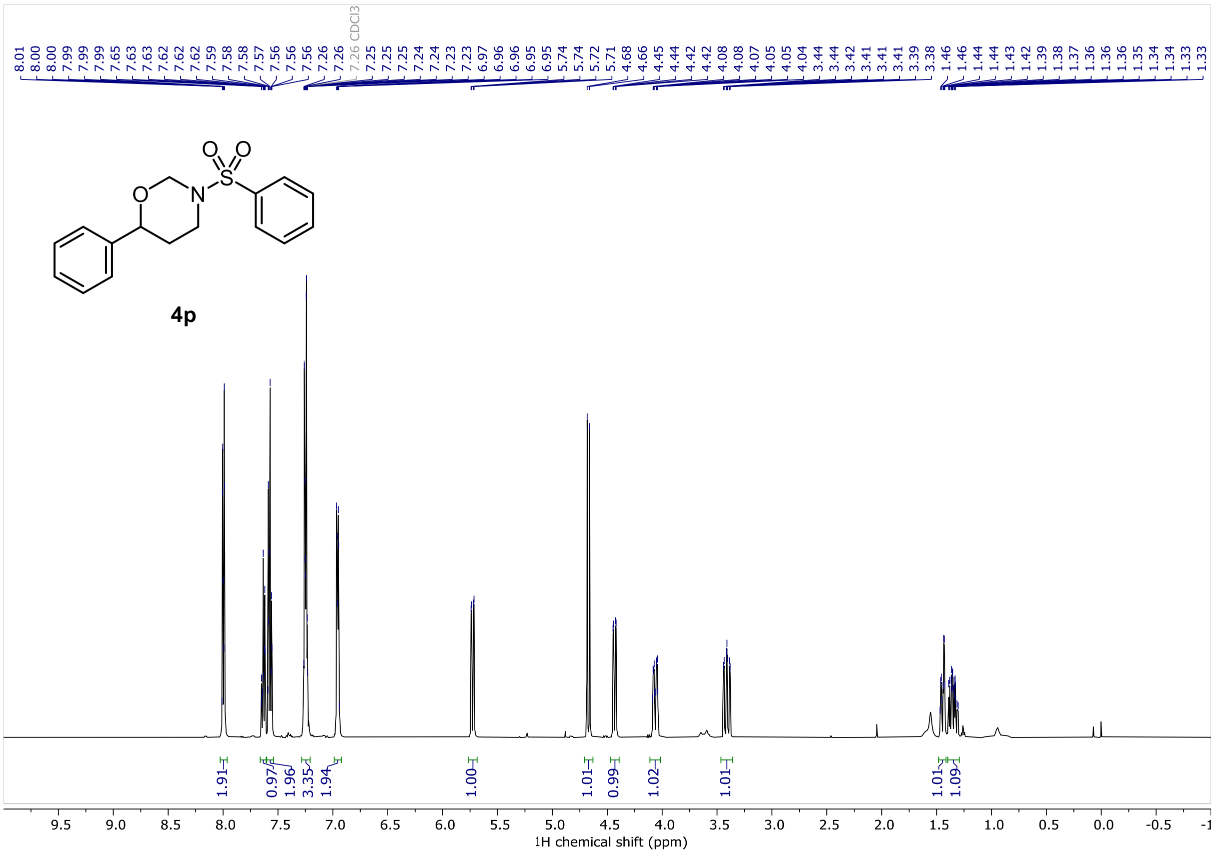


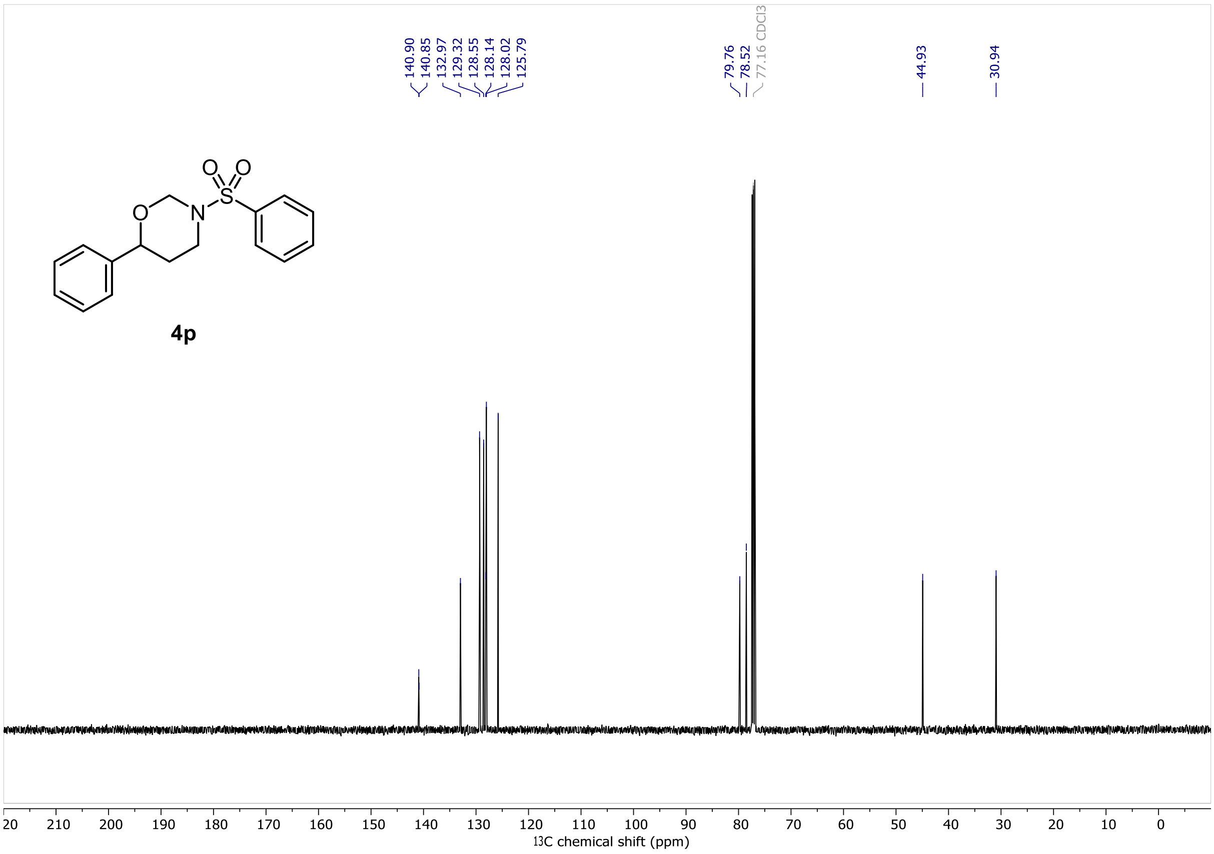


NMR spectra for compound **4p**: $^{1}H$ (501 MHz) and $^{13}C$ (126 MHz), in $CDCl_3$.

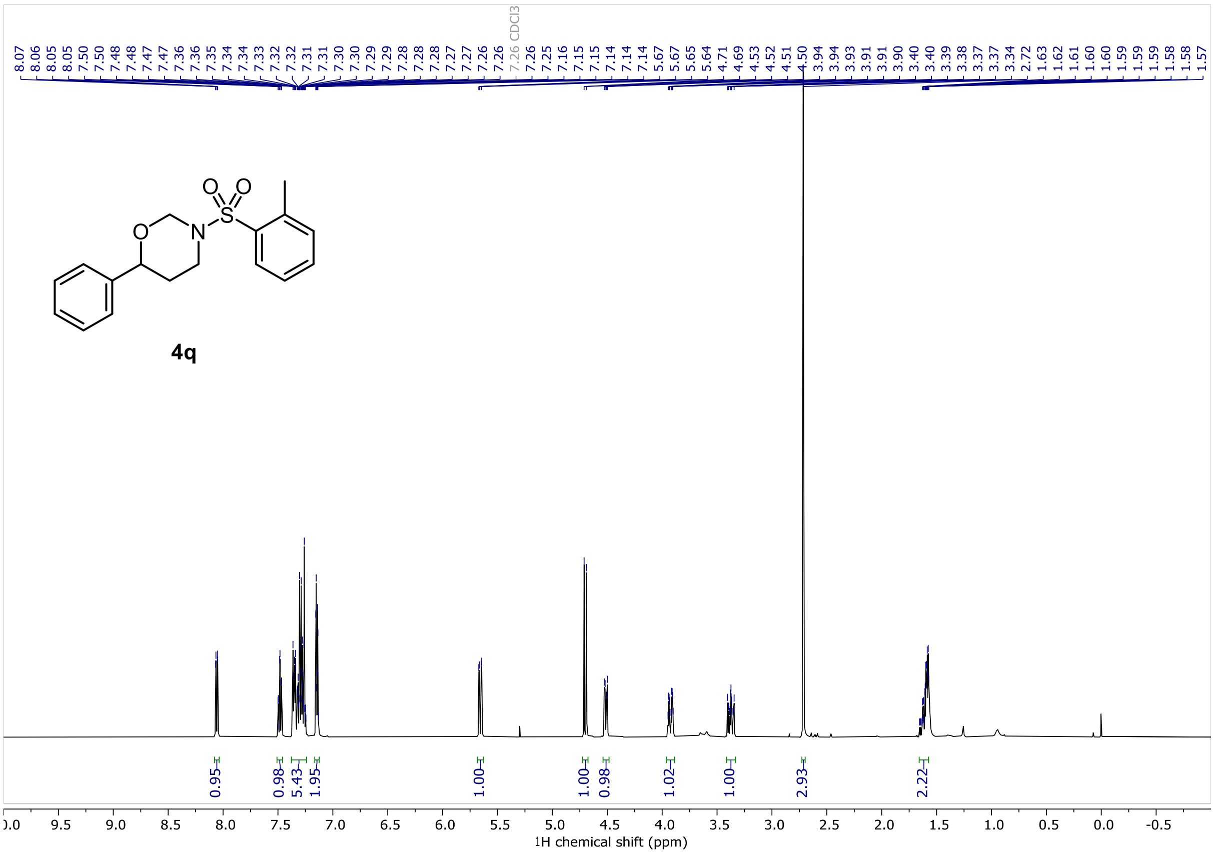

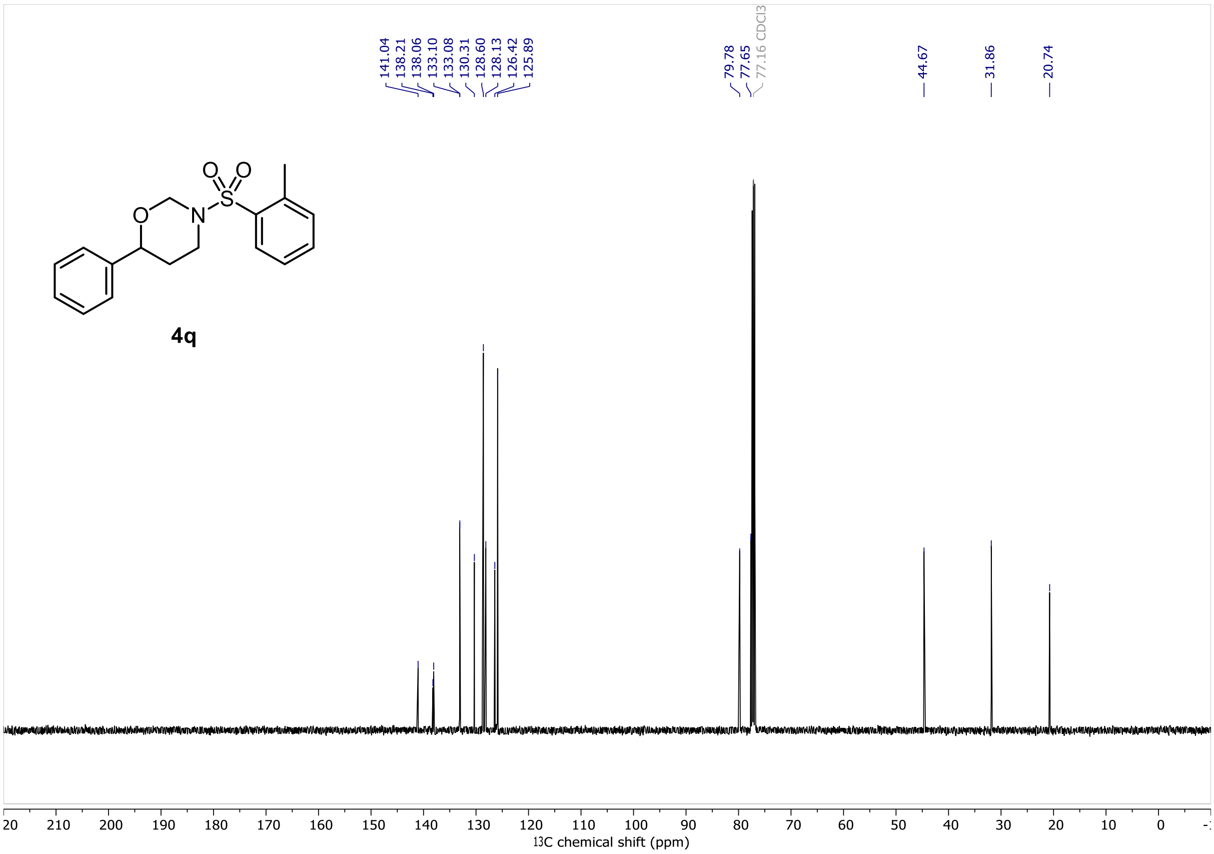

NMR spectra for compound **4q**: $^{1}$H (501 MHz) and $^{13}$C (126 MHz), in $CDCl_3$.

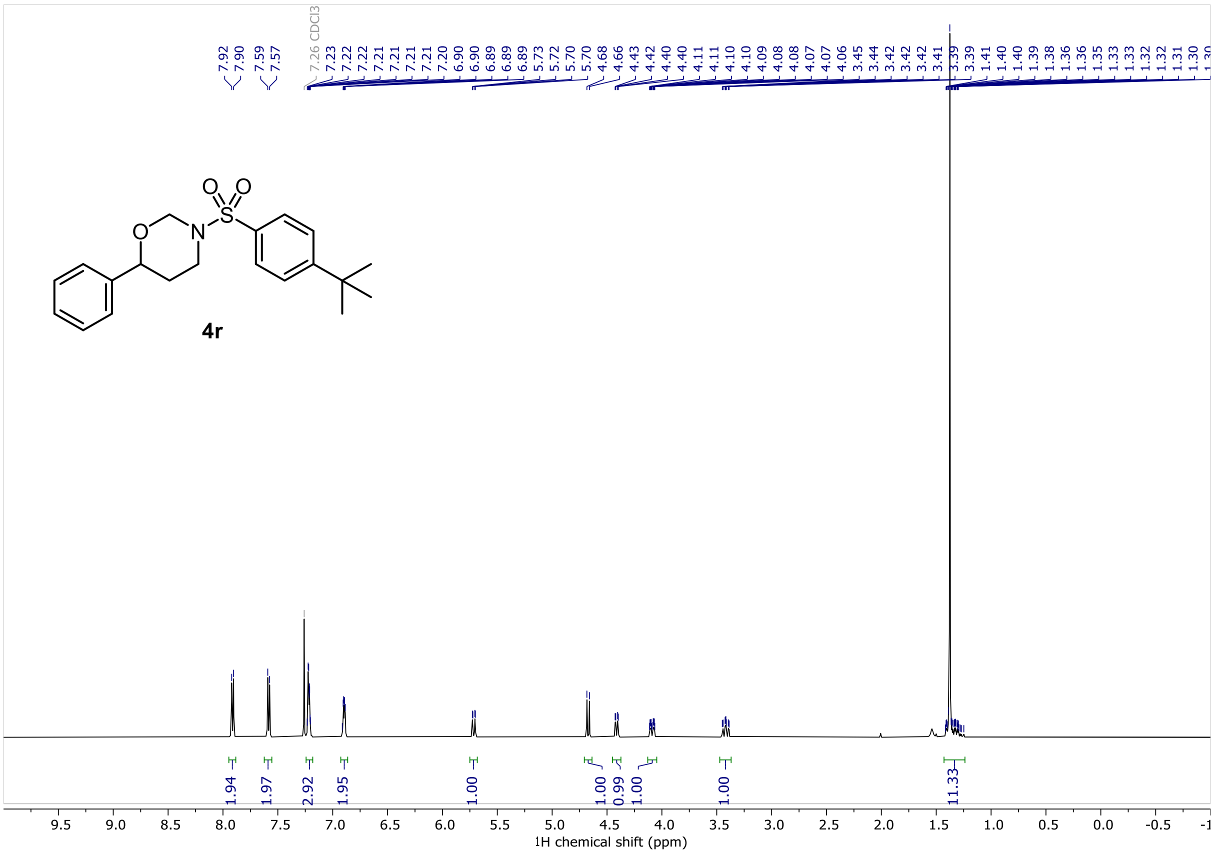

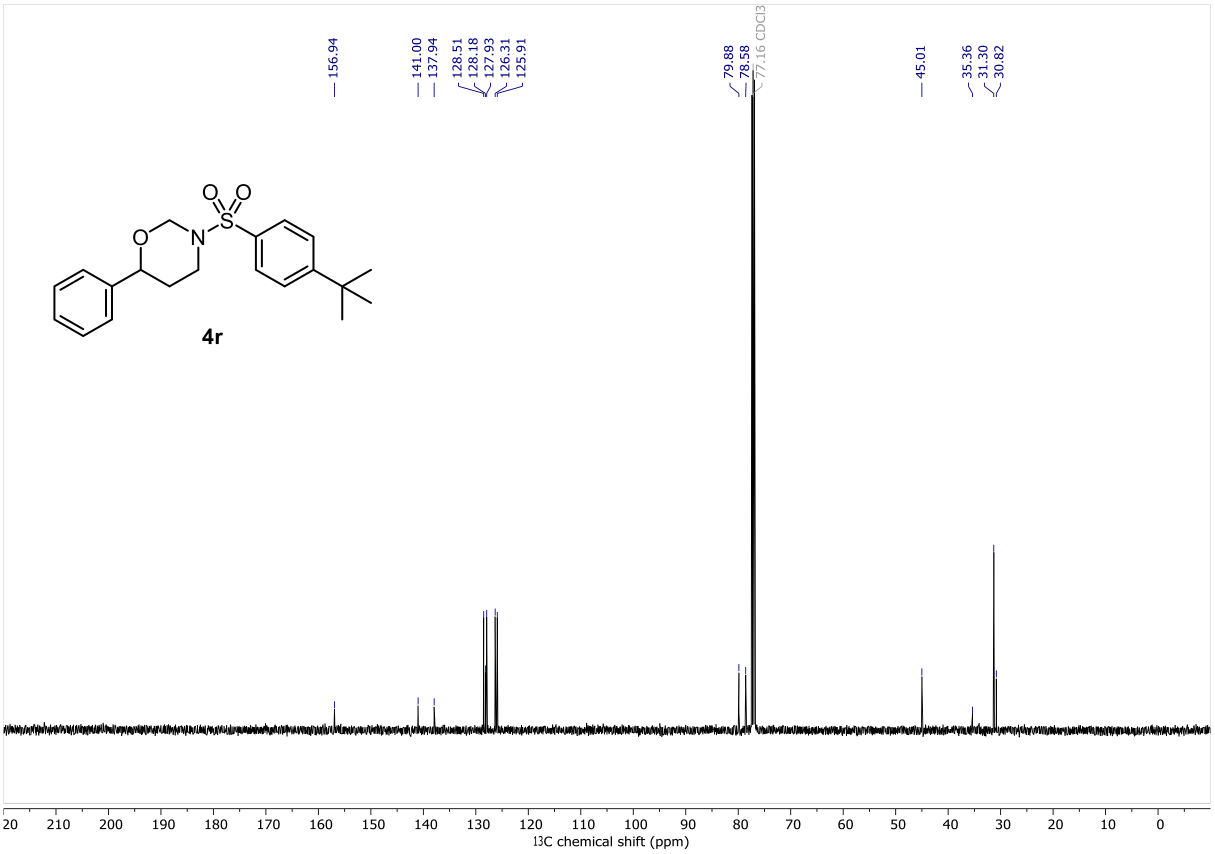

NMR spectra for compound **4r**: $^{1}$H (501 MHz) and $^{13}$C (126 MHz), in $CDCl_3$.

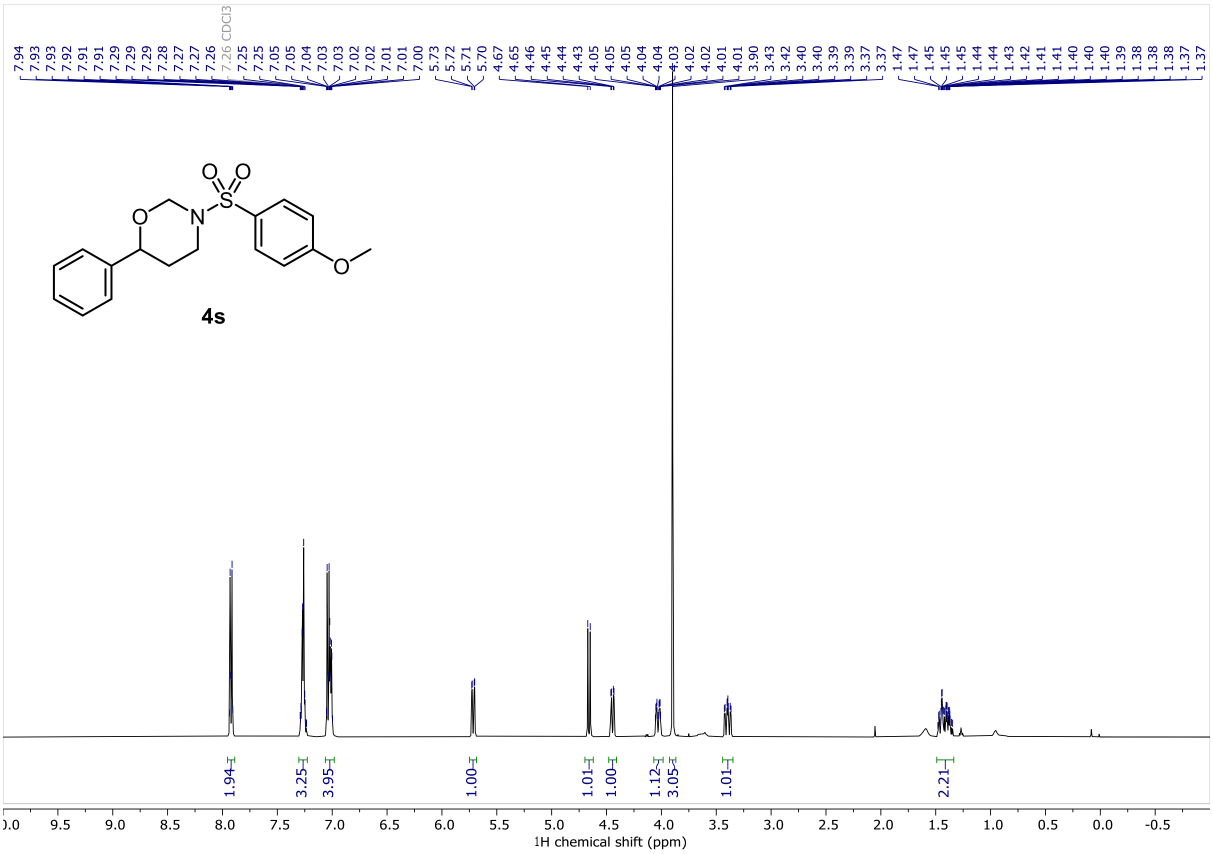


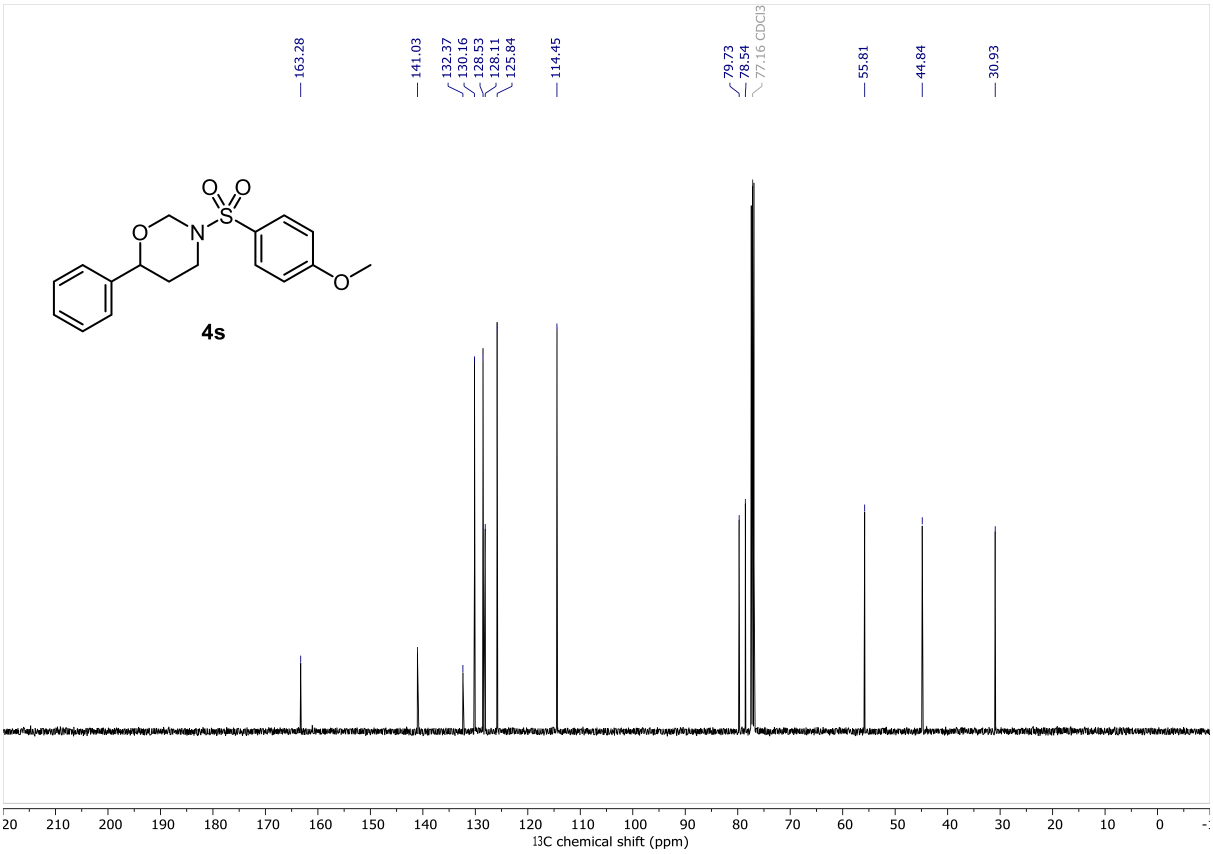


NMR spectra for compound **4s**: $^{1}$H (501 MHz) and $^{13}$C (126 MHz), in $CDCl_3$.

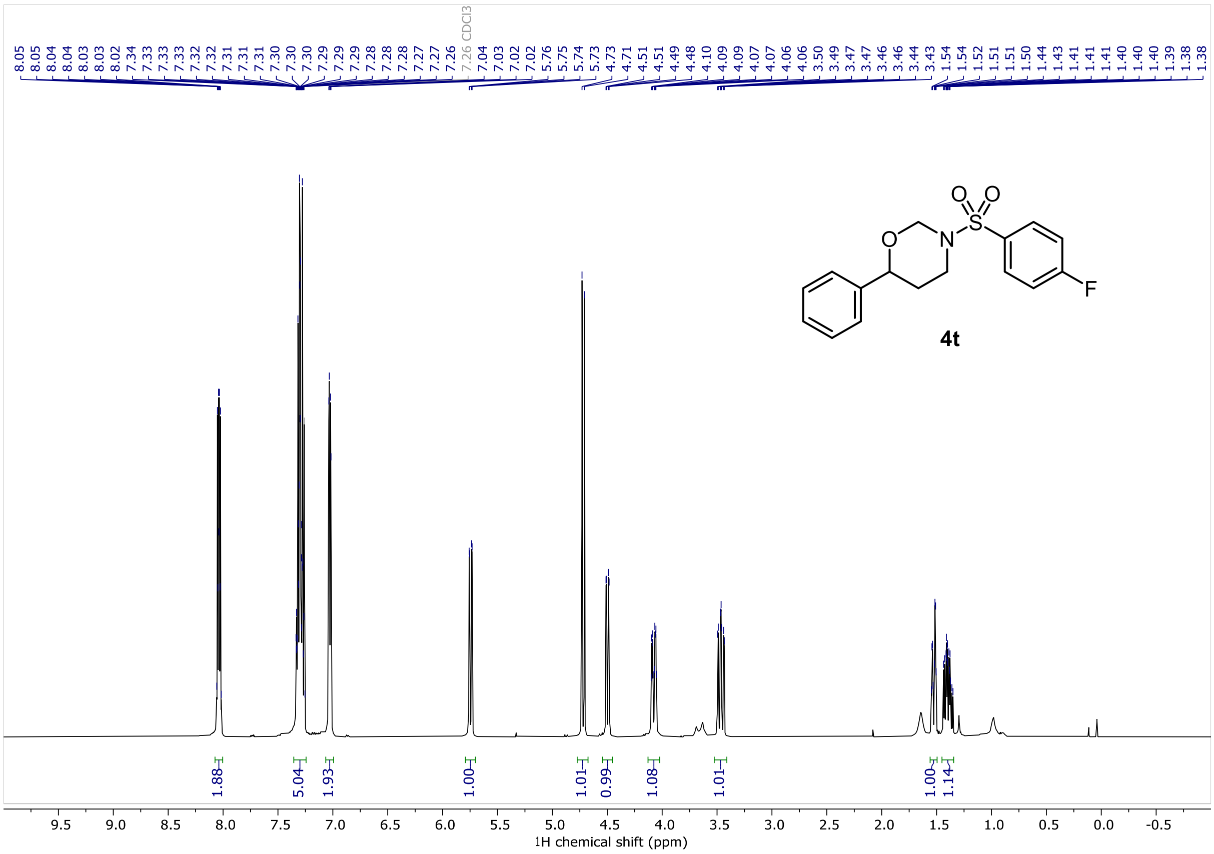


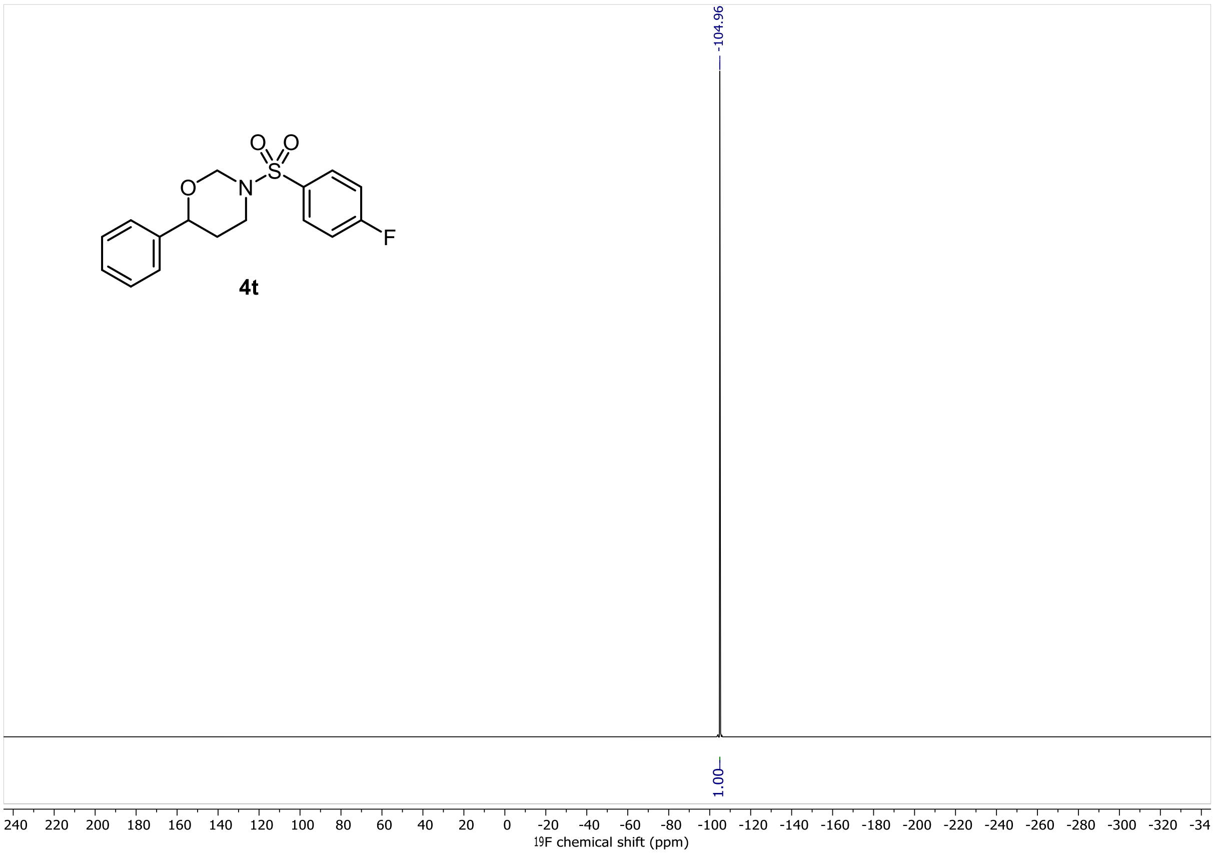


NMR spectra for compound **4t**: $^{1}H$ (501 MHz) and $^{19}F$ (471 MHz), in $CDCl_3$.

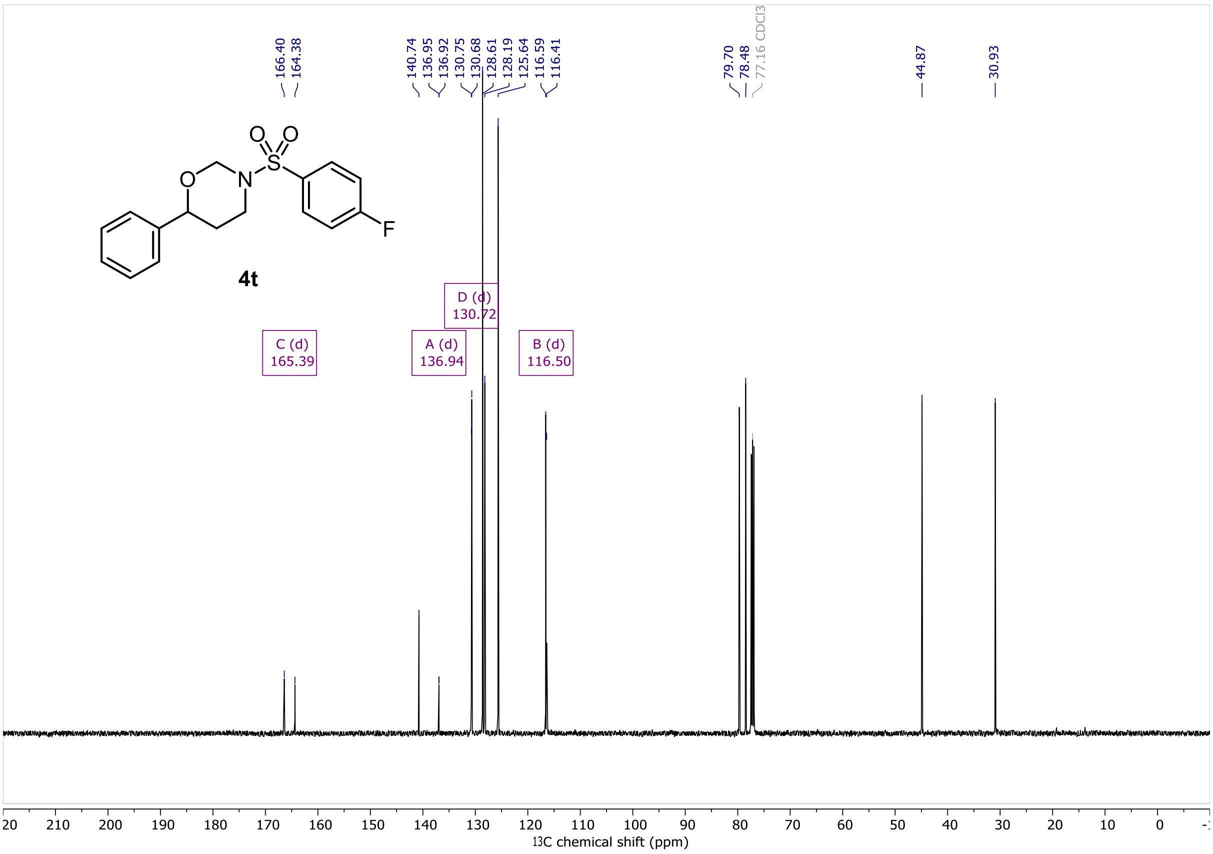


NMR spectra for compound **4t** *(continuation)*: $^{13}C$ (126 MHz), in $CDCl_3$.

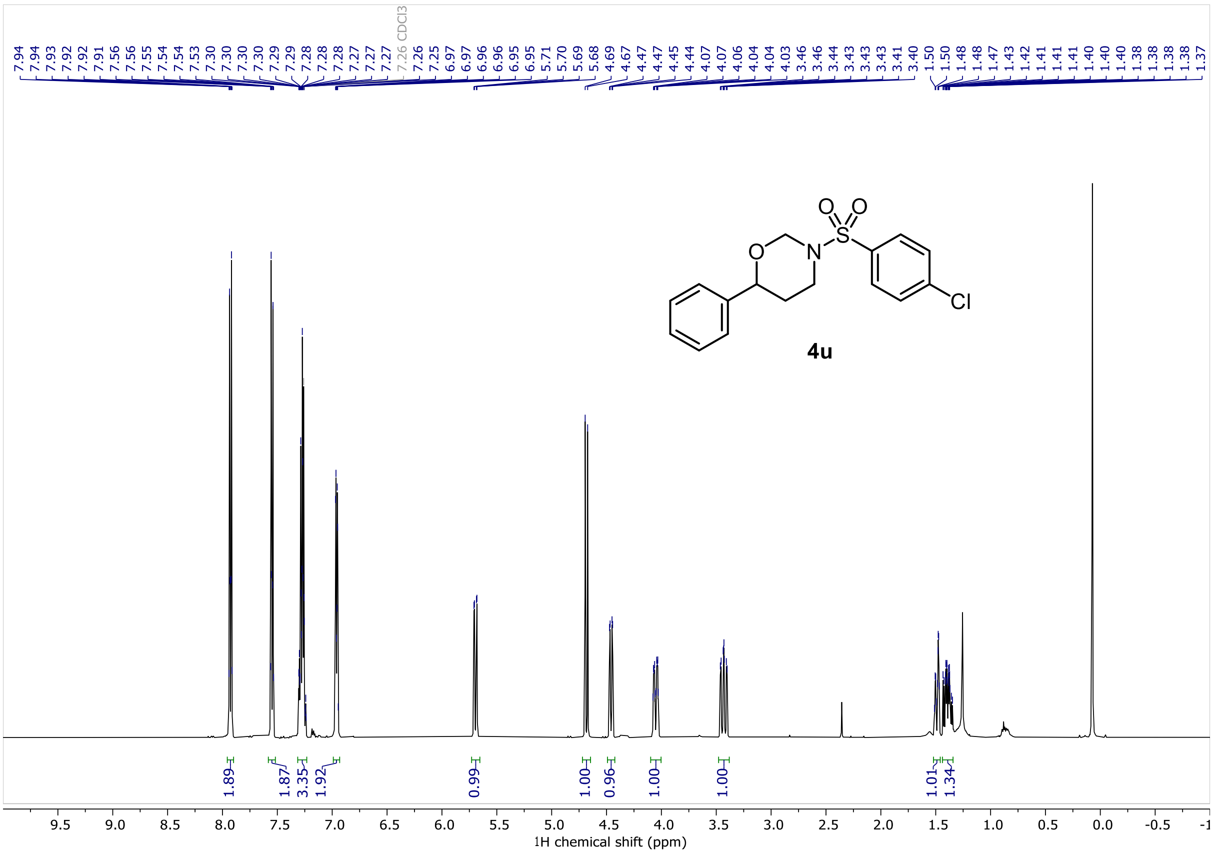

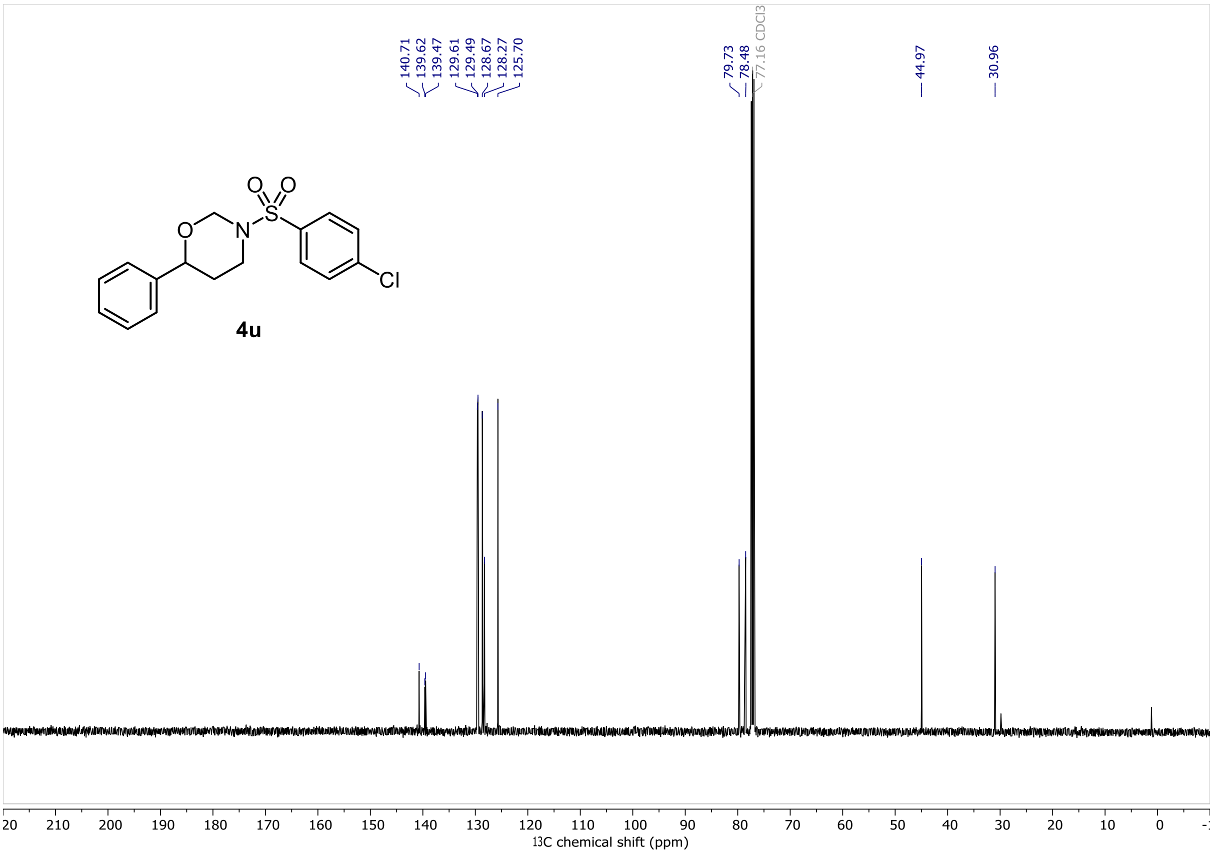

NMR spectra for compound **4u**: $^{1}$H (501 MHz) and $^{13}$C (126 MHz), in $CDCl_3$.

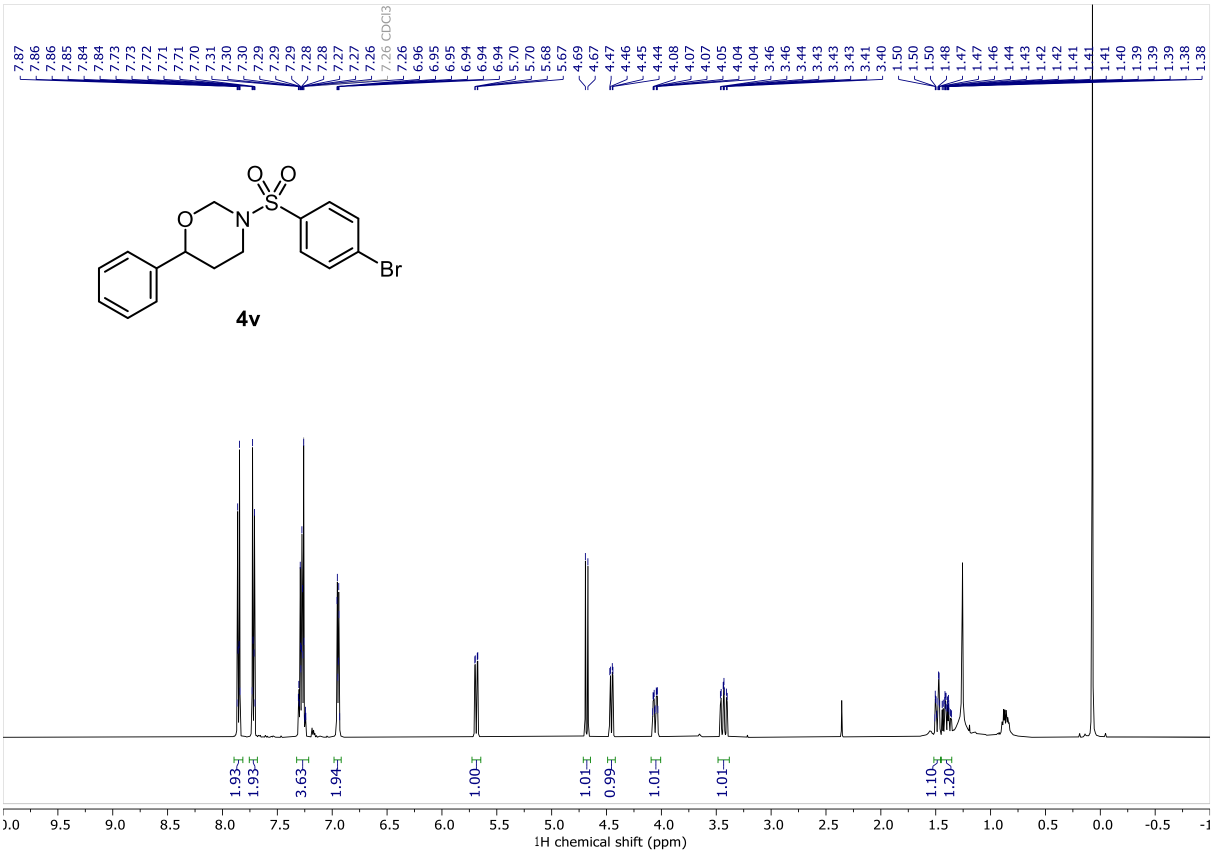


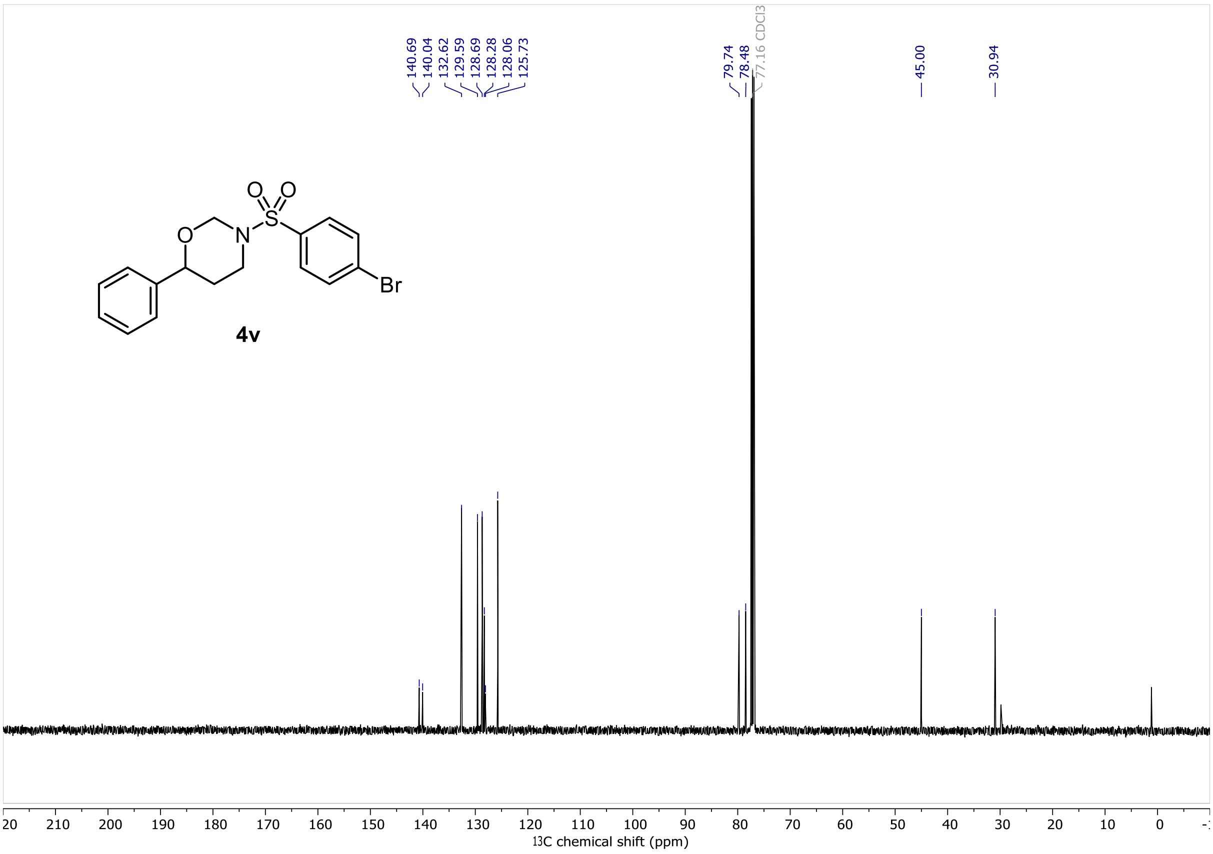


NMR spectra for compound **4v**: $^{1}$H (501 MHz) and $^{13}$C (126 MHz), in $CDCl_3$.

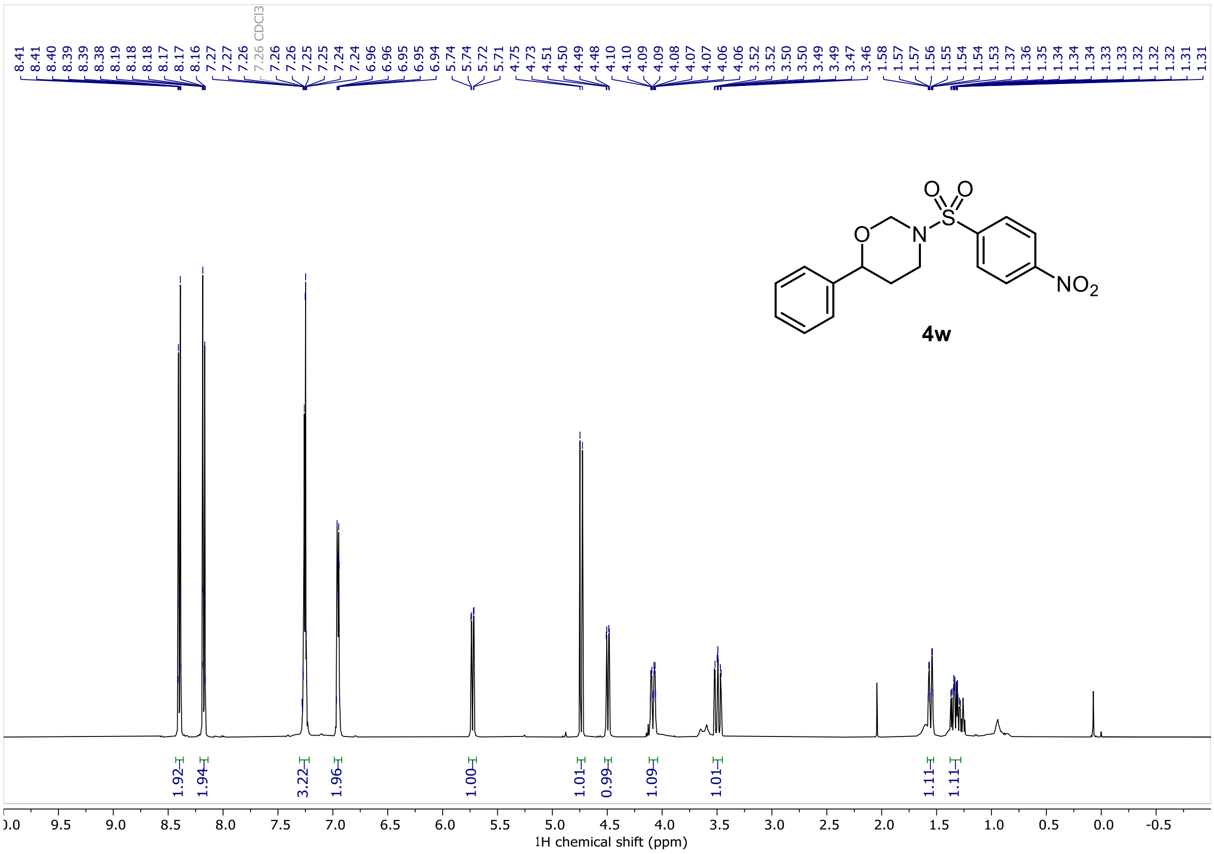

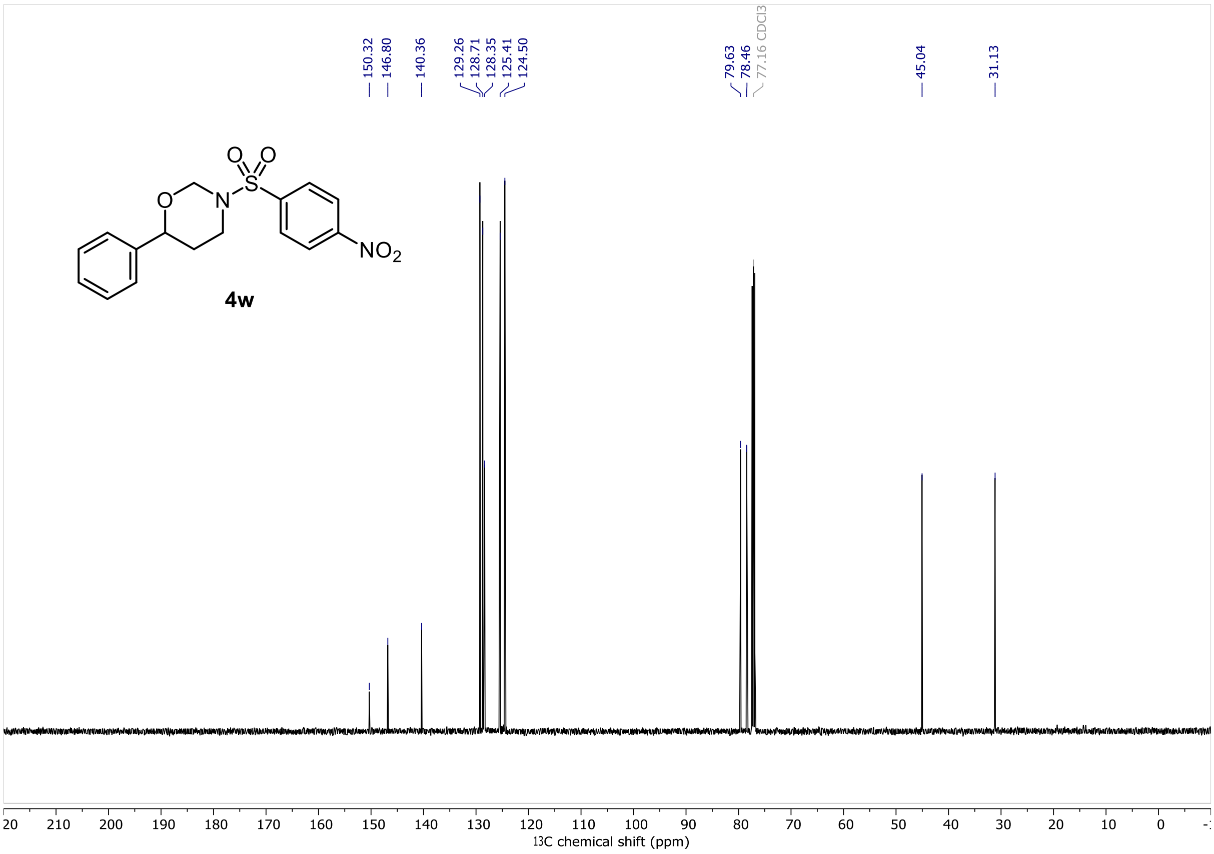

NMR spectra for compound **4w**: $^{1}H$ (501 MHz) and $^{13}C$ (126 MHz), in $CDCl_3$.

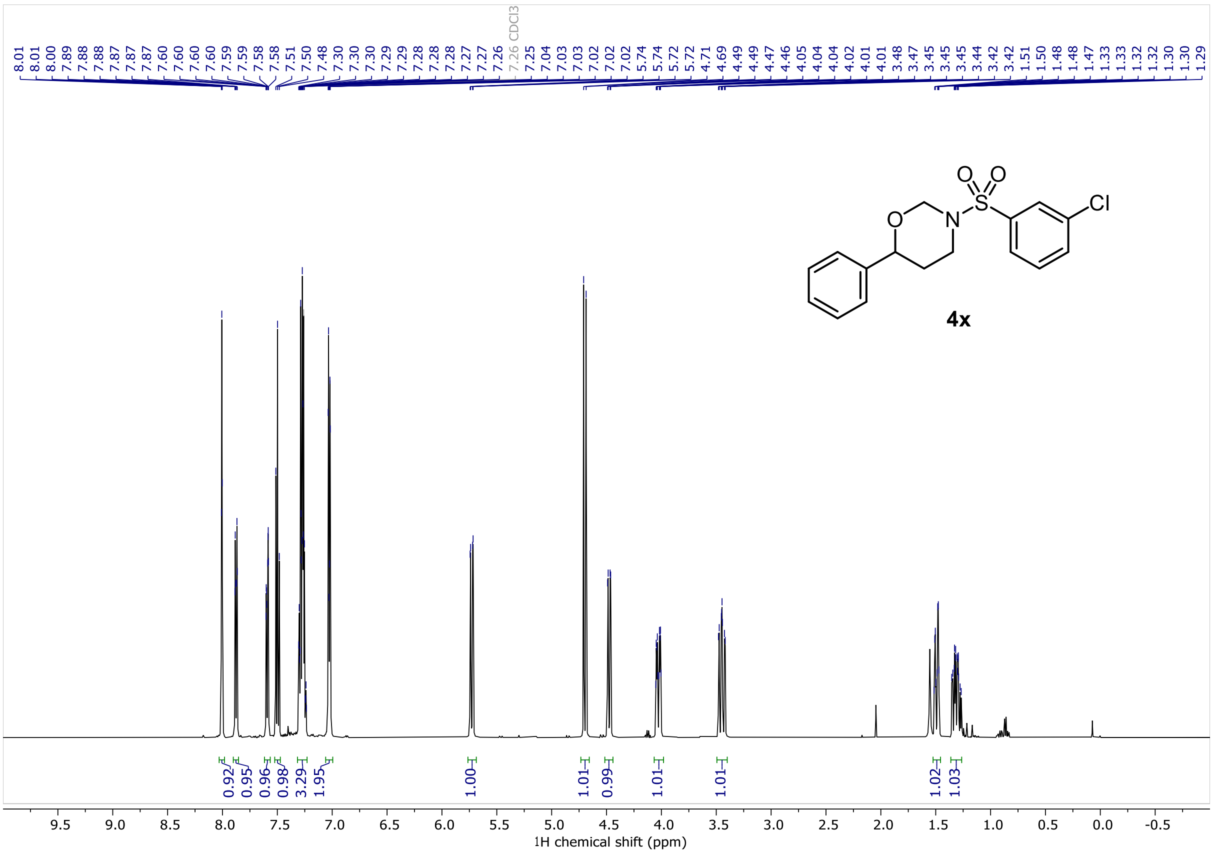

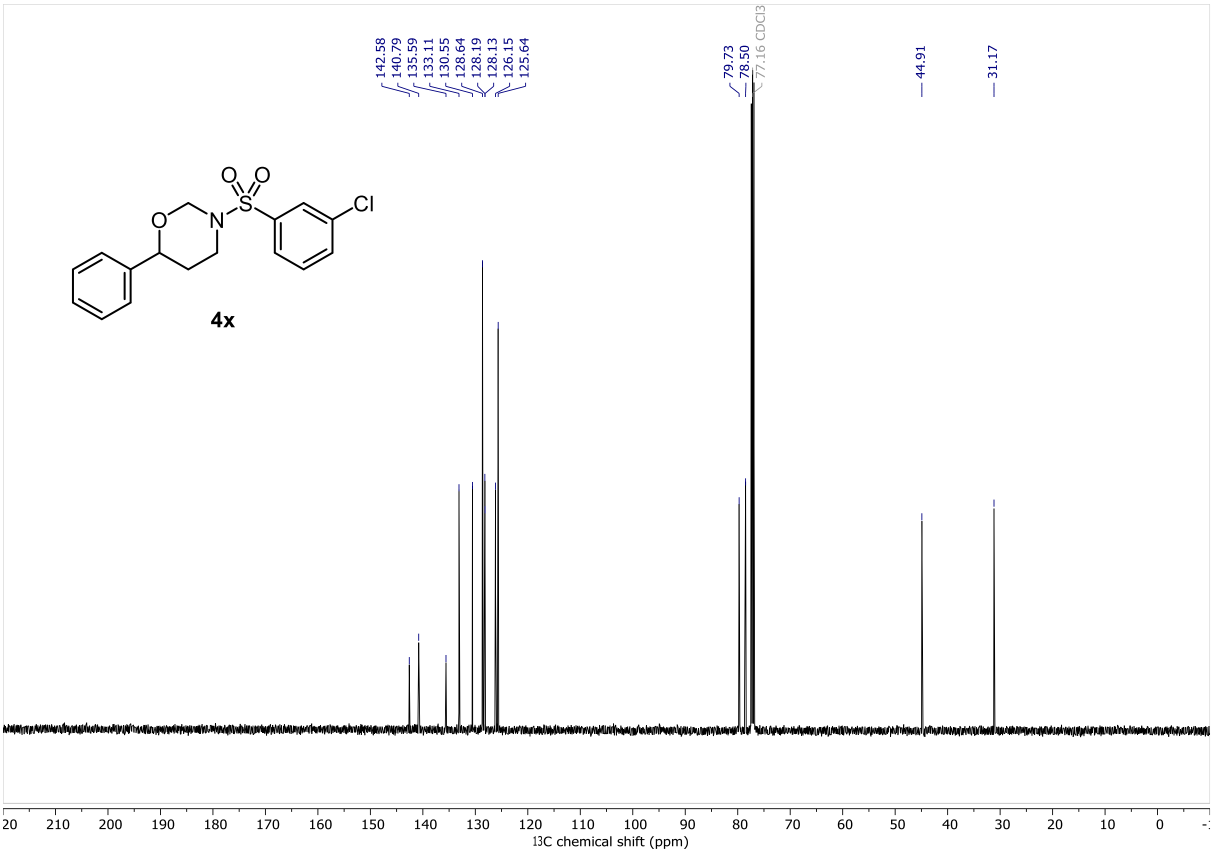

NMR spectra for compound **4x**: $^{1}$H (501 MHz) and $^{13}$C (126 MHz), in $CDCl_3$.

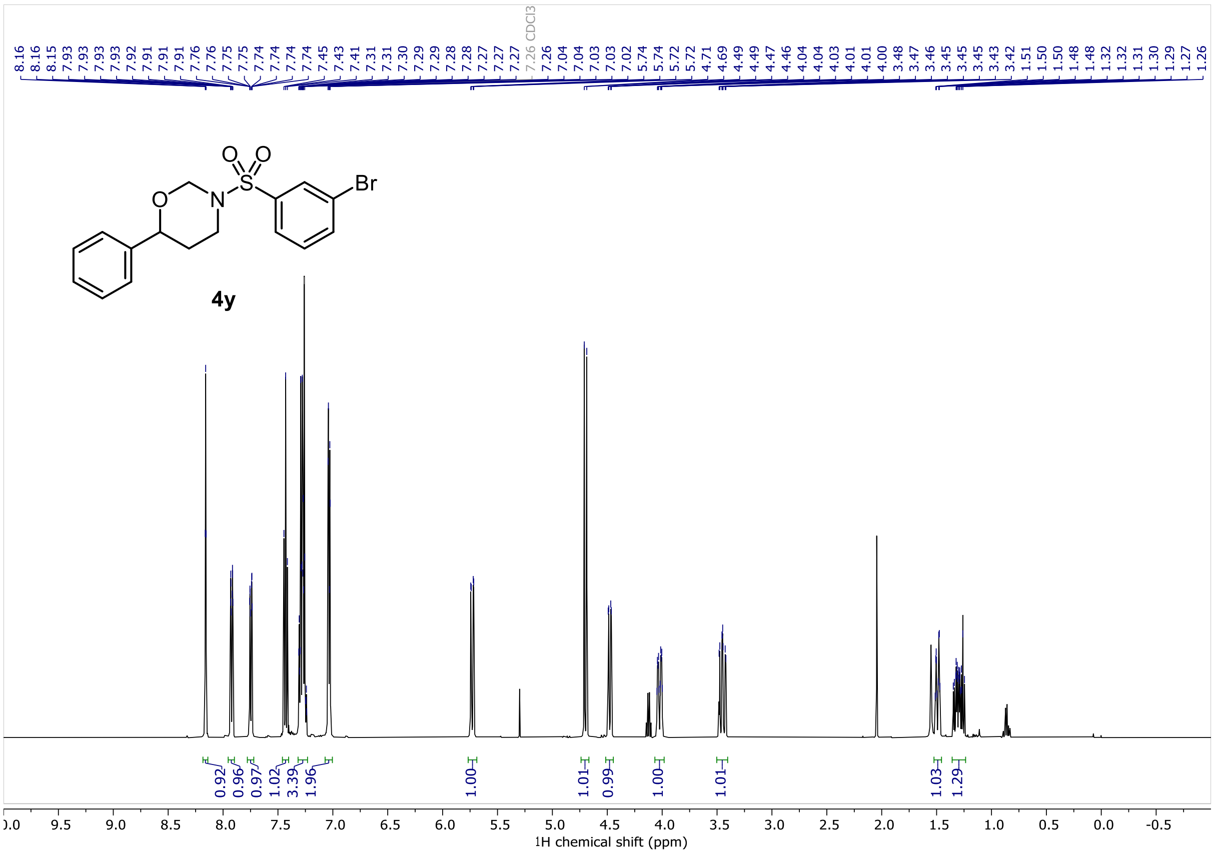

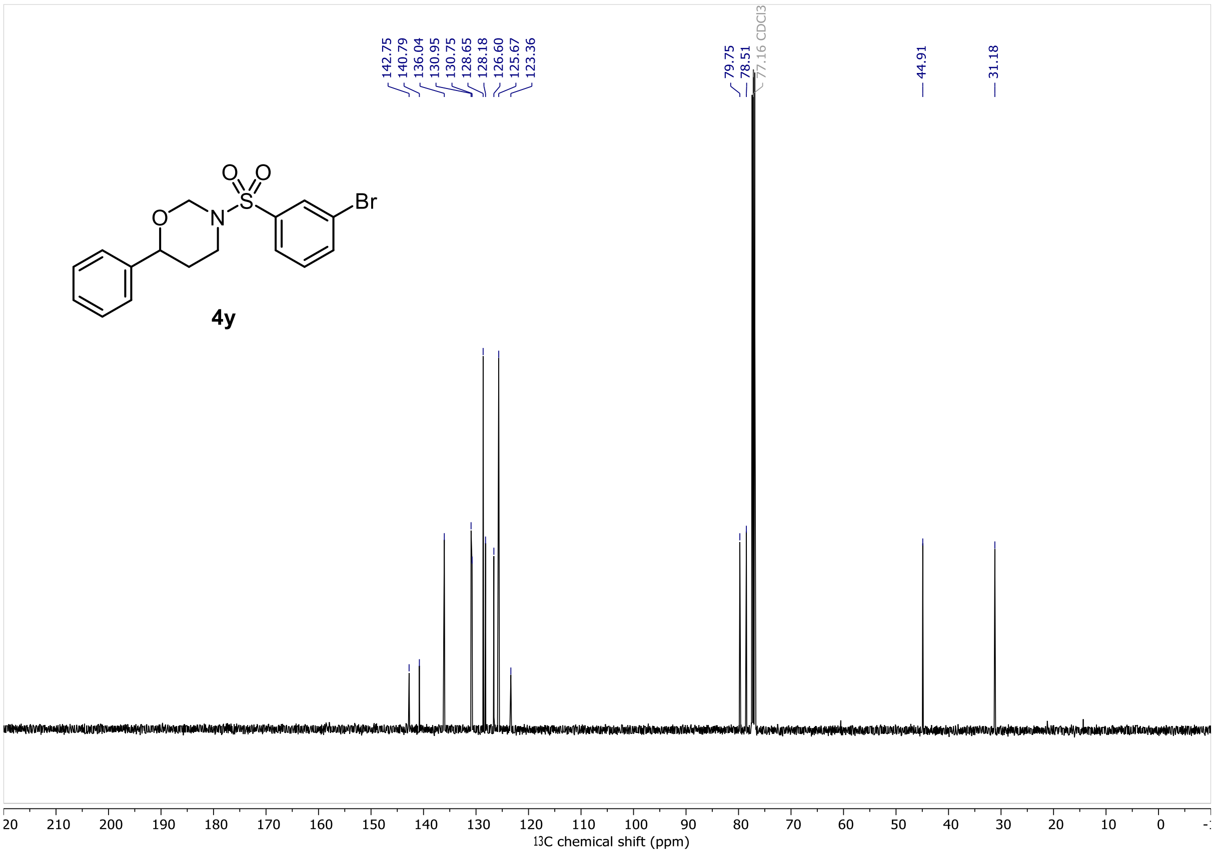

NMR spectra for compound **4y**: $^{1}$H (501 MHz) and $^{13}$C (126 MHz), in $CDCl_3$.

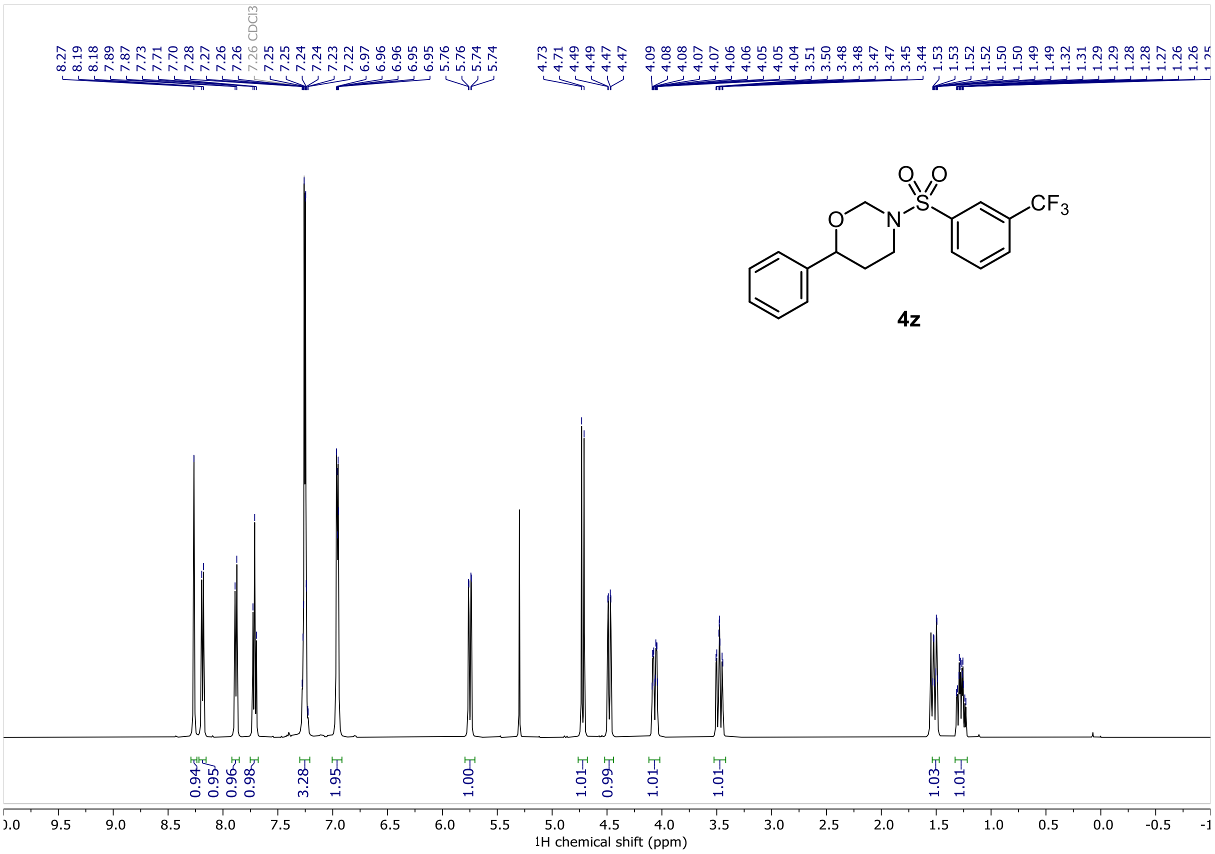


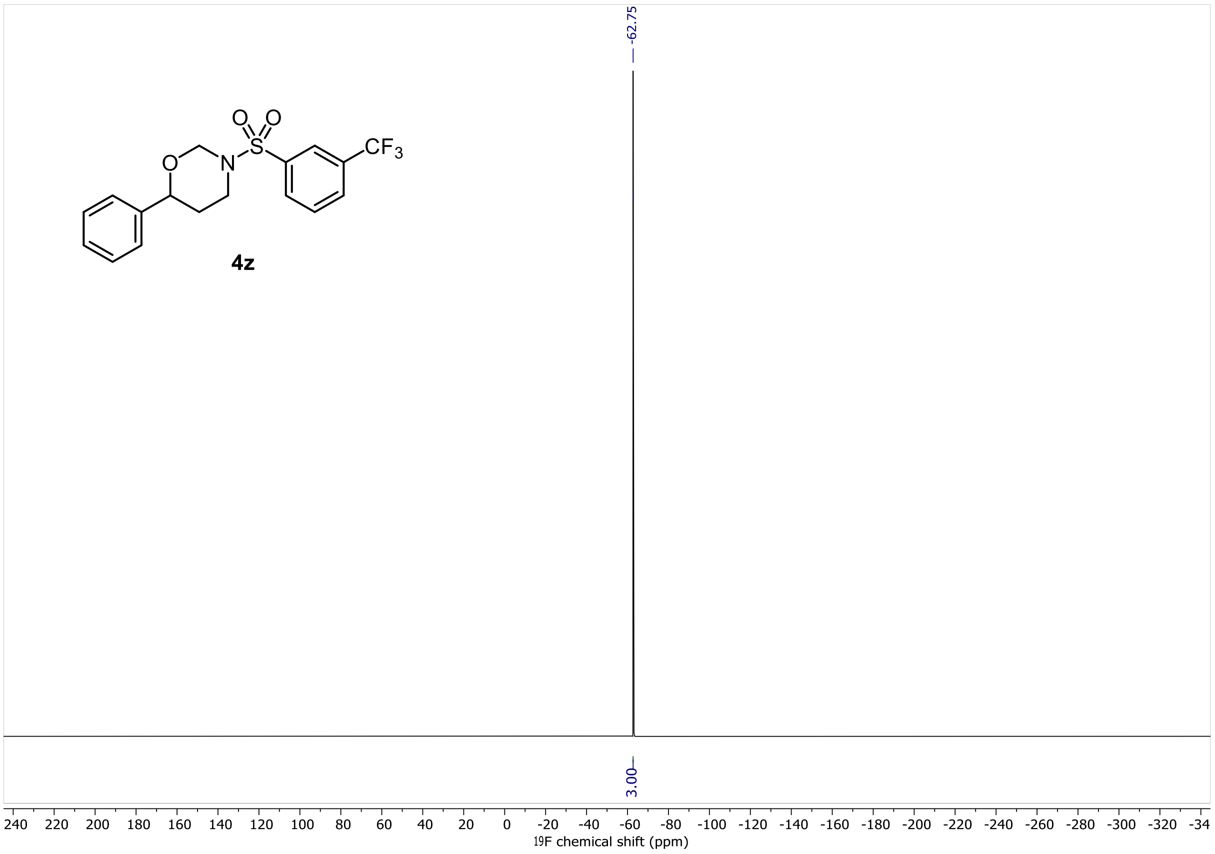


NMR spectra for compound **4z**: $^{1}$H (501 MHz) and $^{19}$F (471 MHz), in $CDCl_3$.

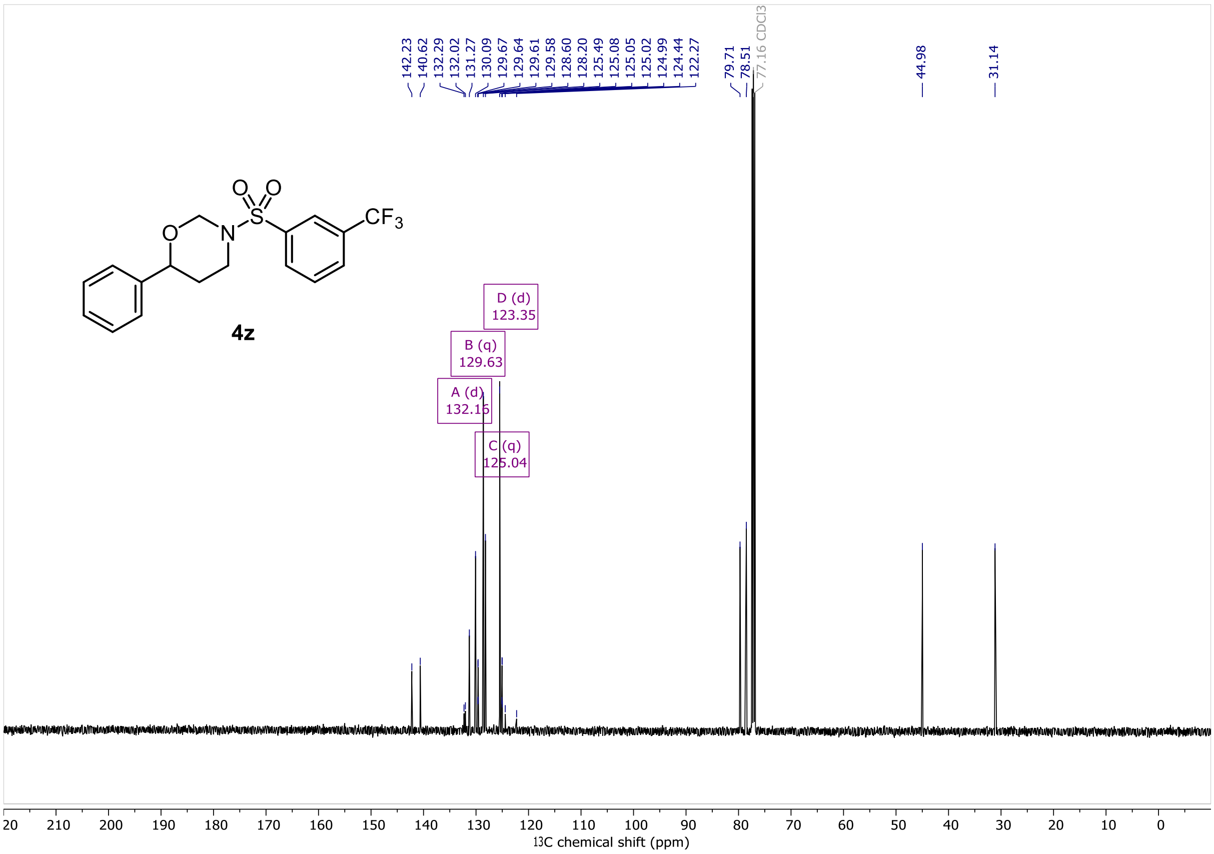


NMR spectra for compound **4z** *(continuation)*: $^{13}C$ (126 MHz), in $CDCl_3$.

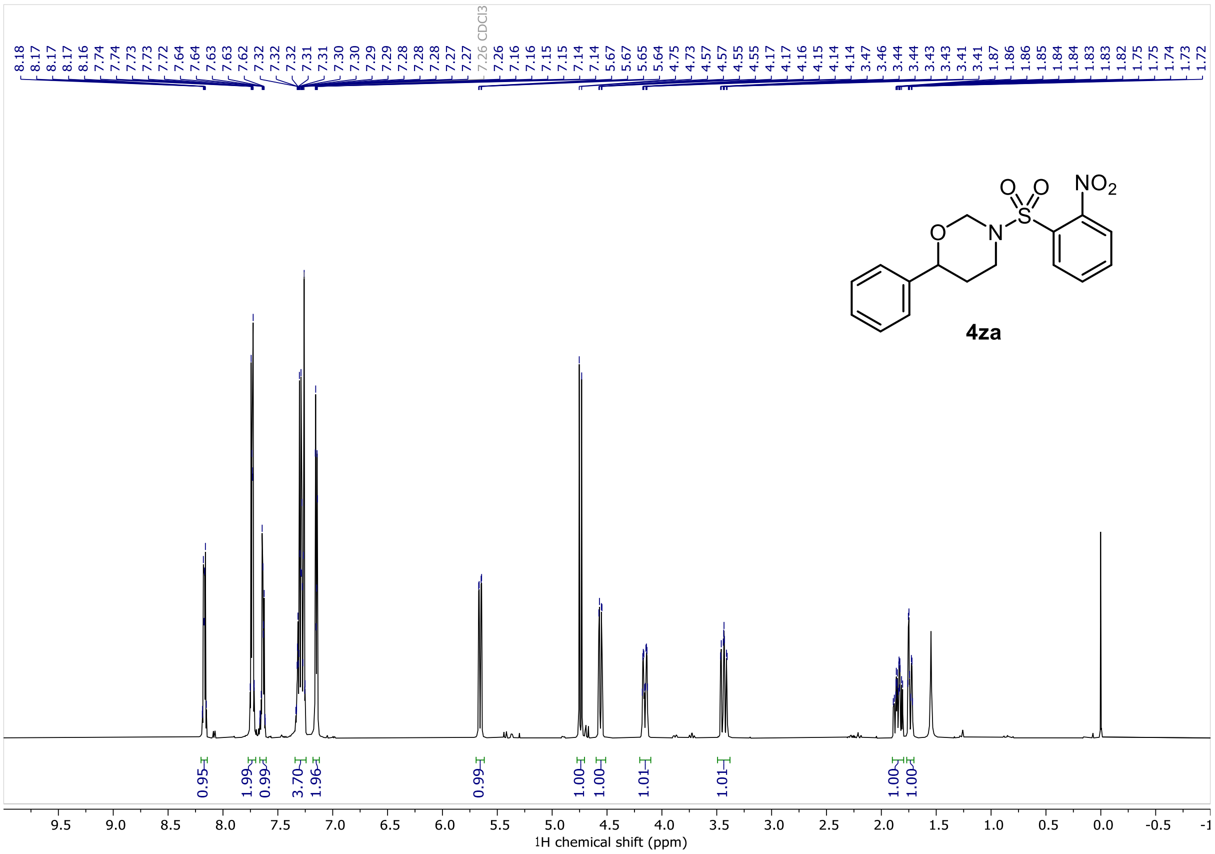

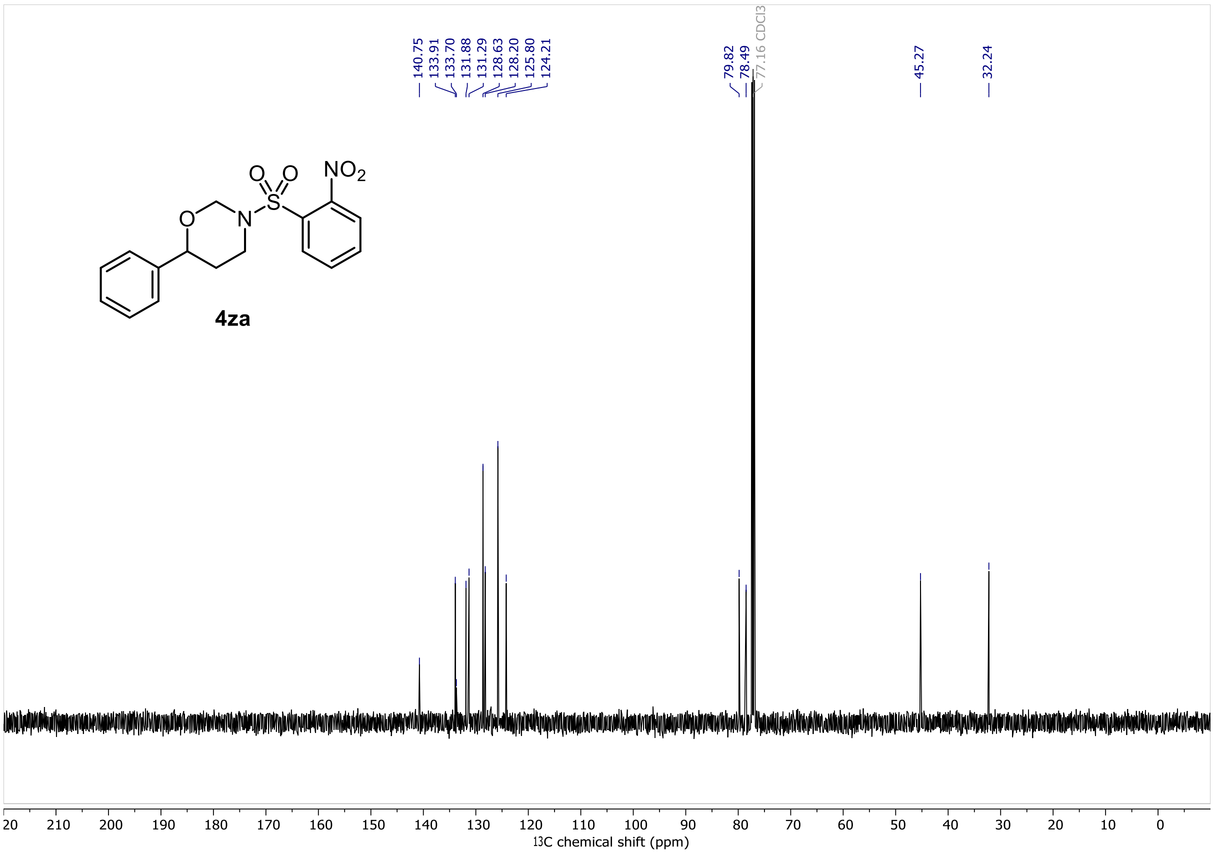

NMR spectra for compound **4za**: $^1$H (501 MHz) and $^{13}$C (126 MHz), in $CDCl_3$.

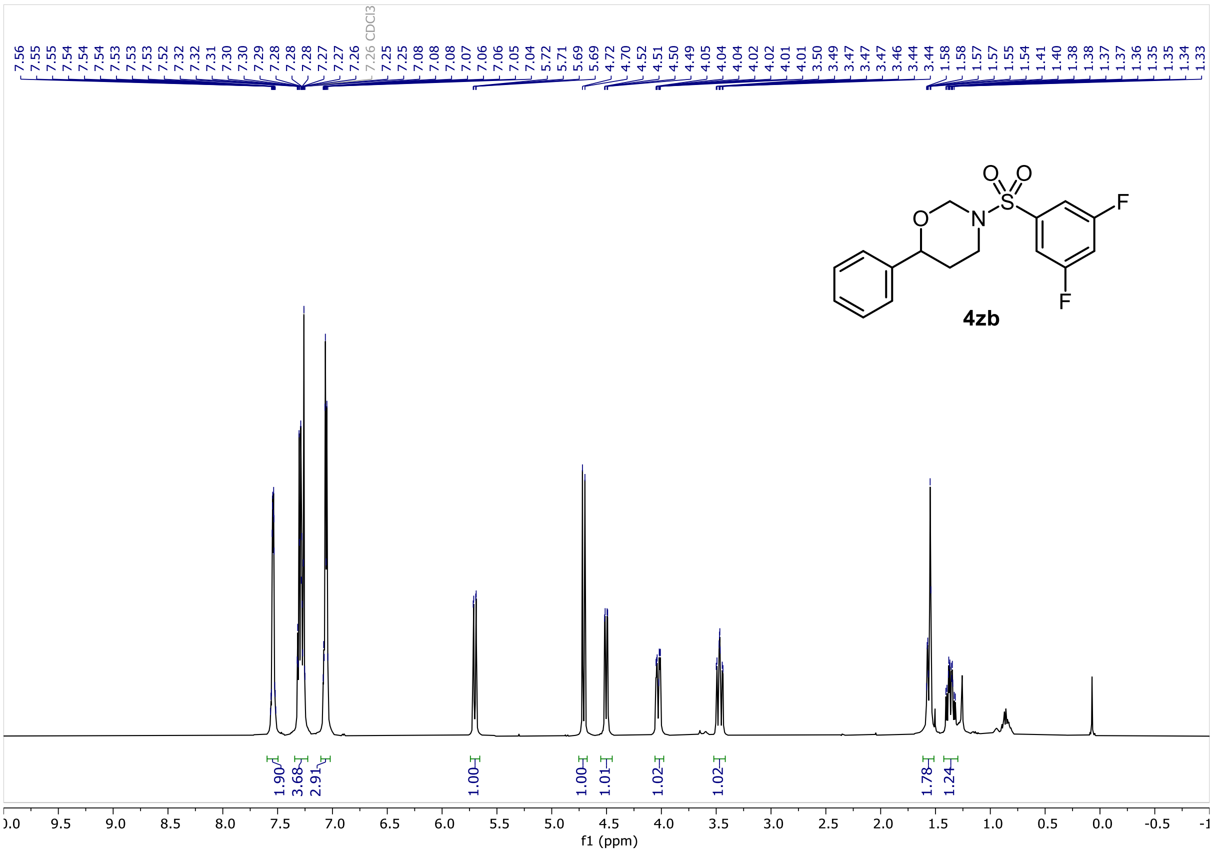

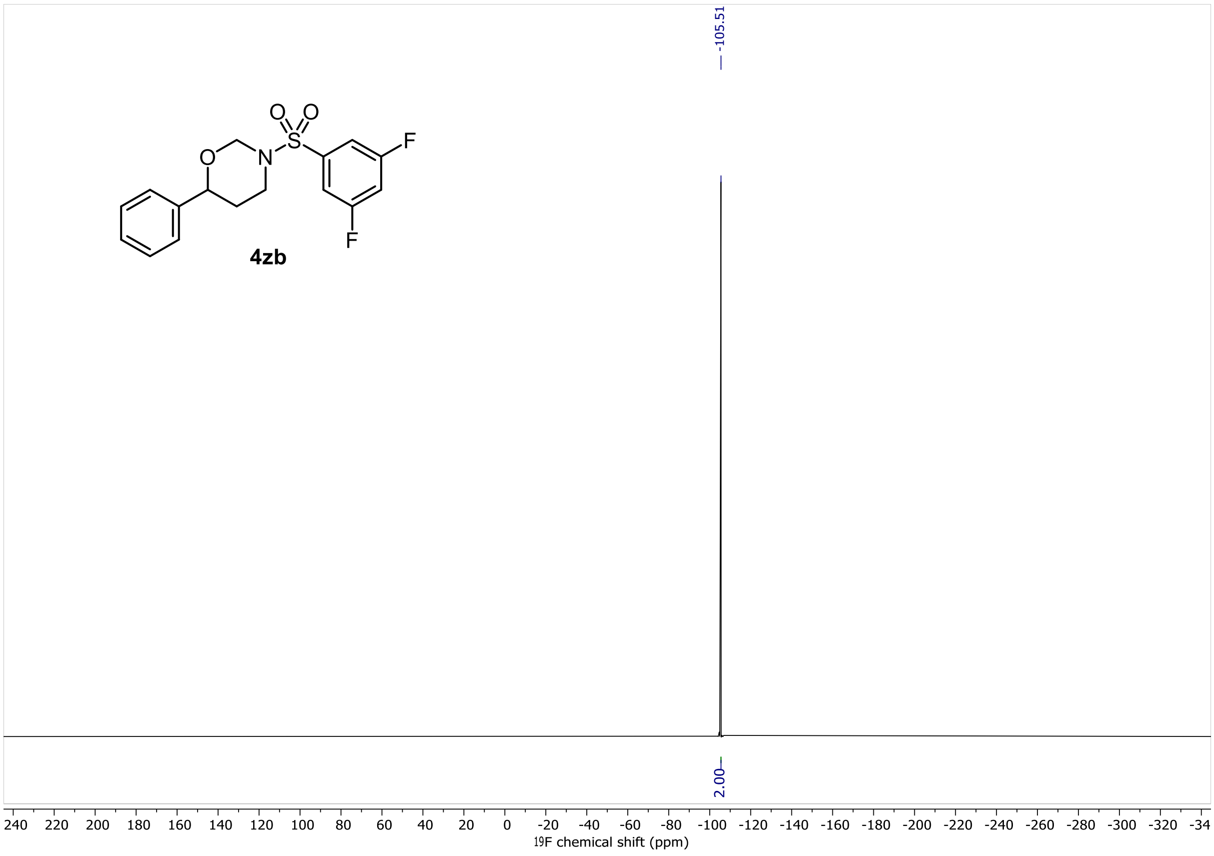

NMR spectra for compound **4zb**: $^{1}H$ (501 MHz) and $^{19}F$ (471 MHz), in $CDCl_3$.

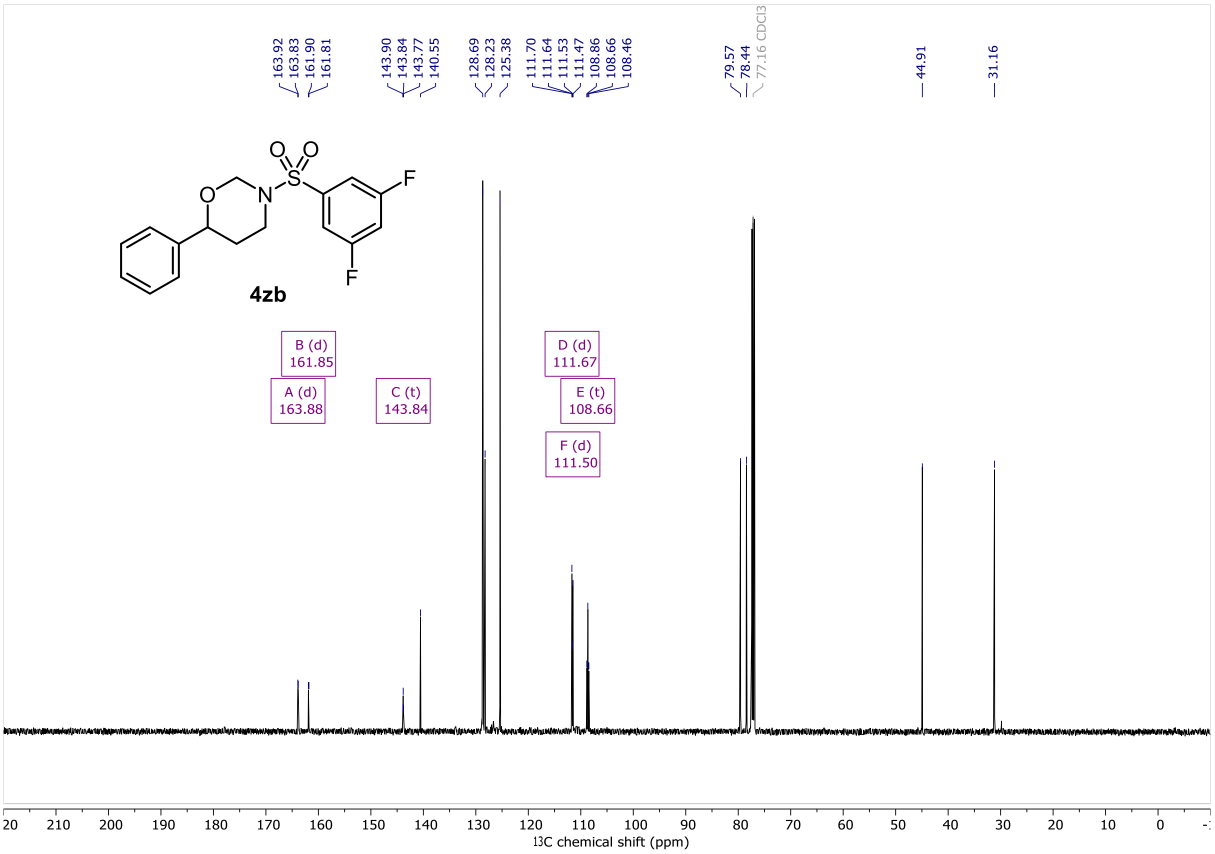


NMR spectra for compound **4zb** *(continuation)*: $^{13}C$ (126 MHz), in $CDCl_3$.

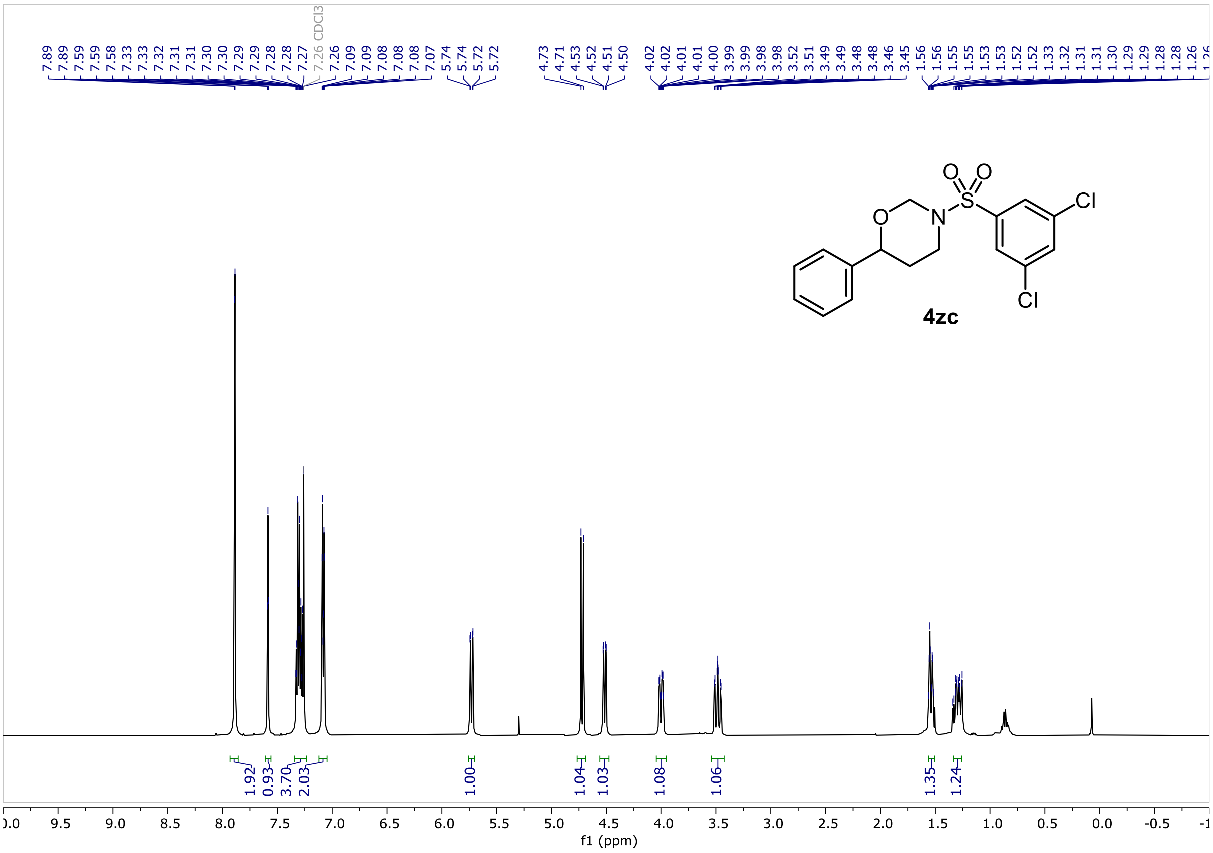


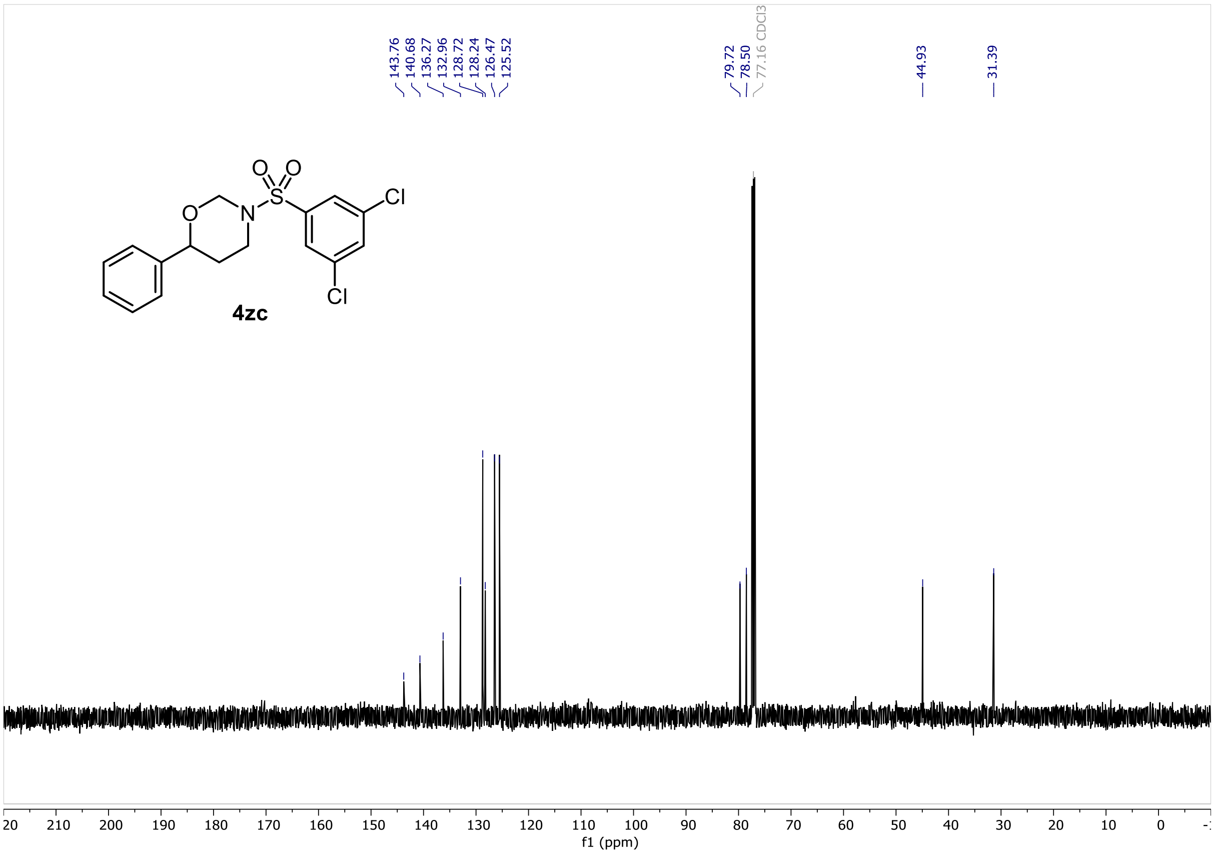


NMR spectra for compound **4zc**: $^{1}H$ (501 MHz) and $^{13}C$ (126 MHz), in $CDCl_3$.

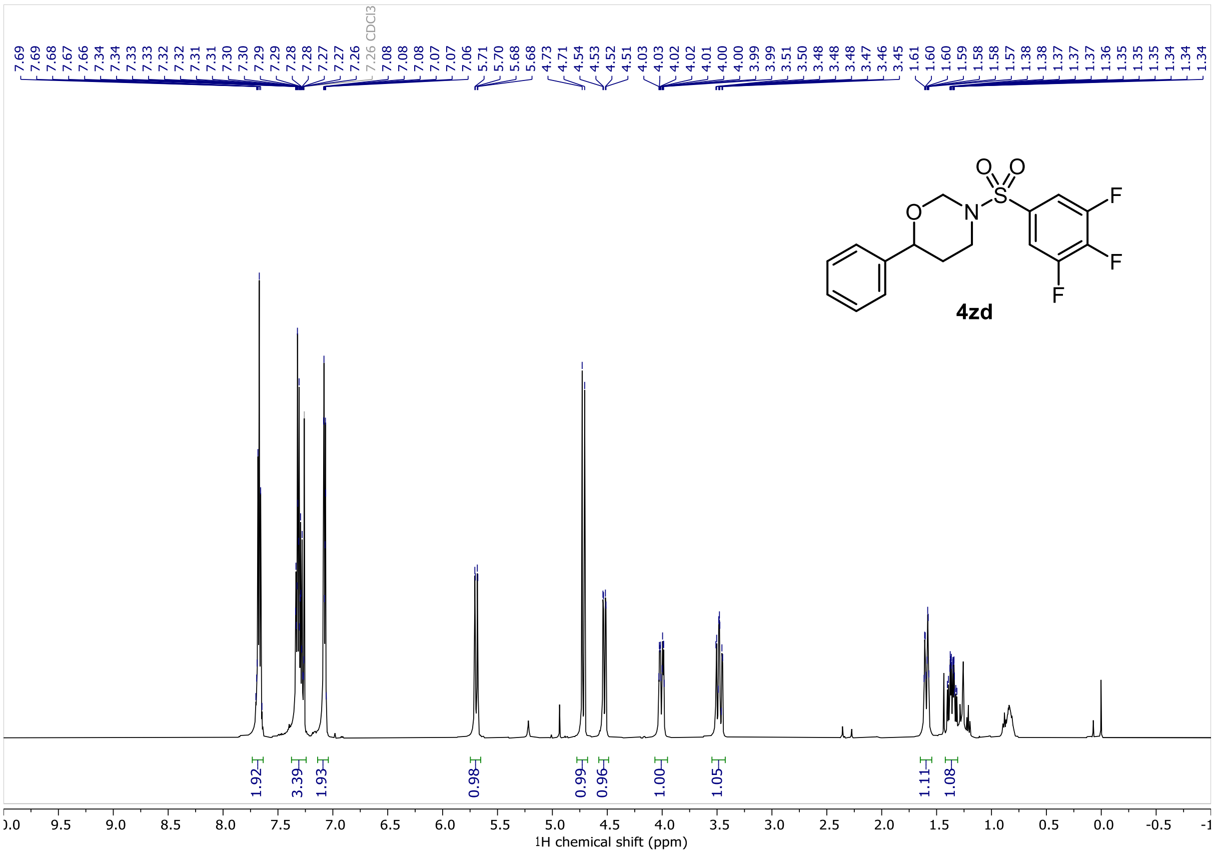


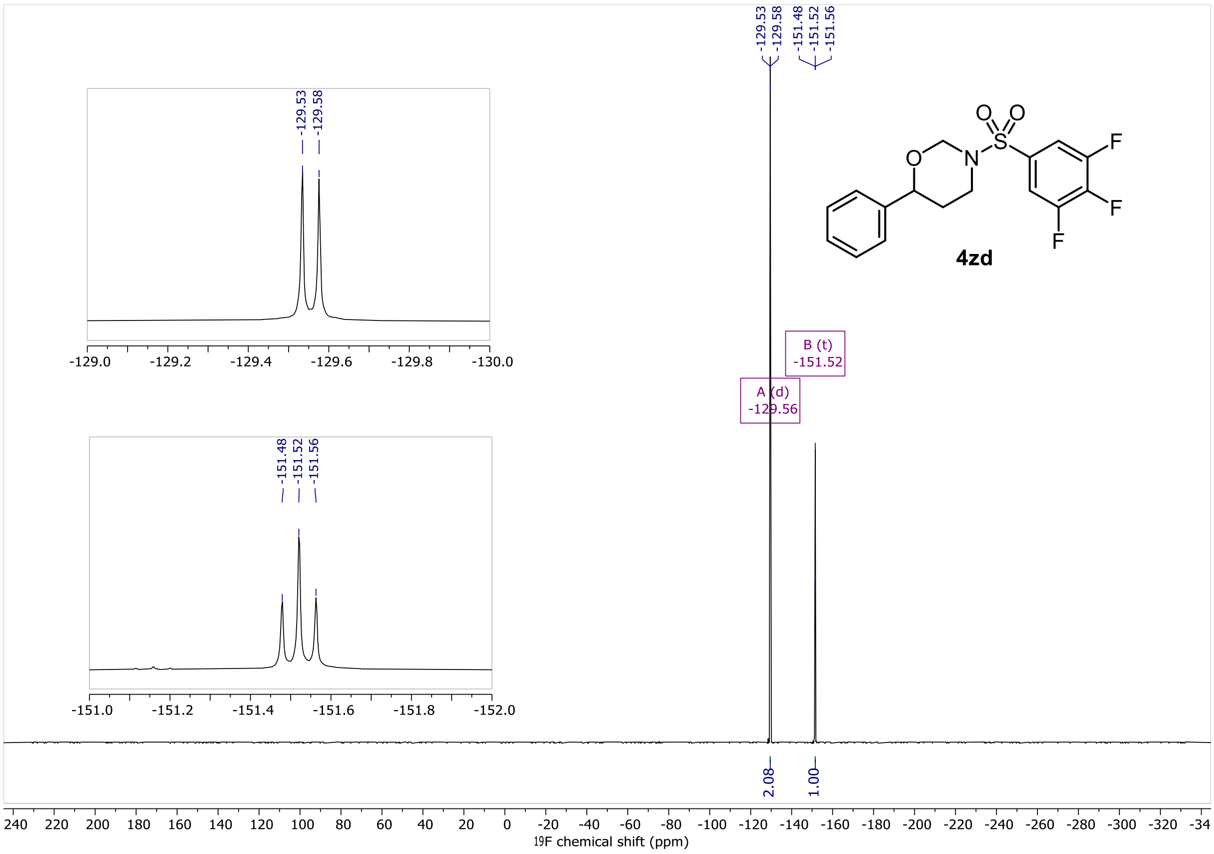


NMR spectra for compound **4zd**: $^{1}H$ (501 MHz) and $^{19}F$ (471 MHz), in $CDCl_3$.

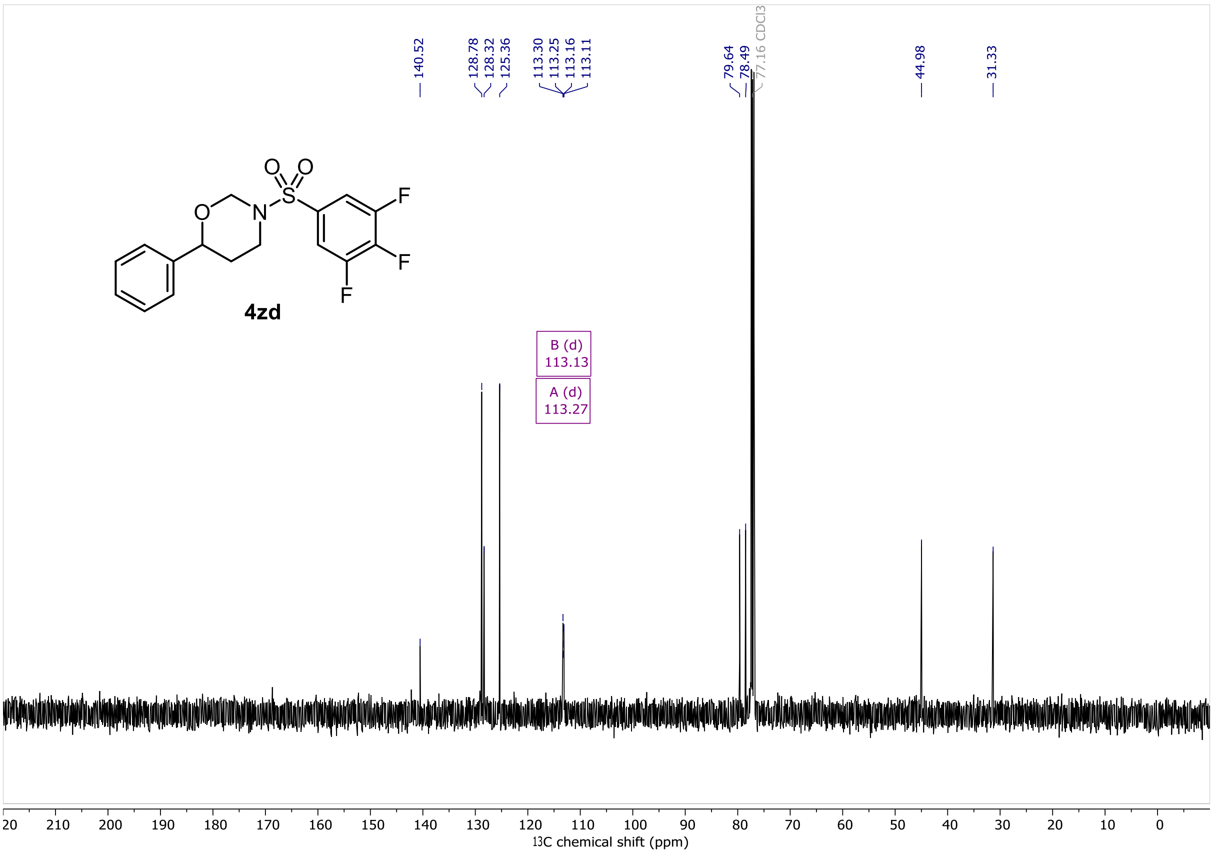


NMR spectra for compound **4zd** *(continuation)*: $^{13}$C (126 MHz), in $CDCl_3$.

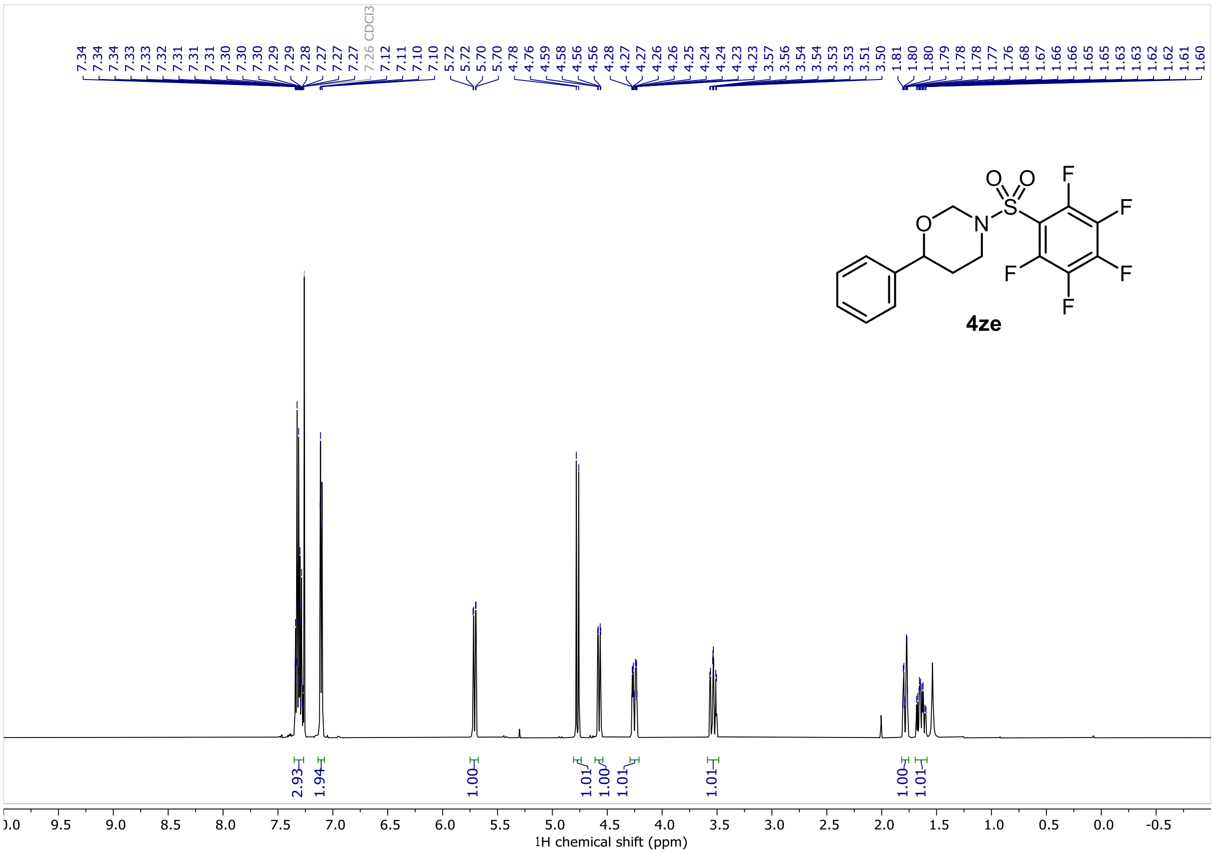


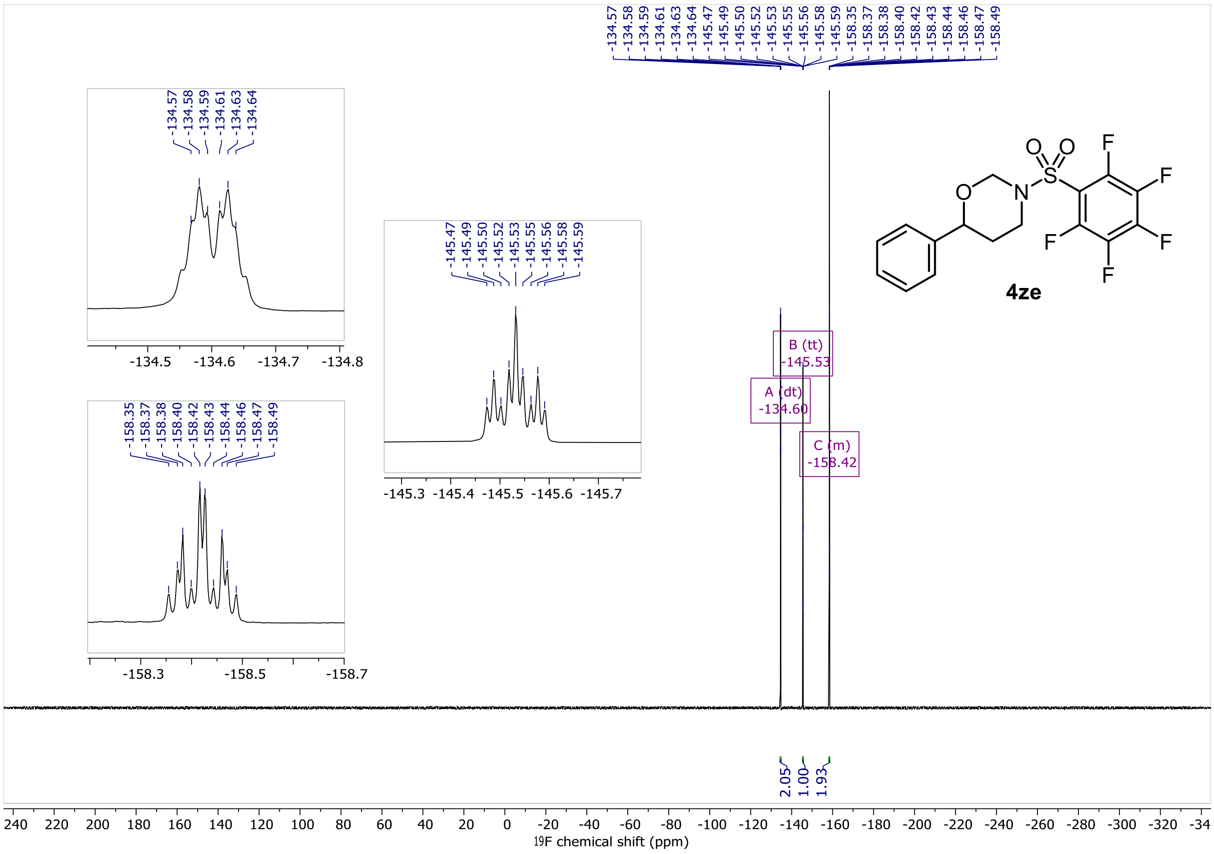


NMR spectra for compound **4ze**: $^{1}H$ (501 MHz) and $^{19}F$ (471 MHz), in $CDCl_3$.

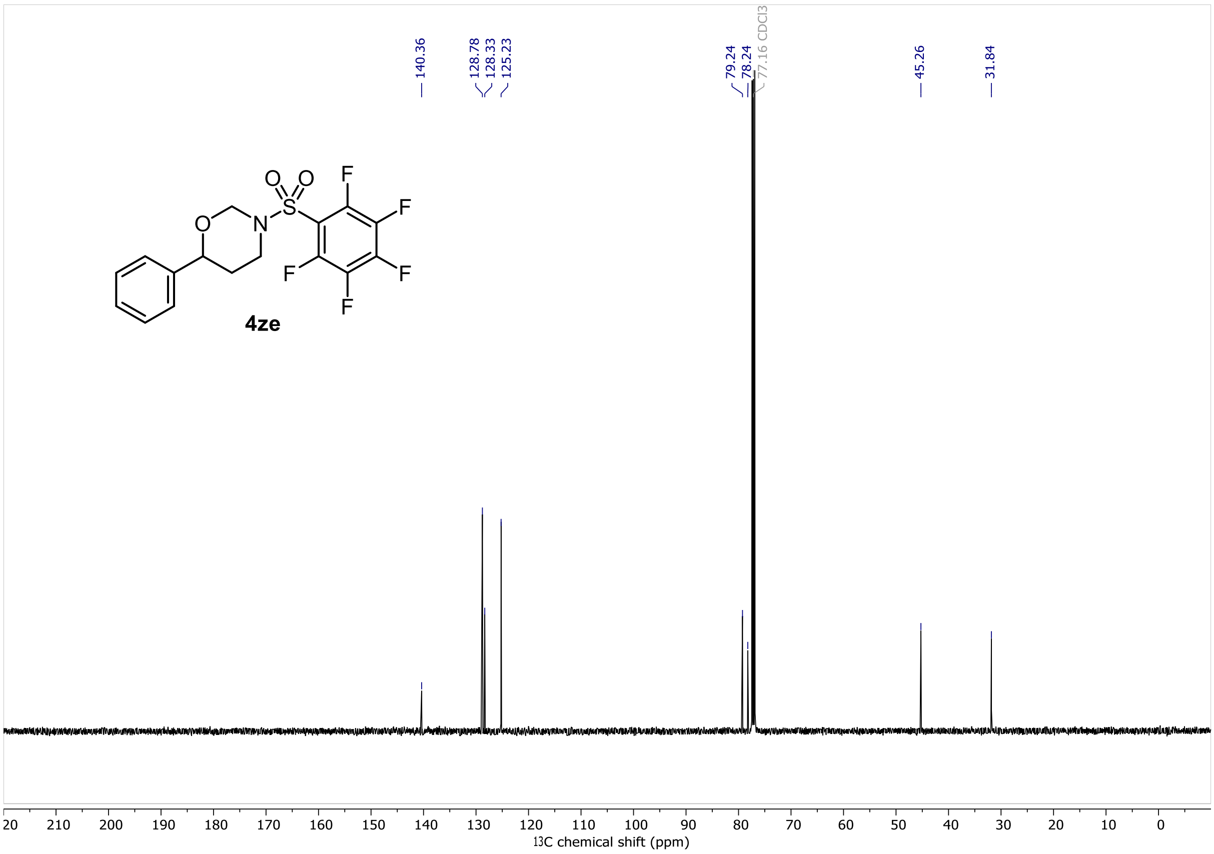


NMR spectra for compound **4ze** *(continuation)*: $^{13}C$ (126 MHz), in $CDCl_3$.

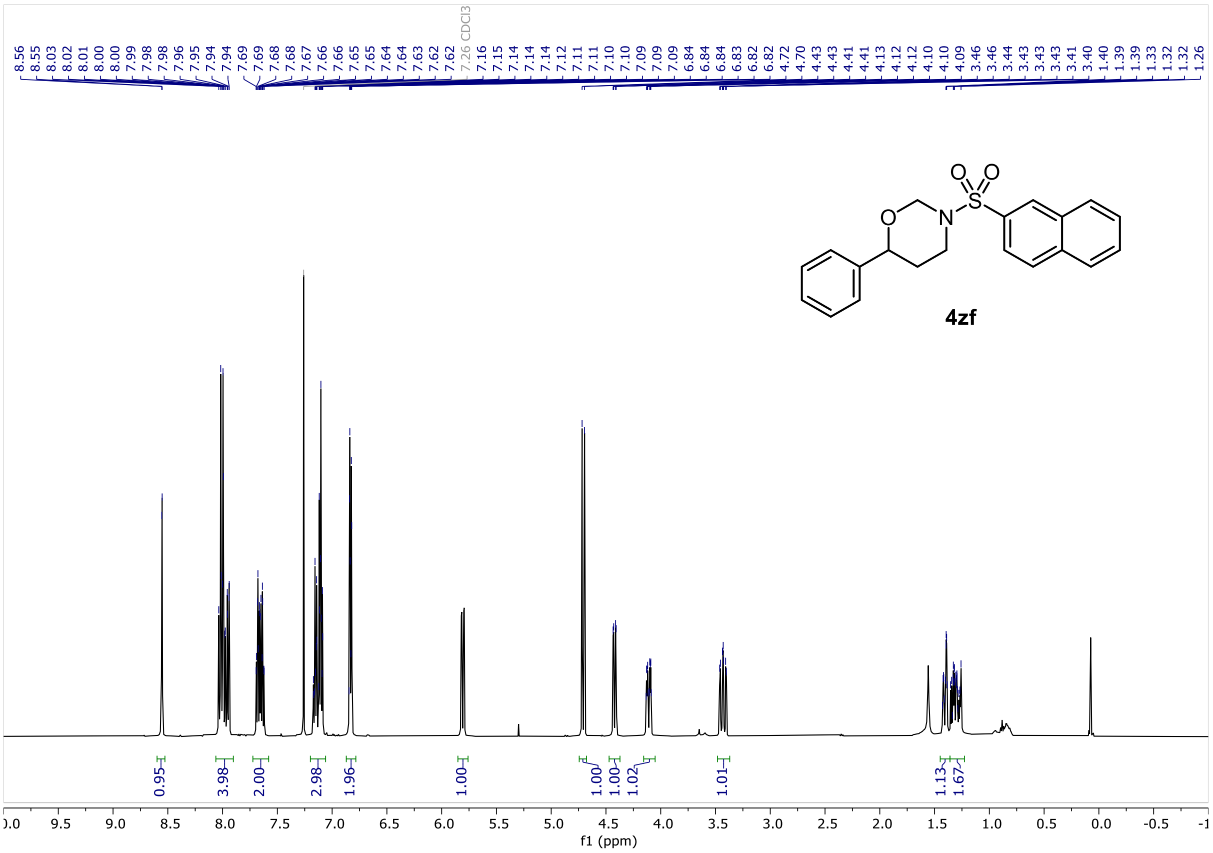

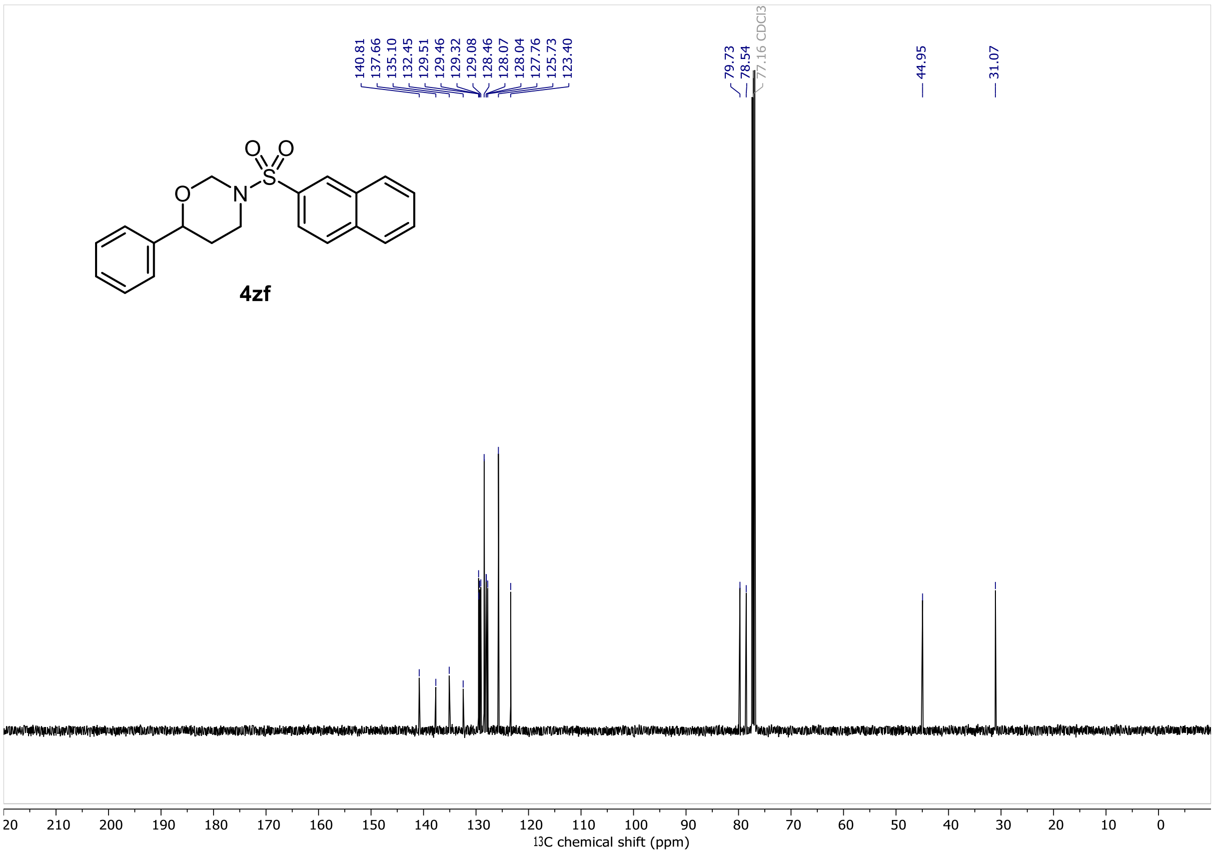

NMR spectra for compound **4zf**: $^{1}H$ (501 MHz) and $^{13}C$ (126 MHz), in $CDCl_3$.

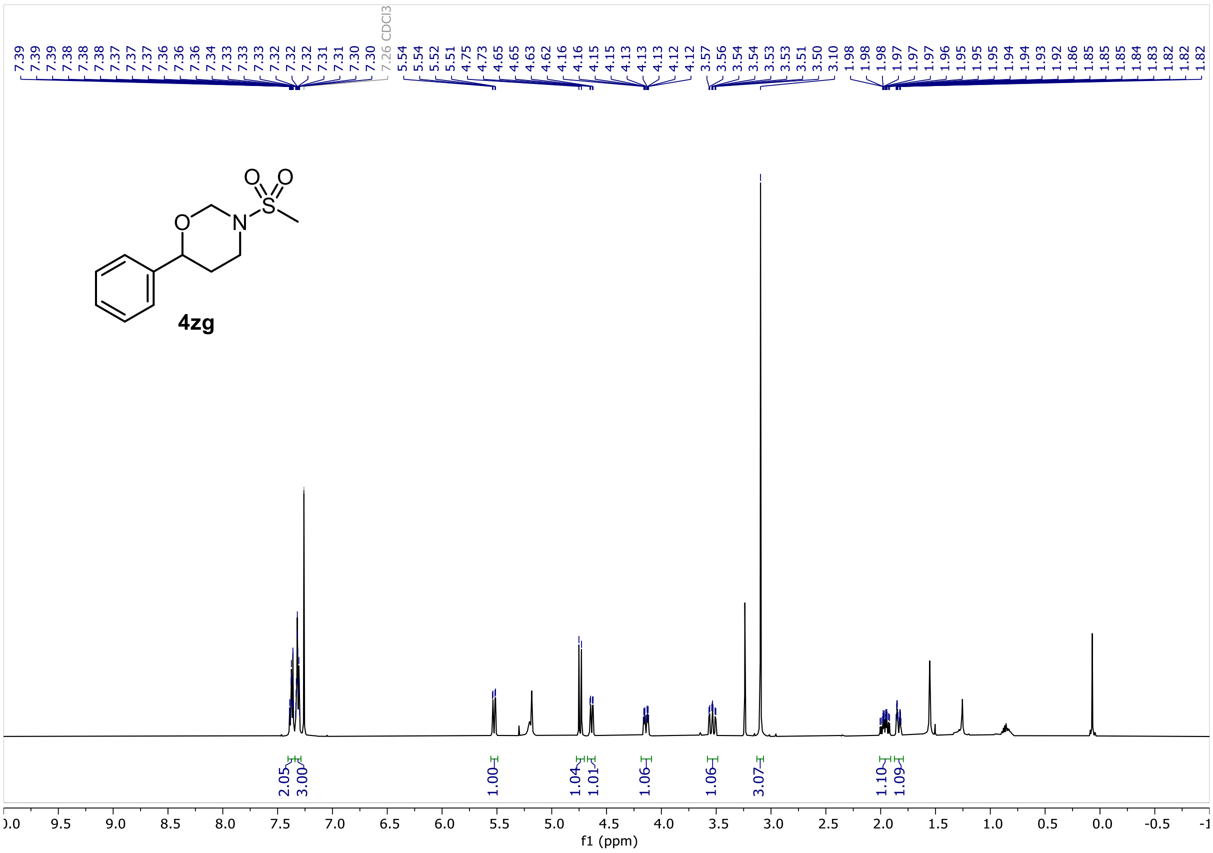

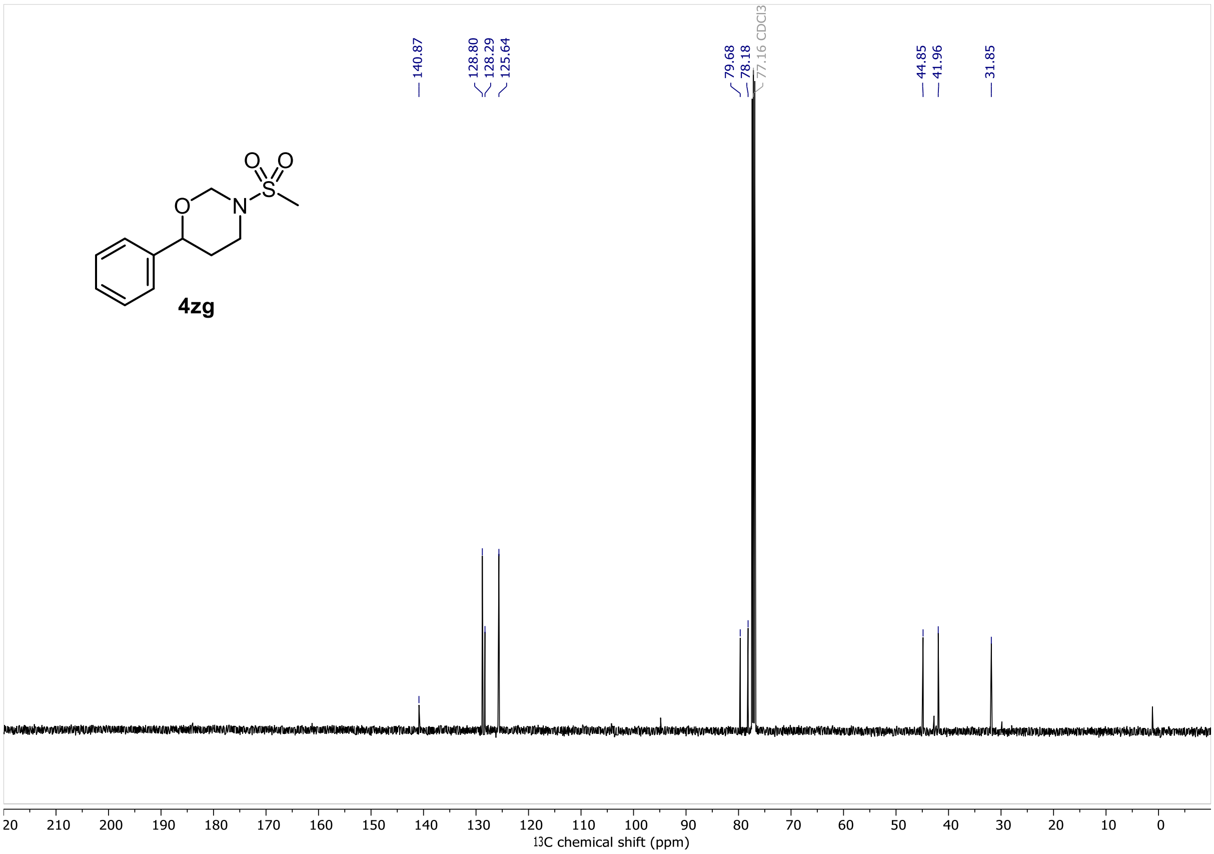

NMR spectra for compound **4zg**: $^{1}H$ (501 MHz) and $^{13}C$ (126 MHz), in $CDCl_3$.

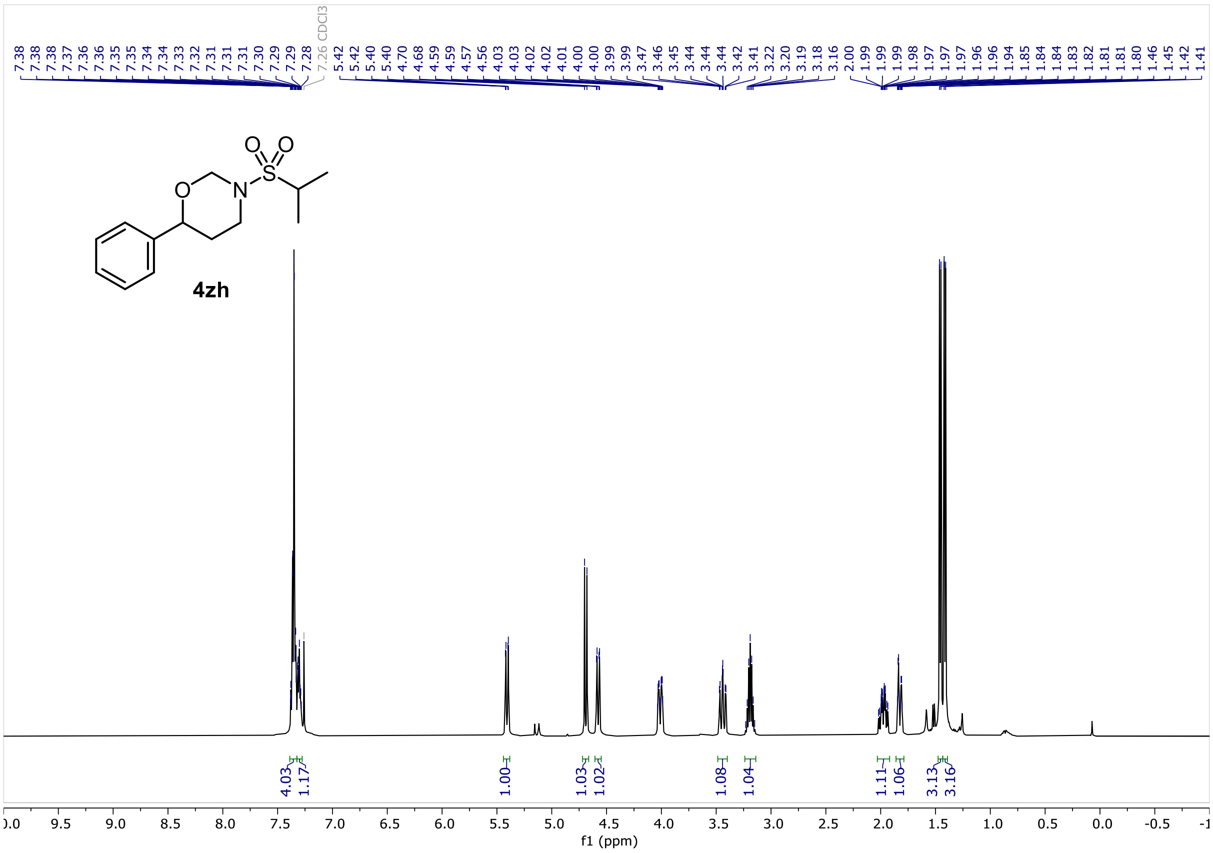


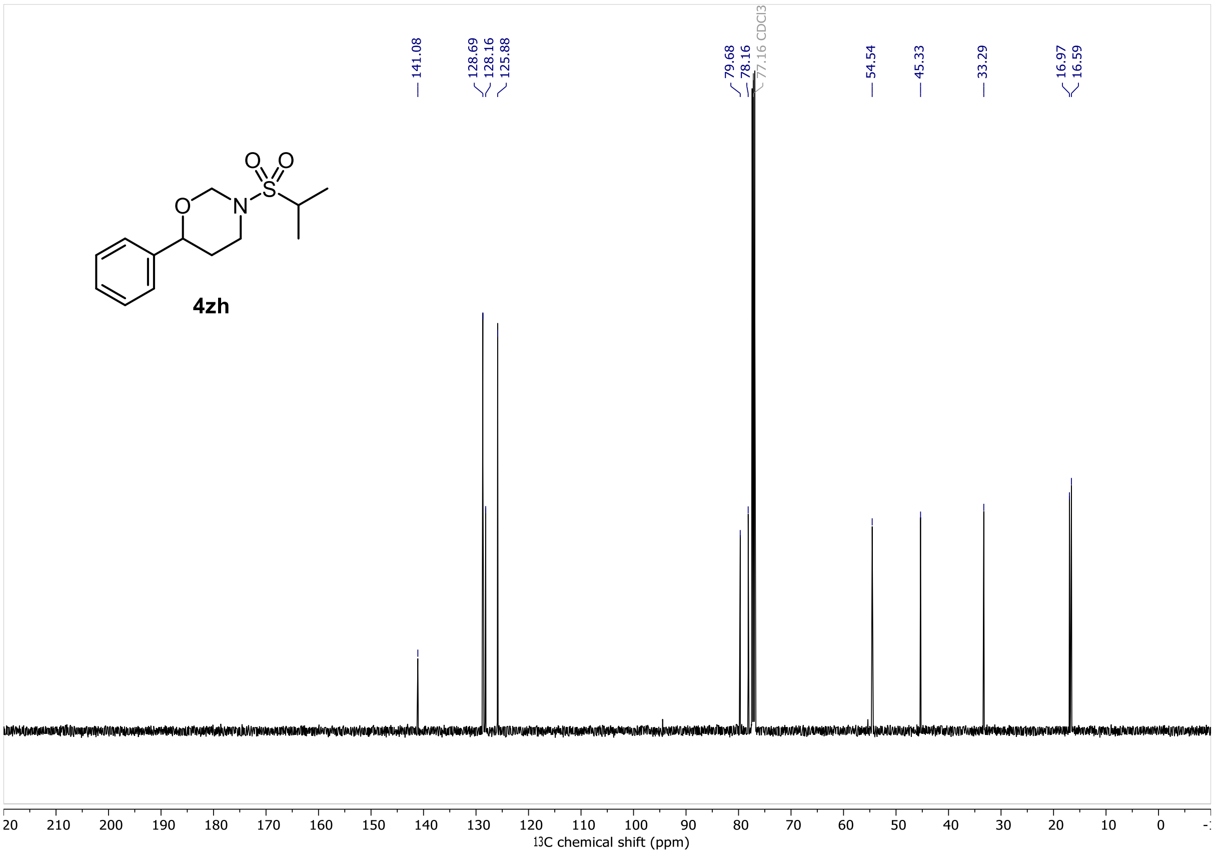


NMR spectra for compound **4zh**: $^{1}H$ (501 MHz) and $^{13}C$ (126 MHz), in $CDCl_3$.

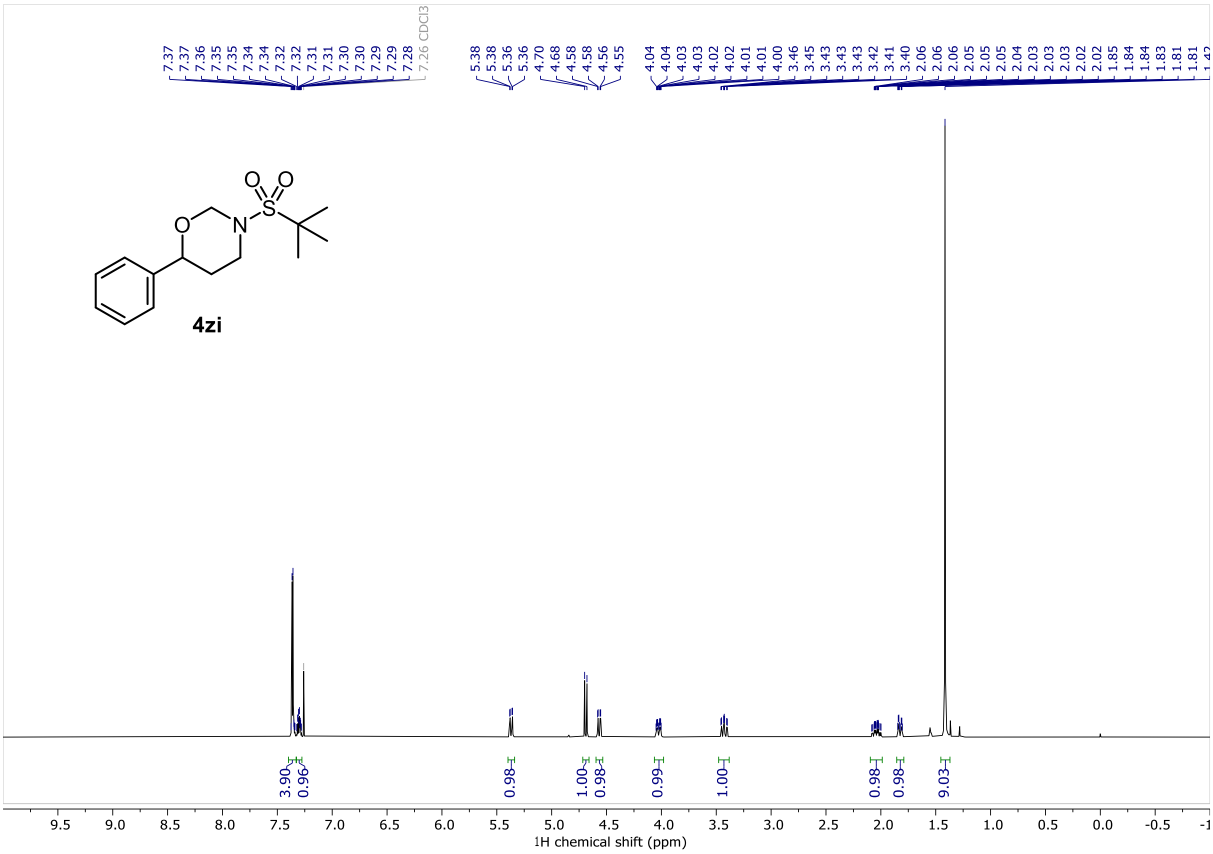


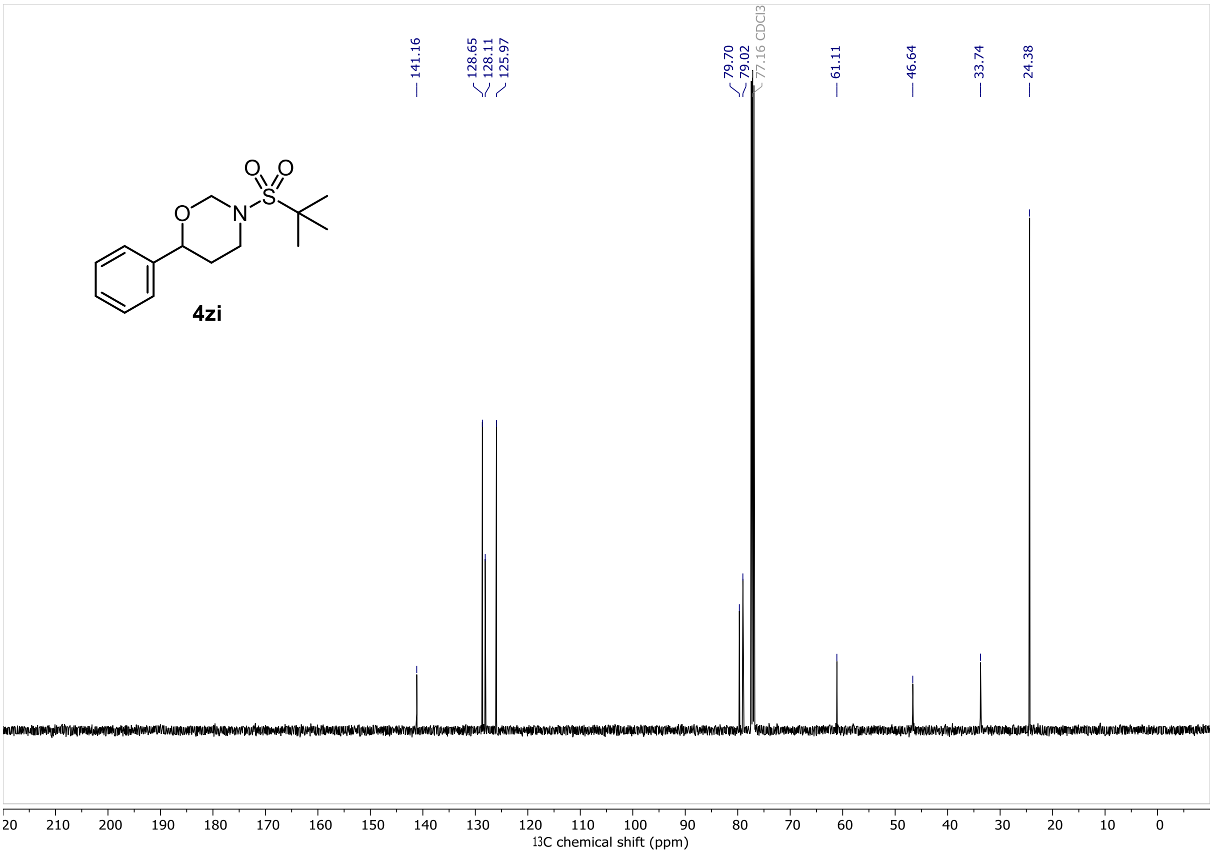


NMR spectra for compound **4zi**: $^{1}H$ (501 MHz) and $^{13}C$ (126 MHz), in $CDCl_3$.

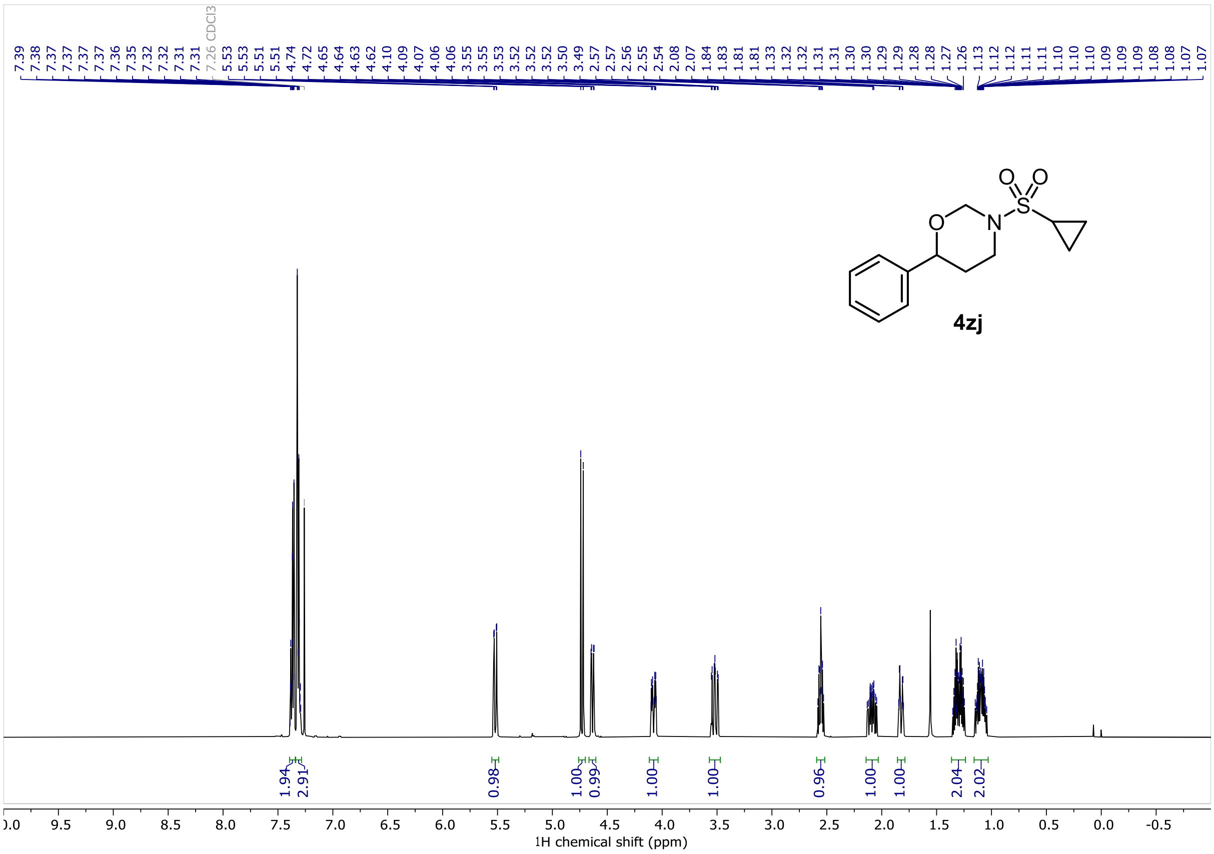


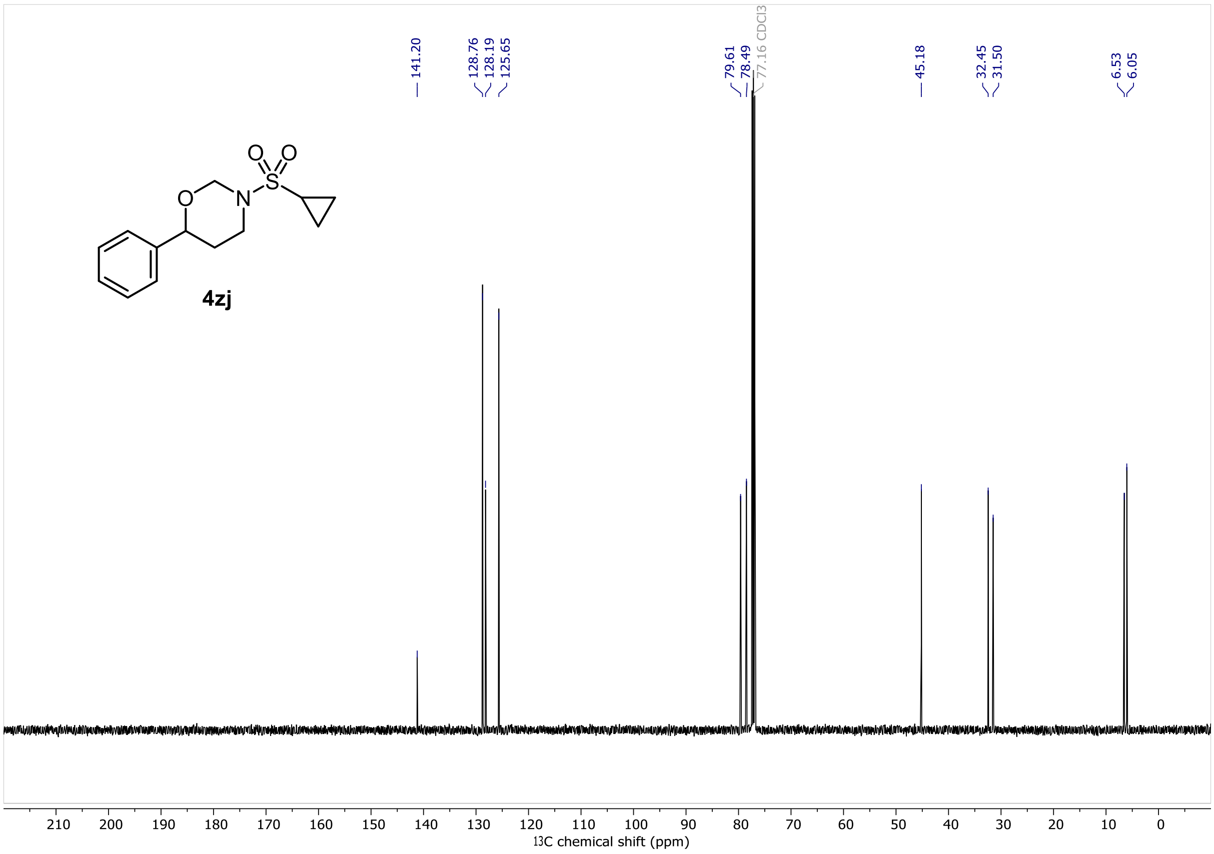


NMR spectra for compound **4zj**: $^{1}H$ (501 MHz) and $^{13}C$ (126 MHz), in $CDCl_3$.

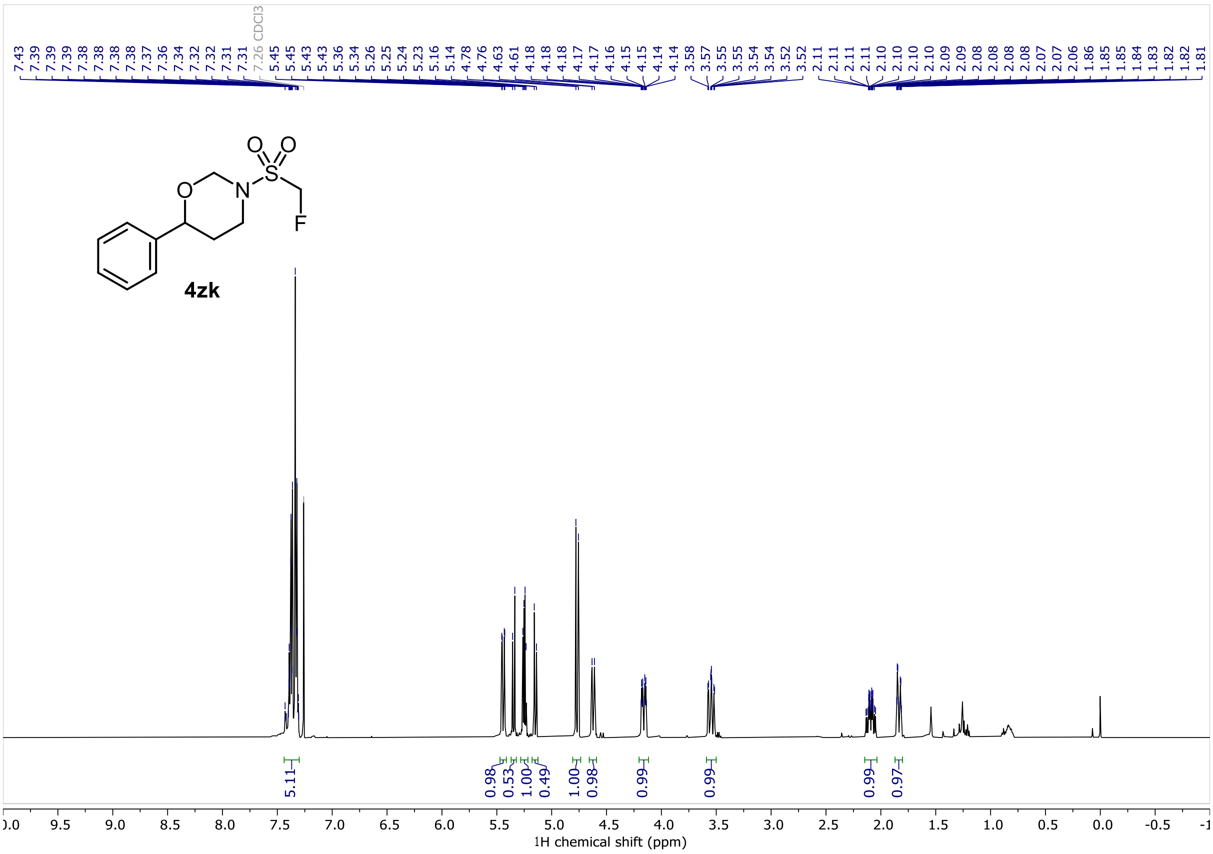


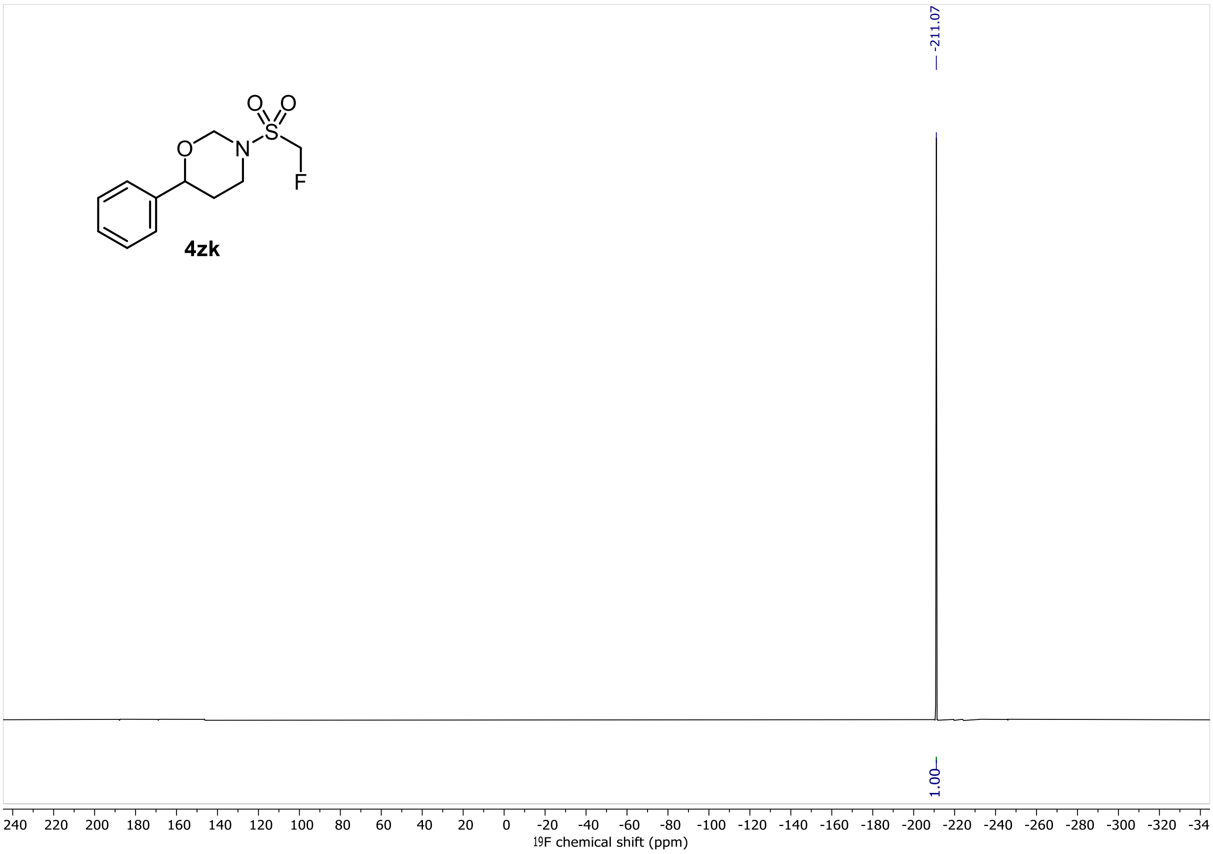


NMR spectra for compound **4zk**: $^{1}H$ (501 MHz) and $^{19}F$ (471 MHz), in $CDCl_3$.

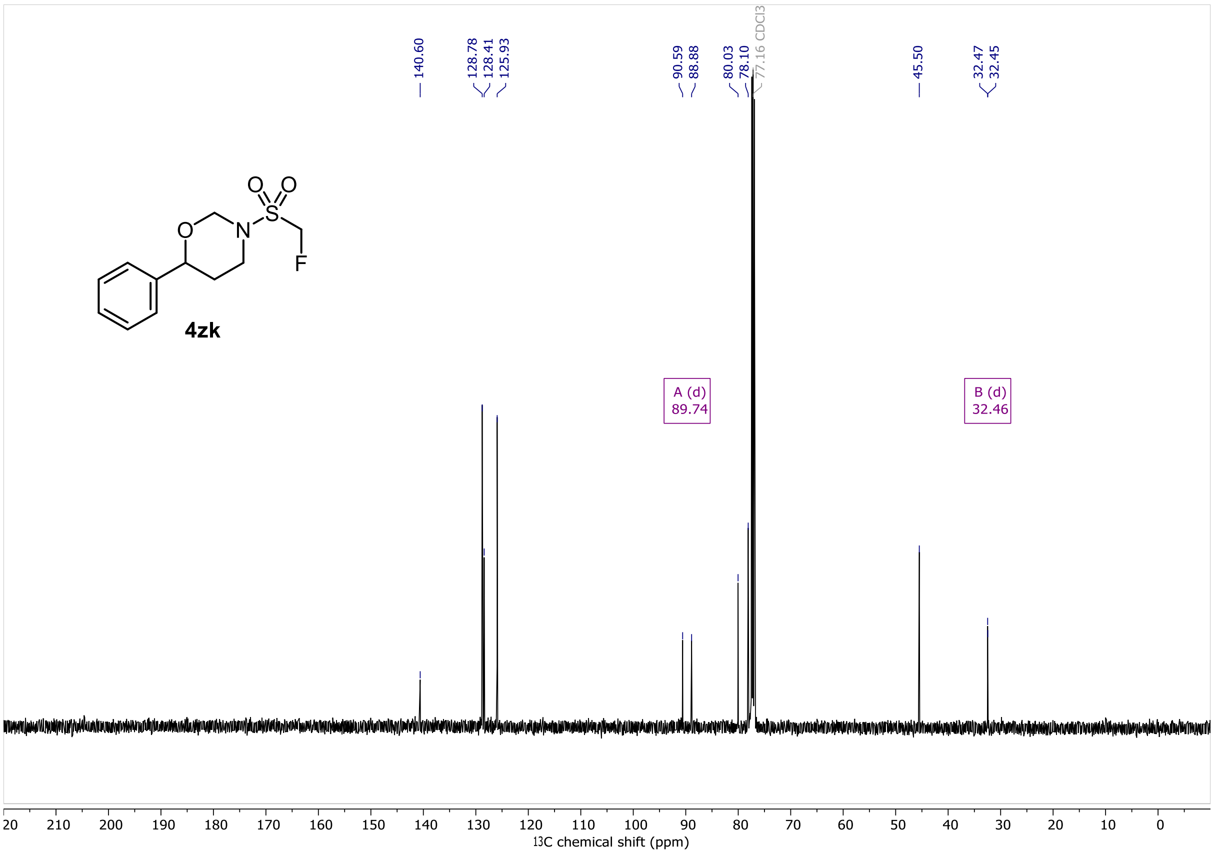


NMR spectra for compound **4zk** *(continuation)*: $^{13}C$ (126 MHz), in $CDCl_3$.

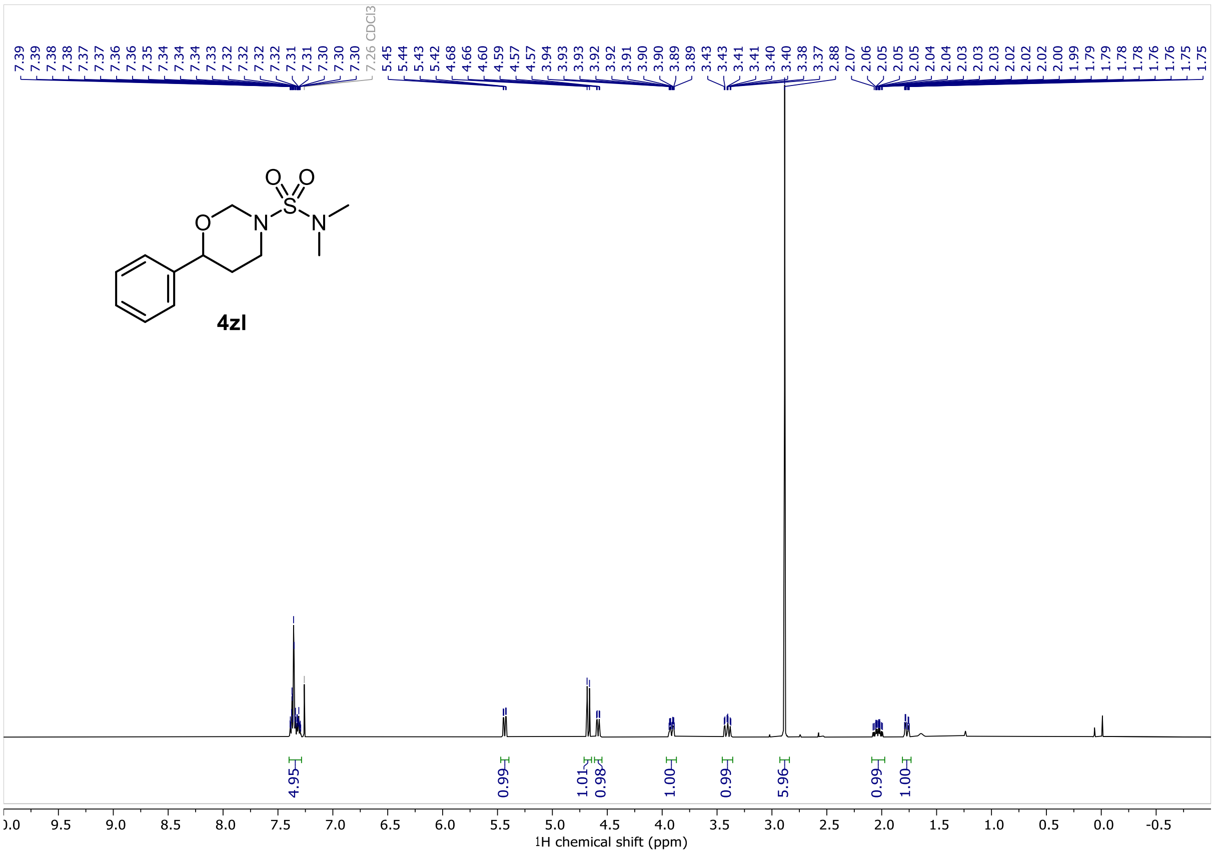


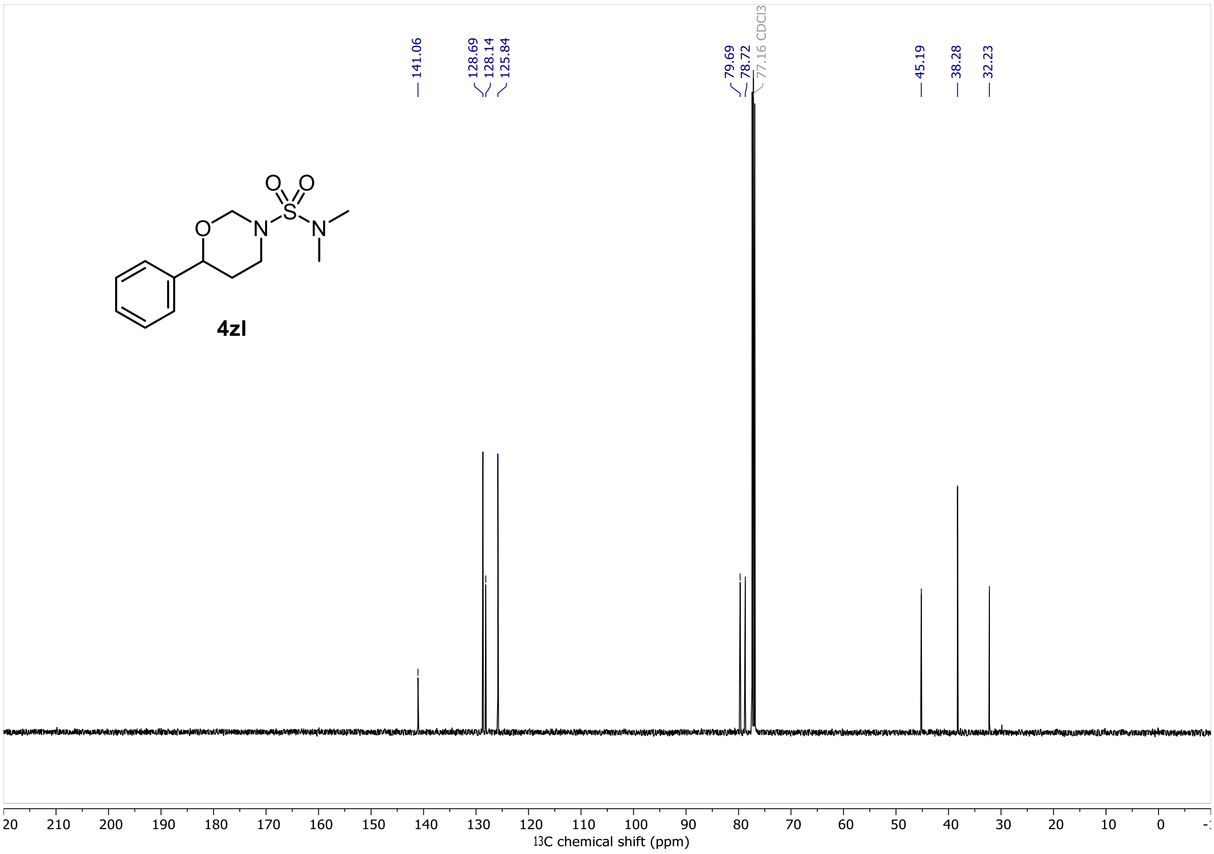


NMR spectra for compound **4zl**: $^{1}H$ (501 MHz) and $^{13}C$ (126 MHz), in $CDCl_3$.

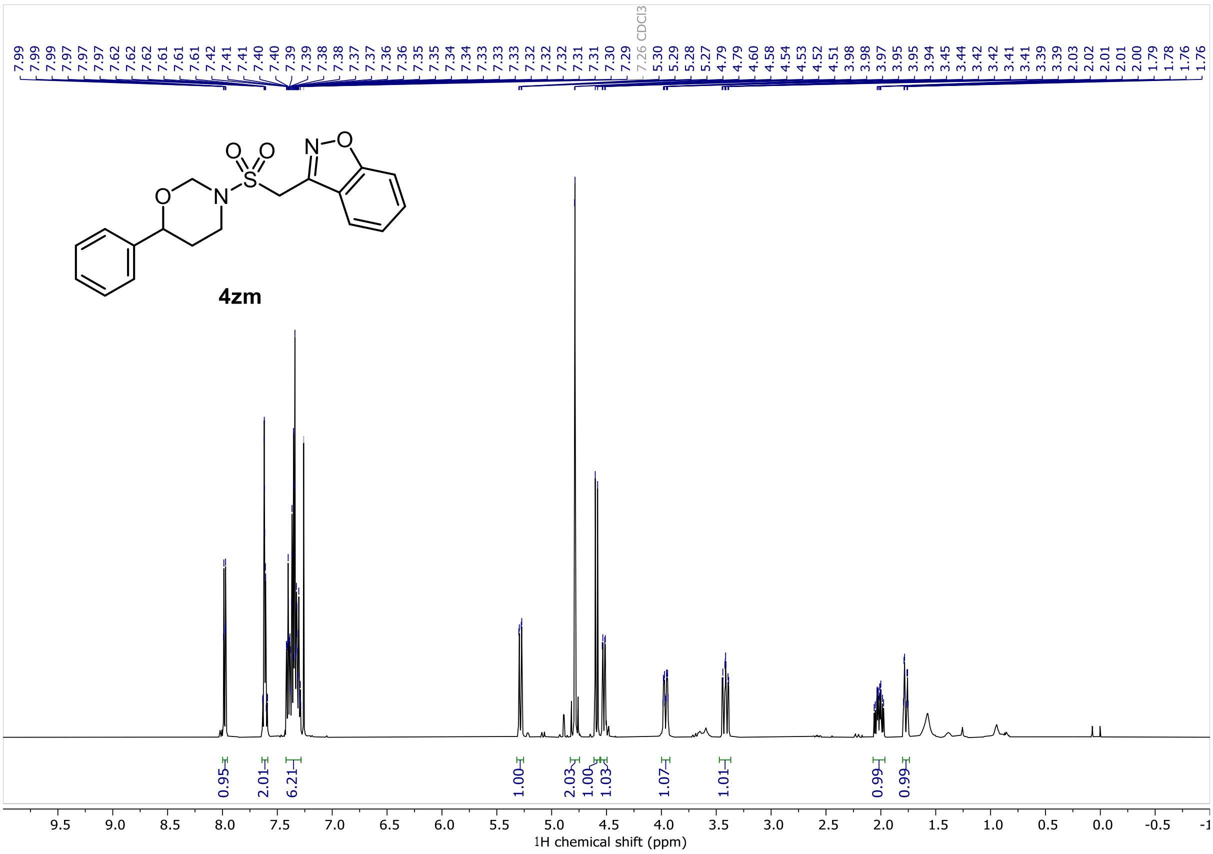


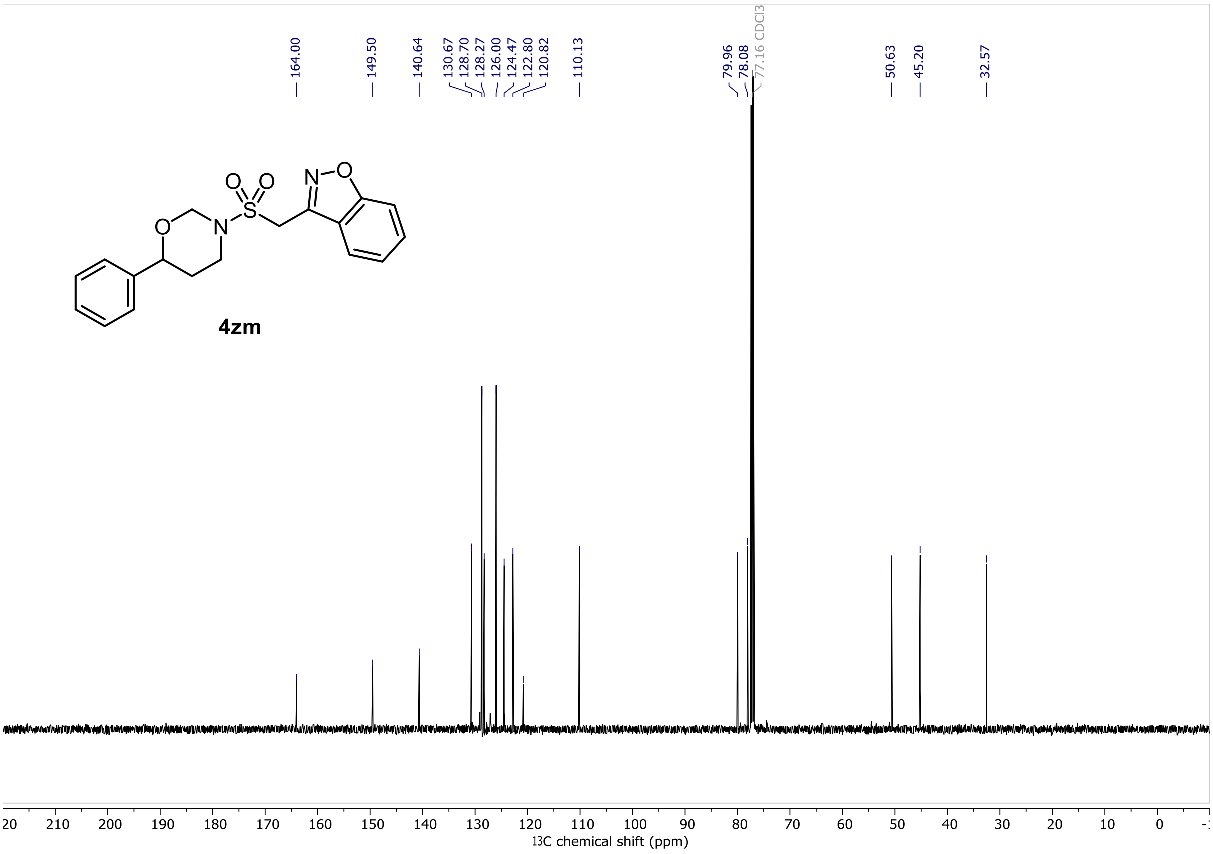


NMR spectra for compound **4zm**: $^{1}H$ (501 MHz) and $^{13}C$ (126 MHz), in $CDCl_3$.

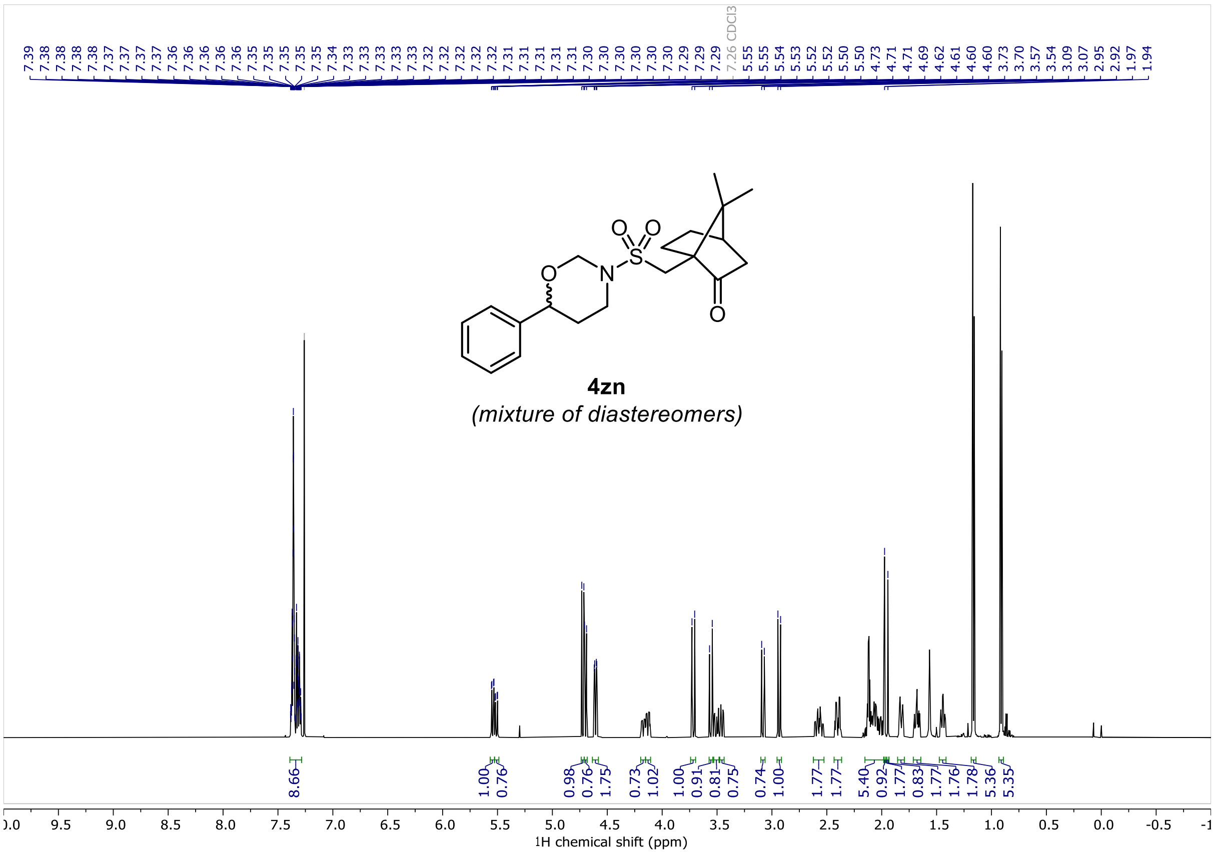

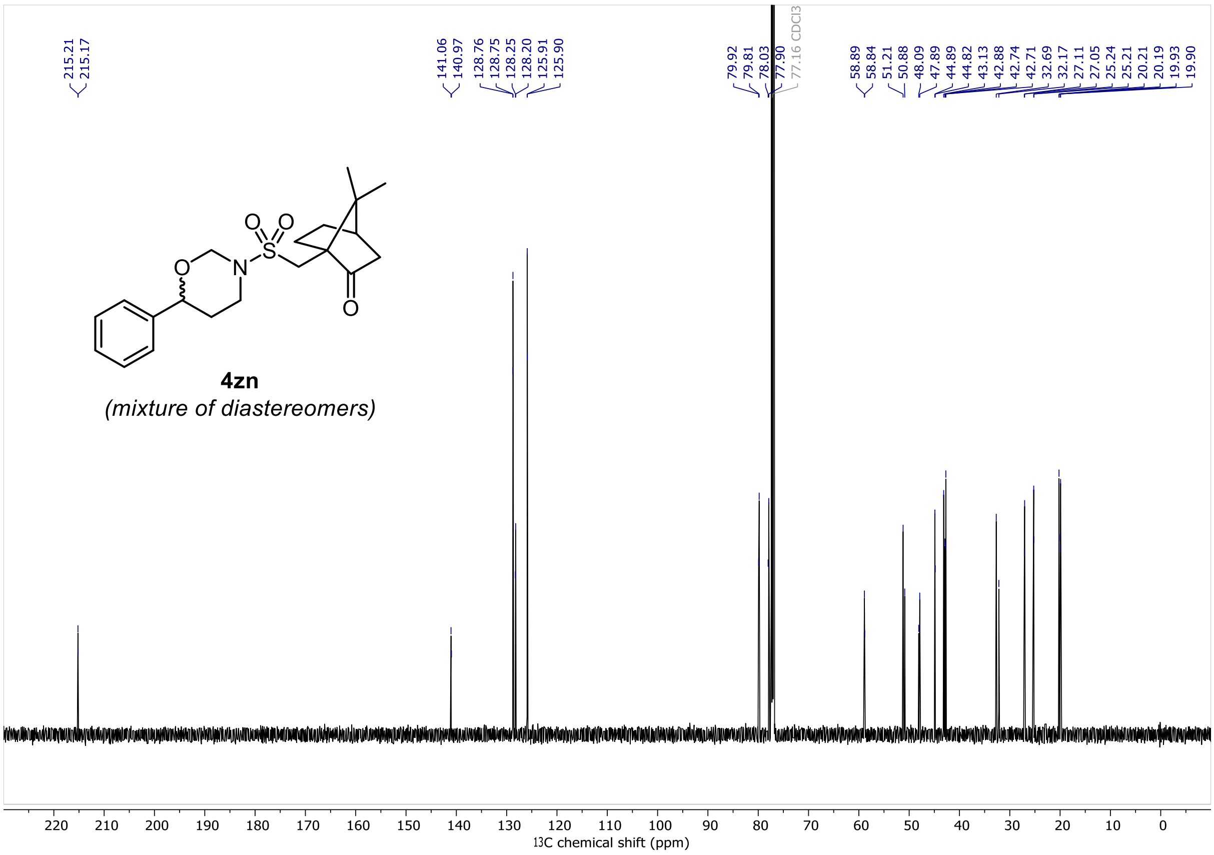

NMR spectra for compound **4zn**: $^{1}H$ (600 MHz) and $^{13}C$ (151 MHz), in $CDCl_3$.

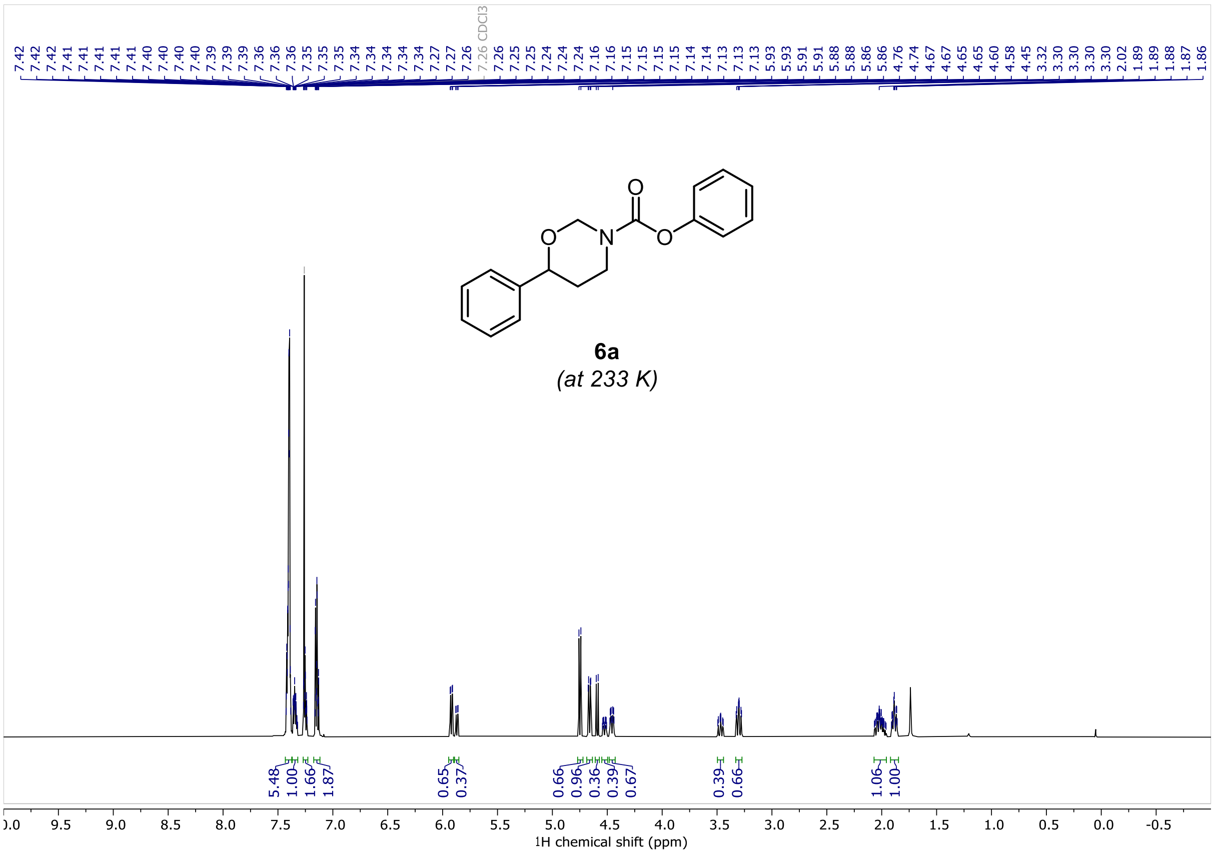


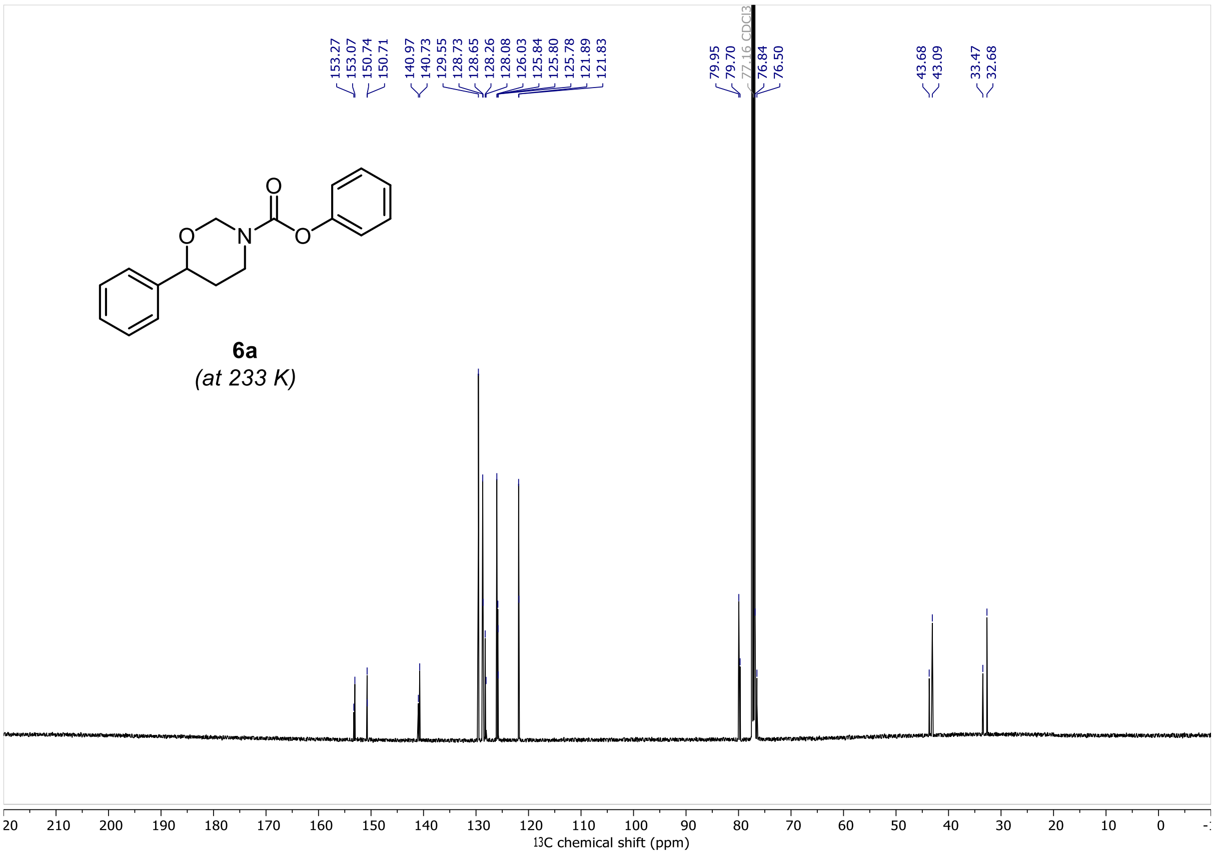


NMR spectra for compound **6a**: $^{1}$H (600 MHz) and $^{13}$C (151 MHz), in $CDCl_3$ at 233 K.

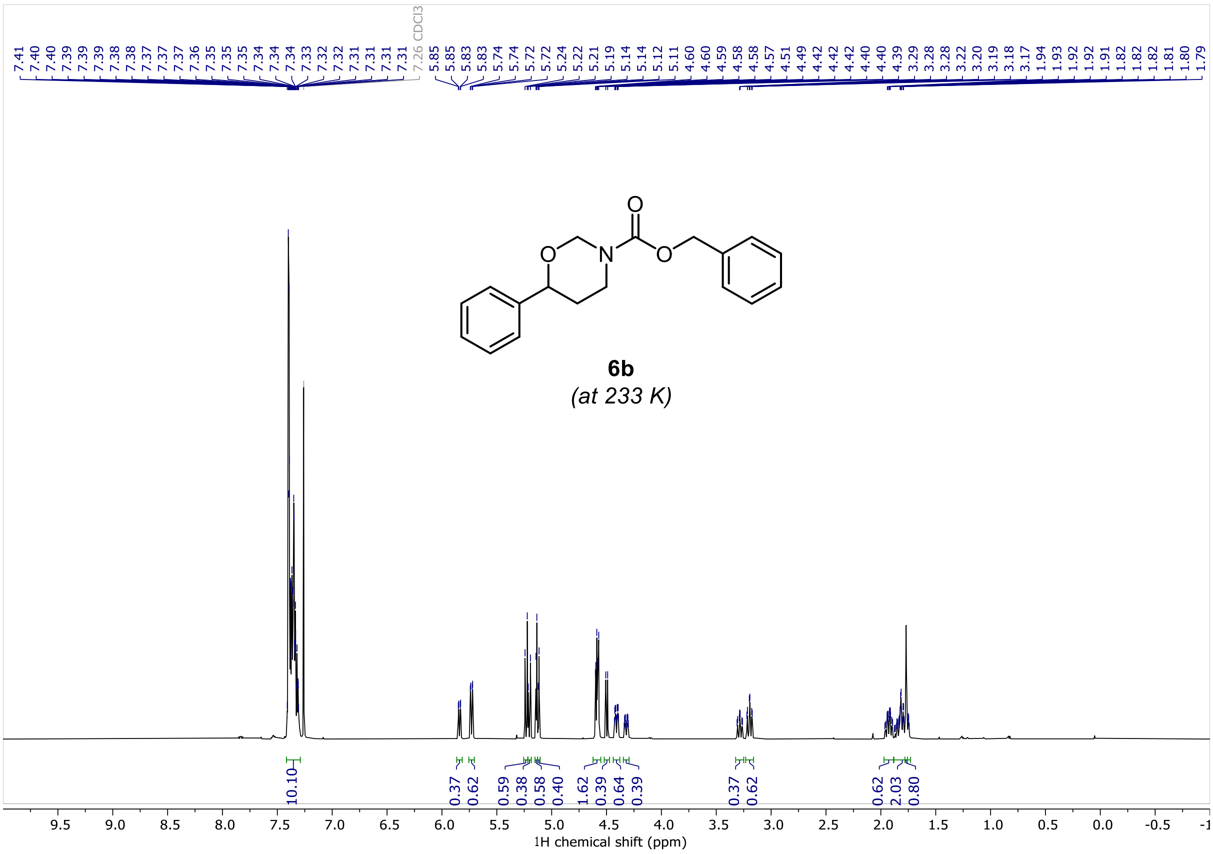

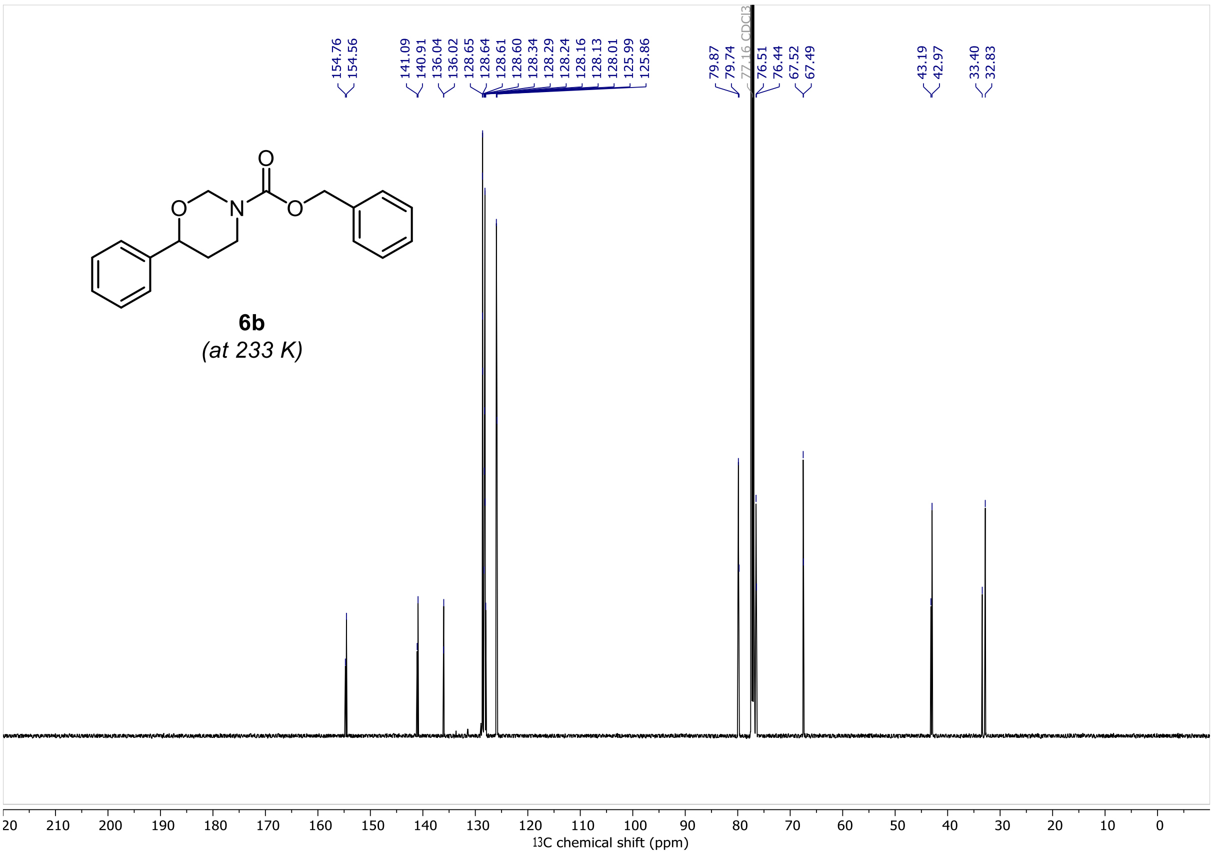

NMR spectra for compound **6b**: $^{1}$H (600 MHz) and $^{13}$C (151 MHz), in $CDCl_3$ at 233 K.

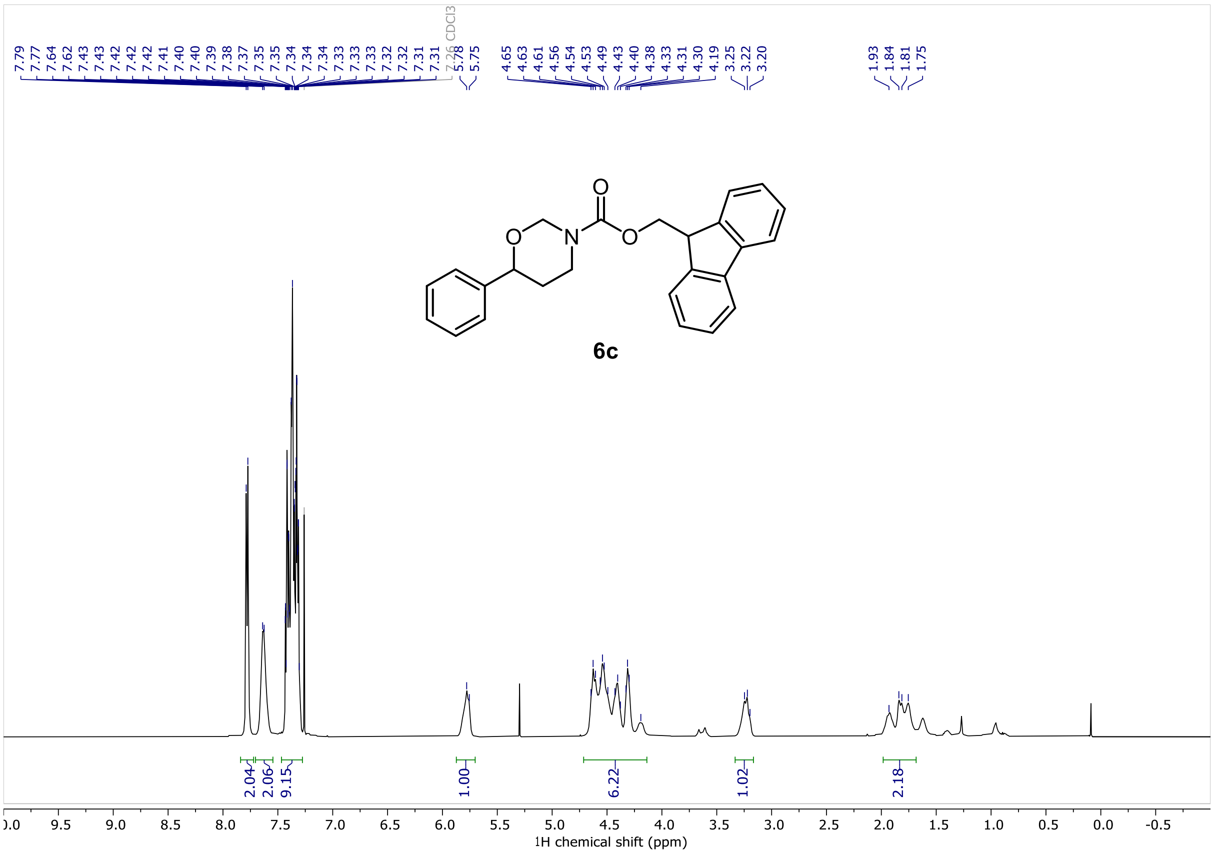


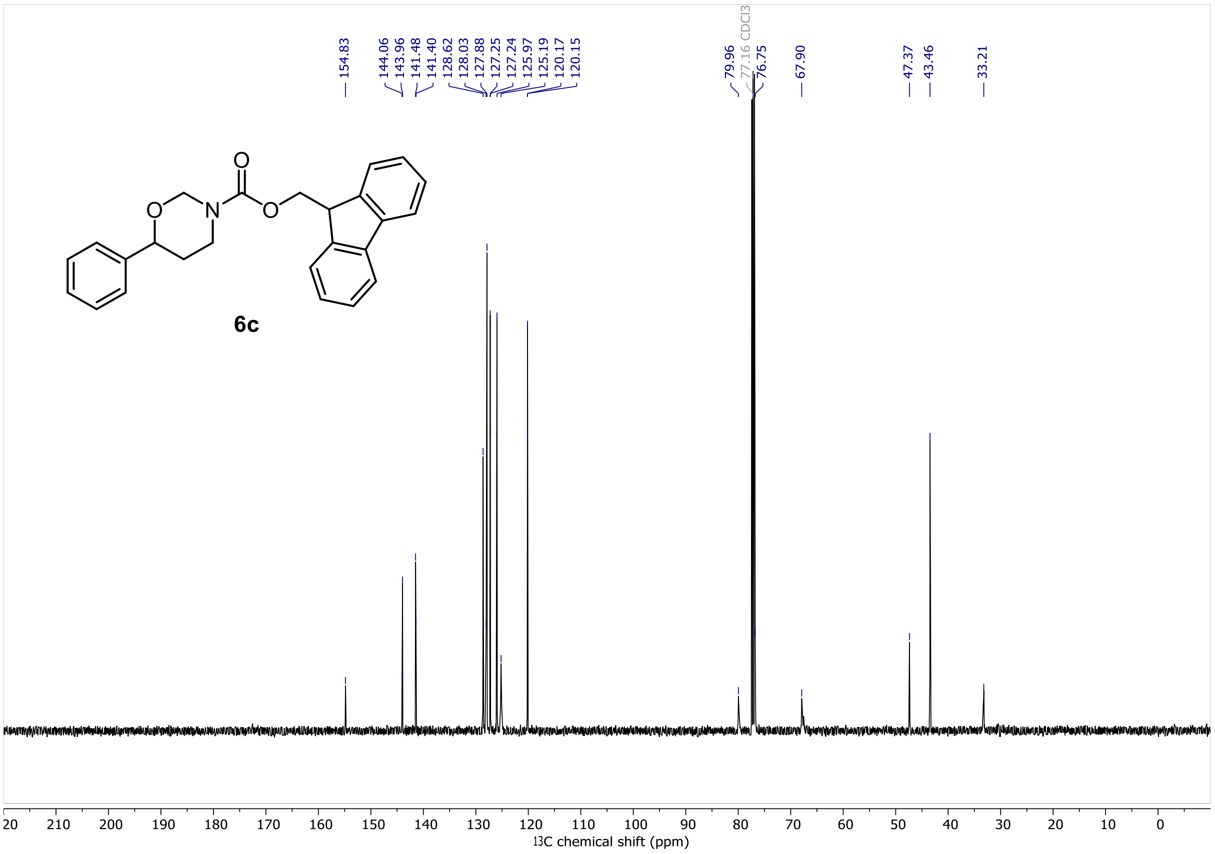


NMR spectra for compound **6c**: $^{1}$H (501 MHz) and $^{13}$C (126 MHz), in $CDCl_3$.

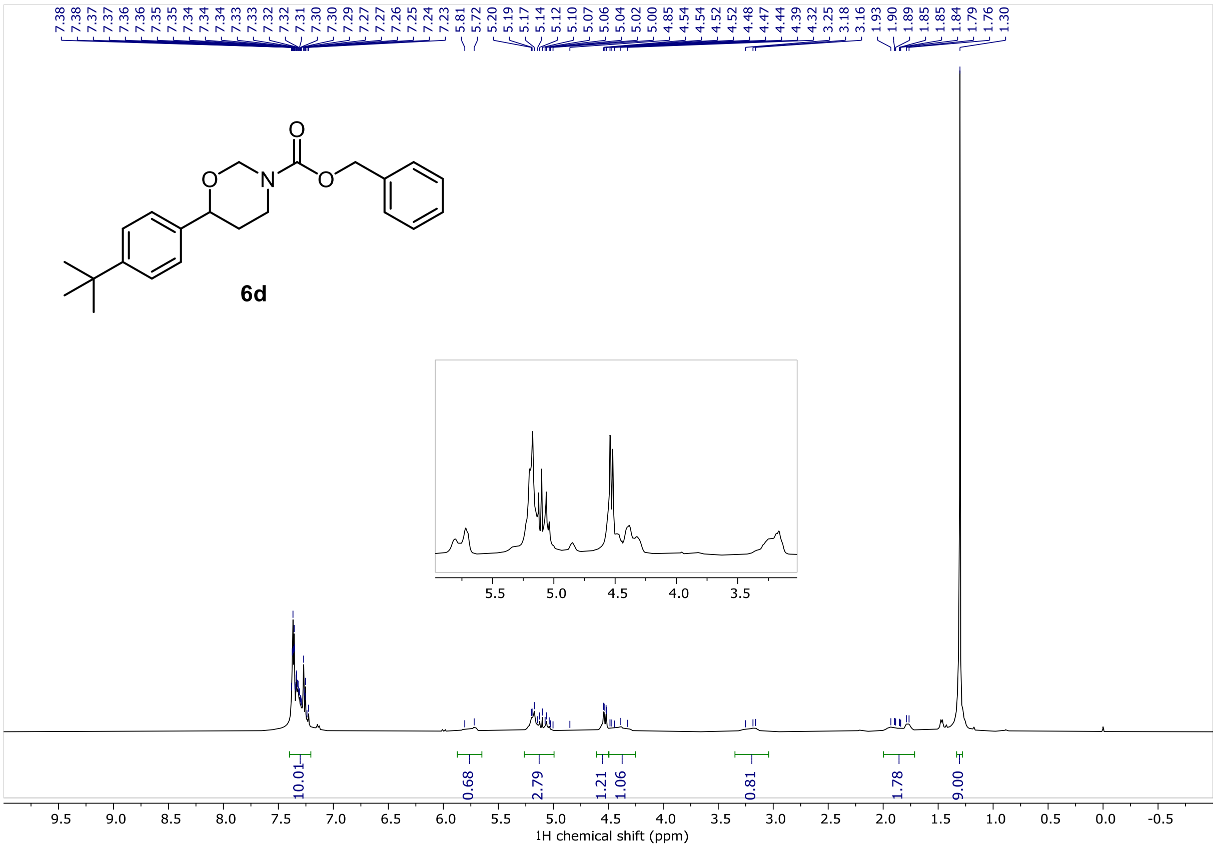


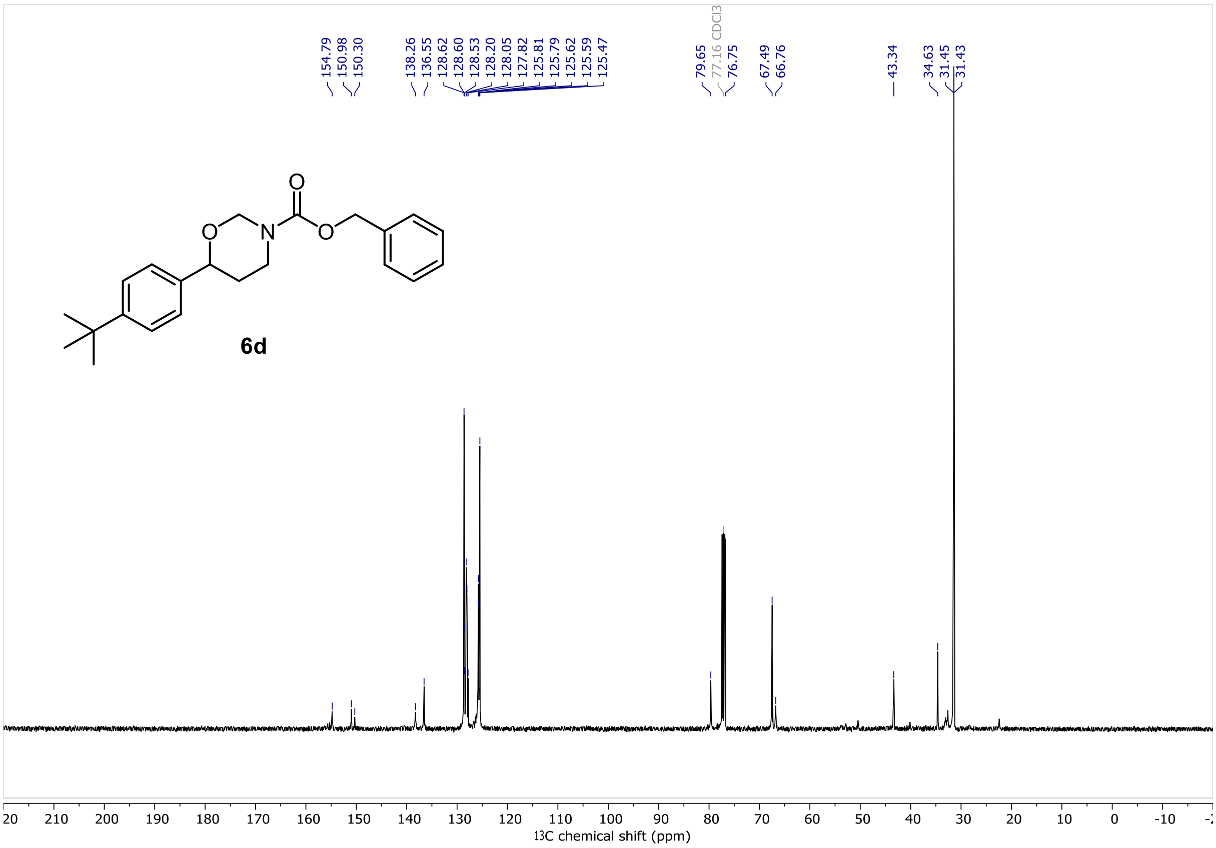


NMR spectra for compound **6d**: $^{1}H$ (501 MHz) and $^{13}C$ (126 MHz), in $CDCl_3$.

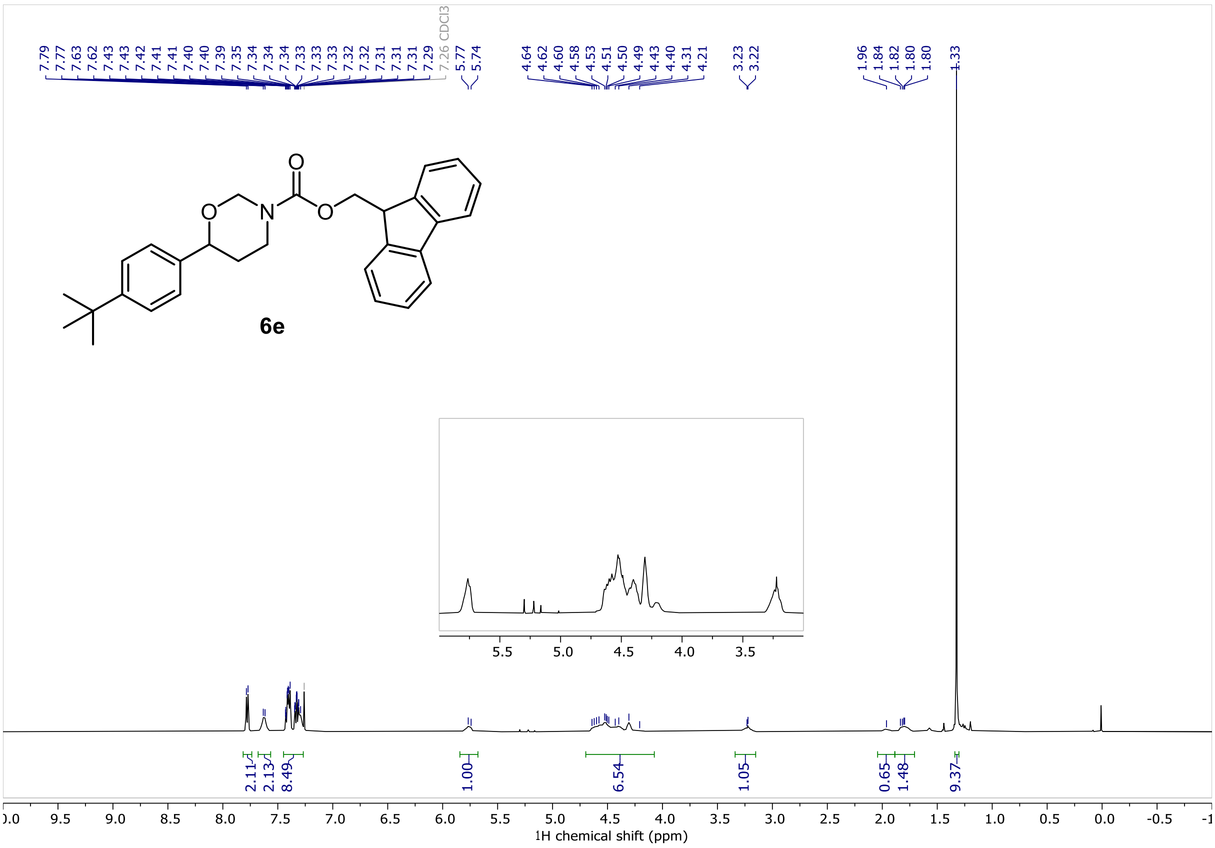

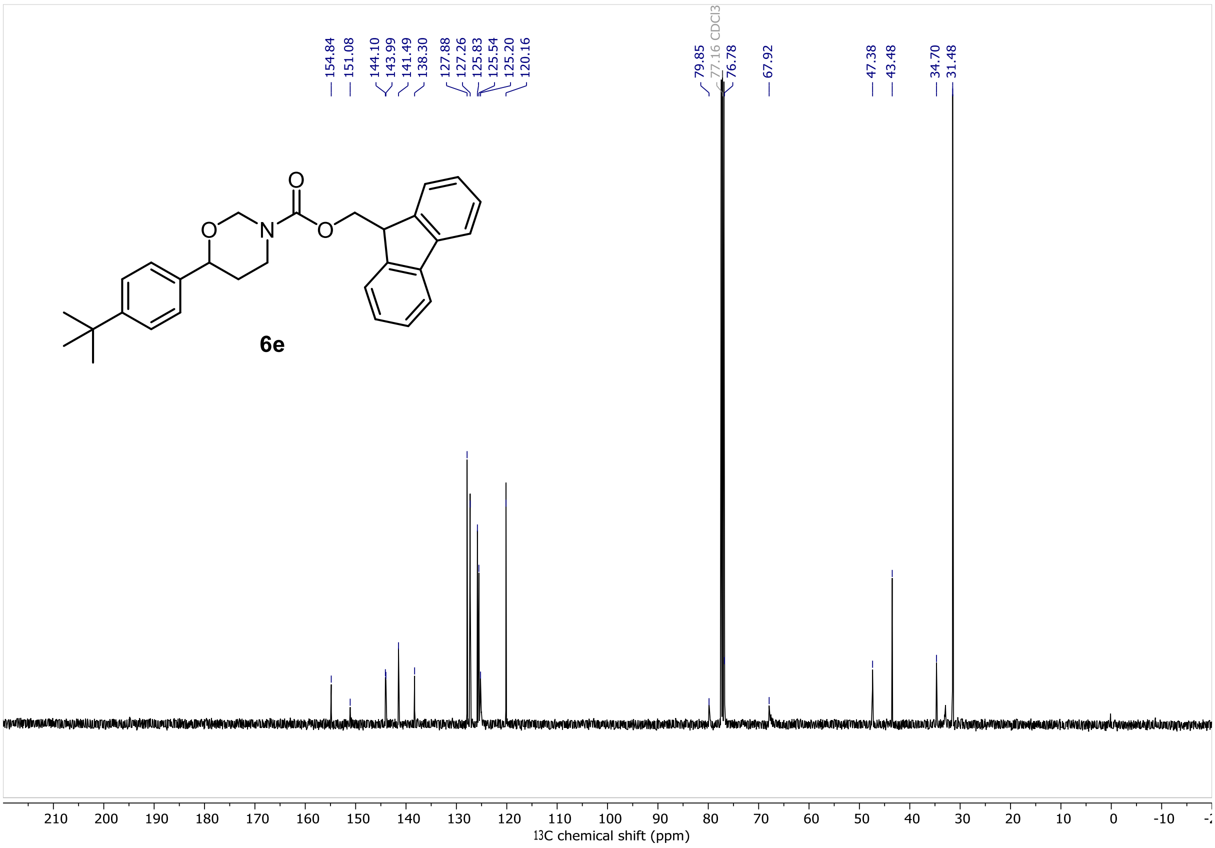

NMR spectra for compound **6e**: $^{1}$H (501 MHz) and $^{13}$C (126 MHz), in $CDCl_3$.

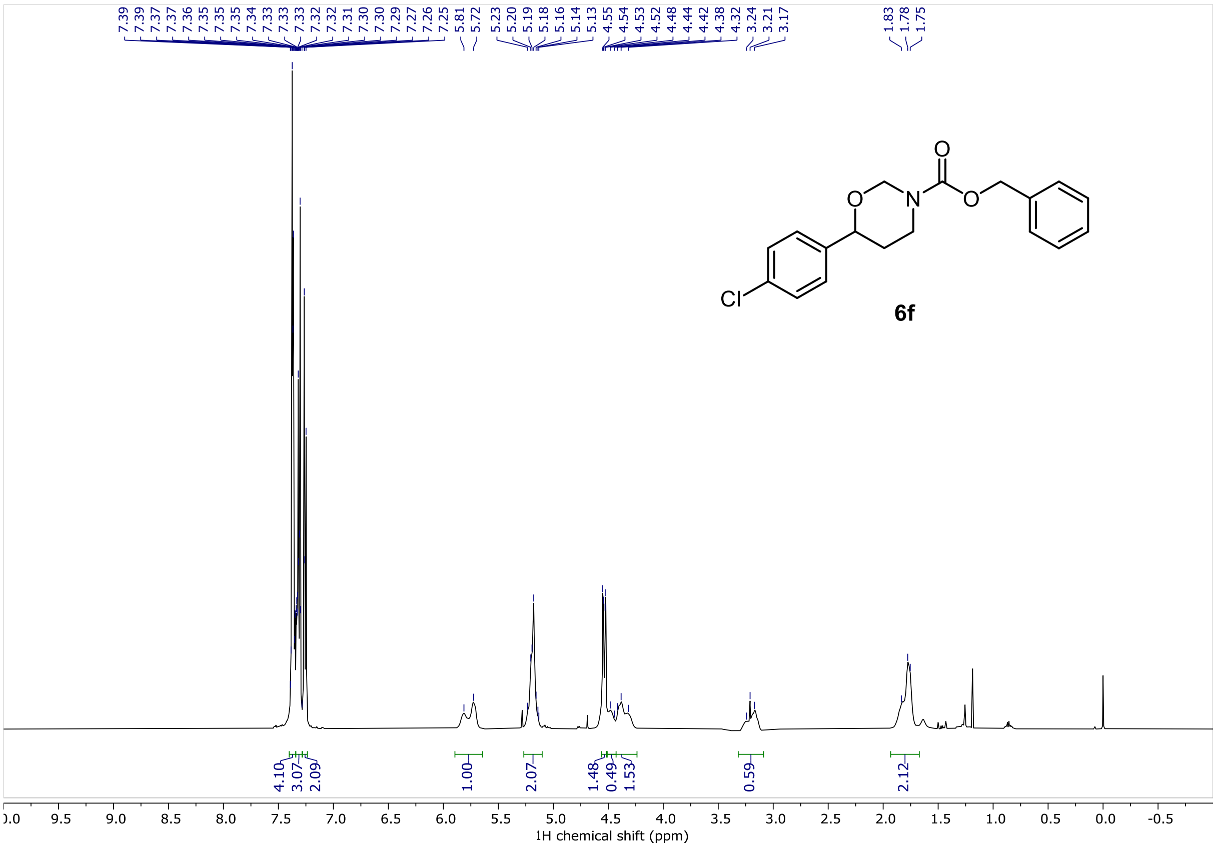


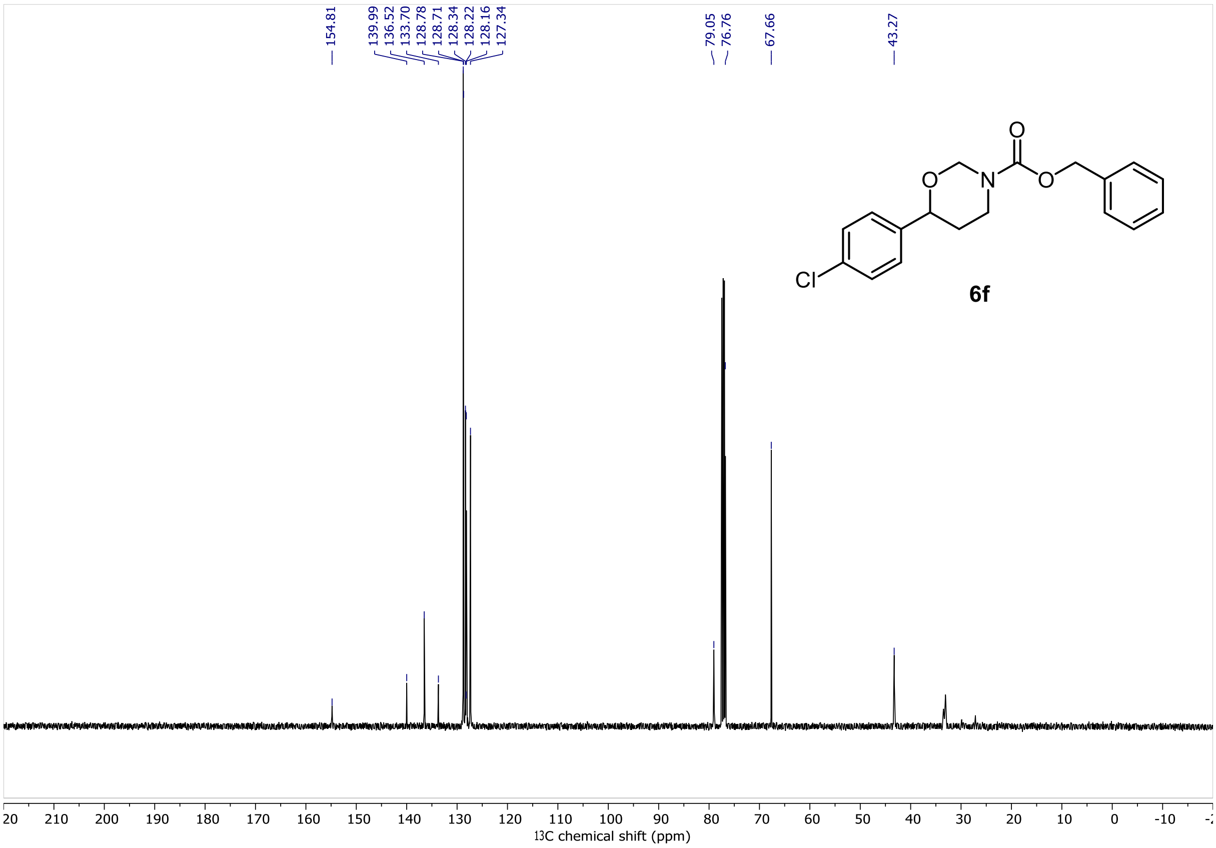


NMR spectra for compound **6f**: $^{1}$H (501 MHz) and $^{13}$C (126 MHz), in $CDCl_3$.

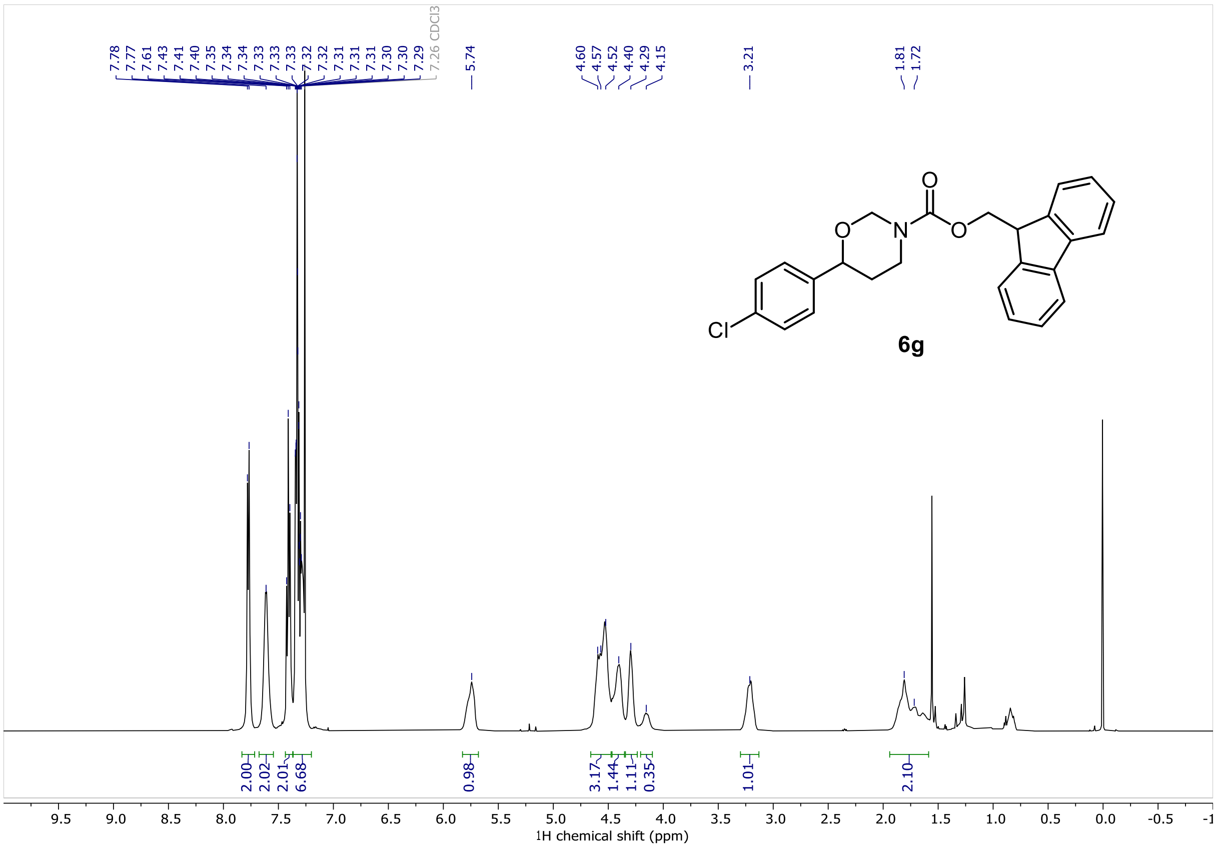

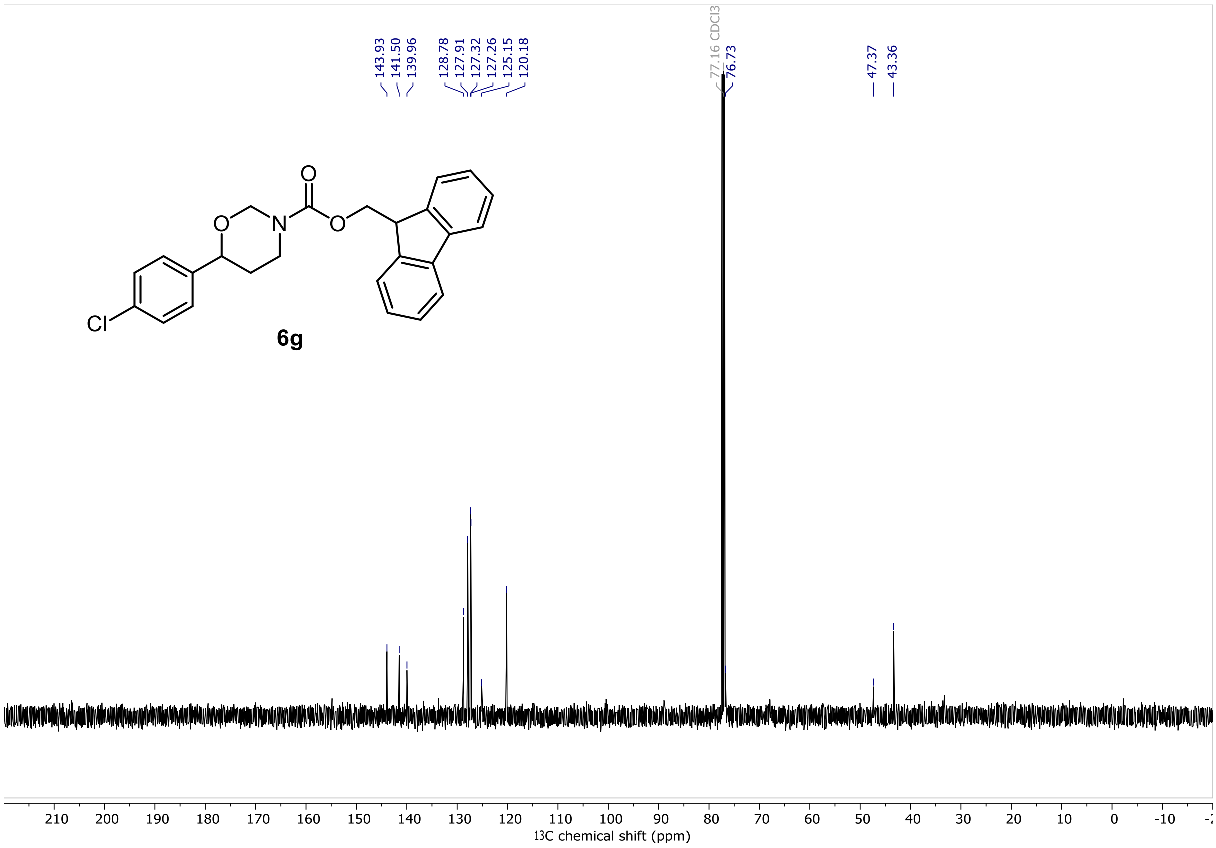

NMR spectra for compound **6g**: $^{1}H$ (501 MHz) and $^{13}C$ (126 MHz), in $CDCl_3$.

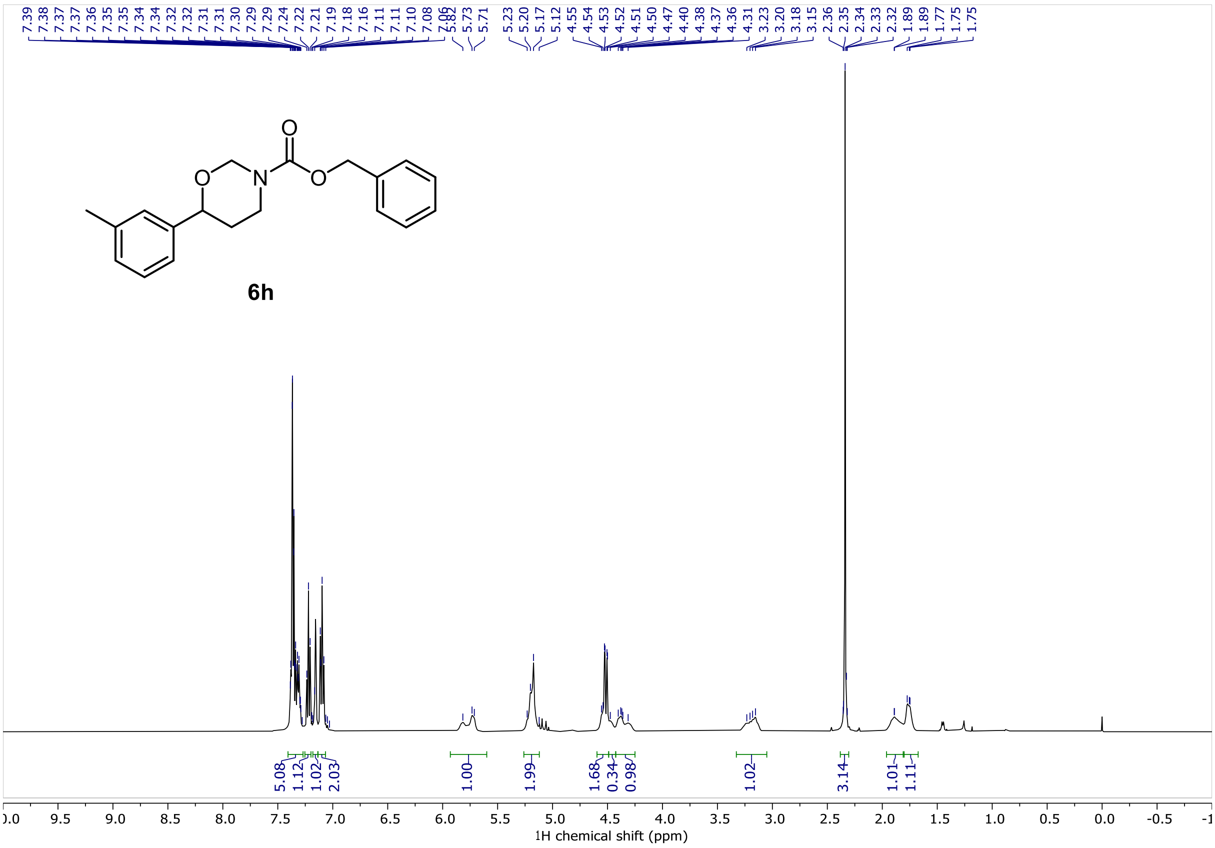

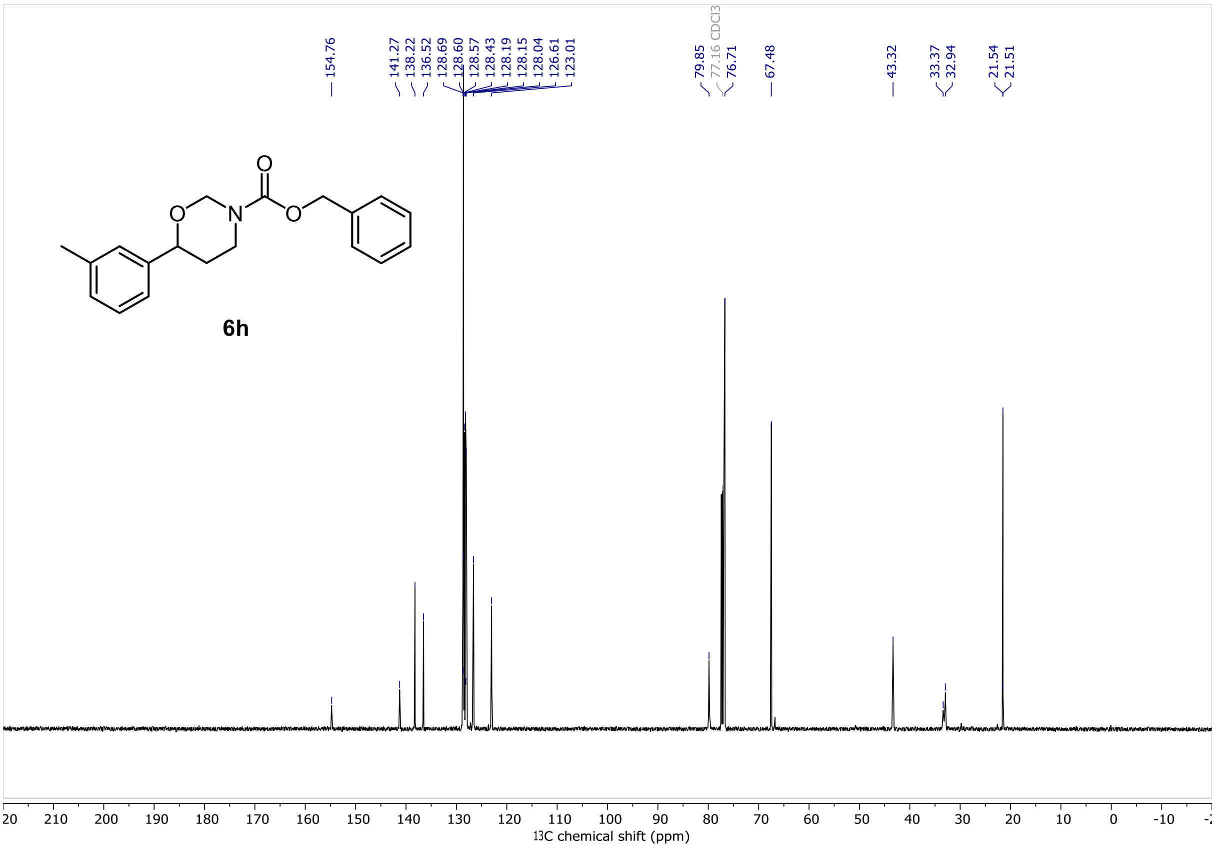

NMR spectra for compound **6h**: $^{1}$H (501 MHz) and $^{13}$C (126 MHz), in $CDCl_3$.

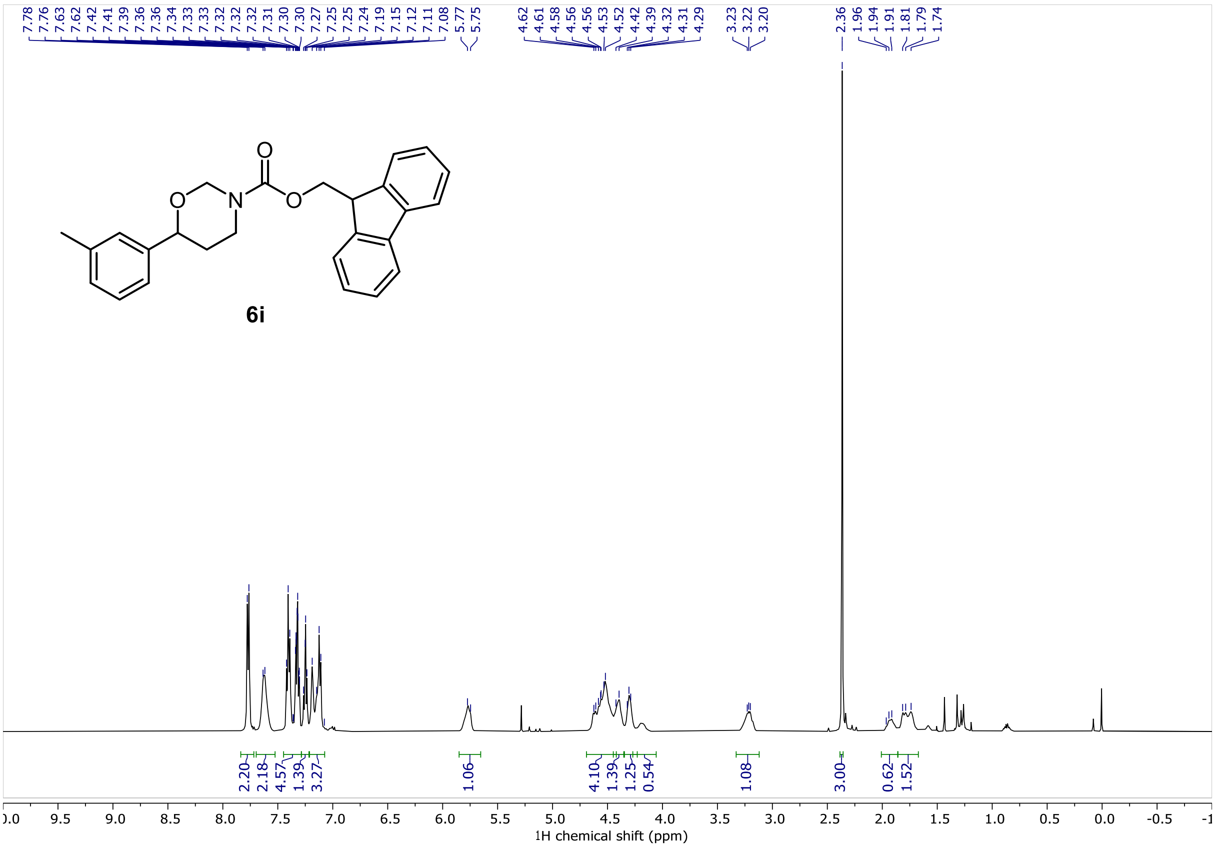

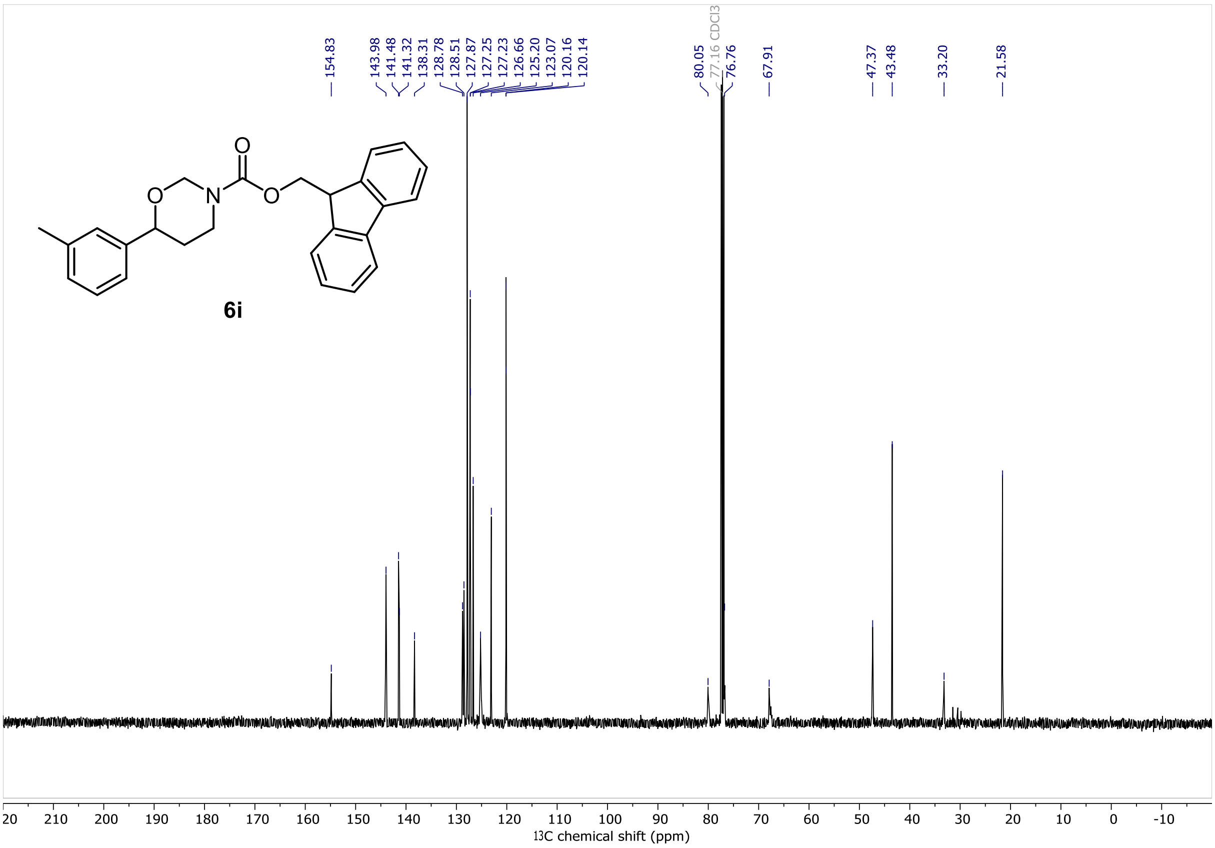

NMR spectra for compound **6i**: $^{1}H$ (501 MHz) and $^{13}C$ (126 MHz), in $CDCl_3$.

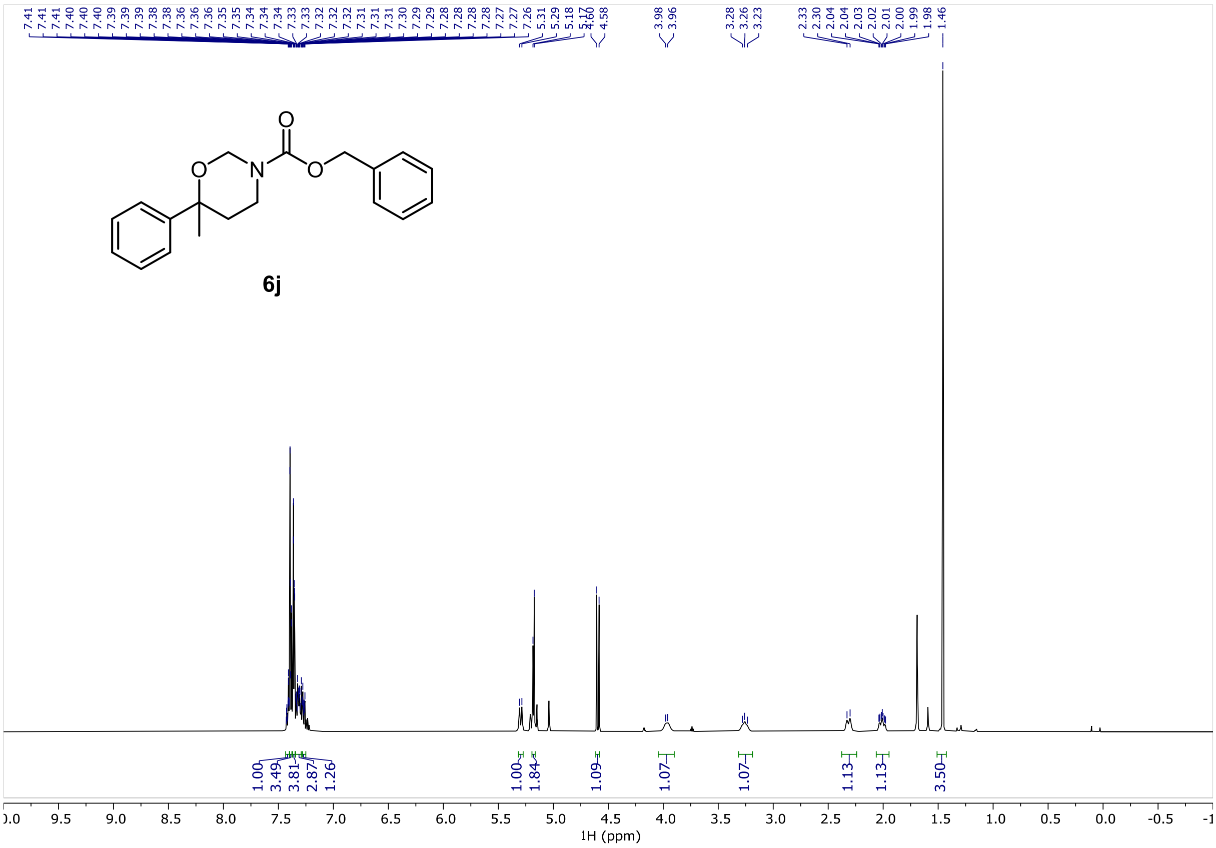

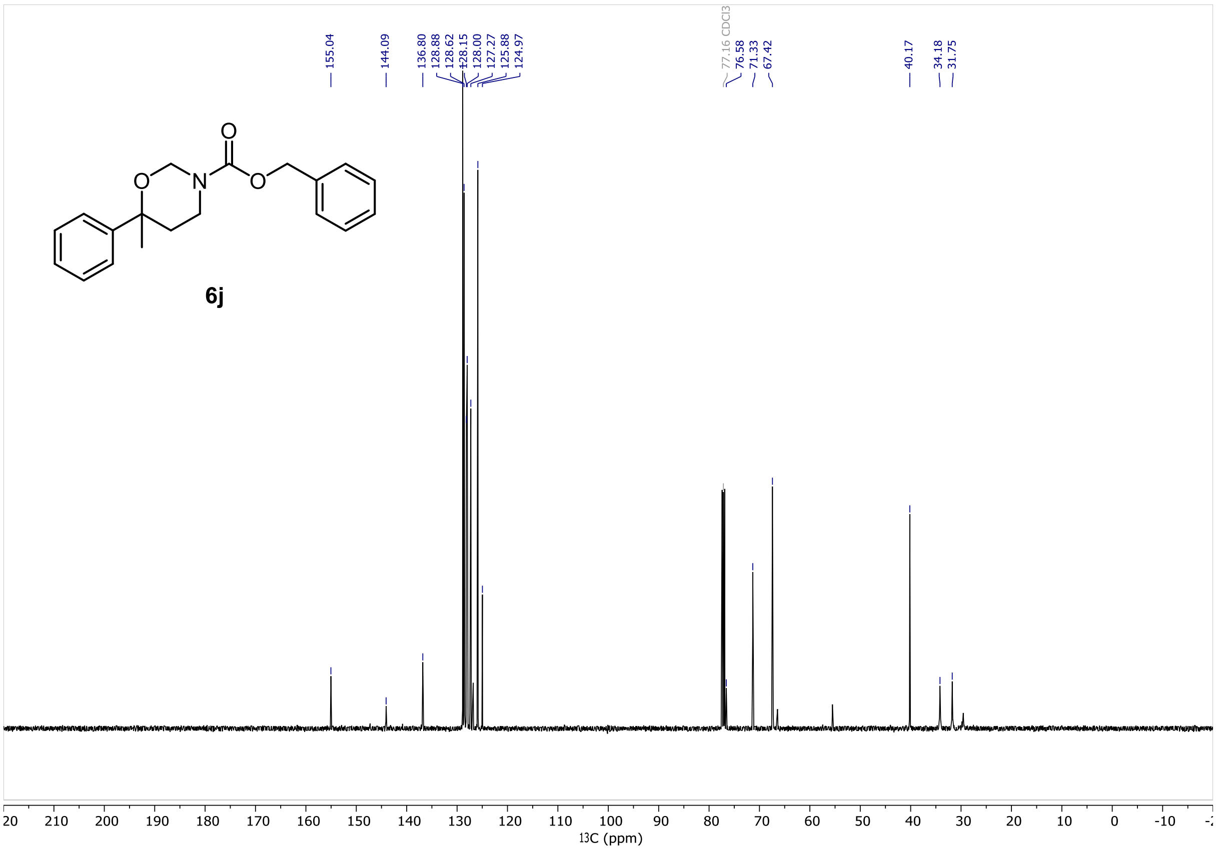

NMR spectra for compound **6j**: $^{1}$H (501 MHz) and $^{13}$C (126 MHz), in $CDCl_3$.

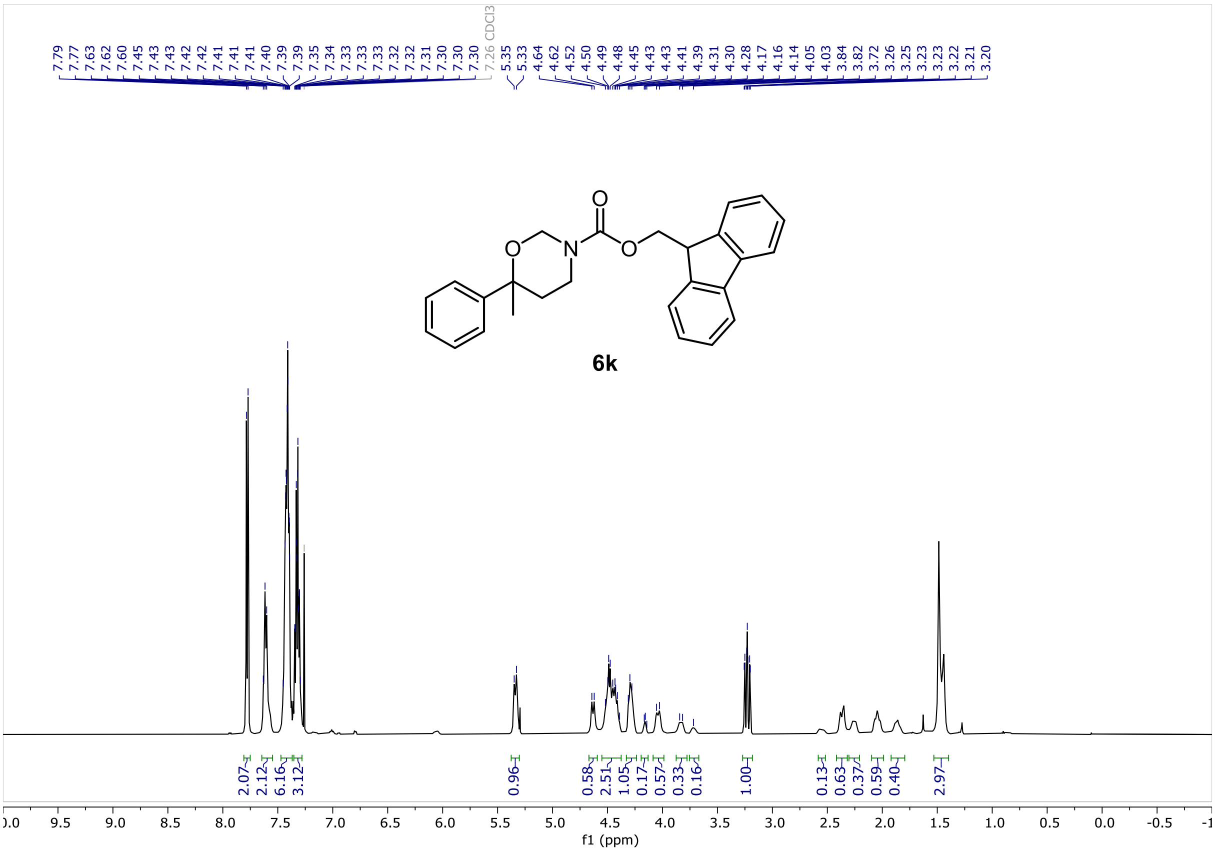

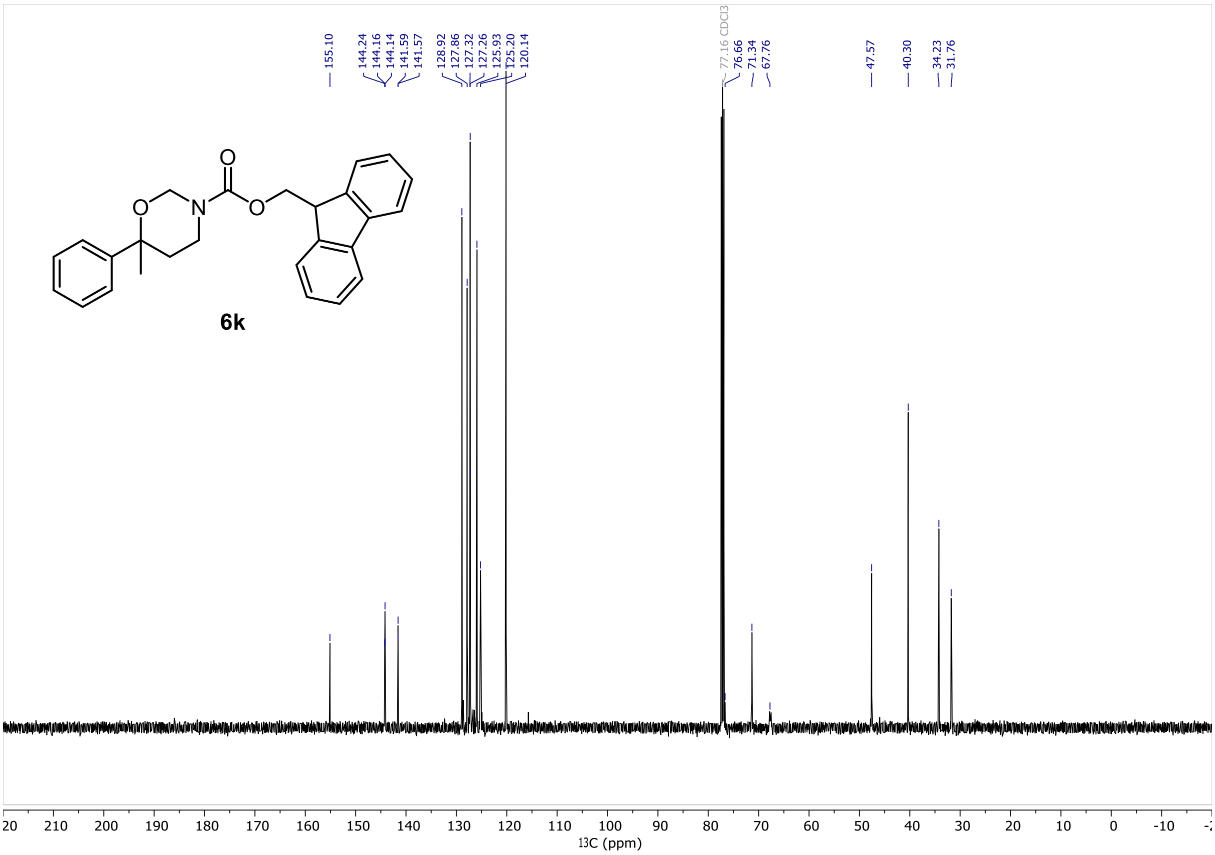

NMR spectra for compound **6k**: $^{1}H$ (501 MHz) and $^{13}C$ (126 MHz), in $CDCl_3$.

# 9. References


1. Armarego, W. L. F., *Purification of laboratory chemicals*. 8th ed.; Butterworth-Heinemann: Woburn, MA, 2003.
2. Harris, R. K.; Becker, E. D.; Cabral de Menezes, S. M.; Granger, P.; Hoffman, R. E.; Zilm, K. W., Further conventions for NMR shielding and chemical shifts (IUPAC Recommendations 2008). *Pure Appl. Chem.* **2008,** *80* (1), 59–84.
3. Díaz-Oviedo, C. D.; Maji, R.; List, B., The Catalytic Asymmetric Intermolecular Prins Reaction. *Journal of the American Chemical Society* **2021,** *143* (49), 20598–20604.
4. Kohler, D. G.; Gockel, S. N.; Kennemur, J. L.; Waller, P. J.; Hull, K. L., Palladium-Catalysed Anti-Markovnikov Selective Oxidative Amination. *Nature Chemistry* **2018,** *10* (3), 333–340.
5. Orazi, O. O.; Corral, R. A., Cyclic Products from Sulphonamides and Formaldehyde. *J. Chem. Soc. Perkin Trans. 1* **1975,** (8), 772–774.
6. Ni, Y.; Zuo, H.; Yu, H.; Wu, Y.; Zhong, F., Synergistic Catalysis-Enabled Thia-Aza-Prins Cyclization with DMSO and Disulfides: Entry to Sulfenylated 1,3-Oxazinanes and Oxazolidines. *Org. Lett.* **2018,** *20* (18), 5899–5904.
7. Feng, X.; Qiu, G.; Liang, S.; Su, J.; Teng, H.; Wu, L.; Hu, X., Efficient Synthesis of Chiral β-and γ-*N*-Tosylaminoalcohols from 1-Aryl-2-aminopropane-1,3-diols. *Russ. J. Org. Chem+* **2006,** *42* (4), 496–500.
8. Viswambharan, B.; Okimura, T.; Suzuki, S.; Okamoto, S., Synthesis and Catalytic Properties of 4-Aryl-2,3-dihydro-4*H*-pyrimido[2,3-*b*]benzothiazoles for Asymmetric Acyl or Carboxyl Group Transfer Reactions. *J. Org. Chem.* **2011,** *76* (16), 6678–6685.
9. Facchetti, G.; Gandolfi, R.; Fusè, M.; Zerla, D.; Cesarotti, E.; Pellizzoni, M.; Rimoldi, I., Simple 1,3-Diamines and their Application as Ligands in Ruthenium(II) Catalysts for Asymmetric Transfer Hydrogenation of Aryl Ketones. *New J. Chem.* **2015,** *39* (5), 3792–3800.
10. Song, J.; Zheng, W.-H., A Highly Enantioselective Approach towards Optically Active γ-Amino Alcohols by Tin-Catalyzed Kinetic Resolution of 1,3-Amino Alcohols. *Chem. Comm.* **2022,** *58* (53), 7392–7395.
11. Mathieu, G.; Patel, H.; Lebel, H., Convenient Continuous Flow Synthesis of *N*-Methyl Secondary Amines from Alkyl Mesylates and Epoxides. *Org. Process Res. Dev.* **2020,** *24* (10), 2157–2168.
12. McMaster, L., Condensations of Some Toluenesulfonamides with Trioxymethylene and with Formaldehyde Solution. *J. Am. Chem. Soc.* **1934,** *56* (1), 204–206.
13. Egginton, C. D.; Lambie, A. J., Reaction between Toluene-*p*-sulphonamide and Formaldehyde. *J. Chem. Soc. C* **1969,** (12), 1623–1625.
14. Meshcheryakov, V. I.; Moskalik, M. Y.; Starke, I.; Shainyan, B. A., Condensation of Trifluoromethanesulfonamide with Paraformaldehyde and Oxamide. *Russ. J. Org. Chem+* **2010,** *46* (10), 1471–1475.
